# Design, Synthesis, and Biological Evaluation of a Series of 5- and 7-Hydroxycoumarin Derivatives as 5-HT_1A_ Serotonin Receptor Antagonists

**DOI:** 10.3390/ph14030179

**Published:** 2021-02-24

**Authors:** Kinga Ostrowska, Anna Leśniak, Zuzanna Czarnocka, Jagoda Chmiel, Magdalena Bujalska-Zadrożny, Bartosz Trzaskowski

**Affiliations:** 1 Department of Organic Chemistry, Faculty of Pharmacy, Medical University of Warsaw, 1 Banacha Str., 02-097 Warsaw, Poland; zuzanna.czarnocka@gmail.com (Z.C.); jagodachmiel170896@gmail.com (J.C.); 2 Department of Pharmacodynamics, Faculty of Pharmacy, Centre for Preclinical Research and Technology, Medical University of Warsaw, 1 Banacha Str., 02-097 Warsaw, Poland; anna.lesniak@wum.edu.pl (A.L.); Magdalena.bujalska@wum.edu.pl (M.B.-Z.); 3 Centre of New Technologies, University of Warsaw, 2C Banacha Str., 02-097 Warsaw, Poland; b.trzaskowski@cent.uw.edu.pl

**Keywords:** molecular docking, microwave-assisted synthesis, hydroxycoumarin derivatives, 5-HT_1A_, 5-HT_2A_, receptors ligands, CNS activity

## Abstract

We have designed and synthesized a series of 60 new 5- and 7-hydroxycoumarin derivatives bearing the piperazine moiety with the expected binding to 5-HT_1A_ and 5-HT_2A_ receptors. Molecular docking of all investigated compounds revealed subnanomolar estimates of 5-HT_1A_R K_i_ for three ligands and 5-HT_2A_R Ki for one ligand as well as numerous low nanomolar estimates of K_i_ for both receptors. Intrigued by these results we synthesized all 60 new derivatives using microwave-assisted protocols. We show that three new compounds show a relatively high antagonistic activity against the 5HT_1A_ receptor, although lower than the reference compound WAY-100635. These compounds also showed relatively low binding affinities to the 5-HT_2A_ receptor. We also provide a detailed structure–activity analysis of this series of compounds and compare it with previously obtained results for an exhaustive series of coumarin derivatives.

## 1. Introduction

N-arylpiperazine-containing ligands are a large class of chemical compounds with various known biological activities, such as enzyme inhibition, antibacterial, antineoplastic, and anticancer properties, as well as adrenergic and serotonin receptor inhibition [1,2,3,4,5,6,7]. This last activity is particularly prominent for this family of compounds, as even some of its simplest members, such as 1-(3-chlorophenyl) piperazine or m-trifluoromethylphenylpiperazine, are known to interact with serotonin receptors [4,8]. The high affinity of these systems to 5HT receptors stems from the highly basic nitrogen atom of the piperazine, which is able to form strong interactions with the conserved acidic amino acids in the GPCR transmembrane domain of these proteins [9]. In order to be effective as 5HT receptor antagonists or agonists, such compounds require in their structure, however, also a relatively bulky moiety connected usually to the N-arylpiperazine via a flexible aliphatic linker. Such a design principle has been first realized in buspirone, a 5-HT_1A_ receptor agonist, which also has moderate activity against other 5HT receptors and selected dopamine receptors, and is followed until today with the goal of finding new agonists/antagonists of 5HT receptors [10,11,12]. 5HT receptors proteins modulate the release of many neurotransmitters, therefore are an important target for a variety of drugs, including antipsychotics, antidepressants, hallucinogens anorectics, and antimigraine agents [13,14,15,16].

**Figure 1:**
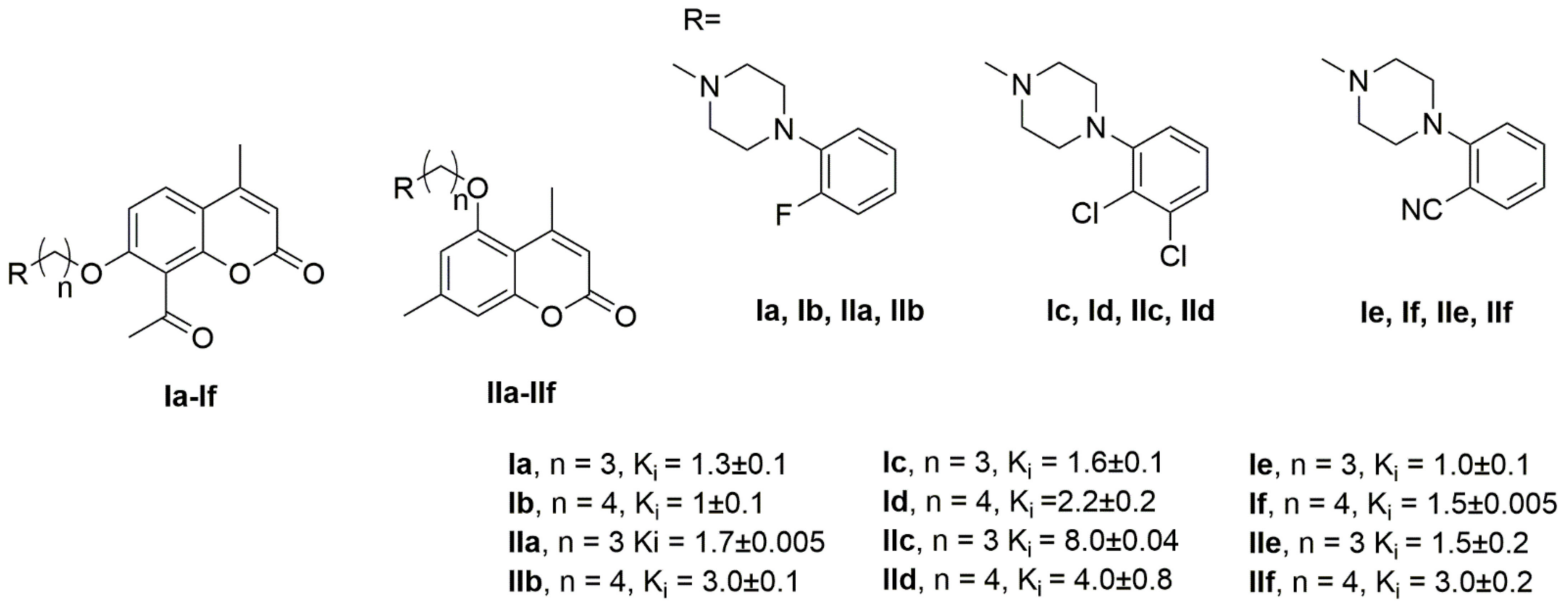
Selected 5-HT_1A_ receptor affinities of previously synthesized coumarins derivatives.

Among many groups considered as the bulky moiety connected to N-arylpiperazine coumarin derivatives have gained some attention, particularly after the investigations of Chen et al., who showed that selected N-arylpiperazines connected to coumarins in position 7 via a (CH_2_)_4_ linker have nanomolar Ki values toward 5-HT_1A_ and 5-HT_2A_ receptors [17,18]. Inspired by these works we have expanded the family of potential serotonin agonists/antagonists based on the same design principle by introducing different arylpiperazine derivatives of 7-hydroxycoumarin, some of which showed subnanomolar affinities to 5-HT_1A_ receptor and low nanomolar affinities to 5-HT_2A_ receptor [19,20]. Later we have also obtained a series of arylpiperazine derivatives of 5-hydroxycoumarin [21,22,23]. We showed that the highest, subnanomolar affinities for 5-HT_1A_ receptor were associated with the presence of the acetyl group in the C-6 position at the coumarin ring and the substituents in the 2 or 3 position in the phenyl ring of piperazine. Finally, in 2020 we designed a new series of arylpiperazinyl derivatives of 6-acetyl-5-hydroxy-4,7-dimethylcoumarin, which also showed very low nanomolar affinities toward 5HT_1A_ and 5HT_2A_ but also low affinities to the D_2_ receptor [24]. In these studies we noticed that the length of the alkyl linker (three-carbon versus four-carbon) had little impact on the obtained Ki values, since the affinities for specific serotonin receptors for analogous compounds containing the same arylpiperazinyl fragments, differing only in the length of the alkyl linker, were very similar. It is worth noting that this finding is not based on single cases but on a large number of cases, showing a clear tendency for this particular length of the linker (Figure 1).

To conclude our search for new biologically active compounds in this series as well as to gain even more knowledge of the structure–activity relationships we have designed two new series of arylpiperazine derivatives of 5-hydroxycoumarins and 7-hydroxycoumarins. These series were designed based on the aryl substituents giving the highest affinities in our previous studies, but with different lengths of the alkyl linkers, consisting of either two or five CH_2_ moieties. In this study we have used molecular docking to homology models of 5-HT_1A_ and 5-HT_2A_ receptors followed by microwave-assisted protocols to synthesize all 60 compounds. We also performed functional activity studies for the 5-HT_1A_ receptor, as well as 5-HT_2A_ receptor affinity studies.

## 2. Results and Discussion

### 2.1. Docking Studies

The results of the Ki estimates obtained from the computational studies are presented in Table 1 and Table 2. While for the starting compounds 1–6 the Ki values were estimated at 56–922 nM for 5-HT_1A_ and above 1 μm for 5-HT_2A_ receptor, some of the functionalized derivatives show nanomolar or even subnanomolar affinities. In particular, there are three new compounds with the estimated Ki versus 5-HT_1A_ receptor below 1 nm (**1a**, **6b**, and **6g**) and one for 5-HT_2A_ receptor (**5j**). As there are many other compounds with the estimated Ki below 10 nM we decided to synthesize all of these systems to verify their 5-HT_1A/2A_ receptor affinities. We also decided to perform a detailed analysis of the predicted binding poses for 5-HT_1A_ receptor, as the Ki values for the 5HT_2A_ receptor are usually less favorable and our previous studies showed that this class of compounds in most cases binds stronger to 5-HT_1A_ receptor than to 5-HT_2A_ receptor [19,20,21,22,23,24].

**Figure 2:**
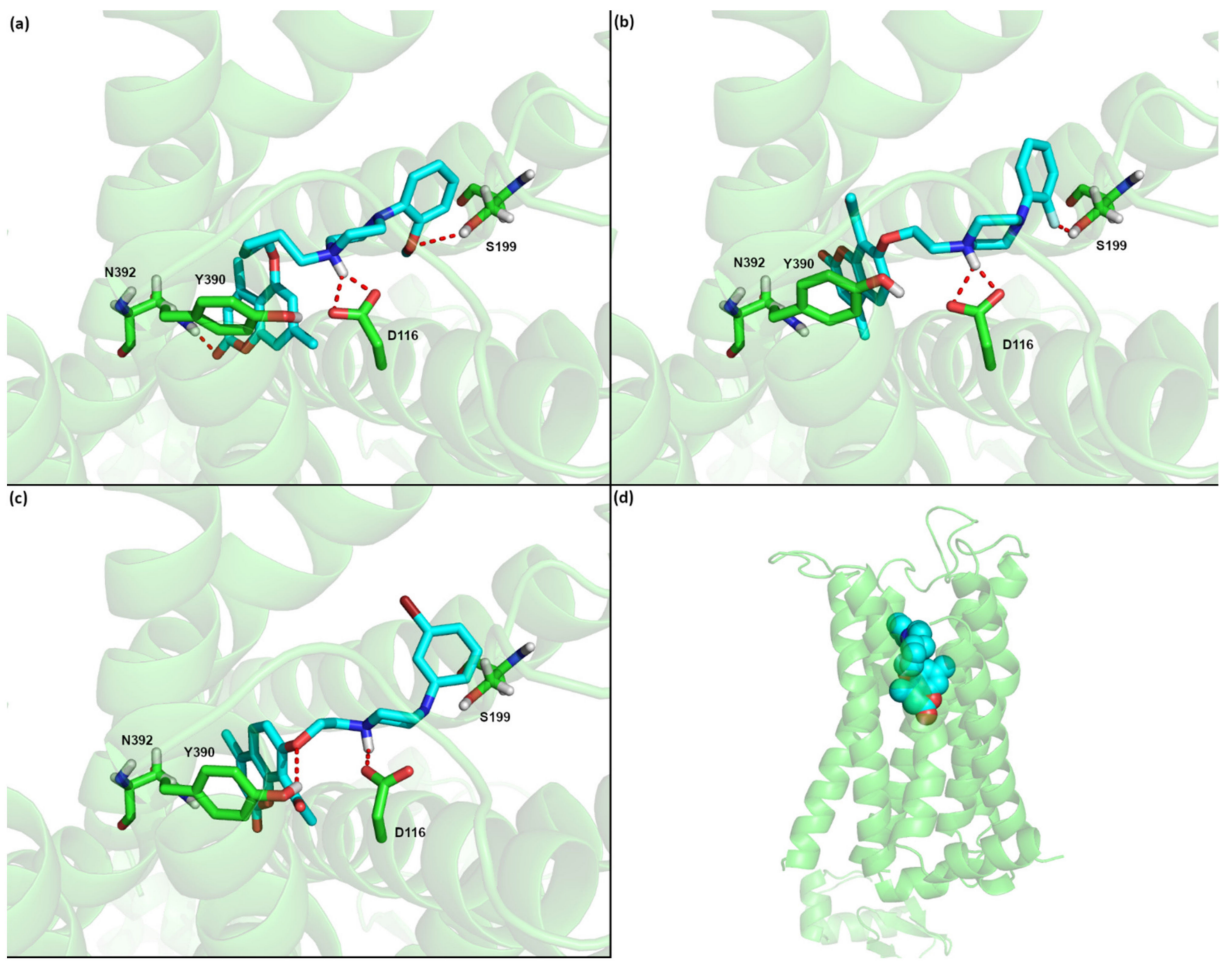
Predicted binding poses for the 5HT_1A_ receptor and compounds (**a**) **1a**, (**b**) **6b,** and (**c**) **6g**, and (**d**) the location of the binding site in the GPCR. Nonpolar hydrogen atoms were omitted for clarity.

Of the three compounds predicted to have subnanomolar affinities to 5-HT_1A_ receptor (**1a**, **6b**, **6g**) all have the crucial interaction between the basic nitrogen atom of the piperazine group with the conserved D116 of the receptor binding site (see Figure 2). As one can see the poses of these three systems are also very similar, with the coumarin part going deep into the binding pocket, toward transmembrane (TM) helix 7 and the arylpiperazine extending toward transmembrane helix 4. Apart from the salt bridge to D114 from transmembrane helix 3, **1a** is predicted to form also hydrogen bonds between the methoxy moiety of the arylpiperazine and S199 (TM4) as well as between the coumarin part and N392 (TM7). On the other hand **6b** is predicted to be additionally stabilized by the interaction between the F atom of the arylpiperazine and S199 (TM4), while **6g** by the hydrogen bond between the oxygen atom of the linker and Y390 (TM7). It is also worth mentioning that these poses are similar to our previously predicted poses for the coumarin derivatives with three CH_2_ moieties. On the other hand for some derivatives with four CH_2_ groups we predicted a different orientation of the ligand, where the coumarin part extends toward TM helix 4, while the arylpiperazine part goes deep into the pocket and interacts with the residues located on TM7 [24].

### 2.2. Chemistry

**Scheme 1:**
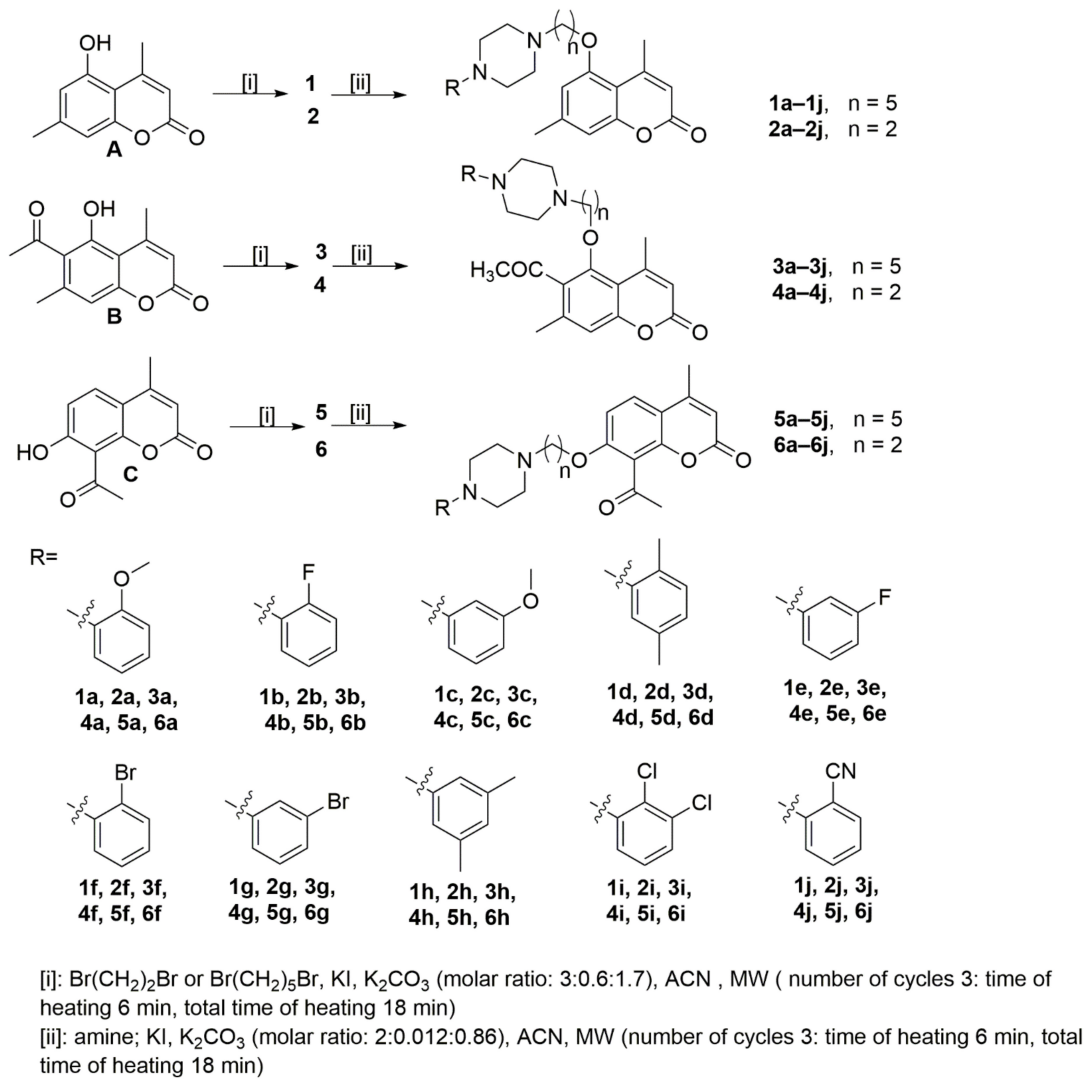
Synthesis of compounds investigated in this work.

The starting coumarins 5-hydroxy-4,7-dimethylchromen-2-one (**A**), 6-acetyl-5-hydroxy-4,7-dimethylchromen-2-one (**B**), and 8-acetyl-7-hydroxy-4-methylchromen-2-one (**C**) were resynthesized according to previously published studies [25,26]. The reaction of starting coumarins (**A–C**) with 1,2-dibromoethane in acetonitrile in the presence of potassium iodide and potassium carbonate afforded with different yields (25–80%) 5-(2-bromo ethoxy)-4,7-dimethyl-2H-chromen-2-one (**2**), 6-acetyl-5-(2-bromoethoxy)-4,7-dimethyl-2H-chromen-2-one (**4**) and 8-acetyl-7-(2-bromoethoxy)-4-methylchromen-2-one (**6**), while upon reaction of 1,5-dibromopentane, in the same conditions, 5-(5-bromopentyloxy)-4,7-dimethyl-2H-chromen-2-one (**1**), 6-acetyl-5-(5-bromopentyloxy)-4,7-dimethyl-2H-chromen-2-one (**3**) and 8-acetyl-7-(5-brompenthoxy)-4-methylchromen-2-one (**5**) were obtained with different yields (44–89%). In the next step, the final compounds were synthesized as pictured in Scheme 1 and according to the previously published study [19]. The synthesis of compounds **1a**–**1j**, **2a**–**2j**, **3a**–**3j**, **4a**–**4j**, **5a**–**5j,** and **6a**–**6j** was carried out by reacting the bromoalkyl derivatives (**1**–**6**) with appropriate arylpiperazine: (4-(2-methoxyphenyl)piperazine, (4-(2-fluorophenyl) piperazine, (4-(3-methoxyphenyl)piperazine, (4-(2,5-dimethylyphenyl)piperazine, (4-(3-fluorophenyl)piperazine, (4-(2-bromophenyl)piperazine, (4-(3-bromophenyl) piperazine, (4-(3,5-dimethylphenyl)piperazine, (4-(2,3-dichlorophenyl)piperazine, (4-(2-cyano phenyl)piperazine) in acetonitrile and in the presence of potassium iodide and potassium carbonate. Reaction progress was monitored by TLC using silica gel plates (eluent: CHCl3: MeOH; 10:0.25). All compounds synthesized in this work were obtained using a microwave reactor and were purified by column chromatography using silica gel and CHCl_3_:MeOH (100:1) as the eluent, as in the previously published study [19]. All syntheses were performed in the millimolar scale, starting from 1 mmol of the starting coumarin derivatives **1**–**6**, and the final yields of the products were in the 43–98% range. All compounds were fully characterized using standard methods, ^1^H NMR, ^13^C NMR spectroscopy, and HRMS spectrometry. All NMR spectra are presented in the Supplementary Materials.

### 2.3. Biological Evaluation

#### 2.3.1. 5-HT_1A_ Receptor Activity

After purification via column chromatography, all newly synthesized compounds were subjected to in vitro evaluation of their functional activity for the 5-HT_1A_ receptor, as well as 5-HT_2A_ receptor affinity studies. Since in our previous study similar coumarin derivatives showed high affinities to 5-HT_1A_ receptor and low to 5-HT_2A_ receptor we decided to employ in this study a functional assay to establish the potency and efficacy of 5-HT_1A_ binding of our series of compound. The major advantage of this approach over determining only receptor affinity is the ability to predict intracellular consequences of receptor binding, leading to either receptor activation, blockage, or alteration of constitutive activity. Moreover, measures of affinity may not correspond to drug potency, owing to the possible existence of a receptor reserve [27]. Also, for ligands displaying functional bias, measuring one distinctive activation pattern allows for the prediction of the therapeutic usefulness of the drug in question [28]. Thus, functional characteristics are of major importance for any drug discovery program which strives for in vivo evaluation of compound activity. For the 5-HT_2A_ receptor we expected, on the hand, low affinities and decided to perform standard receptor affinity studies.

As shown in Table 3, arylpiperazinyl derivatives of coumarin displayed varied selectivity for 5-HT_1A_ receptor with respect to WAY-100635, a reference compound which is a piperazine drug that acts as a selective 5-HT_1A_ receptor antagonist. The highest activity was found for compounds **1a**, **3a**, **4a**, **5a,** and **5b** with the following values: **1a** (EC_50_ = 29.4 ± 7.3 nM) > **5a** (EC_50_ = 30.5 ± 2.56 nM) > **3a** (EC_50_ = 39.4 ± 3.63 nM) > **5b** (EC_50_ = 82 ± 13.4 nM) > **4a** (EC_50_ = 91.6 ± 13.3 nM). Compounds **2b**, **2d**, **2f**, **2h,** and **2**j did not show any activity and four compounds **1d**, **1g**, **3j,** and **6g** were not tested due to a very poor solubility under experimental conditions. The remaining coumarin derivatives showed moderate to low activity ranging from EC_50_ = 527 ± 191 nM for compound **4e**, to EC_50_ = 365,800 ± 46,480 nM for compound **2i**.

The structure–activity studies revealed that the presence of a (2-methoxyphenyl) piperazine moiety and a five carbon linker (**1a**, **3a**, **4a**, **5a**) was the most beneficial for 5-HT_1A_ antagonistic activity. This was a trend independent of the starting coumarin derivative, as a high antagonistic activity was obtained for 5-hydroxy-4,7-dimethylchromen-2-one derivative (**1a**), 6-acetyl-5-hydroxy-4,7-dimethylchromen-2-one (**3a**), and 8-acetyl-7-hydroxy-4-methylchromen- 2-one (**5a**). Only one compound with a two carbon linker, namely **4a** (6-acetyl-5-hydroxy-4,7-dimethylchromen-2-one), showed a similarly high level of activity. Also, a high activity was found for one derivative bearing a (2-fluorophenyl) piperazine moiety, 8-acetyl-7-hydroxy-4-methylchromen-2-one (**5b**).

In the family of 5-hydroxy-4,7-dimethylchromen-2-one (**A**) derivatives, compounds with a five carbon linker were much more active than those with a two carbon linker. Comparing the systems containing the same piperazinyl part within this family, we can see that the five carbon linker derivatives have always a higher activity than the one with two CH_2_ moieties, e.g., EC_50_ = 29.4 ± 7.3 nM for **1a** and EC_50_ = 1881 ± 427 nM for **2a**; EC_50_ = 980 ± 207 nM for **1b** and no activity for **2b**; EC_50_ = 1698 ± 358 nM for **1c** and EC_50_ = 19130 ± 2363 nM for **2c**, etc. For 6-acetyl-5-hydroxy-4,7-dimethylchromen-2-one (**B**) derivatives, which differ from the **A** family in the presence of an additional acetyl group at the position C-6 of the coumarin ring, derivatives with the five-carbon linker had higher activity than those with the two-carbon linker, when they contained 2-methoxyphenyl (see **3a** and **4a**), 3-methoxyphenyl (see **3c** and **4c**), 2,5-dimethylyphenyl (see **3d** and **4d**), 3-bromophenyl (see **3g** and **4g**), or 2,5-dimethylyphenyl moiety (see **3h** and **4h**). On the other hand, derivatives with the two-carbon linker showed higher activity than those with the five-carbon linkers, when they contained the 2-fluorophenyl (see **3b** and **4b**), 3-fluorophenyl (see **3e** and **4e**), 2-bromophenyl (see **3f** and **4f**), or 2,3-dichlorophenyl moiety (see **3i** and **4i**). Such a difference may stem from the fact that a longer alkyl linker may maximize the interactions of the ligand with the receptor’s residues of different transmembrane regions for all derivatives apart from selected **B** derivatives, which due to the presence of the additional acetyl moiety makes the ligand too large for bulkier arylpiperazines to find optimal interactions in the binding site. Molecular docking studies suggest that upon anchoring to D116 coumarin derivatives can extend both toward transmembrane regions 4 and 7 to find favorable interactions within the binding site. The two-carbon linker makes such an extension impossible, lowering in most cases the potency of the antagonist.

Finally, for 8-acetyl-7-hydroxy-4-methylchromen-2-one (**C**) derivatives, all compounds with the five-carbon linker (**5a**–**5i**) showed higher antagonistic activities than their two-carbon linker counterparts (**6a**–**6i**), with the exception of 8-acetyl-7-(2-[4-(2-cyanophenyl)piperazin- 1-yl]ethoxy)-4-methylchromen-2-one (**6j**), which showed a higher activity (EC_50_ = 7804 ± 1876 nM) than its analogue, 8-acetyl-7-(5-[4-(2-cyanophenyl)piperazin-1-yl]penthoxy)-4-methylchromen-2-one (**5j**) (EC_50_ = 13,860 ± 2059 nM). We can speculate that the higher antagonistic activities are a result of a similar structural feature as in the **A** family, due to a different position of the acetyl moiety in the **C** family, which lowers the volume of these derivatives with respect to the **B** family.

There is also a group of compounds with a moderately high 5-HT_1A_ antagonistic activity, which consists of 5-(5-(4-(2-fluorophenyl)piperazin-1-yl)pentyloxy)-4,7-dimethyl-2H-chromen-2-one (**1b**), 6-acetyl-5-(5-(4-(3-fluorophenyl)piperazin-1-yl)pentyloxy)-4,7-dimethyl-2H-chromen-2-one (**3e**), 6-acetyl-5-(5-(4-(2-bromophenyl)piperazin-1-yl)pentyloxy)-4,7-dimethyl-2H-chromen-2-one (**3f**), 6-acetyl-5-(2-(4-(3-fluorophenyl)piperazin-1-yl)ethoxy)-4,7-dimethyl-2H-chromen-2-one (**4e**) and 6-acetyl-5-(2-(4-(2-cyanophenyl)piperazin-1-yl)ethoxy)-4,7-dimethyl-2H-chromen-2-one (**4j**) (EC_50_ = 980 ± 207 nM, EC_50_ = 702 ± 112 nM, EC_50_ = 689 ± 138 nM, EC_50_ = 527 ± 191 nM, and EC_50_ = 538 ± 105 nM, respectively). There is no one particular shared structural feature of this group of compounds as it is composed of both 5-(CH_2_) (**1b**, **3e,** and **3f**) and 2-(CH_2_) (**4e**, **4j**) linkers and various arylpiperazines (2 or 3-fluorophenyl, 2-bromophenyl, or 2-cyanophenyl).

Two compounds, namely 5-(2-(4-(3-fluorophenyl)piperazin-1-yl)ethoxy)-4,7-dimethyl -2H-chromen-2-one (2e) and 5-(4-(4-(3-bromophenyl)piperazin-1-yl)ethoxy)-4,7-dimethyl- 2H-chromen-2-one (2g) acted as weak partial agonists at the 5-HT1A receptor. (Table 4). Interestingly, both of these derivatives contain a two-carbon linker between the arylpiperazinyl and the coumarin core and the phenyl ring on the piperazine with a halogen atom in position C-3. In the case of compound 2e (Emax = 118 ± 1.48) it is a fluorine atom, while in the case of 2g (Emax = 118 ± 6.5) it is a bromine atom.

#### 2.3.2. 5-HT_2A_ Receptor Affinity

As, it was shown in our previous studies, coumarin derivatives containing the three-carbon or four-carbon linker between the coumarin and piperazine moieties showed moderate affinities for the 5-HT_2A_ receptor [19,20,21]. As shown in Table 5, arylpiperazinyl derivatives of coumarin containing the two-carbon or five-carbon carbon linkers displayed varied 5-HT_2A_ receptor binding, but none of them showed affinities comparable to the reference compound, katanserin (K_i_ = 3.6 ± 0.5 nM). The highest binding was found for compounds **5i**, **1j,** and **5g** (K_i_ = 51 ± 8.3 nM, 79 ± 18 nM and 81 ± 19 nM, respectively). Compounds **5b**, **5c**, and **5f** showed moderate binding ranging from K_i_ = 108 ± 24 nM for compound **5f**, K_i_ = 122 ± 43 nM for compound **5b**, to K_i_ = 144 ± 38 nM for compound **5c**. The remaining compounds showed weak 5-HT_2A_ receptor binding, ranging between K_i_ = 291 ± 57 nM for compound **5h** and K_i_ = 11,870 ± 3086 nM for compound **4d**.

Overall, for the 5-HT_2A_ receptor, the group of 8-acetyl-7-hydroxy-4-methylchromen-2-one (**C**) derivatives with the five-carbon linker (compounds **5a**–**5j**) turned out to be the most promising ones. In this group we found compounds with the highest (**5i**, **5g**) and moderate affinity (**5b**, **5c**, **5f**). All derivatives with the five-carbon linker in the remaining families (**A** nad **B**) showed rather weak affinities, except 5-(5-(4-(2-cyanophenyl)piperazin-1-yl)pentyloxy)-4,7-dimethyl-2H-chromen-2-one (**1j**), from the 5-hydroxy-4,7-dimethylchromen-2-one (**A**) group of compounds. All derivatives containing the two-carbon linker also showed rather weak affinities for the 5-HT_2A_ receptor. These results indicate that only compounds bearing the (2-cyanophenyl)piperazin-1-yl (**1j**), (2,2-dichloro)piperazin-1-yl (**5i**) or (3-bromophenyl)piperazin-1-yl (**5g**) moieties were able to bind to the 5-HT_2A_ with high affinities. The introduction of the 2-cyano group to the phenyl ring of piperazine increased the 5-HT_2A_ receptor affinity in the case of 5-hydroxy- 4,7-dimethylchromen-2-one (**A**). On the other hand, introduction of the same cyano moiety to the phenyl ring of piperazine in 8-acetyl-7-hydroxy-4-methylchromen-2-one (**C**), drastically decreased binding from K_i_ = 79 ± 18 for **1j** to K_i_ = 2551 ± 765 for **5j**. The introduction of the 2,3-dichloro or 3-bromo moiety to phenyl ring of piperazine increased the affinity when the coumarin moiety was 8-acetyl-7-hydroxy-4-methyl chromen-2-one (**C**). In the case of 5-hydroxy-4,7-dimethyl chromen-2-one (**A**) and 6-acetyl-5-hydroxy-4,7-dimethylchromen-2-one (**B**) derivatives, introduction of the 2,3-dichloro or 3-bromo moieties resulted in derivatives with weak 5-HT_2A_ receptor affinities as seen for **1i**, **2i**, **3i**, **4i, 6i** (K_i_ = 1452 ± 653, K_i_ = 4306 ± 1550, K_i_ = 486 ± 102, K_i_ = 1642 ± 344 and K_i_ = 755 ± 178, respectively)) and **2g**, **3g**, **4g**, **6g** (K_i_ = 3538 ± 1135, K_i_ = 664 ± 154, K_i_ = 3986 ± 745 and K_i_ = 1494 ± 466, respectively). Changing the C-3 position of the bromo substituent on the phenyl ring of piperazine to the C-2 position slightly decreased binding affinity from K_i_ = 81 ± 19 for 8-acetyl-7-(5-[4-(3-bromohenyl)piperazin-1-yl]penthoxy)- -4-methylchromen-2-one (**5g**) to K_i_ = 108 ± 24 for 8-acetyl-7-(5-[4-(2-bromophenyl)piperazin-1-yl]- penthoxy)-4-methyl chromen-2-one (**5f**). Converting the bromine atom at the C-3 position to a fluorine atom drastically decreased binding affinity from K_i_ = 81 ± 19 for **5g** to K_i_ = 2114 ± 420 for **5e**. Similarly, the replacement of the cyano group at the C-2 position with a fluoro or bromo moiety at the C-2 position resulted in a decrease in affinity for the 5-HT_2A_ receptor, from K_i_ = 79 ± 18 for **1j t**o K_i_ = 1263 ± 479 for **1b** and K_i_ = 1548 ± 323 for **1f**.

The moderate agreement between the experimental and theoretical K_i_ values for 5HT_2A_ receptor warrant a short comment. While the predicted K_i_ values for the newly synthesized set of coumarins derivatives are usually in the low nanomolar range, the experimental values are usually closer to micromolar values. The most likely explanation of these discrepancies is the combination of the imperfection of our computational model of the 5HT_2A_ receptor, particularly in the binding site part and the limited accuracy of the computational methods. The second problem is very well-known in the scientific community, as it has been shown that while Autodock and other similar programs can identify the correct binding poses, they often have problem is predicting correct bonding affinities [29]. As for the accuracy of homology models of GPCRs, they certainly can be improved by resorting to more sophisticated methods, such as e.g., using multiple templates or going beyond the homology modelling, and we are planning to make use of these new methods in the future [30,31,32]. Nevertheless the most 2D schematic representations of the predicted binding sites for the selects, most interesting coumarins derivatives are presented in the Supplementary Materials. Taking compound **1j** as the example we can suggest, that this compound is able to perfectly fit into the binding site of the 5HT_2A_ receptor, keeping the salt bridge to D155, while retaining the coumarin part in the hydrophobic region of the binding site and the piperazine part in the hydrophilic one. This is not true for this compound binding to the 5HT_1A_ receptor, as the salt bridge to D116 forced **1j** to move the coumarin part into a more hydrophilic region, lowering the affinity to the receptor. Additionally, **1j** in the binding site of the 5HT_2A_ receptor is stabilized by two hydrogen bonds and a π-π stacking interactions with F340.

## 3. Materials and Methods

All starting materials were purchased from Aldrich or Merck and used without further purification. Microwave oven Plazmatronika 1000 was used (http://www.plazmatronika.com.pl (accessed on 27 December 2020)). Melting points were determined with ElectroThermal 9001 Digital Melting Point apparatus and are uncorrected. High resolution mass spectra were recorded on Quattro LCT (TOF). ^1^H NMR, ^13^C NMR spectra in solution were recorded at 25 C with a Varian NMRS-300 spectrometer, and standard Varian software was employed. The calculated shielding constants were used as an aid in an assignment of resonances of ^13^C atoms. Chemical shifts d [ppm] were referenced to TMS. TLC was carried out using Kieselgel 60 F254 sheets and spots were visualized by UV e 254 and 365 nm.

### 3.1. Chemistry

Compounds **1**–**6** and **1a**–**1j**, **2a**–**2j**, **3a**–**3j**, **4a**–**4j**, **5a**–**5j**, **6a**–**6j** were prepared in accordance with the previously reported procedures [19,33]. Atom numbering, ^1^H NMR and ^13^C NMR spectra of all synthesized compounds are available in the ESI.

5-(5-bromopentyloxy)-4,7-dimethyl-2H-chromen-2-one (1). Yield 44%; white solid; m.p. 99–101 °C; Rf = 0.86; ^1^H NMR (400 MHz, CDCl_3_, δ, ppm): 6.74 (1H, s, H-8), 6.51 (1H, s, H-6), 6.05 (1H, s, H-3), 4.04 (2H, t, *J* = 8 Hz, H-1′), 3.46 (2H, t, *J* = 8 Hz, H-5′), 2.58 (3H, s, H-10), 2.39 (3H, s, H-9), 2.01–1.85 (4H, m, H-2′, H-4′), 1.73–1.60 (2H, m, H-3′); ^13^C NMR (75 MHz, CDCl_3_, δ, ppm): 161.2 (C-2), 157.4 (C-5), 155.5 (C-4), 154.2 (C-8a), 143.2 (C-7), 113.7 (C-4a), 110.4 (C-6), 108.4 (C-3), 108.0 (C-8), 68.8 (C-1′), 33.6 (C-5′), 33.2 (C-4′), 32.5 (C-2′), 28.4 (C-3′), 25.2 (C-10), 22.2 (C-9); TOF MS ES+: [M+Na]^+^ calcd for C_16_H_19_O_3_Na Br (361.0415) found 361.0401.

5-(5-(4-(2-methoxyphenyl)piperazin-1-yl)pentyloxy)-4,7-dimethyl-2H-chromen-2-one (1a). Yield 90%; white solid; m.p. 66–68 °C; Rf = 0.16; ^1^H NMR (400 MHz, CDCl_3_, δ, ppm): 7.03–6.85 (4H, m, H-3”, H-4”, H-5”, H-6”), 6.73 (1H, s, H-8), 6.51 (1H, s, H-6), 6.04 (1H, s, H-3), 4.03 (2H, t, *J* = 10 Hz, H-1′), 3.86 (3H, s, H-7”), 3.11 (4H, br. s., H-3p, H-5p), 2.67 (4H, br. s., H-2p, H-6p), 2.58 (3H, s, H-10), 2.46 (2H, t, *J* = 10 Hz, H-5′), 2.38 (3H, s, H-9), 1.92–1.88 (2H, m, H-2′), 1.64–1.55 (4H, m, H-3′, H-4′); ^13^C NMR (75 MHz, CDCl_3_, δ, ppm): 161.2 (C-1”), 157.4 (C-2), 155.5 (C-4), 154.3 (C-5), 152.4 (C-8a), 143.2 (C-7, C-2”), 123.4 (C-6”), 121.2 (H-4”), 118.5 (C-5”), 113.6 (C-4a), 111.4 (C-6), 110.3 (C-3), 108.4 (C-3”), 118.1 (C-8), 68.9 (C-1′), 58.5 (C-3p, C-5p), 55.6 (C-5′), 53.5 (C-7”), 50.2 (C-2p, C-6p), 29.2 (C-2′), 24.7 (C-4′), 24.4 (C-10, C-3′), 22.2 (C-9); TOF MS ES+: [M+H]^+^ calcd for C_27_H_35_O_4_N_2_ (451.2597) found 451.2583.

5-(5-(4-(2-fluorophenyl)piperazin-1-yl)pentyloxy)-4,7-dimethyl-2H-chromen-2-one (1b). Yield 91%; white solid; m.p. 106–108 °C; Rf = 0.20; ^1^H NMR (400 MHz, CDCl_3_, δ, ppm): 7.09–6.89 (4H, m, H-3”, H-4”, H-5”, H-6”), 6.75 (1H, s, H-8), 6.51 (1H, s, H-6), 6.04 (1H, s, H-3), 4.03 (2H, t, *J* = 8 Hz, H-1′), 3.12 (4H, t, *J* = 6 Hz, H-3p, H-5p), 2.64 (4H, t, *J* = 6 Hz, H-2p, H-6p), 2.58 (3H, s, H-10), 2.45 (2H, t, *J* = 10 Hz, H-5′), 2.38 (3H, s, H-9), 1.94–1.87 (2H, m, H-2′), 1.66–1.52 (4H, m, H-3′, H-4′); ^13^C NMR (75 MHz, CDCl_3_, δ, ppm): 161.1 (C-2), 157.5 (C-2”), 157.4 (C-4), 155.5 (C-5), 154.2 (C-8a), 143.2 (C-7), 140.1 (C-1”), 124.7 (C-5”), 122.8 (C-4”), 119.2 (C-3”), 116.4 (C-6”), 116.2 (C-4a), 113.6 (C-6), 110.3 (C-3), 108.0 (C-8), 68.9 (C-1′), 58.5 (C-3p, C-5p), 53.4 (C-5′), 50.4 (C-2p, C-6p), 29.2 (C-2′), 26.4 (C-4′), 24.7 (C-3′), 24.4 (C-10), 22.2 (C-9); TOF MS ES+: [M+H]^+^ calcd for C_26_H_32_O_3_N_2_F (439.2397) found 439.2403.

5-(5-(4-(3-methoxyphenyl)piperazin-1-yl)pentyloxy)-4,7-dimethyl-2H-chromen-2-one (1c). Yield 84%; white solid; m.p. 103–105 °C; Rf = 0.31; ^1^H NMR (400 MHz, CDCl_3_, δ, ppm): 7.17 (1H, t, *J* = 12 Hz, H-5”), 6.74 (1H, s, H-8), 6.51–6.43 (4H, m, H-6, H-4”, H-2”, H-6”), 6.04 (1H, s, H-3), 4.03 (2H, t, *J* = 8 Hz, H-1′), 3.79 (3H, s, H-7”), 3.21 (4H, t, *J* = 8 Hz, H-3p, H-5p), 2.62–2.58 (7H, m, H-10, H-2p, H-6p), 2.43 (2H, t, *J* = 8 Hz, H-5′), 2.38 (3H, s, H-9), 1.94–1.85 (2H, m, H-2′), 1.68–1.51 (4H, m, H-3′, H-4′); ^13^C NMR (75 MHz, CDCl_3_, δ, ppm): 161.2 (C-3”), 160.8 (C-2), 157.4 (C-4), 155.5 (C-5), 154.2 (C-8a, C-1”), 143.2 (C-7), 130.1 (C-5”), 113.6 (C-4a), 110.3 (C-6), 109.2 (C-3), 108.4 (C-4”), 108.0 (C-8), 105.0 (C-6”), 103.0 (C-2”), 68.9 (C-1′), 58.4 (C-3p, C-5p), 55.4 (C-5′), 53.1 (C-7”), 48.7 (C-2p, C-6p), 29.2 (C-2′), 24.8 (C-4′, C-3′), 24.4 (C-10), 22.2 (C-9); TOF MS ES+: [M+H]^+^ calcd for C_27_H_35_O_4_N_2_ (451.2597) found 451.2585.

5-(5-(4-(2,5-dimethylphenyl)piperazin-1-yl)pentyloxy)-4,7-dimethyl-2H-chromen-2-one (1d). Yield 82%; white solid; m.p. 132–134 °C; Rf = 0.29; ^1^H NMR (400 MHz, CDCl_3_, δ, ppm): 7.05 (1H, d, *J* = 12 Hz, H-3”), 6.83–6.78 (2H, m, H-6, H-4”), 6.74 (1H, s, H-6”), 6.52 (1H, s, H-8), 6.05 (1H, s, H-3), 4.04 (2H, t, *J* = 8 Hz, H-1′), 2.95 (4H, t, *J* = 6 Hz, H-3p, H-5p), 2.62–2.59 (7H, m, H-2p, H-6p, H-10), 2.46 (2H, t, *J* = 10 Hz, H-5′), 2.39 (3H, s, H-9), 2.30 (3H, s, H-7”), 2.25 (3H, s, H-8”), 1.93–1.88 (2H, m, H-2′), 1.60–1.55 (4H, m, H-3′, H-4′); ^13^C NMR (75 MHz, CDCl_3_, δ, ppm): 161.2 (C-2), 157.4 (C-4), 155.5 (C-5), 154.2 (C-8a, C-1”), 143.2 (C-7), 136.6 (C-5”), 131.1 (C-2”), 129.4 (H-3”), 124.7 (C-4′), 120.3 (C-4a), 113.6 (C-6), 110.3 (C-3), 108.4 (C-6”), 108.0 (C-8), 68.8 (C-1′, C-5′), 58.3 (C-3p, C-5p), 51.8 (C-2p, C-6p), 29.1 (C-2′), 24.8 (C-4′), 24.3 (C-3′), 22.2 (C-10), 21.3 (C-9), 17.6 (C-7”, C-8”); TOF MS ES+: [M+H]^+^ calcd for C_28_H_37_O_3_N_2_ (449.2804) found 449.2790.

5-(5-(4-(3-fluorophenyl)piperazin-1-yl)pentyloxy)-4,7-dimethyl-2H-chromen-2-one (1e). Yield 77%; white solid; m.p. 101–102 °C; Rf = 0.28; ^1^H NMR (400 MHz, CDCl_3_, δ, ppm): 7.22–7.14 (1H, m, H-5”), 6.73 (1H, s, H-2”), 6.68–6.51 (4H, m, H-6, H-8, H-4”, H-6”), 6.04 (1H, s, H-3), 4.03 (2H, t, *J* = 8 Hz, H-1′), 3.21 (4H, t, *J* = 6 Hz, H-3p, H-5p), 2.61–2.57 (7H, m, H-10, H-2p, H-6p), 2.43 (2H, t, *J* = 10 Hz, H-5′), 2.38 (3H, s, H-9), 1.92–1.87 (2H, m, H-2′), 1.60–1.54 (4H, m, H-3′, H-4′); ^13^C NMR (75 MHz, CDCl_3_, δ, ppm): 165.7 (C-3”), 161.2 (C-2), 157.5 (C-4), 155.5 (C-5), 154.3 (C-1”), 153.0 (C-8a), 143.2 (C-7), 130.4 (C-5”), 113.6 (C-4a), 111.2 (C-6), 110.3 (C-3), 108.4 (C-6”), 106.2 (C-4”),105.9 (C-8), 102.6 (C-2”), 69.0 (C-1′), 58.6 (C-3p, C-5p), 53.3 (C-5′), 48.8 (C-2p, C-6p), 29.3 (C-2′), 26.8 (C-4′), 24.8 (C-10), 24.5 (C-3′), 22.2 (C-9); TOF MS ES+: [M+H]^+^ calcd for C_26_H_32_O_3_N_2_F (439.2386) found 439.2391.

5-(5-(4-(2-bromophenyl)piperazin-1-yl)pentyloxy)-4,7-dimethyl-2H-chromen-2-one (1f). Yield 48%; white solid; m.p. 143–144 °C; Rf = 0.34; ^1^H NMR (400 MHz, CDCl_3_, δ, ppm): 7.56 (1H, dd, *J* = 12 Hz, H-3”), 7.30–7.24 (1H, m, H-5”), 7.05 (1H, dd, *J* = 12 Hz, H-4”), 6.91 (1H, t, *J* = 12 Hz, H-6”), 6.74 (1H, s, H-8), 6.52 (1H, s, H-6), 6.04 (1H, s, H-3), 4.04 (2H, t, *J* = 8 Hz, H-1′), 3.08 (4H, br. s., H-3p, H-5p), 2.66 (4H, br. s., H-2p, H-6p), 2.59 (3H, s, H-10), 2.47 (2H, t, *J* = 10 Hz, H-5′), 2.39 (3H, s, H-9), 1.93–1.88 (2H, m, H-2′), 1.60–1.55 (4H, m, H-3′, H-4′); ^13^C NMR (75 MHz, CDCl_3_, δ, ppm): 161.2 (C-2), 157.5 (C-4), 155.5 (C-5), 154.3 (C-8a, C-1”), 143.2 (C-7), 134.0 (C-3”), 128.5 (C-4”), 124.6 (H-5”), 121.2 (C-6”, C-2”), 120.0 (C-4a), 113.6 (C-6), 110.3 (C-3), 108.0 (C-8), 69.0 (C-1′), 58.6 (C-3p, C-5p), 53.6 (C-5′), 51.8 (C-2p, C-6p), 29.3 (C-2′), 24.8 (C-4′), 24.5 (C-3′, C-10), 22.2 (C-9); TOF MS ES+: [M+H]^+^ calcd for C_26_H_32_O_3_N_2_Br (499.1596) found 499.1594.

5-(5-(4-(3-bromophenyl)piperazin-1-yl)pentyloxy)-4,7-dimethyl-2H-chromen-2-one (1g). Yield 66%; white solid; m.p. 107–109 °C; Rf = 0.26; ^1^H NMR (400 MHz, CDCl_3_, δ, ppm): 7.13–7.07 (1H, m, H-4”), 7.03 (1H, t, *J* = 4 Hz, H-5”), 6.96–6.93 (1H, m, H-6”), 6.84–6.81 (1H, m, H-2”), 6.74 (1H, s, H-8), 6.51 (1H, s, H-6), 6.04 (1H, s, H-3), 4.04 (2H, t, *J* = 10 Hz, H-1′), 3.20 (4H, t, *J* = 6 Hz, H-3p, H-5p), 2.61–2.58 (7H, m, H-10, H-2p, H-6p), 2.43 (2H, t, *J* = 8 Hz, H-5′), 2.38 (3H, s, H-9), 1.92–1.87 (2H, m, H-2′), 1.59–1.54 (4H, m, H-3′, H-4′); ^13^C NMR (75 MHz, CDCl_3_, δ, ppm): 161.2 (C-2), 157.5 (C-4), 155.5 (C-5), 154.3 (C-1”), 152.6 (C-8a), 143.2 (C-7), 130.5 (C-5”), 123.4 (C-3”), 122.4 (C-4”), 118.8 (C-4a), 114.5 (C-6), 113.6 (C-2”), 110.3 (C-6”), 108.4 (C-3), 108.0 (C-8), 69.0 (C-1′), 58.6 (C-3p, C-5p), 53.3 (C-5′), 48.8 (C-2p, C-6p), 29.3 (C-2′), 26.7 (C-4′), 24.8 (C-10), 24.5 (C-3′), 22.2 (C-9); TOF MS ES+: [M+H]^+^ calcd for C_26_H_32_O_3_N_2_Br (499.1596) found 439.1612.

5-(5-(4-(3,5-dimethylphenyl)piperazin-1-yl)pentyloxy)-4,7-dimethyl-2H-chromen-2-one (1h). Yield 65%; white solid; m.p. 104–106 °C; Rf = 0.34; ^1^H NMR (400 MHz, CDCl_3_, δ, ppm): 6.74 (1H, s, H-8), 6.56 (2H, s, H-2”, H-6”), 6.52 (2H, s, H-4”, H-6), 6.04 (1H, s, H-3), 4.03 (2H, t, *J* = 8 Hz, H-1′), 3.19 (4H, t, *J* = 8 Hz, H-3p, H-5p), 2.62–2.58 (7H, m, H-2p, H-6p, H-10), 2.43 (2H, t, *J* = 10 Hz, H-5′), 2.38 (3H, s, H-9), 2.27 (6H, s, H-7”, H-8”), 1.92–1.87 (2H, m, H-2′), 1.63–1.54 (4H, m, H-3′, H-4′); ^13^C NMR (75 MHz, CDCl_3_, δ, ppm): 161.2 (C-2), 157.5 (C-4), 155.5 (C-5), 154.3 (C-8a), 151.6 (C-1”), 143.2 (C-7), 136.8 (C-3”, C-5”), 121.9 (C-4”), 114.2 (C-4a), 113.6 (C-2”, C-6”), 110.3 (C-6), 108.4 (C-3), 108.0 (C-8), 69.0 (C-1′), 58.7 (C-3p, C-5p), 53.6 (C-5′), 49.4 (C-2p, C-6p), 29.3 (C-2′), 26.8 (C-4′), 24.8 (C-3′), 24.5 (C-10), 22.2 (C-9), 21.9 (C-7”, C-8”); TOF MS ES+: [M+H]^+^ calcd for C_28_H_37_O_3_N_2_ (449.2804) found 449.2809.

5-(5-(4-(2,3-dichlorophenyl)piperazin-1-yl)pentyloxy)-4,7-dimethyl-2H-chromen-2-one (1i). Yield 62%; white solid; m.p. 162–163 °C; Rf = 0.38; ^1^H NMR (400 MHz, CDCl_3_, δ, ppm): 7.16–7.14 (2H, m, H-4”, H-5”), 6.97–6.94 (1H, m, H-6”), 6.74 (1H, s, H-8), 6.52 (1H, s, H-6), 6.05 (1H, s, H-3), 4.04 (2H, t, *J* = 8 Hz, H-1′), 3.09 (4H, t, br. s., H-3p, H-5p), 2.66 (4H, br. s., H-2p, H-6p), 2.59 (3H, s, H-10), 2.47 (2H, t, *J* = 8 Hz, H-5′), 2.39 (3H, s, H-9), 1.93–1.88 (2H, m, H-2′), 1.60–1.57 (4H, m, H-3′, H-4′); ^13^C NMR (75 MHz, CDCl_3_, δ, ppm): 161.2 (C-2), 157.5 (C-4), 155.5 (C-5), 154.3 (C-8a, C-1”), 143.2 (C-7), 134.3 (C-3”), 127.2 (C-5”), 124.9 (C-2”), 118.8 (C-4”), 113.7 (C-6”), 110.3 (C-4a), 108.4 (C-3, C-6), 108.0 (C-8), 69.0 (C-1′), 58.6 (C-3p, C-5p), 53.5 (C-5′), 51.3 (C-2p, C-6p), 29.3 (C-2′), 26.6 (C-4′), 24.8 (C-3′), 24.5 (C-10), 22.2 (C-9); TOF MS ES+: [M+H]^+^ calcd for C_26_H_31_O_3_N_2_Cl_2_ (489.1712) found 489.1695.

5-(5-(4-(2-cyanophenyl)piperazin-1-yl)pentyloxy)-4,7-dimethyl-2H-chromen-2-one (1j). Yield 84%; cream solid; m.p. 148–149 °C; Rf = 0.32; ^1^H NMR (400 MHz, CDCl_3_, δ, ppm): 7.56 (1H, d, *J* = 8 Hz, H-3”), 7.49 (1H, t, *J* = 10 Hz, H-5”), 7.02–7.00 (2H, m, H-4”, H-6”), 6.47 (1H, s, H-8), 6.52 (1H, s, H-6), 6.04 (1H, s, H-3), 4.02 (2H, t, *J* = 8 Hz, H-1′), 3.25 (4H, t, *J* = 6 Hz, H-3p, H-5p), 2.68 (4H, br. s., H-2p, H-6p), 2.58 (3H, s, H-10), 2.47 (2H, t, *J* = 10 Hz, H-5′), 2.39 (3H, s, H-9), 1.93–1.88 (2H, m, H-2′), 1.64–1.55 (4H, m, H-3′, H-4′); ^13^C NMR (75 MHz, CDCl_3_, δ, ppm): 161.2 (C-2), 157.5 (C-4), 155.8 (C-5), 155.5 (C-8a), 154.3 (C-1”), 143.2 (C-7), 134.6 (C-5”), 134.0 (C-3”), 122.0 (C-7”), 118.9 (C-4”), 118.6 (C-6”), 113.6 (C-4a), 110.3 (C-6), 108.4 (C-7), 108.0 (C-3), 106.3 (C-8, C-2”), 69.0 (C-1′), 58.5 (C-3p, C-5p), 53.4 (C-5′), 51.6 (C-2p, C-6p), 29.3 (C-3′), 26.6 (C-4′), 24.5 (C-10), 22.2 (C-9); TOF MS ES+: [M+H]^+^ calcd for C_27_H_32_O_3_N_3_ (446.2444) found 446.2455.

5-(2-bromoethoxy)-4,7-dimethyl-2H-chromen-2-one (2). Yield 25%; white solid; m.p. 121–123 °C; Rf = 0.86; ^1^H NMR (400 MHz, CDCl_3_, δ, ppm): 6.81 (1H, s, H-8), 6.50 (1H, s, H-6), 6.10 (1H, s, H-3), 4.40 (2H, t, *J* = 8 Hz, H-1′), 3.74 (2H, t, *J* = 8 Hz, H-2′), 2.66 (3H, s, H-10), 2.41 (3H, s, H-9); ^13^C NMR (75 MHz, CDCl_3_, δ, ppm): 161.0 (C-2), 156.4 (C-4), 155.6 (C-5), 154.2 (C-8a), 143.2 (C-7), 114.1 (C-4a), 111.2 (C-6, C-3), 107.9 (C-8), 68.8 (C-1′), 29.0 (C-2′), 24.9 (C-10), 22.2 (C-9); TOF MS ES+: [M+Na]^+^ calcd for C_13_H_13_O_3_Na Br (318.9946) found 318.9961.

5-(2-(4-(2-methoxyphenyl)piperazin-1-yl)ethoxy)-4,7-dimethyl-2H-chromen-2-one (2a). Yield 70%; cream solid; m.p. 146–148 °C; Rf = 0.72; ^1^H NMR (400 MHz, CDCl_3_, δ, ppm): 7.04–6.86 (4H, m, H-3”, H-4”, H-5”, H-6”), 6.76 (1H, s, H-8), 6.56 (1H, s, H-6), 6.05 (1H, s, H-3), 4.22 (2H, t, *J* = 6 Hz, H-1′), 3.87 (3H, s, H-7”), 3.13 (4H, br. s., H-3p, H-5p), 2.95 (2H, t, *J* = 10 Hz, H-2′), 2.80 (4H, br. s., H-2p, H-6p), 2.62 (3H, s, H-10), 2.39 (3H, s, H-9); ^13^C NMR (75 MHz, CDCl_3_, δ, ppm): 161.1 (C-1”), 157.2 (C-2), 155.5 (C-4), 154.4 (C-5), 152.5 (C-8a), 143.2 (C-2”), 141,6 (C-7), 123.3 (C-6”), 121.2 (H-5”), 118.4 (C-4”, C-3”), 113.7 (C-4a), 111.4 (C-6), 110.6 (C-3), 108.3 (C-8), 66.8 (C-1′), 57.2 (C-3p, C-5p), 55.6 (C-2′), 53.9 (C-7”), 50.8 (C-2p, C-6p), 24.8 (C-10), 22.2 (C-9); TOF MS ES+: [M+Na]^+^ calcd for C_24_H_28_O_4_N_2_Na (431.1947) found 431.1954.

5-(2-(4-(2-fluorophenyl)piperazin-1-yl)ethoxy)-4,7-dimethyl-2H-chromen-2-one (2b). Yield 98%; white solid; m.p. 121–123 °C; Rf = 0.82; ^1^H NMR (400 MHz, CDCl_3_, δ, ppm): 7.09–7.01 (2H, m, H-3”, H-5”), 6.99–6.90 (2H, m, H-4”, H-6”), 6.74 (1H, s, H-8), 6.55 (1H, s, H-6), 6.04 (1H, s, H-3), 4.19 (2H, t, *J* = 8 Hz, H-1′), 3.22–3.20 (4H, m, H-3p, H-5p), 2.93 (2H, t, *J* = 8 Hz, H-2′), 2.77 (4H, t, *J* = 8 Hz, H-2p, H-6p), 2.61 (3H, s, H-10), 2.39 (3H, s, H-9); ^13^C NMR (75 MHz, CDCl_3_, δ, ppm): 161.1 (C-2), 157.5 (C-2”), 157.2 (C-4), 155.4 (C-5), 154.2 (C-8a), 143.2 (C-7), 140.2 (C-1”), 124.7 (C-5”), 124.6 (C-4a), 122.8 (C-4”), 119.1 (C-3′), 116.4 (C-6”), 113.6 (C-6), 110.5 (C-3), 108.5 (C-8), 66.8 (C-1′), 57.2 (C-2′), 53.8 (C-3p, C-5p), 50.7 (C-2p, C-6p), 24.7 (C-10), 22.2 (C-9); TOF MS ES+: [M+Na]^+^ calcd for C_23_H_25_O_3_N_2_FNa (419.1747) found 419.1729.

5-(2-(4-(3-methoxyphenyl)piperazin-1-yl)ethoxy)-4,7-dimethyl-2H-chromen-2-one (2c). Yield 89%; cream solid; m.p. 124–125 °C; Rf = 0.70; ^1^H NMR (400 MHz, CDCl_3_, δ, ppm): 7.17 (1H, t, *J* = 16 Hz, H-5”), 6.75 (1H, s, H-8), 6.55–6.53 (2H, m, H-6, H-6”), 6.47–6.41 (2H, m, H-2”, H-4”), 6.04 (1H, s, H-3), 4.19 (2H, t, *J* = 8 Hz, H-1′), 3.97 (3H, s, H-7”), 3.53 (4H, t, *J* = 6 Hz, H-3p, H-5p), 2.91 (2H, t, *J* = 8 Hz, H-2′), 2.73 (4H, t, *J* = 6 Hz, H-2p, H-6p), 2.60 (3H, s, H-10), 2.39 (3H, s, H-9); ^13^C NMR (75 MHz, CDCl_3_, δ, ppm): 161.1 (C-3”), 160.8 (C-2), 157.1 (C-4), 155.5 (C-5), 154.3 (C-8a), 152.7 (C-1”), 143.2 (C-7), 129.9 (C-5”), 113.7 (C-4a), 110.6 (C-6), 109.1 (C-3), 108.6 (C-4”), 108.3 (C-8), 104.7 (C-6”), 102.8 (C-2”), 66.8 (C-1′), 57.2 (C-2′), 55.4 (C-3p, C-5p), 53.7 (C-7”), 49.3 (C-2p, C-6p), 24.8 (C-10), 22.2 (C-9); TOF MS ES+: [M+Na]^+^ calcd for C_24_H_28_O_4_N_2_Na (431.1947) found 431.1929.

5-(2-(4-(2,5-dimethylphenyl)piperazin-1-yl)ethoxy)-4,7-dimethyl-2H-chromen-2-one (2d). Yield 74%; cream solid; m.p. 119–120 °C; Rf = 0.90; ^1^H NMR (400 MHz, CDCl_3_, δ, ppm): 7.05 (1H, d, *J* = 12 Hz, H-3”), 6.83–6.79 (2H, m, H-6”, H-2”), 6.74 (1H, s, H-8), 6.56 (1H, s, H-6), 6.04 (1H, s, H-3), 4.20 (2H, t, *J* = 8 Hz, H-1′), 2.96–2.91 (6H, m, H-3p, H-5p, H-2′), 2.73 (4H, br. s, H-2p, H-6p), 2.62 (3H, s, H-9), 2.39 (3H, s, H-10), 2.30 (3H, s, H-7′), 2.26 (3H, s, H-8”); ^13^C NMR (75 MHz, CDCl_3_, δ, ppm): 161.1 (C-2), 157.2 (C-4), 155.5 (C-5), 154.4 (C-8a), 151.3 (C-1”), 143.2 (C-7), 136.3 (C-5”), 131.1 (C-2”), 129.4 (H-3”), 124.0 (C-4”), 119.9 (C-4a), 113.7 (C-6”), 110.5 (C-6), 108.6 (C-3), 108.3 (C-8), 66.8 (C-1′), 57.2 (C-2′), 54.3 (C-3p, C-5p), 51.9 (C-2p, C-6p), 24.7 (C-10), 22.2 (C-9), 21.4 (C-8”), 17.6 (C-7”); TOF MS ES+: [M+Na]^+^ calcd for C_25_H_30_O_3_N_2_Na (429.2154) found 429.2164.

5-(2-(4-(3-fluorophenyl)piperazin-1-yl)ethoxy)-4,7-dimethyl-2H-chromen-2-one (2e). Yield 90%; brown solid; m.p. 120–122 °C; Rf = 0.80; ^1^H NMR (400 MHz, CDCl_3_, δ, ppm): 7.19 (1H, q, H-5”), 6.74 (1H, s, H-2”), 6.69–6.60 (2H, m, H-8, H-6”), 6.55–6.49 (2H, m, H-6, H-4”), 6.03 (1H, s, H-3), 4.18 (2H, t, *J* = 6 Hz, H-1′), 3.22 (4H, t, *J* = 6 Hz, H-3p, H-5p), 2.91 (2H, t, *J* = 8 Hz, H-2′), 2.72 (4H, t, *J* = 6 Hz, H-2p, H-6p), 2.60 (3H, s, H-10), 2.39 (3H, s, H-10); ^13^C NMR (75 MHz, CDCl_3_, δ, ppm): 165.6 (C-3”), 162.4 (C-2), 161.1 (C-4), 155.5 (C-5), 154.3 (C-1”), 153.0 (C-8a), 143.2 (C-7), 130.4 (C-5”), 113.7 (C-4a), 111.3 (C-6), 110.5 (C-3), 108.5 (C-4”), 108.3 (C-6”),106.3 (C-8), 103.0 (C-2”), 66.7 (C-1′), 57.1 (C-2′), 53.5 (C-3p, C-5p), 48.8 (C-2p, C-6p), 24.7 (C-10), 22.1 (C-9); TOF MS ES+: [M+Na]^+^ calcd for C_23_H_25_O_3_N_2_FNa (419.1747) found 419.1761.

5-(2-(4-(2-bromophenyl)piperazin-1-yl)ethoxy)-4,7-dimethyl-2H-chromen-2-one (2f). Yield 75%; yellow solid; m.p. 129–130 °C; Rf = 0.93; ^1^H NMR (400 MHz, CDCl_3_, δ, ppm): 7.56 (1H, dd, *J_1_* = 4 Hz, *J_2_* = 12 Hz, H-3”), 7.30–7.25 (1H, m, H-5”), 7.10–7.04 (1H, m, H-4”), 6.95–6.89 (1H, m, H-6”), 6.76 (1H, s, H-8), 6.56 (1H, s, H-6), 6.06 (1H, s, H-3), 4.21 (2H, t, *J* = 8 Hz, H-1′), 3.10 (4H, br. s., H-3p, H-5p), 2.95 (2H, t, *J* = 6 Hz, H-2′), 2.79 (4H, br. s., H-2p, H-6p), 2.62 (3H, s, H-10), 2.40 (3H, s, H-9); ^13^C NMR (75 MHz, CDCl_3_, δ, ppm): 161.1 (C-2), 157.0 (C-4), 155.5 (C-5), 154.2 (C-1”), 150.4 (C-8a), 146.9 (C-7), 143.3 (C-3”), 134.1 (C-4”), 128.6 (H-5”), 124.9 (C-6”), 121.2 (C-2”), 120.1 (C-4a), 113.9 (C-6), 110.8 (C-3), 108.6 (C-8), 66.6 (C-1′), 57.1 (C-2′), 53.9 (C-3p, C-5p), 51.5 (C-2p, C-6p), 24.8 (C-10), 22.2 (C-9); TOF MS ES+: [M+Na]^+^ calcd for C_23_H_25_O_3_N_2_BrNa (479.0946) found 479.0930.

5-(4-(4-(3-bromophenyl)piperazin-1-yl)ethoxy)-4,7-dimethyl-2H-chromen-2-one (2g). Yield 69%; cream solid; m.p. 126–127 °C; Rf = 0.78; ^1^H NMR (400 MHz, CDCl_3_, δ, ppm): 7.13–7.07 (1H, m, H-4”), 7.07–7.02 (1H, m, H-5”), 6.97–9.94 (1H, m, H-6”), 6.84–6.81 (1H, m, H-2”), 6.74 (1H, s, H-8), 6.54 (1H, s, H-6), 6.03 (1H, s, H-3), 4.18 (2H, t, *J* = 8 Hz, H-1′), 3.21 (4H, t, *J* = 6 Hz, H-3p, H-5p), 2.90 (2H, t, *J* = 8 Hz, H-2′), 2.71 (4H, t, *J* = 6 Hz, H-2p, H-6p), 2.59 (3H, s, H-10), 2.39 (3H, s, H-9); ^13^C NMR (75 MHz, CDCl_3_, δ, ppm): 161.1 (C-2), 157.1 (C-4), 154.5 (C-5), 154.3 (C-1”), 152.5 (C-8a), 143.2 (C-7), 130.7 (C-5”), 123.4 (C-3”), 122.5 (C-4”), 119.9 (C-4a), 118.9 (C-6), 115.6 (C-2”), 114.6 (C-6”), 110.5 (C-3), 108.3 (C-8), 66.7 (C-1′), 57.1 (C-2′), 53.5 (C-3p, C-5p), 48.9 (C-2p, C-6p), 24.7 (C-10), 22.2 (C-9); TOF MS ES+: [M+Na]^+^ calcd for C_23_H_25_O_3_N_2_BrNa (479.0946) found 479.0956.

5-(2-(4-(3,5-dimethylphenyl)piperazin-1-yl)ethoxy)-4,7-dimethyl-2H-chromen-2-one (2h). Yield 54%; white solid; m.p. 149–150 °C; Rf = 0.90; ^1^H NMR (400 MHz, CDCl_3_, δ, ppm): 6.78 (2H, s, H-2”, H-6”), 6.47 (2H, s H-6, H-8), 6.07 (2H, s, H-4”, H-3), 4.40–4.33 (6H, m, H-1′, H-3p, H-5p), 3.73 (4H, t, *J* = 8 Hz, H-2p, H-6p), 3.51 (2H, t, *J* = 8 Hz, H-2′), 2.64 (6H, s, H-10, H-9), 2.39 (6H, s, H-7”, H-8”); ^13^C NMR (75 MHz, CDCl_3_, δ, ppm): 161.00 (C-2), 156.4 (C-4), 155.6 (C-5), 154.2 (C-8a), 154.0 (C-1”), 143.2 (C-7, C-3”, C-5”), 113.9 (C-4”), 111.1 (C-4a), 108.5 (C-2”), 108.4 (C-6”), 108.0 (C-6, C-3), 107.9 (C-8), 69.7 (C-1′), 68.8 (C-2′), 29.1 (C-3p, C-5p), 25.2 (C-2p, C-6p), 24.9 (C-10, C-9), 22.2 (C-7”, C-8”); TOF MS ES+: [M+Na]^+^ calcd for C_25_H_30_O_3_N_2_Na (429.2154) found 429.2165.

5-(2-(4-(2,3-dichlorophenyl)piperazin-1-yl)ethoxy)-4,7-dimethyl-2H-chromen-2-one (2i). Yield 72%; ceram solid; m.p. 142–145 °C; Rf = 0.70; ^1^H NMR (400 MHz, CDCl_3_, δ, ppm): 7.18–7.12 (2H, m, H-4”, H-3”), 6.97–6.94 (1H, m, H-2”), 6.76 (1H, s, H-8), 6.56 (1H, s, H-6), 6.05 (1H, s, H-3), 4.20 (2H, t, *J* = 8 Hz, H-1′), 3.09 (4H, t, br. s., H-3p, H-5p), 2.95 (2H, t, *J* = 8 Hz, H-2′), 2.78 (4H, br. s., H-2p, H-6p), 2.55 (3H, s, H-10), 2.39 (3H, s, H-9); ^13^C NMR (75 MHz, CDCl_3_, δ, ppm): 161.1 (C-2), 157.2 (C-4), 155.5 (C-5), 154.3 (C-8a), 151.2 (C-1”), 143.2 (C-7), 134.3 (C-3”), 127.7 (C-5”), 124.9 (C-2”), 118.8 (C-3”, C-6”), 113.8 (C-4a), 110.6 (C-6), 108.6 (C-3), 108.3 (C-8), 66.8 (C-1′), 57.2 (C-2′), 53.8 (C-3p, C-5p), 51.5 (C-2p, C-6p), 24.8 (C-10), 22.2 (C-9); TOF MS ES+: [M+Na]^+^ calcd for C_23_H_24_O_3_N_2_Cl_2_Na (469.1062) found 469.1068.

5-(2-(4-(2-cyanophenyl)piperazin-1-yl)ethoxy)-4,7-dimethyl-2H-chromen-2-one (2j). Yield 74%; cream solid; m.p. 127–129 °C; Rf = 0.32; ^1^H NMR (400 MHz, CDCl_3_, δ, ppm): 7.58–7.47 (2H, m, H-3”, H-5”), 7.05–7.01 (2H, m, H-4”, H-6”), 6.75 (1H, s, H-8), 6.56 (1H, s, H-6), 6.05 (1H, s, H-3), 4.20 (2H, t, *J* = 8 Hz, H-1′), 3.26 (4H, t, *J* = 6 Hz, H-3p, H-5p), 2.95 (2H, t, *J* = 8 Hz, H-2′), 2.81 (4H, t, *J* = 6 Hz, H-2p, H-6p), 2.62 (3H, s, H-10), 2.40 (3H, s, H-9); ^13^C NMR (75 MHz, CDCl_3_, δ, ppm): 161.1 (C-2), 157.2 (C-4), 155.7 (C-5), 155.5 (C-8a), 154.4 (C-1”), 143.2 (C-7), 134.5 (C-5”), 134.0 (C-3”), 122.1 (C-7”), 118.9 (C-4”), 118.6 (C-6”), 113.7 (C-4a), 110.5 (C-6), 108.6 (C-3), 108.3 (C-8a), 106.3 (C-2”), 66.7 (C-1′), 57.0 (C-2′), 53.6 (C-3p, C-5p), 51.7 (C-2p, C-6p), 24.7 (C-10), 22.2 (C-9); TOF MS ES+: [M+Na]^+^ calcd for C_24_H_25_O_3_N_3_Na (426.1794) found 426.1779.

6-acetyl-5-(5-bromopentyloxy)-4,7-dimethyl-2H-chromen-2-one (3). Yield 89%; white solid; m.p. 78–80 °C; Rf = 0.84; ^1^H NMR (400 MHz, CDCl_3_, δ, ppm): 6.97 (1H, s, H-8), 6.18 (1H, s, H-3), 3.82 (2H, t, *J* = 8.8 Hz, H-1′), 3.43 (2H, t, *J* = 8.8 Hz, H-5′), 2.59 (3H, s, H-12), 2.54 (3H, s, H-10), 2.29 (3H, s, H-9), 1.96–1.86 (2H, m, H-2′), 1.83–1.73 (2H, m, H-4′), 1.62–1.52 (2H, m, H-3′); ^13^C NMR (75 MHz, CDCl_3_, δ, ppm): 204.5 (C-11), 160.0 (C-2), 154.7 (C-5), 154.2 (C-4), 152.0 (C-8a), 139.2 (C-7), 133.5 (C-6), 116.0 (C-3), 115.2 (C-8), 112.5 (C-4a), 78.0 (C-1′), 33.3 (C-5′), 32.6 (C-12), 32.3 (C-4′), 29.0 (C-2′), 24.5 (C-3′), 22.5 (C-10), 19.3 (C-9); TOF MS ES+: [M+Na]^+^ calcd for C_18_H_21_O_4_BrNa (403.021) found 403.0506

6-acetyl-5-(5-(4-(2-methoxyphenyl)piperazin-1-yl)pentyloxy)-4,7-dimethyl-2H-chromen-2-one (3a). Yield 79%; oil; Rf = 0.18; ^1^H NMR (400 MHz, CDCl_3_, δ, ppm): 7.00–6.85 (5H, m, H-3”, H-4”, H-5”, H-6”, H-8), 6.18 (1H, s, H-3), 3.87 (3H, s, H-7”), 3.82 (2H, t, *J* = 6.6 Hz, H-1′), 3.11 (4H, br. s., H-3p, H-5p), 2.67 (4H, br. s., H-2p, H-6p), 2.60 (3H, s, H-12), 2.55 (3H, s, H-10), 2.44 (2H, t, *J* = 10 Hz, H-5′), 2.29 (3H, s, H-9), 1.84–1.75 (2H, m, H-2′), 1.64–1.54 (2H, m, H-4′), 1.49–1.41 (2H, m, H-3′); ^13^C NMR (75 MHz, CDCl_3_, δ, ppm): 204.7 (C-11), 161.2 (C-1”), 154.9 (C-2), 154.5 (C-4), 152.5 (C-5), 152.3 (C-8a), 141.4 (C-7), 139.4 (C-2”), 133.6 (C-6”), 123.2 (H-4”), 121.2 (C-5”), 118.4 (C-6), 116.1 (C-3), 115.3 (C-8), 112.7 (C-3”), 111.4 (C-4a), 78.6 (C-1′), 58.7 (C-3p, C-5p), 55.6 (C-5′), 53.7 (C-7”), 50.7 (C-2p, C-6p), 32.7 (C-12), 30.0 (C-2′), 26.7 (C-4′), 24.0 (C-10), 22.8 (C-3′), 19.5 (C-9); TOF MS ES+: [M+H]^+^ calcd for C_29_H_37_O_5_N_2_ (493.2702) found 493.2704.

6-acetyl-5-(5-(4-(2-fluorophenyl)piperazin-1-yl)pentyloxy)-4,7-dimethyl-2H-chromen-2-one (3b). Yield 76%; cream solid; m.p. 137–139 °C; Rf = 0.32; ^1^H NMR (400 MHz, CDCl_3_, δ, ppm): 7.08–6.90 (5H, m, H-3”, H-4”, H-5”, H-6”, H-8), 6.18 (1H, s, H-3), 3.82 (2H, t, *J* = 8 Hz, H-1′), 3.13 (4H, t, *J* = 6 Hz, H-3p, H-5p), 2.65 (4H, t, *J* = 6 Hz, H-2p, H-6p), 2.60 (3H, s, H-12), 2.55 (3H, s, H-10), 2.43–2.40 (2H, m, H-5′), 2.29 (3H, s, H-9), 1.82–1.77 (2H, m, H-2′), 1.59–1.56 (2H, m, H-4′), 1.46–1.44 (2H, m, H-3′); ^13^C NMR (75 MHz, CDCl_3_, δ, ppm): 204.6 (C-11), 160.2 (C-2), 157.5 (C-5), 154.8 (C-4), 154.5 (C-2”), 154.3 (C-8a), 152.3 (C-7), 140.3 (C-1”), 139.4 (C-5”), 133.6 (C-4”), 124.6 (C-3”), 122.6 (C-6”), 119.1 (C-6), 116.4 (C-3), 155.3 (C-4a), 115.3 (C-8), 78.5 (C-1′), 58.6 (C-3p, C-5p), 53.5 (C-5′), 50.6 (C-2p, C-6p), 32.7 (C-12), 29.9 (C-2′), 26.7 (C-4′), 24.0 (C-3′), 22.7 (C-10), 19.5 (C-9); TOF MS ES+: [M+H]^+^ calcd for C_28_H_34_O_4_N_2_F (481.2503) found 481.2517.

6-acetyl-5-(5-(4-(3-methoxyphenyl)piperazin-1-yl)pentyloxy)-4,7-dimethyl-2H-chromen-2-one (3c). Yield 83%; white solid; m.p. 86–88 °C; Rf = 0.28; ^1^H NMR (400 MHz, CDCl_3_, δ, ppm): 7.17 (1H, t, *J* = 12 Hz, H-5”), 6.97 (1H, s, H-8), 6.47 (1H, d, *J* = 12 Hz, H-6”), 6.46–6.40 (2H, m, H-2”, H-4”), 6.17 (1H, s, H-3), 3.84–3.79 (5H, m, H-7”, H-1′), 3.21 (4H, t, *J* = 5.3 Hz, H-3p, H-5p), 2.60 (7H, br. s., H-12, H-2p, H-6p), 2.55 (3H, s, H-10), 2.41 (2H, t, *J* = 10 Hz, H-5′), 2.29 (3H, s, H-9), 1.84–1.75 (2H, m, H-2′), 1.64–1.54 (2H, m, H-4′)1.49–1.39 (2H, m, H-3′); ^13^C NMR (75 MHz, CDCl_3_, δ, ppm): 204.6 (C-11), 160.8 (C-3”), 160.2 (C-2), 154.8 (C-4), 154.5 (C-5), 152.8 (C-8a), 152.2 (C-1”), 139.4 (C-7), 133.6 (C-5”), 129.9 (C-6), 116.0 (C-3), 115.2 (C-8), 112.7 (C-4”), 109.0 (C-4a), 104.6 (C-6”), 102.7 (C-2”), 78.5 (C-1′), 58.6 (C-3p, C-5p), 55.4 (C-7”), 53.4 (C-5′), 49.2 (C-2p, C-6p), 32.7 (C-12), 29.9 (C-2′), 26.8 (C-4′), 23.9 (C-10), 22.7 (C-3′), 19.5 (C-9); TOF MS ES+: [M+H]^+^ calcd for C_29_H_37_O_5_N_2_ (493.2702) found 493.2713.

6-acetyl-5-(5-(4-(2,5-dimethylphenyl)piperazin-1-yl)pentyloxy)-4,7-dimethyl-2H-chromen-2-one (3d). Yield 75%; cream solid; m.p. 97–99 °C; Rf = 0.20; ^1^H NMR (400 MHz, CDCl_3_, δ, ppm): 7.05 (1H, d, *J* = 8 Hz, H-3”), 6.97 (1H, s, H-8), 6.83–6.78 (2H, m, H-4”, H-6”), 6.18 (1H, s, H-3), 3.82 (2H, t, *J* = 10 Hz, H-1′), 2.94 (4H, t, *J* = 6 Hz, H-3p, H-5p), 2.60 (7H, br. s, H-2p, H-6p, H-12), 2.55 (3H, s, H-10), 2.43 (2H, t, *J* = 10 Hz, H-5′), 2.29 (6H, s, H-9, H-7”), 2.25 (3H, s, H-8”), 1.85–1.75 (2H, m, H-2′), 1.64–1.54 (2H, m, H-4′), 1.49–1.39 (2H, m, H-3′); ^13^C NMR (75 MHz, CDCl_3_, δ, ppm): 204.6 (C-11), 160.2 (C-2), 154.9 (C-4), 154.6 (C-5), 152.3 (C-8a), 151.5 (C-1”), 139.4 (C-7), 136.2 (C-5”), 133.6 (C-2”), 131.0 (H-3”), 129.4 (C-4”), 123.9 (C-6), 119.9 (C-6”), 116.0 (C-3), 115.3 (C-8), 112.7 (C-4a), 78.6 (C-1′), 58.7 (C-5′), 54.0 (C-3p, C-5p), 51.8 (C-2p, C-6p), 32.7 (C-12), 29.9 (C-2′), 26.8 (C-4′), 24.0 (C-9), 22.7 (C-3′), 21.4 (C-10), 19.5 (C-7”),17.6 (C-8”); TOF MS ES+: [M+H]^+^ calcd for C_30_H_39_O_4_N_2_ (491.2910) found 491.2898.

6-acetyl-5-(5-(4-(3-fluorophenyl)piperazin-1-yl)pentyloxy)-4,7-dimethyl-2H-chromen-2-one (3e). Yield 68%; white solid; m.p. 150–152 °C; Rf = 0.27; ^1^H NMR (400 MHz, CDCl_3_, δ, ppm): 7.22–7.14 (1H, m, H-5”), 6.97 (1H, s, H-8), 6.68–6.52 (3H, m, H-2”, H-4”, H-6”), 6.17 (1H, s, H-3), 3.82 (2H, t, *J* = 8 Hz, H-1′), 3.21 (4H, t, *J* = 6 Hz, H-3p, H-5p), 2.58 (7H, br. s., H-12, H-2p, H-6p), 2.55 (3H, s, H-10), 2.41 (2H, t, *J* = 10 Hz, H-5′), 2.29 (3H, s, H-9), 1.84–1.75 (2H, m, H-2′), 1.63–1.39 (4H, m, H-3′, H-4′); ^13^C NMR (75 MHz, CDCl_3_, δ, ppm): 204.6 (C-11), 165.3 (C-3”), 162.4 (C-2), 160.2 (C-5), 154.8 (C-4), 153.0 (C-1”), 152.2 (C-8a), 139.3 (C-7), 133.6 (C-5”), 116.1 (C-6), 112.7 (C-3), 111.3 (C-8), 106.3 (C-4a), 106.0 (C-6”), 103.1 (C-4”), 102.7 (C-2”), 78.5 (C-1′), 58.4 (C-3p, C-5p), 53.1 (C-5′), 48.6 (C-2p, C-6p), 32.7 (C-12), 29.9 (C-2′), 26.5 (C-4′), 23.9 (C-10), 22.7 (C-3′), 19.5 (C-9); TOF MS ES+: [M+H]^+^ calcd for C_28_H_34_O_4_N_2_F (481.2503) found 481.2492.

6-acetyl-5-(5-(4-(2-bromophenyl)piperazin-1-yl)pentyloxy)-4,7-dimethyl-2H-chromen-2-one (3f). Yield 51.8%; cream solid; m.p. 108–109 °C; Rf = 0.31; ^1^H NMR (400 MHz, CDCl_3_, δ, ppm): 7.55 (1H, d, *J* = 12 Hz, H-3”), 7.27 (1H, t, *J* = 12 Hz, H-5”), 7.06 (1H, d, *J* = 12 Hz, H-6”), 6.97 (1H, s, H-8), 6.91 (1H, t, *J* = 10 Hz, H-4”), 6.18 (1H, s, H-3), 3.82 (2H, t, *J* = 10 Hz, H-1′), 3.09 (4H, br. s., H-3p, H-5p), 2.66 (4H, br. s., H-2p, H-6p), 2.60 (3H, s, H-12), 2.55 (3H, s, H-10), 2.44 (2H, t, *J* = 10 Hz, H-5′), 2.29 (3H, s, H-9), 1.85–1.75 (2H, m, H-2′), 1.64–1.42 (4H, m, H-3′, H-4′); ^13^C NMR (75 MHz, CDCl_3_, δ, ppm): 204.6 (C-11), 160.1 (C-2), 154.8 (C-4), 154.5 (C-5), 152.2 (C-8a), 150.6 (C-1”), 139.4 (C-7), 133.9 (C-3”), 133.6 (C-4”), 128.5 (H-5”), 124.7 (C-6”), 121.2 (C-2”), 120.0 (C-6), 116.0 (C-3), 115.3 (C-8), 112.7 (C-4a), 78.5 (C-1′), 58.5 (C-3p, C-5p), 53.5 (C-5′), 51.5 (C-2p, C-6p), 32.7 (C-12), 29.9 (C-2′), 26.5 (C-4′), 23.9 (C-3′), 22.7 (C-10), 19.5 (C-9); TOF MS ES+: [M+H]^+^ calcd for C_28_H_34_O_4_N_2_Br (541.1702) found 541.1720.

6-acetyl-5-(5-(4-(3-bromophenyl)piperazin-1-yl)pentyloxy)-4,7-dimethyl-2H-chromen-2-one (3g). Yield 43%; cream solid; m.p. 100–102 °C; Rf = 0.40; ^1^H NMR (400 MHz, CDCl_3_, δ, ppm): 7.13–6.94 (4H, m, H-2”, H-4”, H-5”, H-8), 6.83 (1H, d, *J* = 6 Hz, H-6”), 6.18 (1H, s, H-3), 3.82 (2H, t, *J* = 10 Hz, H-1′), 3.21 (4H, t, *J* = 6 Hz, H-3p, H-5p), 2.60 (7H, br. s., H-12, H-2p, H-6p), 2.55 (3H, s, H-10), 2.41 (2H, t, *J* = 10 Hz, H-5′), 2.30 (3H, s, H-9), 1.85–1.75 (2H, m, H-2′), 1.64–1.42 (4H, m, H-3′, H-4′); ^13^C NMR (75 MHz, CDCl_3_, δ, ppm): 204.6 (C-11), 160.2 (C-2), 154.9 (C-4), 155.5 (C-5), 152.2 (C-1”, C-8a), 139.3 (C-7), 133.6 (C-5”), 130.5 (C-3”), 123.4 (C-4”), 122.6 (C-6), 118.9 (C-2”), 116.1 (C-6”),115.3 (C-3), 114.6 (C-8), 112.7 (C-4a), 78.4 (C-1′), 58.4 (C-3p, C-5p), 53.1 (C-5′), 48.6 (C-2p, C-6p), 32.7 (C-12), 29.9 (C-2′), 26.5 (C-4′), 23.9 (C-10), 22.7 (C-3′), 19.5 (C-9); TOF MS ES+: [M+H]^+^ calcd for C_28_H_34_O_4_N_2_Br (541.1702) found 541.1701.

6-acetyl-5-(5-(4-(3,5-dimethylphenyl)piperazin-1-yl)pentyloxy)-4,7-dimethyl-2H-chromen-2-one (3h). Yield 83%; cream solid; m.p. 100–102 °C; Rf = 0.33; ^1^H NMR (400 MHz, CDCl_3_, δ, ppm): 6.97 (1H, s, H-8), 6.56 (2H, s, H-2”, H-6”), 6.52 (1H, s, H-4”), 6.18 (1H, s, H-3), 3.81 (2H, t, *J* = 10 Hz, H-1′), 3.20 (4H, t, *J* = 10 Hz, H-3p, H-5p), 2.59 (7H, br. s, H-2p, H-6p, H-12), 2.54 (3H, s, H-10), 2.41 (2H, t, *J* = 10 Hz, H-5′), 2.29 (3H, s, H-9), 2.27 (6H, s, H-7”, H-8”), 1.84–1.75 (2H, m, H-2′), 1.64–1.54 (2H, m, H-4′), 1.48–1.41 (2H, m, H3′); ^13^C NMR (75 MHz, CDCl_3_, δ, ppm): 204.6 (C-11), 160.2 (C-2), 154.9 (C-5), 154.5 (C-4), 152.3 (C-8a), 151.6 (C-1”), 139.4 (C-7), 138.8 (C-5”), 133.6 (C-3”), 121.9 (C-4”), 116.1 (C-6”, C-2”), 115.3 (C-6), 114.3 (C-3, C-8), 112.7 (C-4a), 78.5 (C-1′), 58.6 (C-3p, C-5p), 53.5 (C-5′), 49.4 (C-2p, C-6p), 32.7 (C-12), 29.9 (C-2′), 26.8 (C-4′), 24.0 (C-10), 22.8 (C-9), 21.8 (C-3′), 19.5 (C-7”, C-8”); TOF MS ES+: [M+H]^+^ calcd for C_30_H_39_O_4_N_2_ (491.2910) found 491.2918.

6-acetyl-5-(5-(4-(2,3-dichlorophenyl)piperazin-1-yl)pentyloxy)-4,7-dimethyl-2H-chromen-2-one (3i). Yield 64%; white solid; m.p. 129–130 °C; Rf = 0.38; ^1^H NMR (400 MHz, CDCl_3_, δ, ppm): 7.16–7.11 (2H, m, H-4”, H-5”), 6.97–6.95 (2H, m, H-6”, H-8), 6.18 (1H, s, H-3), 3.82 (2H, t, *J* = 8 Hz, H-1′), 3.09 (4H, t, br. s., H-3p, H-5p), 2.65 (4H, br. s., H-2p, H-6p), 2.60 (3H, s, H-12), 2.55 (3H, s, H-10), 2.44 (2H, t, *J* = 10 Hz, H-5′), 2.29 (3H, s, H-9), 1.85–1.75 (2H, m, H-2′), 1.64–1.54 (2H, m, H-4′), 1.49–1.41 (2H, m, H-3′); ^13^C NMR (75 MHz, CDCl_3_, δ, ppm): 204.7 (C-11), 160.2 (C-2), 154.9 (C-5), 154.5 (C-4), 152.3 (C-8a), 139.4 (C-1”), 134.2 (C-7), 133.6 (C-3”), 127.7 (C-5”), 124.9 (C-2”), 118.8 (C-4”), 116.1 (C-6”, C-6), 115.3 (C-3, C-8), 112.7 (C-4a), 78.5 (C-1′), 58.6 (C-3p, C-5p), 53.5 (C-5′), 51.4 (C-2p, C-6p),32.7 (C-12), 29.9 (C-2′), 23.9 (C-4′), 22.8 (C-3′, C-10), 19.5 (C-9); TOF MS ES+: [M+H]^+^ calcd for C_28_H_33_O_4_N_2_Cl_2_ (531.1817) found 531.1835.

6-acetyl-5-(5-(4-(2-cyanophenyl)piperazin-1-yl)pentyloxy)-4,7-dimethyl-2H-chromen-2-one (3j). Yield 67%; brown solid; m.p. 116–118 °C; Rf = 0.38; ^1^H NMR (400 MHz, CDCl_3_, δ, ppm): 7.58–7.46 (2H, m, H-3”, H-5”), 7.03–6.97 (3H, m, H-8, H-4”, H-6”), 6.18 (1H, s, H-3), 3.82 (2H, t, *J* = 8 Hz, H-1′), 3.26 (4H, t, *J* = 6 Hz, H-3p, H-5p), 2.69 (4H, t, *J* = 6 Hz, H-2p, H-6p), 2.60 (3H, s, H-12), 2.55 (3H, s, H-10), 2.45 (2H, t, *J* = 10 Hz, H-5′), 2.29 (3H, s, H-9), 1.85–1.75 (2H, m, H-2′), 1.64–1.54 (2H, m, H-4′), 1.49–1.39 (2H, m, H-3′); ^13^C NMR (75 MHz, CDCl_3_, δ, ppm): 204.6 (C-11), 160.2 (C-2), 155.8 (C-4), 154.8 (C-5), 154.5 (C-8a), 152.3 (C-1”), 139.4 (C-7), 134.5 (C-5”), 134.0 (C-3”), 133.6 (C-7”), 121.9 (C-4”), 118.8 (C-6”), 118.6 (C-6), 116.1 (C-3), 115.3 (C-8), 112.7 (C-4a), 106.2 (C-2”), 78.5 (C-1′), 58.4 (C-3p, C-5p), 53.9 (C-5′), 51.6 (C-2p, C-6p), 32.7 (C-12), 29.9 (C-2′), 26.7 (C-4′), 23.9 (C-10), 22.8 (C-3′), 21.5 (C-9); TOF MS ES+: [M+H]^+^ calcd for C_29_H_34_O_4_N_3_ (488.2549) found 488.2537.

6-acetyl-5-(2-bromoethoxy)-4,7-dimethyl-2H-chromen-2-one (4). Yield 50%; white solid; m.p. 168–169 °C; Rf = 0.85; ^1^H NMR (400 MHz, CDCl_3_, δ, ppm): 7.26 (1H, s, H-8), 6.20 (1H, s, H-3), 4.15 (2H, t, *J* = 8 Hz, H-1′), 3.57 (2H, t, *J* = 8 Hz, H-2′), 2.62 (3H, s, H-12), 2.58 (3H, s, H-10), 2.30 (3H, s, H-9); ^13^C NMR (75 MHz, CDCl_3_, δ, ppm): 204.7 (C-11), 160.0 (C-2), 154.7 (C-5), 153.0 (C-3), 151.8 (C-8a), 139.4 (C-7), 133.7 (C-6), 116.5 (C-3), 115.9 (C-8), 112.7 (C-4a), 77.4 (C-1′), 33.1 (C-12), 28.9 (C-2′), 22.9 (C-10), 19.5 (C-9); TOF MS ES+: [M+Na]^+^ calcd for C_15_H_15_O_4_BrNa (361.0051) found 361.0038.

6-acetyl-5-(2-(4-(2-methoxyphenyl)piperazin-1-yl)ethoxy)-4,7-dimethyl-2H-chromen-2-one (4a). Yield 69%; cream solid; m.p. 132–134 °C; Rf = 0.56; ^1^H NMR (400 MHz, CDCl_3_, δ, ppm): 7.04–6.85 (5H, m, H-3”, H-4”, H-5”, H-6”, H-8), 6.18 (1H, s, H-3), 3.96 (2H, t, *J* = 8 Hz, H-1′), 3.88 (3H, s, H-7”), 3.10 (4H, br. s., H-3p, H-5p), 2.76 (2H, t, *J* = 8 Hz, H-2′), 2.70 (4H, br. s., H-2p, H-6p), 2.66 (3H, s, H-12), 2.59 (3H, s, H-10), 2.30 (3H, s, H-9); ^13^C NMR (75 MHz, CDCl_3_, δ, ppm): 204.9 (C-11), 160.1 (C-1”), 154.9 (C-2, C-5), 152.4 (C-4, C-8a), 139.4 (C-7), 133.6 (C-1”), 123.4 (H-6”), 121.2 (C-4”), 118.5 (C-5”), 116.2 (C-6), 115.6 (C-3”), 112.8 (C-3), 111.5 (C-8a, C-4a), 77.6 (C-1′), 57.7 (C-2′, C-7”), 55.6 (C-3p, C-5p), 54.0 (C-2p, C-6p), 32.9 (C-12), 22.9 (C-10), 19.6 (C-9); TOF MS ES+: [M+Na]^+^ calcd for C_26_H_30_O_5_N_2_Na (473.2052) found 473.2059.

6-acetyl-5-(2-(4-(2-fluorophenyl)piperazin-1-yl)ethoxy)-4,7-dimethyl-2H-chromen-2-one (4b). Yield 68%; cream solid; m.p. 118–119 °C; Rf = 0.84; ^1^H NMR (400 MHz, CDCl_3_, δ, ppm): 7.10–6.91 (5H, m, H-3”, H-4”, H-5”, H-6”, H-8), 6.19 (1H, s, H-3), 3.97 (2H, br. s, H-1′), 3.13 (4H, br. s, H-3p, H-5p), 2.78 (6H, br. s, H-2′, H-2p, H-6p), 2.71–2.65 (3H, br. s, H-12), 2.61 (3H, s, H-10), 2.30 (3H, s, H-9); ^13^C NMR (75 MHz, CDCl_3_, δ, ppm): 204.9 (C-11), 160.8 (C-2), 160.1 (C-5), 154.8 (C-2”), 154.2 (C-4), 152.6 (C-8a), 152.3 (C-7), 139.4 (C-1”), 133.6 (C-5”), 130.0 (C-4”), 116.1 (C-3”), 115.5 (C-6”), 112.7 (C-6), 109.0 (C-3), 104.8 (C-4a), 102.7 (C-8), 75.0 (C-1′), 57.6 (C-2′), 55.4 (C-3p, C-5p), 53.8 (C-2p, C-6p), 32.9 (C-12), 22.8 (C-10), 19.6 (C-9); TOF MS ES+: [M+Na]^+^ calcd for C_25_H_27_O_4_N_2_FNa (461.1853) found 461.1872.

6-acetyl-5-(2-(4-(3-methoxyphenyl)piperazin-1-yl)ethoxy)-4,7-dimethyl-2H-chromen-2-one (4c). Yield 73%; cream solid; m.p. 75–76 °C; Rf = 0.70; ^1^H NMR (400 MHz, CDCl_3_, δ, ppm): 7.18 (1H, t, *J* = 10 Hz, H-5”), 6.98 (1H, s, H-8), 6.55 (1H, d, *J* = 12 Hz, H-6”), 6.48–6.41 (2H, m, H-2”, H-4”), 6.18 (1H, s, H-3), 3.96 (2H, t, *J* = 6 Hz, H-1′), 3.80 (3H, s, H-7”), 3.21 (4H, t, *J* = 8 Hz, H-3p, H-5p), 2.75 (2H, t, *J* = 6 Hz, H-2′), 2.65–2.64 (7H, m., H-12, H-2p, H-6p), 2.60 (3H, s, H-10), 2.30 (3H, s, H-9); ^13^C NMR (75 MHz, CDCl_3_, δ, ppm): 204.9 (C-11), 160.1 (C-3”), 157.5 (C-2), 154.9 (C-5), 154.3 (C-4), 152.4 (C-8a), 152.3 (C-1”), 139.4 (C-7), 133.6 (C-5”), 124.7 (C-6), 119.2 (C-3), 116.5 (C-8), 116.2 (C-4”), 115.6 (C-4a), 115.5 (C-6”), 112.8 (C-2”), 76.8 (C-1′), 57.7 (C-2′), 53.9 (C-3p, C-5p), 50.5 (C-7”), 50.4 (C-2p, C-6p), 32.9 (C-12), 22.9 (C-10), 19.6 (C-9); TOF MS ES+: [M+Na]^+^ calcd for C_26_H_30_O_5_N_2_Na (473.2052) found 473.2067.

6-acetyl-5-(2-(4-(2,5-dimethylphenyl)piperazin-1-yl)ethoxy)-4,7-dimethyl-2H-chromen-2-one (4d). Yield 75%; cream solid; m.p. 97–99 °C; Rf = 0.20; ^1^H NMR (400 MHz, CDCl_3_, δ, ppm): 7.05 (1H, d, *J* = 8 Hz, H-3”), 6.97 (1H, s, H-8), 6.83–6.78 (2H, m, H-4”, H-6”), 6.18 (1H, s, H-3), 3.82 (2H, t, *J* = 10 Hz, H-1′), 2.94 (4H, t, *J* = 6 Hz, H-3p, H-5p), 2.60 (7H, br. s, H-2p, H-6p, H-12), 2.55 (3H, s, H-10), 2.43 (2H, t, *J* = 10 Hz, H-5′), 2.29 (6H, s, H-9, H-7”), 2.25 (3H, s, H-8”), 1.85–1.75 (2H, m, H-2′), 1.64–1.54 (2H, m, H-4′), 1.49–1.39 (2H, m, H-3′); ^13^C NMR (75 MHz, CDCl_3_, δ, ppm): 204.6 (C-11), 160.2 (C-2), 154.9 (C-4), 154.6 (C-5), 152.3 (C-8a), 151.5 (C-1”), 139.4 (C-7), 136.2 (C-5”), 133.6 (C-2”), 131.0 (H-3”), 129.4 (C-4”), 123.9 (C-6), 119.9 (C-6”), 116.0 (C-3), 115.3 (C-8), 112.7 (C-4a), 78.6 (C-1′), 58.7 (C-5′), 54.0 (C-3p, C-5p), 51.8 (C-2p, C-6p), 32.7 (C-12), 29.9 (C-2′), 26.8 (C-4′, 24.0 (C-9), 22.7 (C-3′), 21.4 (C-10), 19.5 (C-7”),17.6 (C-8”); TOF MS ES+: [M+H]^+^ calcd for C_30_H_39_O_4_N_2_ (491.2910) found 491.2898.

6-acetyl-5-(2-(4-(3-fluorophenyl)piperazin-1-yl)ethoxy)-4,7-dimethyl-2H-chromen-2-one (4e). Yield 93%; cream solid; m.p. 135–136 °C; Rf = 0.70; ^1^H NMR (400 MHz, CDCl_3_, δ, ppm): 7.23–7.15 (1H, m, H-5”), 6.98 (1H, s, H-8), 6.69–6.65 (1H, m, H-2”), 6.62–6.50 (2H, m, H-4”, H-6”), 6.18 (1H, s, H-3), 3.96 (2H, t, *J* = 8 Hz, H-1′), 3.22 (4H, t, *J* = 8 Hz, H-3p, H-5p), 2.75 (2H, t, *J* = 8 Hz, H-2′), 2.67 (7H, br. s., H-12, H-2p, H-6p), 2.60 (3H, s, H-10), 2.30 (3H, s, H-9); ^13^C NMR (75 MHz, CDCl_3_, δ, ppm): 204.9 (C-11), 165.6 (C-3”), 162.4 (C-2), 160.1 (C-5), 154.8 (C-4), 154.2 (C-1”), 152.9 (C-8a), 139.5 (C-7), 133.6 (C-5”), 116.2 (C-6), 114.5 (C-4”), 112.7 (C-3), 111.3 (C-8), 111.2 (C-6”), 106.3 (C-4a), 103.0 (C-2”), 75.2 (C-1′), 57.7 (C-2′), 53.7 (C-3p, C-5p), 48.7 (C-2p, C-6p), 32.9 (C-12), 22.8 (C-10), 19.6 (C-9); TOF MS ES+: [M+Na]^+^ calcd for C_25_H_27_O_4_N_2_FNa (461.1853) found 461.1845.

6-acetyl-5-(2-(4-(2-bromophenyl)piperazin-1-yl)ethoxy)-4,7-dimethyl-2H-chromen-2-one (4f). Yield 70%; ceram solid; m.p. 83–85 °C; Rf = 0.38; ^1^H NMR (400 MHz, CDCl_3_, δ, ppm): 7.50 (1H, dd, *J*_1_ = 8 Hz, *J*_2_ = 4 Hz H-3”), 7.23–7.17 (1H, m, H-5”), 6.98 (1H, dd, *J*_1_ = 8 Hz, *J*_2_ = 4 Hz H-4”), 6.91 (1H, s, H-8), 6.87–6.81 (1H, m, H-6”), 6.11 (1H, s, H-3), 3.90 (2H, t, *J* = 8 Hz, H-1′), 2.99 (4H, br. s., H-3p, H-5p), 2.71 (2H, t, *J* = 8 Hz, H-2′), 2.63 (4H, br. s., H-2p, H-6p), 2.59 (3H, s, H-12), 2.54 (3H, s, H-10), 2.23 (3H, s, H-9); ^13^C NMR (75 MHz, CDCl_3_, δ, ppm): 204.8 (C-11), 160.2 (C-2), 154.8 (C-4), 154.4 (C-5), 152.4 (C-8a), 150.6 (C-1”), 139.5 (C-7), 134.0 (C-3”), 133.5 (C-4”), 128.5 (H-5”), 124.6 (C-6”), 121.0 (C-2”), 120.0 (C-6), 116.1 (C-3), 115.4 (C-8), 112.8 (C-4a), 75.3 (C-1′), 57.8 (C-2′), 54.0 (C-3p, C-5p), 51.7 (C-2p, C-6p), 32.9 (C-12), 22.9 (C-10), 19.6 (C-9); TOF MS ES+: [M+Na]^+^ calcd for C_25_H_27_O_4_N_2_BrNa (521.1052) found 521.1048.

6-acetyl-5-(2-(4-(2-bromophenyl)piperazin-1-yl)ethgoxy)-4,7-dimethyl-2H-chromen-2-one (4g). Yield 62.4%; ceram solid; m.p. 127–128 °C; Rf = 0.31; ^1^H NMR (400 MHz, CDCl_3_, δ, ppm): 7.10 (1H, t, *J* = 10 Hz, H-4”), 7.03 (1H, t, *J* = 4 Hz, H-5”), 6.98–6.94 (2H, m, H-6”, H-8), 6.84–6.81 (1H, m, H-2”), 6.17 (1H, s, H-3), 3.95 (2H, t, *J* = 8 Hz, H-1′), 3.20 (4H, t, *J* = 6 Hz, H-3p, H-5p), 2.74 (2H, t, *J* = 8 Hz, H-2′), 2.65–2.62 (7H, m, H-2p, H-6p, H-12), 2.60 (3H, s, H-10), 2.30 (3H, s, H-9); ^13^C NMR (75 MHz, CDCl_3_, δ, ppm): 204.9 (C-11), 160.1 (C-2), 154.8 (C-4), 154.1 (C-5), 152.4 (C-8a), 152.2 (C-1”), 139.4 (C-7), 133.6 (C-3”), 130.5 (C-5”), 123.4 (H-4”), 122.6 (C-6”), 118.9 (C-2”), 116.2 (C-6), 116.5 (C-3), 114.6 (C-8), 112.7 (C-4a), 74.9 (C-1′), 57.6 (C-2′), 53.6 (C-3p, C-5p), 48.6 (C-2p, C-6p), 32.9 (C-12), 22.9 (C-10), 19.5 (C-9); TOF MS ES+: [M+Na]^+^ calcd for C_25_H_27_O_4_N_2_BrNa (521.1052) found 521.1066.

6-acetyl-5-(2-(4-(3,5-dimethylphenyl)piperazin-1-yl)ethoxy)-4,7-dimethyl-2H-chromen-2-one (4h). Yield 59%; oil; Rf = 0.42; ^1^H NMR (400 MHz, CDCl_3_, δ, ppm): 6.91 (1H, s, H-8), 6.47 (2H, s, H-2”, H-6”), 6.46 (1H, s, H-4”), 6.11 (1H, s, H-3), 3.88 (2H, t, *J* = 6 Hz, H-1′), 3.12 (4H, t, *J* = 6 Hz, H-3p, H-5p), 2.67 (2H, t, *J* = 8 Hz, H-2′), 2.58 (7H, s, H-2p, H-6p, H-12), 2.52 (3H, s, H-10), 2.21 (6H, s, H-7”, H-8”), 2.22 (3H, s, H-9); ^13^C NMR (75 MHz, CDCl_3_, δ, ppm): 204.9 (C-11), 160.1 (C-2), 154.9 (C-5), 154.3 (C-4), 152.4 (C-8a), 151.4 (C-1”), 139.5 (C-7), 138.9 (C-5”), 133.6 (C-3”), 122.0 (C-4”), 116.1 (C-2”), 115.5 (C-6, C-6”), 114.9 (C-3), 114.2 (C-8), 112.8 (C-4a), 75.1 (C-1′), 57.6 (C-2′), 53.9 (C-3p, C-5p), 49.3 (C-2p, C-6p), 32.9 (C-12), 22.9 (C-10), 21.8 (C-9), 19.6 (C-7”, C-8”); TOF MS ES+: [M+Na]^+^ calcd for C_27_H_32_O_4_N_2_Na (471.2260) found 471.2251.

6-acetyl-5-(2-(4-(2,3-dichlorophenyl)piperazin-1-yl)ethoxy)-4,7-dimethyl-2H-chromen-2-one (4i). Yield 72%; yellow solid; m.p. 162–163 °C; Rf = 0.46; ^1^H NMR (400 MHz, CDCl_3_, δ, ppm): 7.19–7.15 (2H, m, H-4”, H-5”), 6.99–6.92 (3H, m, H-6”, H-6, H-8), 6.19 (1H, s, H-3), 3.96 (2H, t, *J* = 8 Hz, H-1′), 3.06 (4H, t, br. s., H-3p, H-5p), 2.78 (2H, t, *J* = 8 Hz, H-2′), 2.70–2.66 (10H, m, H-2p, H-6p, H-12, H-10), 2.30 (3H, s, H-9); ^13^C NMR (75 MHz, CDCl_3_, δ, ppm): 204.8 (C-11), 160.1 (C-2), 154.8 (C-5), 154.2 (C-4), 152.3 (C-8a), 151.1 (C-1”), 139.4 (C-7), 134.3 (C-3”), 133.5 (C-5”), 127.7 (C-2”), 124.9 (C-4”), 118.8 (C-6”), 116.2 (C-6), 115.5 (C-3, C-8), 112.7 (C-4a), 76.8 (C-1′), 57.6 (C-3p, C-5p), 53.9 (C-2p, C-6p),51.1 (C-2′), 32.9 (C-12), 22.9 (C-10), 19.6 (C-9); TOF MS ES+: [M+Na]^+^ calcd for C_25_H_26_O_4_N_2_Cl_2_Na (511.1167) found 511.1147.

6-acetyl-5-(2-(4-(2-cyanophenyl)piperazin-1-yl)ethoxy)-4,7-dimethyl-2H-chromen-2-one (4j). Yield 70.5%; white solid; m.p. 153–155 °C; Rf = 0.37; ^1^H NMR (400 MHz, CDCl_3_, δ, ppm): 7.59–7.47 (2H, m, H-3”, H-5”), 7.05–6.99 (3H, m, H-8, H-4”, H-6”), 6.19 (1H, s, H-3), 3.98 (2H, t, *J* = 6 Hz, H-1′), 3.25 (4H, br. s, H-3p, H-5p), 2.80–2.75 (6H, m, H-2p, H-6p, H-2), 2.66 (3H, s, H-12), 2.61 (3H, s, H-10), 2.31 (3H, s, H-9); ^13^C NMR (75 MHz, CDCl_3_, δ, ppm): 204.9 (C-11), 160.1 (C-2), 154.9 (C-4, C-5), 152.2 (C-8a, C-1”), 139.4 (C-7), 134.5 (C-5”), 134.1 (C-3”), 133.6 (C-7”), 118.9 (C-4”), 118.5 (C-6”), 116.2 (C-6, C-3), 115.6 (C-8), 112.7 (C-4a), 102.5 (C-2”), 76.8 (C-1′), 57.5 (C-2′), 53.7 (C-3p, C-5p), 51.4 (C-2p, C-6p), 32.9 (C-12), 22.9 (C-10), 19.6 (C-9); TOF MS ES+: [M+Na]^+^ calcd for C_26_H_27_O_4_N_3_Na (468.1899) found 468.1913

8-acetyl-7-(5-bromopenthoxy)-4-methylchromen-2-one (5). Yield 89%; white solid; m.p.: 101–103 °C; Rf = 0.84; ^1^H NMR (400 MHz, CHCl_3_) δ ppm: 7.55 (1H, d, *J* = 9 Hz, H-5), 6.86 (1H, d, *J* = 9.0 Hz, H-6), 6.14 (1H, s, H-3), 4.09 (2H, t, *J* = 6.2 Hz, H-1′), 3.43 (2H, t, *J* = 6.6 Hz, H-5′), 2.59 (3H, s, H-11), 2.39 (3H, s, H-9), 1.89 (4H, m, H-2′, H-4′), 1.63 (2H, m, H-3′); ^13^C NMR (75 MHz, CHCl_3_) δ ppm: 199.4 (C-10), 160.1 (C-2), 158.0 (C-7), 152.2 (C-8a), 150.9 (C-4), 126.5 (C-5), 119.9 (C-8), 114.2 (C-6), 112.8 (C-3), 108.5 (C-4a), 69.0 (C-1′), 33.6 (C-5′), 32.6 (C-11), 32.4 (C-4′), 28.3 (C-2′), 24.8 (C-3′), 18.9 (C-9); TOF MS ES+: [M + Na]^+^ calcd for C_17_H_19_O_4_BrNa: 389.0364 found 389.0375.

8-acetyl-7-(5-[4-(2-methoxyphenyl)piperazin-1-yl]penthoxy)-4-methylchromen-2-one (5a). Yield 83%; brown solid; m.p.: 126–128 °C; Rf = 0.22; ^1^H NMR (300 MHz, CDCl_3_) δ ppm: 7.56 (1H, d, *J* = 8.7 Hz, H-5), 6.94 (5H, m, H-6, H-3”, H-4”, H-5”, H-6”), 6.14 (1H, s, H-3), 4.09 (2H, t, *J* = 8.6 Hz, H-1′), 3.86 (3H, s, H-7”), 3.14 (4H, br. s, H-3p, H-5p), 2.71 (4H, br. s, H-2p, H-6p), 2.59 (3H, s, H-11), 2.48 (2H, t, *J* = 7.5 Hz, H-5′), 2.39 (3H, s, H-9), 1.85 (2H, m, H-2′), 1.63 (2H, m, H-4′), 1.51 (2H, m, H-3′); ^13^C NMR (75 MHz, CDCl_3_) δ ppm: 199.5 (C-10), 160.1 (C-1”), 158.1 (C-2), 152.4 (C-7), 152.3 (C-8a), 150.8 (C-4), 141.0 (C-2”), 126.5 (C-5), 123.3 (C-6”), 121.2 (C-5′), 119.8 (C-4”), 118.5 (C-8), 114.1 (C-6), 112.7 (C-3”), 111.4 (C-4), 108.5 (C-4a), 69.1 (C-1′), 58.5 (C-3p, C-5p), 55.5 (C-5′), 53.5 (C-2p), 50.2 (C-6p), 32.6 (C-11), 28.9 (C-2′, C-7”), 26.0 (C-4′), 23.9 (C-3′), 18.9 (C-9); TOF MS ES + [M + Na]^+^ calcd for C_28_H_34_O_5_N_2_Na 501.2365, found 501.2345.

8-acetyl-7-(5-[4-(2-fluorophenyl)piperazin-1-yl]penthoxy)-4-methylchromen-2-one (5b). Yield 79.6%; white solid; m.p.: 95–97 °C; Rf = 0.20; ^1^H NMR (300 MHz, CDCl_3_) δ ppm: 7.54 (1H, d, *J* = 8.7 Hz, H-5), 6.94 (5H, m, H-6, H-3”, H-4”, H-5”, H-6”), 6.13 (1H, s, H-3), 4.08 (2H, t, *J* = 6.3 Hz, H-1′), 3.14 (4H, br. s, H-3p, H-5p), 2.66 (4H, br. s, H-2p, H-6p), 2.58 (3H, s, H-11), 2.54 (2H, t, *J* = 7.5 Hz, H-5′), 2.39 (3H, s, H-9), 1.84 (2H, m, H-2′), 1.64 (2H, m, H-4′), 1.49 (2H, m, H-3′); ^13^C NMR (75 MHz, CDCl_3_) δ ppm: 199.5 (C-10), 160.1 (C-2), 158.1 (C-7), 154.2 (C-2”), 152.2 (C-8a), 150.8 (C-4), 140.0 (C-1”), 126.5 (C-5), 124.7 (C-5”), 124.6 (C-4”), 122.8 (C-3”), 119.8 (C-6”), 119.2 (C-8), 116.1 (C-6), 112.7 (C-3), 108.5 (C-4a), 69.1 (C-1′), 58.4 (C-5′), 53.3 (C-3p, C-5p), 50.2 (C-2p, C-6p), 32.6 (C-11), 28.9 (C-2′), 26.2 (C-4′), 24.0 (C-3′), 18.9 (C-9); TOF MS ES+: [M + Na]^+^ calcd for C_27_H_31_O_4_N_2_FNa 489.2166, found 489.2182.

8-acetyl-7-(5-[4-(3-methoxyphenyl)piperazin-1-yl]penthoxy)-4-methylchromen-2-one (5c). Yield 69%; white solid; m.p.: 76–78 °C; Rf = 0.24; ^1^H NMR (300 MHz, CDCl_3_) δ ppm: 7.54 (1H, d, *J* = 8.7 Hz, H-5), 7.16 (1H, t, *J* = 8 Hz, H-5”), 6.86 (1H, d, *J* = 9 Hz, H-6), 6.55 (1H, d, *J* = 9 Hz, H-6”), 6.45 (1H, s, H-2”), 6.44 (1H, d, *J* = 8Hz, H-4”), 6.13 (1H, s, H-3), 4.09 (2H, t, *J* = 6.3 Hz, H-1′), 3.78 (3H, s, H-7”), 3.22 (4H, m, H-3p, H-5p), 2.63 (4H, m, H-2p, H-6p), 2.59 (3H, s, H-11), 2.44 (2H, t, *J* = 7.5 Hz, H-5′), 2.39 (3H, s, H-9), 1.84 (2H, m, H-2′), 1.64 (2H, m, H-4′), 1.49 (2H, m, H-3′); ^13^C NMR (75 MHz, CDCl_3_) δ ppm: 199.5 (C-10), 160.7 (C-3”), 160.1 (C-2), 158.1 (C-7), 152.6 (C-8a), 152.3 (C-4), 150.8 (C-1”), 126.9 (C-5), 126.5 (C-5”), 118.8 (C-8), 114.1 (C-6), 112.7 (C-3), 109.1 (C-4a), 108.5 (C-4”), 104.8 (C-6”), 102.8 (C-2”), 69.1 (C-1′), 58.4 (C-5′), 55.4 (C-3p, C-5p), 53.2 (C-7”), 48.9 (C-2p, C-6p), 32.6 (C-11), 28.9 (C-2′), 26.2 (C-4′), 24.0 (C-3′), 18.9 (C-9); TOF MS ES+: [M + Na]^+^ calcd for C_28_H_34_O_5_N_2_Na 501.2365, found 501.2373.

8-acetyl-7-(5-[4-(2, 5-dimethylphenyl)piperazin-1-yl]penthoxy)-4-methylchromen-2-one (5d). Yield 54%; white solid; m.p.: 92–94 °C; Rf = 0.17; ^1^H NMR (300 MHz, CDCl_3_) δ ppm: 7.55 (1H, d, *J* = 9 Hz, H-5), 7.05 (1H, d, *J* = 6 Hz, H-6), 6.84 (3H, m, H-3”, H-4”, H-6”), 6.13 (1H, s, H-3), 4.09 (2H, t, *J* = 6 Hz, H-1′), 2.94 (4H, m, H-3p, H-5p), 2.59 (7H, m, H-2p, H-6p, H-11), 2.39 (5H, m, H-9, H-5′), 2.30 (3H, s, H-8”), 2.25 (3H, s, H-7”), 1.84 (2H, m, H-2′), 1.54 (4H, m, H-4′, H-3′); ^13^C NMR (75 MHz, CDCl_3_) δ ppm: 199.4 (C-10), 160.1 (C-2), 158.2 (C-7), 152.2 (C-8a), 151.4 (C-4), 150.8 (C-1”), 136.2 (C-5”), 131.0 (C-2”), 129.4 (C-3”), 126.5 (C-5), 123.9 (C-4”), 119.9 (C-8), 119.8 (C-6), 114.1 (C-6”), 112.7 (C-2), 108.5 (C-4a), 69.2 (C-1′), 58.7 (C-5′), 53.9 (C-3p, C-5p), 51.8 (C-2p, C-6p), 32.6 (C-11), 29.1 (C-2′), 26.7 (C-4′), 24.1 (C-3′), 21.4 (C-8”), 18.9 (C-9), 17.6 (C-7”); TOF MS ES+: [M + Na]^+^ calcd for C_29_H_36_O_4_N_2_Na 499.2573, found 499.2560.

8-acetyl-7-(5-[4-(3-fluorophenyl)piperazin-1-yl]penthoxy)-4-methylchromen-2-one (5e). Yield 47%; white solid; m.p.: 117–119 °C; Rf = 0.24; ^1^H NMR (300 MHz, CDCl_3_) δ ppm: 7.55 (1H, d, *J* = 9 Hz, H-5), 7.17 (1H, m, H-5”), 6.86 (1H, d, *J* = 9 Hz, H-6), 6.60 (3H, m, H-2”, H-4”), 6.13 (1H, s, H-3), 4.09 (2H, t, *J* = 7.5 Hz, H-1′), 3.20 (4H, t, *J* = 6 Hz, H-3p, H-5p), 2.57 (7H, m, H-2p, H-6p, H-11), 2.39 (5H, m, H-5′, H-9), 1.84 (2H, m, H-2′), 1.54 (4H, m, H-4′, H-3′); ^13^C NMR (75 MHz, CDCl_3_) δ ppm: 199.4 (C-10), 165.6 (C-3”), 160.2 (C-2), 160.1 (C-7), 158.1 (C-8a), 153.2 (C-4), 150.8 (C-1”), 130.3 (C-5), 126.5 (C-5”), 119.8 (C-8), 114.1 (C-6), 112.7 (C-2), 112.2 (C-4a), 108.4 (C-4”), 105.8 (C-6”), 102.6 (C-2”), 69.2 (C-1′), 58.5 (C-5′), 53.3 (C-3p, C-5p), 48.8 (C-2p, C-6p), 32.6 (C-11), 29.1 (C-2′), 26.7 (C-4′), 24.1 (C-3′), 18.9 (C-9); TOF MS ES+: [M + H]^+^ calcd for C_27_H_32_O_4_N_2_F 467.2346, found 467.2332.

8-acetyl-7-(5-[4-(2-bromophenyl)piperazin-1-yl]penthoxy)-4-methylchromen-2-one (5f). Yield 85%; cream solid; m.p.: 102–103 °C; Rf = 0.22; ^1^H NMR (300 MHz, CDCl_3_) δ ppm: 7.55 (2H, d, *J* = 9 Hz, H-5, H-3”), 7.27 (2H, m, H-6, H-5”), 7.07 (1H, d, *J* = 9 Hz, H-4), 6.90 (1H, m, H-6”), 6.13 (1H, s, H-3), 4.09 (2H, t, *J* = 12 Hz, H-1′), 3.08 (4H, br. s, H-3p, H-5p), 2.56 (4H, br. s, H-2p, H-6p), 2.47 (3H, s, H-11), 2.45 (2H, m, H-5′), 2.42 (3H, s, H-9), 1.84 (2H, m, H-2′), 1.55 (4H, m, H-4′, H-3′); ^13^C NMR (75 MHz, CDCl_3_) δ ppm: 199.4 (C-10), 160.2 (C-2), 158.2 (C-7), 152.2 (C-4), 150.8 (C-8a), 150.8 (C-1”), 133.9 (C-3”), 128.5 (C-5), 126.5 (C-4”), 124.5 (C-5”), 121.1 (C-6”), 120.0 (C-2”), 119.8 (C-8), 114.1 (C-6), 112.7 (C-2), 104.4 (C-4a), 69.2 (C-1′), 58.6 (C-5′), 53.6 (C-3p, C-5p), 51.8 (C-2p, C-6p), 32.6 (C-11), 29.1 (C-2′), 26.7 (C-4′), 24.1 (C-3′), 18.9 (C-9); TOF MS ES+: [M + H]^+^ calcd for C_27_H_32_O_4_N_2_Br 527.1545, found 527.1537.

8-acetyl-7-(5-[4-(3-bromohenyl)piperazin-1-yl]penthoxy)-4-methylchromen-2-one (5g). Yield 64%; white solid; m.p.: 119–121 °C; Rf = 0.19; ^1^H NMR (300 MHz, CDCl_3_) δ ppm: 7.54 (1H, d, *J* = 9 Hz, H-5), 7.09 (1H, m, H-4”), 7.02 (1H, m, H-5”), 6.94 (1H, d, *J* = 6 Hz, H-6), 6.88 (1H, s, H-2”), 6.83 (1H, m, H-6”), 6.14 (1H, s, H-3), 4.09 (2H, t, *J* = 7.5 Hz, H-1′), 3.20 (4H, m, H-3p, H-5p), 2.56 (7H, m, H-2p, H-6p, H-11), 2.39 (5H, m, H-5′, H-9), 1.85 (2H, m, H-2′), 1.56 (4H, m, H-4′, H-3′); ^13^C NMR (75 MHz, CDCl_3_) δ ppm: 199.4 (C-10), 160.2 (C-2), 158.2 (C-7), 152.7 (C-8a), 152.2 (C-4), 150.9 (C-1”), 130.5 (C-5), 126.5 (C-5”), 123.4 (C-3”), 122.4 (C-4”), 119.8 (C-8), 118.8 (C-6), 114.5 (C-2”), 114.1 (C-6”), 112.8 (C-3), 108.5 (C-4a), 69.2 (C-1′), 58.5 (C-5′), 53.3 (C-3p, C-5p), 48.8 (C-2p, C-6p), 32.6 (C-11), 29.1 (C-2′), 26.7 (C-4′), 24.1 (C-3′), 18.9 (C-9); TOF MS ES+: [M + Na]^+^ calcd for C_27_H_31_O_4_N_2_BrNa 549.1365, found 549.1378.

8-acetyl-7-(5-[4-(3, 5-dimethylphenyl)piperazin-1-yl]penthoxy)-4-methylchromen-2-one (5h). Yield 87%; white solid; m.p.: 113–115 °C; Rf = 0.22; ^1^H NMR (300 MHz, CDCl_3_) δ ppm: 7.54 (1H, d, *J* = 9 Hz, H-6), 6.87 (1H, d, *J* = 9 Hz, H-5), 6.54 (3H, d, *J* = 12 Hz, H-2”, H-4”, H-6”), 6.14 (1H, s, H-3), 4.08 (2H, t, *J* = 7.5 Hz, H-1′), 3.18 (4H, t, *J* = 4.5 Hz, H-3p, H-5p), 2.59 (7H, m, H-2p, H-6p, H-11), 2.39 (5H, m, H-9, H-5′), 2.27 (6H, s, H-7”, H-8”), 1.84 (2H, m, H-2′), 1.55 (4H, m, H-4′, H-3′); ^13^C NMR (75 MHz, CDCl_3_) δ ppm: 199.4 (C-10), 160.2 (C-2), 158.2 (C-7), 152.2 (C-8a), 151.6 (C-4), 150.9 (C-1”), 138.8 (C-5”, C-3”), 126.5 (C-5), 121.9 (C-4”), 119.8 (C-8), 114.2 (C-6), 114.1 (C-2”, C-6”), 112.8 (C-3), 108.5 (C-4a), 69.2 (C-1′), 58.7 (C-5′), 53.6 (C-3p, C-5p), 48.4 (C-2p, C-6p), 32.6 (C-11), 29.1 (C-2′), 26.7 (C-4′), 24.1 (C-3′), 21.9 (C-8”), 19.6 (C-7”), 18.9 (C-9); TOF MS ES+: [M + H]^+^ calcd for C_29_H_37_O_4_N_2_ 477.2753, found 477.2735

8-acetyl-7-(5-[4-(2, 3-dichlorophenyl)piperazin-1-yl]penthoxy)-4-methylchromen-2-one (5i). Yield 68%; white solid; m.p.: 137–139 °C; Rf = 0.17; ^1^H NMR (300 MHz, CDCl_3_) δ ppm: 7.55 (1H, d, *J* = 8.7 Hz, H-5), 7.14 (2H, m, H-6, H-4”), 6.96 (1H, m, H-5”), 6.86 (1H, d, *J* = 9 Hz, H-6”), 6.13 (1H, s, H-3), 4.09 (2H, t, *J* = 6.3 Hz, H-1′), 3.11 (4H, br. s, H-3p, H-5p), 2.70 (4H, br. s, H-2p, H-6p), 2.59 (3H, s, H-11), 2.49 (2H, t, *J* = 7.3 Hz, H-5′), 2.39 (3H, s, H-9), 1.84 (2H, m, H-2′), 1.64 (2H, m, H-4′), 1.50 (2H, m, H-3′); ^13^C NMR (75 MHz, CDCl_3_) δ ppm: 199.5 (C-10), 160.1 (C-2), 158.1 (C-7), 152.2 (C-8a, C-4), 150.9 (C-1”), 134.2 (C-3”), 127.7 (C-6), 126.5 (C-5”), 125.1 (C-2”), 119.8 (C-4”), 118.9 (C-6”), 114.1 (C-8, C-6), 112.8 (C-2), 108.5 (C-4a), 69.1 (C-1′), 58.3 (C-5′), 53.3 (C-3p, C-5p), 50.8 (C-2p, C-6p), 32.6 (C-11), 28.9 (C-2′), 25.9 (C-4′), 23.9 (C-3′), 18.9 (C-9); TOF MS ES+: [M + Na]^+^ calcd for C_27_H_30_O_4_N_2_Cl_2_Na 539.1480, found 539.1464.

8-acetyl-7-(5-[4-(2-cyanophenyl)piperazin-1-yl]penthoxy)-4-methylchromen-2-one (5j). Yield 69.6%; white solid; m.p.: 59–61 °C; Rf = 0.29; ^1^H NMR (300 MHz, CDCl_3_) δ ppm: 7.48 (3H, m, H-5, H-3”, H-5”), 7.00 (2H, m, H-6”, H-4”), 6.86 (1H, d, *J* = 8.7 Hz, H-6), 6.13 (1H, s, H-3), 4.09 (2H, t, *J* = 6.3 Hz, H-1′), 3.27 (4H, br. s, H-3p, H-5p), 2.72 (4H, br. s, H-2p, H-6p), 2.51 (3H, s, H-11), 2.49 (2H, t, *J* = 7.3 Hz, H-5′), 2.39 (3H, s, H-9), 1.83 (2H, m, H-2′), 1.62 (2H, m, H-4′), 1.49 (2H, m, H-3′); ^13^C NMR (75 MHz, CDCl_3_) δ ppm: 199.3 (C-10), 159.9 (C-2), 157.9 (C-7), 155.3 (C-8a), 152.1 (C-4), 150.7 (C-1”), 134.3 (C-5), 133.9 (C-3”), 126.3 (C-5”), 122.1 (C-7”), 119.6 (C-4”), 118.8 (C-6”), 118.3 (C-8), 113.9 (C-6), 112.6 (C-3), 108.3 (C-4a), 106.2 (C-2”), 68.9 (C-1′), 58.0 (C-5′), 52.9 (C-3p, C-5p), 50.9 (C-2p, C-6p), 32.5 (C-11), 28.7 (C-2′), 25.7 (C-4′), 23.7 (C-3′), 18.8 (C-9); TOF MS ES+: [M + Na]^+^ calcd for C_28_H_31_O_4_N_3_Na 496.2212, found 496.2226.

8-acetyl-7-(2-bromoethoxy)-4-methylchromen-2-one (6). Yield 80.4 %; yellow solid; m.p.: 140–142 °C; Rf = 0.81; ^1^H NMR (400 MHz, CHCl_3_) δ ppm: 7.57 (1H, d, *J* = 8 Hz, H-5), 6.86 (1H, d, *J* = 12 Hz, H-6), 6.17 (1H, s, H-3), 4.40 (2H, t, *J* = 8 Hz, H-1′), 3.65 (2H, t, *J* = 8 Hz, H-2′), 2.63 (3H, s, H-11), 2.41 (3H, s, H-9); ^13^C NMR (75 MHz, CHCl_3_) δ ppm: 199.2 (C-10), 159.9 (C-2), 156.9 (C-7), 152.1 (C-8a), 150.9 (C-4), 126.6 (C-5), 120.3 (C-8), 114.9 (C-6), 113.3 (C-3), 108.6 (C-4a), 69.1 (C-1′), 32.8 (C-11), 28.6 (C-2′) 18.9 (C-9); TOF MS ES+: [M + H]^+^ calcd for C_14_H_14_O_4_Br: 325.0075 found 325.0064.

8-acetyl-7-(2-[4-(2-methoxyphenyl)piperazin-1-yl]ethoxy)-4-methylchromen-2-one (6a). Yield 76%; white solid; m.p.: 157–159 °C; Rf = 0.23; ^1^H NMR (300 MHz, CDCl_3_) δ ppm: 7.56 (1H, d, *J* = 12 Hz, H-5), 6.93 (5H, m, H-6, H-3”, H-4”, H-5”, H-6”), 6.15 (1H, s, H-3), 4.26 (2H, t, *J* = 6 Hz, H-1′), 3.87 (3H, s, H-7”), 3.11 (4H, br. s, H-3p, H-5p), 2.90 (2H, t, *J* = 6 Hz, H-2′), 2.79 (4H, t, *J* = 6 Hz, H-2p, H-6p), 2.61 (3H, s, H-11), 2.40 (3H, s, H-9); ^13^C NMR (75 MHz, CDCl_3_) δ ppm: 199.3 (C-10), 160.0 (C-1”), 157.4 (C-2), 152.4 (C-7), 152.2 (C-8a), 150.9 (C-4), 140.7 (C-2”), 126.7 (C-5), 123.6 (C-6”), 121.2 (C-5′), 119.9 (C-4”), 118.6 (C-8), 114.6 (C-6), 113.1 (C-3”), 111.5 (C-3), 108.7 (C-4a), 56.8 (C-1′, C-2′), 55.6 (C-3p, C-5p), 53.9 (C-7”), 50.0 (C-2p, C-6p), 32.7 (C-11), 18.9 (C-9); TOF MS ES+: [M + H]^+^ calcd for C_25_H_29_O_5_N_2_ 437.2076, found 437.2059.

8-acetyl-7-(2-[4-(2-fluorophenyl)piperazin-1-yl]ethoxy)-4-methylchromen-2-one (6b). Yield 53%; cream solid; m.p.: 137–139 °C; Rf = 0.15; ^1^H NMR (300 MHz, CDCl_3_) δ ppm: 7.56 (1H, d, *J* = 12 Hz, H-5), 6.97 (5H, m, H-6, H-3”, H-4”, H-5”, H-6”), 6.15 (1H, s, H-3), 4.25 (2H, t, *J* = 6 Hz, H-1′), 3.12 (4H, t, *J* = 6 Hz, H-3p, H-5p), 2.89 (2H, t, *J* = 8 Hz, H-2′), 2.75 (4H, t, *J* = 6 Hz, H-2p, H-6p), 2.61 (3H, s, H-11), 2.40 (3H, s, H-9); ^13^C NMR (75 MHz, CDCl_3_) δ ppm: 199.3 (C-10), 160.0 (C-2), 157.7 (C-7), 154.2 (C-2”), 152.2 (C-8a), 150.9 (C-4), 140.1 (C-1”), 126.6 (C-5), 124.7 (C-5′), 124.6 (C-4”), 119.9 (C-3”), 119.2 (C-6”), 116.4 (C-8), 116.2 (C-6), 112.9 (C-3), 108.6 (C-4a), 67.4 (C-1′), 56.9 (C-2′), 53.9 (C-3p, C-5p), 50.5 (C-2p, C-6p), 32.7 (C-11), 18.9 (C-9); TOF MS ES+: [M + H]^+^ calcd for C_24_H_25_O_4_N_2_FNa 447.1696, found 447.1713.

8-acetyl-7-(2-[4-(3-methoxyphenyl)piperazin-1-yl]ethoxy)-4-methylchromen-2-one (6c). Yield 93%; brown solid; m.p.: 122–124 °C; Rf = 0.33; ^1^H NMR (300 MHz, CDCl_3_) δ ppm: 7.56 (1H, d, *J* = 12 Hz, H-5), 7.17 (1H, t, *J* = 6 Hz, H-5”), 6.90 (1H, d, *J* = 12 Hz, H-6), 6.52 (1H, m, H-6”), 6.43 (2H, m, H-3”, H-4”), 6.14 (1H, s, H-3), 4.25 (2H, t, *J* = 8 Hz, H-1′), 3.78 (3H, s, H-7”), 3.20 (4H, t, *J* = 6 Hz,, H-3p, H-5p), 2.87 (2H, t, *J* = 8 Hz, H-2′), 2.71 (4H, t, *J* = 6 Hz, H-2p, H-6p), 2.61 (3H, s, H-11), 2.39 (3H, s, H-9); ^13^C NMR (75 MHz, CDCl_3_) δ ppm: 199.4 (C-10), 160.8 (C-3”), 160.1 (C-2), 157.7 (C-7), 152.6 (C-8a), 152.2 (C-4), 150.9 (C-1”), 130.0 (C-5), 126.6 (C-5”), 119.9 (C-8), 114.4 (C-6), 112.9 (C-3), 109.1 (C-4a), 108.6 (C-4”), 104.9 (C-6”), 102.8 (C-2”), 67.6 (C-1′), 56.9 (C-2′), 55.4 (C-3p, C-5p), 53.8 (C-2p, C-6p), 49.1 (C-7”), 32.7 (C-11), 18.9 (C-9); TOF MS ES+: [M + Na]^+^ calcd for C_25_H_28_O_5_N_2_Na 459.1896, found 459.1911.

8-acetyl-7-(2-[4-(2,5-dimethylphenyl)piperazin-1-yl]ethoxy)-4-methylchromen-2-one (6d). Yield 93%; cream solid; m.p.: 96–98 °C; Rf = 0.21; ^1^H NMR (300 MHz, CDCl_3_) δ ppm: 7.56 (1H, d, *J* = 12 Hz, H-5), 7.05 (1H, d, *J* = 8 Hz, H-6), 6.91 (1H, d, *J* = 12 Hz H-3”), 6.79 (2H, m, H-4”, H-6”), 6.14 (1H, s, H-3), 4.26 (2H, t, *J* = 8 Hz, H-1′), 2.91 (6H, m, H-3p, H-5p, H-2′), 2.72 (4H, br. s, H-2p, H-6p), 2.62 (3H, s, H-11), 2.40 (3H, s, H-9), 2.30 (3H, s, H-8”), 2.25 (3H, s, H-7”); ^13^C NMR (75 MHz, CDCl_3_) δ ppm: 199.4 (C-10), 160.0 (C-2), 157.7 (C-7), 152.2 (C-8a), 151.2 (C-4), 150.9 (C-1”), 136.3 (C-5”), 131.1 (C-3”, C-5), 129.4 (C-2”), 126.6 (C-4), 124.1 (C-8), 119.9 (C-6), 114.3 (C-6”), 112.9 (C-3), 108.6 (C-4a), 67.5 (C-1′), 56.9 (C-2′), 54.3 (C-3p, C-5p), 51.7 (C-2p, C-6p), 32.6 (C-11), 21.4 (C-9), 18.9 (C-8”), 17.6 (C-7”),); TOF MS ES+: [M + Na]^+^ calcd for C_26_H_30_O_4_N_2_Na 457.2103, found 457.2116.

8-acetyl-7-(2-[4-(3-fluorophenyl)piperazin-1-yl]ethoxy)-4-methylchromen-2-one (6e). Yield 62%; brown solid; m.p.: 86–88 °C; Rf = 0.16; ^1^H NMR (300 MHz, CDCl_3_) δ ppm: 7.56 (1H, d, *J* = 12 Hz, H-5), 7.20 (1H, q, *J* = 8 Hz, H-5”), 6.90 (1H, d, *J* = 12 Hz, H-6), 6.60 (3H, m, H-6”, H-4”, H-2”), 6.15 (1H, s, H-3), 4.26 (2H, t, *J* = 8 Hz, H-1′), 3.21 (4H, t, *J* = 6 Hz,, H-3p, H-5p), 2.87 (2H, t, *J* = 8 Hz, H-2′), 2.72 (4H, t, *J* = 6 Hz, H-2p, H-6p), 2.61 (3H, s, H-11), 2.40 (3H, s, H-9); ^13^C NMR (75 MHz, CDCl_3_) δ ppm: 199.4 (C-10), 165.6 (C-3”), 162.4 (C-2), 160.0 (C-7), 157.5 (C-8a), 152.7 (C-4), 150.9 (C-1”), 130.5 (C-5), 126.7 (C-5”), 119.9 (C-8), 114.5 (C-6), 113.1 (C-3), 111.5 (C-4a), 108.7 (C-4”), 106.6 (C-6”), 102.9 (C-2”), 67.3 (C-1′), 56.8 (C-2′), 55.5 (C-3p, C-5p), 48.5 (C-2p, C-6p), 32.7 (C-11), 18.9 (C-9); TOF MS ES+: [M + Na]^+^ calcd for C_24_H_25_O_4_N_2_FNa 447.1696, found 447.1689.

8-acetyl-7-(2-[4-(2-bromophenyl)piperazin-1-yl]ethoxy)-4-methylchromen-2-one (6f). Yield 72%; white solid; m.p.: 145–147 °C; Rf = 0.17; ^1^H NMR (300 MHz, CDCl_3_) δ ppm: 7.55 (2H, m, H-5, H-3”), 7.27 (1H, m, H-6), 7.05 (1H, H-5”), 6.91 (2H, m, H-4”, H-6”), 6.15 (1H, s, H-3), 4.26 (2H, t, *J* = 8 Hz, H-1′), 3.07 (4H, t, *J* = 4 Hz, H-3p, H-5p), 2.90 (2H, t, *J* = 8 Hz, H-2′), 2.76 (4H, t, *J* = 6 Hz, H-2p, H-6p), 2.62 (3H, s, H-11), 2.40 (3H, s, H-9); ^13^C NMR (75 MHz, CDCl_3_) δ ppm: 199.3 (C-10), 160.0 (C-2), 157.7 (C-7), 152.2 (C-8a), 150.9 (C-4), 150.5 (C-1”), 134.0 (C-3”), 128.5 (C-5), 126.6 (C-4”), 124.7 (C-5”), 121.1 (C-6”), 120.0 (C-2”), 119.9 (C-8), 114.4 (C-6), 112.9 (C-3), 108.6 (C-4a), 67.4 (C-1′), 56.8 (C-2′), 53.9 (C-3p, C-5p), 51.6 (C-2p, C-6p), 32.7 (C-11), 18.9 (C-9); TOF MS ES+: [M + Na]^+^ calcd for C_24_H_25_O_4_N_2_BrNa 507.0895, found 507.0876.

8-acetyl-7-(2-[4-(3-bromohenyl)piperazin-1-yl]ethoxy)-4-methylchromen-2-one (6g). Yield 77%; yellow solid; m.p.: 110–112 °C; Rf = 0.15; ^1^H NMR (300 MHz, CDCl_3_) δ ppm: 7.56 (1H, d, *J* = 6 Hz, H-5), 6.97 (5H, m, H-6, H-2”, H-4”, H-5”, H-6”), 6.15 (1H, s, H-3), 4.26 (2H, t, *J* = 6 Hz, H-1′), 3.20 (4H, t, *J* = 6 Hz,, H-3p, H-5p), 2.87 (2H, t, *J* = 8 Hz, H-2′), 2.71 (4H, t, *J* = 6 Hz, H-2p, H-6p), 2.61 (3H, s, H-11), 2.40 (3H, s, H-9); ^13^C NMR (75 MHz, CDCl_3_) δ ppm: 199.4 (C-10), 160.0 (C-2), 157.6 (C-7), 152.4 (C-8a), 152.2 (C-4), 150.9 (C-1”), 130.6 (C-5), 126.6 (C-5”), 123.4 (C-3”), 122.7 (C-4”), 119.9 (C-8), 119.0 (C-6), 114.7 (C-2”), 114.5 (C-6”), 113.0 (C-3), 108.6 (C-4a), 67.4 (C-1′), 56.8 (C-2′), 53.6 (C-3p, C-5p), 48.6 (C-2p, C-6p), 32.7 (C-11), 18.9 (C-9); TOF MS ES+: [M + Na]^+^ calcd for C_24_H_25_O_4_N_2_BrNa 507.0895, found 507.0909.

8-acetyl-7-(2-[4-(3,5-dimethylphenyl)piperazin-1-yl]ethoxy)-4-methylchromen-2-one (6h). Yield 97%; brown solid; m.p.: 120–121 °C; Rf = 0.33; ^1^H NMR (300 MHz, CDCl_3_) δ ppm: 7.56 (1H, d, *J* = 12 Hz, H-6), 6.90 (1H, d, *J* = 12 Hz, H-5), 6.53 (3H, d, *J* = 12 Hz, H-2”, H-4”, H-6”), 6.14 (1H, s, H-3), 4.25 (2H, t, *J* = 6 Hz, H-1′), 3.18 (4H, t, *J* = 8 Hz, H-3p, H-5p), 2.87 (2H, t, *J* = 6 Hz, H-2′), 2.71 (4H, t, *J* = 6 Hz, H-2p, H-6p), 2.61 (3H, s, H-11), 2.40 (3H, s, H-9), 2.27 (6H, s, H-7”, H-8”); ^13^C NMR (75 MHz, CDCl_3_) δ ppm: 199.4 (C-10), 160.0 (C-2), 157.7 (C-7), 152.2 (C-8a), 151.3 (C-4), 150.9 (C-1”), 138.8 (C-5”, C-3”), 126.6 (C-5), 122.1 (C-4”), 119.9 (C-8), 114.4 (C-2”, C-6”), 114.3 (C-6), 112.9 (C-3), 108.6 (C-4a), 67.5 (C-1′), 56.9 (C-2′), 53.9 (C-3p, C-5p), 49.3 (C-2p, C-6p), 32.7 (C-11), 21.9 (C-8”, C-7”), 18.9 (C-9); TOF MS ES+: [M + Na]^+^ calcd for C_26_H_30_O_4_N_2_Na 457.2103, found 457.2086.

8-acetyl-7-(2-[4-(2,3-dichlorophenyl)piperazin-1-yl]ethoxy)-4-methylchromen-2-one (6i). Yield 85%; white solid; m.p.: 159–161 °C; Rf = 0.23; ^1^H NMR (300 MHz, CDCl_3_) δ ppm: 7.57 (1H, d, *J* = 12 Hz, H-5), 7.14 (2H, m, H-6, H-4”), 6.94 (2H, m, H-5”, H-6”), 6.14 (1H, s, H-3), 4.26 (2H, t, *J* = 6 Hz, H-1′), 3.07 (4H, br. s, H-3p, H-5p), 2.90 (2H, t, *J* = 8 Hz, H-2′), 2.76 (4H, br. s, H-2p, H-6p), 2.61 (3H, s, H-11), 2.40 (3H, s, H-9; ^13^C NMR (75 MHz, CDCl_3_) δ ppm: 199.3 (C-10), 160.0 (C-2), 157.7 (C-7), 152.2 (C-8a), 151.1 (C-4), 150.9 (C-1”), 134.2 (C-3”), 127.7 (C-5), 126.6 (C-5”), 124.9 (C-2”), 119.9 (C-4”), 118.8 (C-6”, C-8), 114.4 (C-6), 112.9 (C-3), 108.6 (C-4a), 67.4 (C-1′), 56.8 (C-2′), 53.8 (C-3p, C-5p), 51.2 (C-2p, C-6p), 32.7 (C-11), 18.9 (C-9); TOF MS ES+: [M + Na]^+^ calcd for C_24_H_24_O_4_N_2_Cl_2_Na 497.1011, found 497.1026.

8-acetyl-7-(2-[4-(2-cyanophenyl)piperazin-1-yl]ethoxy)-4-methylchromen-2-one (6j). Yield 96%; cream solid; m.p.: 155–157 °C; Rf = 0.14; ^1^H NMR (300 MHz, CDCl_3_) δ ppm: 7.54 (3H, m, H-5, H-3”, H-5”), 7.01 (2H, m, H-6”, H-4”), 6.91 (1H, d, *J* = 12 Hz, H-6), 6.15 (1H, s, H-3), 4.26 (2H, t, *J* = 8 Hz, H-1′), 3.24 (4H, t, *J* = 6 Hz, H-3p, H-5p), 2.91 (2H, t, *J* = 8 Hz, H-2′), 2.79 (4H, t, *J* = 6 Hz, H-2p, H-6p), 2.62 (3H, s, H-11), 2.41 (3H, s, H-9); ^13^C NMR (75 MHz, CDCl_3_) δ ppm: 199.3 (C-10), 160.0 (C-2), 157.6 (C-7), 155.5 (C-8a), 152.2 (C-4), 150.9 (C-1”), 134.5 (C-5”), 134.0 (C-3”), 126.6 (C-5), 122.2 (C-7”), 119.9 (C-4”), 118.9 (C-6”), 118.5 (C-8), 114.4 (C-6), 112.9 (C-3), 108.7 (C-4a), 106.3 (C-2”), 67.2 (C-1′), 56.7 (C-2′), 53.7 (C-3p, C-5p), 51.4 (C-2p, C-6p), 32.7 (C-11), 18.9 (C-9); TOF MS ES+: [M + Na]^+^ calcd for C_25_H_25_O_4_N_3_Na 454.1743, found 454.1744.

### 3.2. Docking Studies

In the computational part of this study we used a protocol similar to our previous investigation on this topic [20,21,24]. In short, the 3D models of 5HT_1A/2A_ receptors were prepared using homology modelling based on the crystal structures of dopamine D3 receptor (PBD code: 3PBL) and β1 adrenergic receptor (PDB code: 2Y00), respectively [34,35]. We used flexible docking algorithm as implemented in Autodock 4.2 [36] with the ligand and the following residues described in a flexible manner: D116, V117, W358, F361, F362, N386, and Y390 for 5HT_1A_ receptor and D155, V156, S159, W336, F339, F340, N363, and Y370 for 5HT_2A_ receptor. We used a 48 × 52 × 40 Å ^3^ box and 60 × 54 × 50 Å^3^ box for 5HT_2A_ receptor, centered in both cases on the binding site. We also used standard Autodock 4.2 parameters for the Lamarckian genetic algorithm, but with 100 runs for each ligand-receptor pair for a total of 132 separate runs. Schematic figures of the ligand binding sites have been prepared using the Ligand Interaction Diagram (Schrödinger Release 2020–4: Maestro, Schrödinger, LLC, New York, NY, USA, 2020).

### 3.3. Biological Evaluation

#### 3.3.1. Membrane Preparation

Male Sprague–Dawley rats were decapitated, their brains removed, and placed on ice. Hippocampi were dissected and homogenized with a glass homogenizer in 30 vol. ice-cold TED buffer (50 mM Tris-HCl, 1 mM EDTA, 1mM dithiotheritol, pH 7.4). Next, the homogenate was centrifuged at 21,000× *g* for 30 min at 4 °C. The pellet was suspended in 30 vol TED buffer (pH 7.4) and incubated in a water bath for 10 min at 37 °C to remove endogenous serotonin. The suspension was centrifuged again at 21,000× *g* for 30 min at 4 °C. The pellet was resuspended in 30 vol. TED buffer (pH 7.4) and the centrifugation step was repeated. The final pellet was suspended in 10 vol 50 mM Tris-HCl (pH 7.4) and stored at −80 °C until use.

#### 3.3.2. Antagonist Activity for the 5-HT_1A_ Receptor

Compounds were dissolved in 9.5% DMSO and 0.5% Kolliphor^®^ EL (Sigma Aldrich, Taufkirchen, Germany). Serial dilutions of the compounds tested (10^−10^–10^−5^ M) were incubated in triplicate with 0.8 nM [^35^S]GTPγS in assay buffer (50 mM Tris-HCl, pH = 7.4, 1 mM EGTA, 3 mM MgCl_2_, 100 mM NaCl, 30 µM GDP) and 8-OH-DPAT (final concentration 1.4 × 10^−7^ M) in the final assay volume of 250 µL. Hippocampal homogenates (15 μg/mL) were added to each tube as the 5-HT_1A_ receptor source. The final DMSO and Kolliphor^®^ EL concentrations were 0.95% and 0.05%, respectively. Non-specific binding was determined with 10 µM of unlabeled GTPγS. The reaction mixture was incubated for 90 min at 37 °C in a volume of 250 µL. Next, 96-well Unifilter^®^ Plates (Perkin Elmer, Waltham, MA, USA) were presoaked for 1 h with 50 mM Tris-HCl (pH = 7.4) before harvesting. The reaction was terminated by vacuum filtration onto filter plates with the FilterMate Harvester^®^ (Perkin Elmer, Waltham, MA, USA). The samples were then rapidly washed with 2 mL of 50 mM Tris-HCl (pH = 7.4) buffer. Filter plates were dried for 2 h at 50 °C. After drying, 45 µL of EcoScint-20 scintillant (Perkin Elmer) was added to every well. Radioactivity was counted in a Trilux MicroBeta^2^ counter (Perkin Elmer). Data were analyzed with GraphPad Prism 5.0 software (GraphPad Software, San Diego, CA, USA, www.graphpad.com). Curves were fitted with a one-site non-linear regression model. Efficacy (Emax) and half maximal inhibitory concentration (IC50) were calculated from the Cheng–Prusoff equation and expressed as means ± SEM.

#### 3.3.3. Membrane Preparation for the 5-HT_2A_ Receptor Binding

Male SD rats were decapitated and their brains removed and placed on ice. Frontal cortices were homogenized with a glass homogenizer in 30 vol ice-cold homogenization buffer (50 mM Tris-HCl. 1 mM EDTA. 5 mM MgCl_2_. pH 7.4). Next, the homogenate was centrifuged at 20,000× *g* for 15 min at 4 °C. The pellet was suspended in 30 vol 50 mM Tris-HCl (pH 7.4) and incubated in a water bath for 15 min at 37 °C to remove endogenous serotonin. The suspension was again centrifuged at 20,000× *g* for 15 min at 4 °C. The pellet was resuspended in 10 vol. 50 mM Tris-HCl (pH 7.4) and the centrifugation step was repeated. The final pellet was suspended in 10 vol 50 mM Tris-HCl (pH 7.4) and stored at −80 °C.

#### 3.3.4. 5-HT_2A_ Competition Binding Assay

For the 5-HT_2A_ assay frontal cortex homogenates (160 µg protein/mL) were incubated in triplicate with 1 nM [^3^H]ketanserin for 60 min at 36 °C in a 50 mM Tris-HCl (pH 7.4) buffer containing 0.1% ascorbate (3 mM CaCl_2_ and 10 µM pargyline) and increasing the concentrations (10^−9^–10^−5^ M) of the compound of interest. Non-specific binding was determined in the presence of 10 μM mianserin. After incubation, the reaction mixture was deposited onto UniFilter-96 GF/B plates with the aid of a FilterMate-96 Harvester. Filter plates were presoaked beforehand with 0.4% PEI for 1 h. Next, each filter well was washed with 1.75 mL of 50 mM Tris-HCl (pH 7.4) and left to dry on a heating block set to 50 °C for 2 h. Then 45 µL of Microscint-20 scintillation fluid was added to each filter well and left to equilibrate overnight. Filter-bound radioactivity was counted in a MicroBeta^2^ Microplate Counter. Binding curves were fitted with one site non-linear regression. Affinity was presented as the inhibitory constant (pK_i_ ± SEM and K_i_ ± SEM) from two or three separate experiments.

## 4. Conclusions

**Figure 3:**
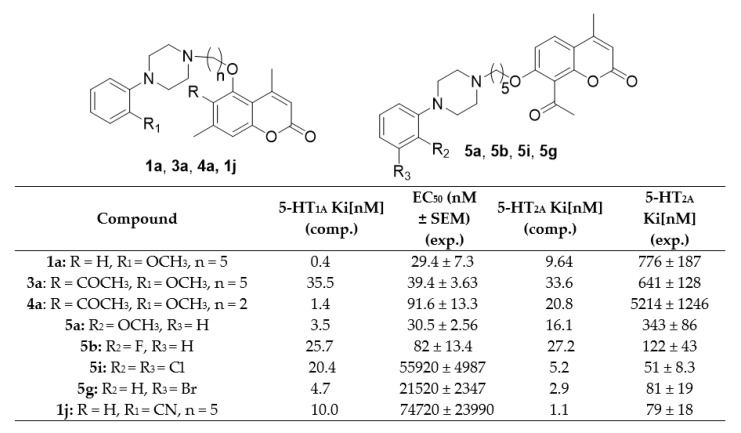
Summary of the results for the most active compounds.

Sixty new aryl-piperazinyl derivatives of 5-hydroxy-4,7-dimethylchromen-2-one (A), 6-acetyl-5-hydroxy-4,7-dimethylchromen-2-one (B) and 8-acetyl-7-hydroxy-4-methylchromen-2-one (C) were designed, synthesized, and evaluated in silico and experimentally for their 5-HT_1A_ and 5-HT_2A_ receptor-binding affinities. Figure 3 present summary of the results for the most active compounds. Five compounds showed high antagonistic activities against the 5-HT_1A_ receptor (**1a**, **3a**, **4a**, **5a**, and **5b**), though lower than WAY-100635, the reference 5HT_1A_ antagonist, while three compounds showed moderate affinity for 5-HT_2A_ receptors (**5i**, **1j**, and **5g**) with respect to ketanserin. The designed derivatives had two- or five-carbon alkyl linkers between coumarin and arylpiperazinyl moiety. The studies showed that the new compounds showed less profound binding for the tested serotonin receptors than the derivatives containing three- or four-carbon linkers, which we described in our previous works. While the differences in 5HT_1A_ activities between the three-carbon or four-carbon linker derivatives were minimal, further shortening or lengthening of the linker quite significantly lowered the potency of coumarin derivative to bind to this receptor. Overall, the results for the series of 5- and 7-hydroxycoumarin derivatives obtained in this and our previous investigations on this topic provide an exhaustive structure–activity relationship database, which can be used in future search for novel agents acting on serotonin receptors, either based on coumarin derivatives or other organic scaffolds.

## Figures and Tables

**Table 1:** Computational Ki values for compounds for compounds of series **1**, **2**, and **3**.

Compound	5-HT_1A_ Ki[nM]	5-HT_2A_ Ki[nM]
**1**	206.3	1023
**1a**	0.4	9.64
**1b**	3.2	36.1
**1c**	1.2	7.7
**1d**	2.6	19.6
**1e**	5.0	9.2
**1f**	10.8	30.5
**1g**	4.6	6.4
**1h**	1.6	2.4
**1i**	4.0	2.0
**1j**	10.0	1.1
**2**	922.3	6610
**2a**	3.9	22.3
**2b**	6.6	44.8
**2c**	8.0	29.8
**2d**	1.5	12.7
**2e**	13.1	40.3
**2f**	31.9	12.4
**2g**	4.6	4.4
**2h**	9.4	20.6
**2i**	5.9	11.3
**2j**	16.6	18.9
**3**	154.4	3080
**3a**	35.5	33.6
**3b**	23.3	53.2
**3c**	5.9	6.8
**3d**	928.5	7.0
**3e**	106.2	59.7
**3f**	1.9	1.1
**3g**	3.2	10.0
**3h**	12.4	11.8
**3i**	3.6	5.2
**3j**	37.2	31.5
ketanserin	71.3	58.7
WAY-100635	50.5	73.9

**Table 2:** Computational Ki values for compounds for compounds of series **4**, **5,** and **6**.

Compound	5-HT_1A_ Ki[nm]	5-HT_2A_ Ki[nm]
**4**	429.2	3270
**4a**	1.4	20.8
**4b**	3.1	44.7
**4c**	10.3	16.3
**4d**	10.8	30.8
**4e**	52.2	17.1
**4f**	17.7	25.5
**4g**	3.9	1.6
**4h**	7.3	17.8
**4i**	2.0	5.1
**4j**	2.7	2.1
**5**	57.7	2100
**5a**	3.5	16.1
**5b**	25.7	27.2
**5c**	72.2	5.8
**5d**	8.3	2.7
**5e**	12.5	3.5
**5f**	58.5	5.2
**5g**	4.7	2.9
**5h**	20.6	5.0
**5i**	20.4	5.2
**5j**	3.7	0.9
**6**	171.6	4430
**6a**	17.3	10.8
**6b**	0.7	25.4
**6c**	1.9	8.7
**6d**	2.6	5.8
**6e**	3.7	36.9
**6f**	4.6	6.9
**6g**	0.3	10.3
**6h**	1.5	9.9
**6i**	2.8	9.9
**6j**	10.0	2.9
ketanserin	71.3	58.7
WAY-100635	50.5	73.9

**Table 3:** Antagonistic activity of compounds for the 5-HT_1A_ receptor.

Compound	pIC_50_ ± SEM	EC_50_ (nM ± SEM)
**1a**	7.5 ± 0.21	29.4 ± 7.3
**1b**	6.0 ± 0.2	980 ± 207
**1c**	5.8 ± 0.22	1698 ± 358
**1d**	not tested	not tested
**1e**	4.6 ± 0.3	24,320 ± 3730
**1f**	4.7 ± 0.14	20,920 ± 2072
**1g**	not tested	not tested
**1h**	3.7 ± 0.18	198,100 ± 51,350
**1i**	4.8 ± 0.14	13,740 ± 1733
**1j**	4.1 ± 0.38	74,720 ± 23,990
**2a**	5.7 ± 0.18	1881 ± 427
**2b**	no activity	no activity
**2c**	4.7 ± 0.11	19,130 ± 2363
**2d**	no activity	no activity
**2f**	no activity	no activity
**2h**	no activity	no activity
**2i**	3.4 ± 1.4	365,800 ± 46,480
**2j**	no activity	no activity
**3a**	7.4 ± 0.17	39.4 ± 3.63
**3b**	4.7 ± 0.1	19,200 ± 1177
**3c**	5.8 ± 0.2	1549 ± 190
**3d**	5.0 ± 0.17	9434 ± 1037
**3e**	6.15 ± 0.11	702 ± 112
**3f**	6.16 ± 0.14	689 ± 138
**3g**	5.89 ± 0.12	1284 ± 254
**3h**	5.9 ± 0.16	1245 ± 112
**3i**	4.8 ± 0.11	15,400 ± 1290
**3j**	not tested	not tested
**4a**	7.0 ± 0.11	91.6 ± 13.3
**4b**	5.3 ± 0.13	5003 ± 218
**4c**	5.3 ± 0.07	5007 ± 117
**4d**	4.1 ± 0.04	74,730 ± 4576
**4e**	6.3 ± 0.18	527 ± 191
**4f**	5.08 ± 0.10	8317 ± 1497
**4g**	4.9 ± 0.11	11,070 ± 1395
**4h**	4.4 ± 0.11	26,840 ± 5904
**4i**	5.5 ± 0.15	3098 ± 148
**4j**	6.3 ± 0.06	538 ± 105
**5a**	7.5 ± 0.11	30.5 ± 2.56
**5b**	7.1 ± 0.13	82 ± 13.4
**5c**	4.6 ± 0.14	22,860 ± 2899
**5d**	4.9 ± 0.05	12,940 ± 802
**5e**	4.5 ± 0.12	33,890 ± 2563
**5f**	4.5 ± 0.11	27,440 ± 1986
**5g**	4.6 ± 0.13	21,520 ± 2347
**5h**	4.5 ± 0.14	32,890 ± 2417
**5i**	4.2 ± 0.41	55,920 ± 4987
**5j**	4.8 ± 0.22	13,860 ± 2059
**6a**	5.5 ± 0.1	2735 ± 626
**6b**	4.3 ± 0.07	45,330 ± 8178
**6c**	4.3 ± 0.09	47,150 ± 9511
**6d**	4.2 ± 0.11	59,890 ± 12,467
**6e**	4.0 ± 0.11	98,950 ± 15,583
**6f**	4.3 ± 0.07	46,850 ± 8125
**6g**	not tested	not tested
**6h**	4.3 ± 0.09	47,790 ± 9556
**6i**	3.7 ± 0.24	204,000 ± 56,920
**6j**	5.1 ± 0.1	7804 ± 1876
**WAY-100635**	8.4 ± 0.12	4.3 ± 0.86

**Table 4:** G-protein enhancing effect of compounds **2e** and **2f** at the 5-HT_1A_ receptor.

Compound	pEC_50_	EC_50_ (nM ± SEM)	Emax (% ± SEM)
**2e**	6.7 ± 0.25	178.7 ± 39.3	118 ± 1.48
**2g**	5.2 ± 0.73	5083 ± 2636	113 ± 6.5

**Table 5:** 5-HT_2A_ receptor binding of investigated compounds.

Compound	pK_i_ (M ± SEM)	K_i_ (nM ± SEM)
**1a**	6.191 ± 0.12	776 ± 187
**1b**	5.90 ± 0.2	1263 ± 479
**1c**	6.21 ± 0.13	610 ± 139
**1d**	not tested	not tested
**1e**	5.63 ± 0.16	2346 ± 673
**1f**	5.81 ± 0.09	1548 ± 323
**1g**	not tested	not tested
**1h**	5.74 ± 0.14	1818 ± 636
**1i**	5.84 ± 0.11	1452 ± 653
**1j**	7.10 ± 0.12	79 ± 18
**2a**	5.77 ± 0.15	1713 ± 582
**2b**	5.67 ± 0.11	2129 ± 662
**2c**	6.33 ± 0.10	458 ± 109
**2d** **2e**	5.13 ± 0.12 not tested	7321 ± 2079 not tested
**2f** **2g**	5.02 ± 0.12 5.45 ± 0.11	9600 ± 2841 3538 ± 1135
**2h**	5.53 ± 0.13	2920 ± 992
**2i**	5.37 ± 0.17	4306 ± 1550
**2j**	5.25 ± 0.14	5619 ± 1854
**3a**	6.19 ± 0.12	641 ± 128
**3b**	5.93 ± 0.11	1223± 166
**3c**	6.31 ± 0.14	492 ± 99.7
**3d**	5.71 ± 0.14	1938 ± 388
**3e**	5.85 ± 0.09	1421 ± 426.3
**3f**	6.45 ± 0.09	354 ± 110
**3g**	6.18 ± 0.11	664 ± 154
**3h**	5.75 ± 0.13	1796 ± 521
**3i**	6.31 ± 0.09	486 ± 102
**3j**	not tested	not tested
**4a**	5.28 ± 0.13	5214 ± 1246
**4b**	5.15 ± 0.12	7053 ± 1650
**4c**	6.29 ± 0.15	513 ± 137
**4d**	4.93 ± 0.14	11,870 ± 3086
**4e**	5.41 ± 0.11	3859 ± 810
**4f**	4.97 ± 0.16	10,610 ± 3045
**4g**	5.40 ± 0.07	3986 ± 745
**4h**	5.72 ± 0.08	1924 ± 382
**4i**	5.78 ± 0.13	1642 ± 344
**4j**	5.61 ± 0.04	2429 ± 374
**5a**	6.46 ± 0.11	343 ± 86
**5b**	6.91 ± 0.17	122 ± 43
**5c**	6.84 ± 0.13	144 ± 38
**5d**	5.82 ± 0.08	1497 ± 279
**5e**	5.67 ± 0.09	2114 ± 420
**5f**	6.96 ± 0.10	108 ± 24
**5g**	7.09 ± 0.13	81 ± 19
**5h**	6.54 ± 0.09	291 ± 57
**5i**	7.20 ± 0.07	51 ± 8.3
**5j**	5.59 ± 0.13	2551 ± 765
**6a**	6.12 ± 0.11	752 ± 171
**6b**	5.82 ± 0.11	1509 ± 344
**6c**	6.03 ± 0.09	920 ± 160
**6d**	5.69 ± 0.10	2046 ± 405
**6e**	5.78 ± 0.08	1671 ± 274
**6f**	5.82 ± 0.08	1503 ± 268
**6g**	5.83 ± 0.12	1494 ± 466
**6h**	6.32 ± 0.11	479 ± 96
**6i**	6.12 ± 0.11	755 ± 178
**6j**	6.30 ± 0.11	496 ± 121
**ketanserin**	8.44 ± 0.07	3.6 ± 0.5

## Data Availability

Data is contained within the article.

## Supplementary Materials

The following are available online at https://www.mdpi.com/1424-8247/14/3/179/s1.
