# Supplementary material for: Design, Synthesis, and Biological Evaluation of a Series of 5- and 7-Hydroxycoumarin Derivatives as 5-HT1A Serotonin Receptor Antagonists
Source: Pharmaceuticals (Basel). 2021 Feb 24;14(3):179. doi: 10.3390/ph14030179 (PMC7996328; doi:10.3390/ph14030179)
Supplement: Supplementary file 1 [file pharmaceuticals-14-00179-s001.pdf]

Design, Synthesis and Biological Evaluation of a Series of 5- and 7-hydroxycoumarin Derivatives as 5-HT_{1A} Serotonin Receptor Antagonists

Kinga Ostrowska [1,*], Anna Leśniak[2], Zuzanna Czarnocka [1], Jagoda Chmiel [1], Magdalena Bujalska-Zadrożny [2] and Bartosz Trzaskowski [3]

[1] Department of Organic Chemistry, Faculty of Pharmacy, Medical University of Warsaw, 1 Banacha Str., 02 097 Warsaw, Poland, kostrowska@wum.edu.pl, zuzanna.czarnocka@gmail.com, jagodachmiel178096@gmail.com

[2] Department of Pharmacodynamics, Faculty of Pharmacy, Centre for Preclinical Research and Technology, Medical University of Warsaw, 1 Banacha Str., 02-097 Warsaw, Poland, anna.lesniak@wum.edu.pl, magdalena.bujalska@wum.edu.pl

[3] Centre of New Technologies, University of Warsaw, 2C Banacha Str., 02-097 Warszawa, b.trzaskowski@cent.uw.edu.pl

* Correspondence: kostrowska@wum.edu.pl ; Tel.: +48-22-572-0669

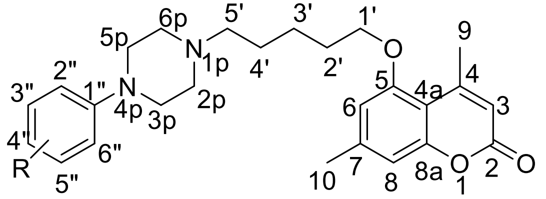

1a-1j

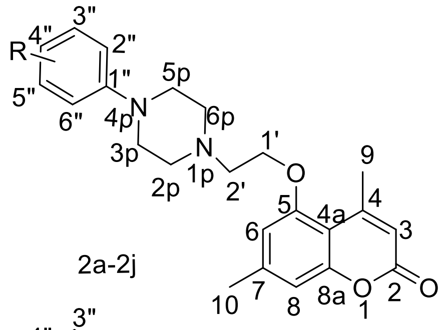

2a-2j

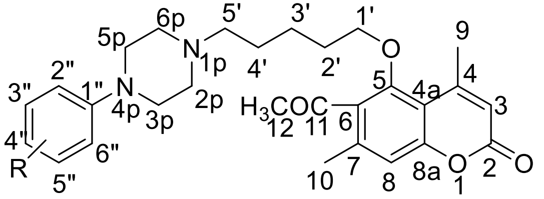

3a-3j

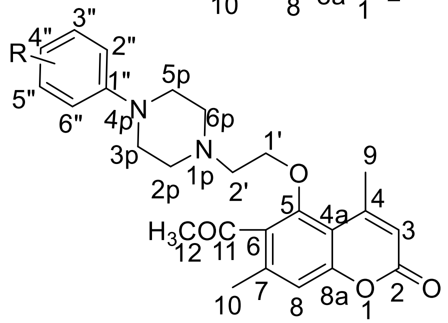

4a-4j

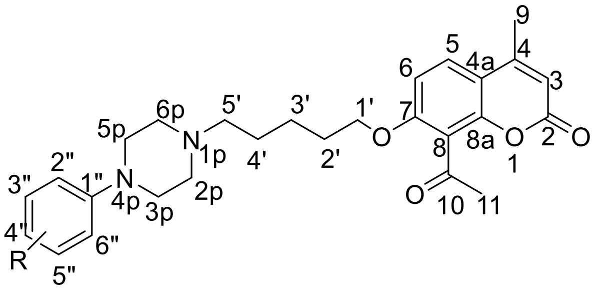

5a-5j

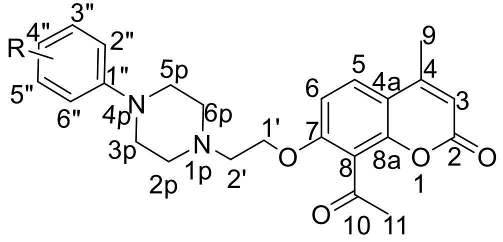

6a-6j

Figure 1S. Atom Numbering of compounds 1a-1j, 2a-2j, 3a-3j, 4a-4j, 5a-5j, 6a-6j.

^{1}H NMR and ^{13}C NMR Spectra

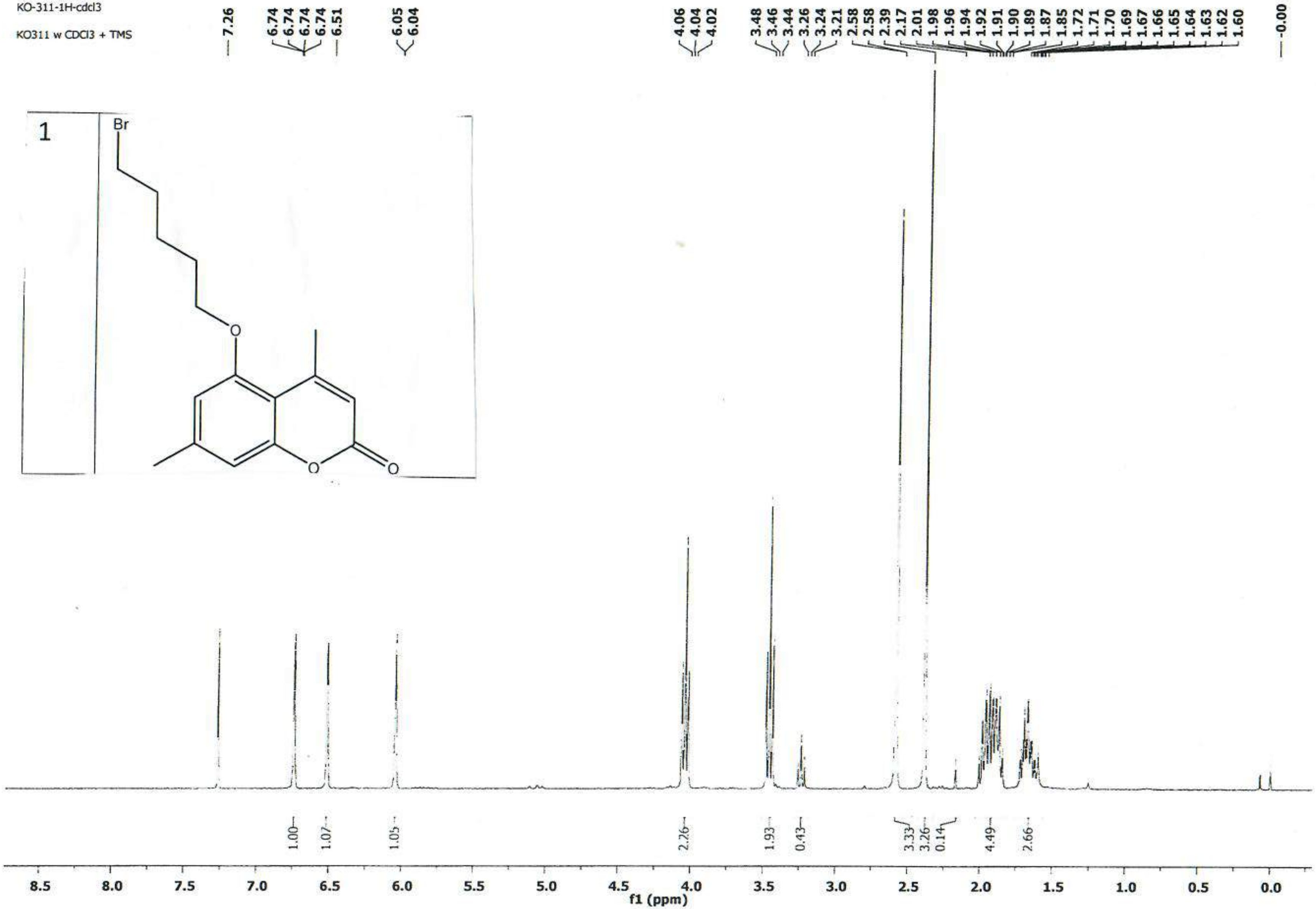
KO-311-1H-cdcl3
KO311 w CDCl3 + TMS
7.26
6.74
6.74
6.74
6.74
6.51
6.05
6.04
4.06
4.04
4.02
3.48
3.46
3.44
3.26
3.24
3.21
2.58
2.58
2.39
2.17
2.01
1.98
1.96
1.94
1.92
1.91
1.90
1.89
1.87
1.85
1.72
1.71
1.70
1.69
1.67
1.66
1.65
1.64
1.63
1.62
1.60
-0.00
1
Br
O
O
O
1.00
1.07
1.05
2.26
1.93
0.43
3.33
3.26
0.14
4.49
2.66
8.5
8.0
7.5
7.0
6.5
6.0
5.5
5.0
4.5
4.0
3.5
3.0
2.5
2.0
1.5
1.0
0.5
0.0
f1 (ppm)

4

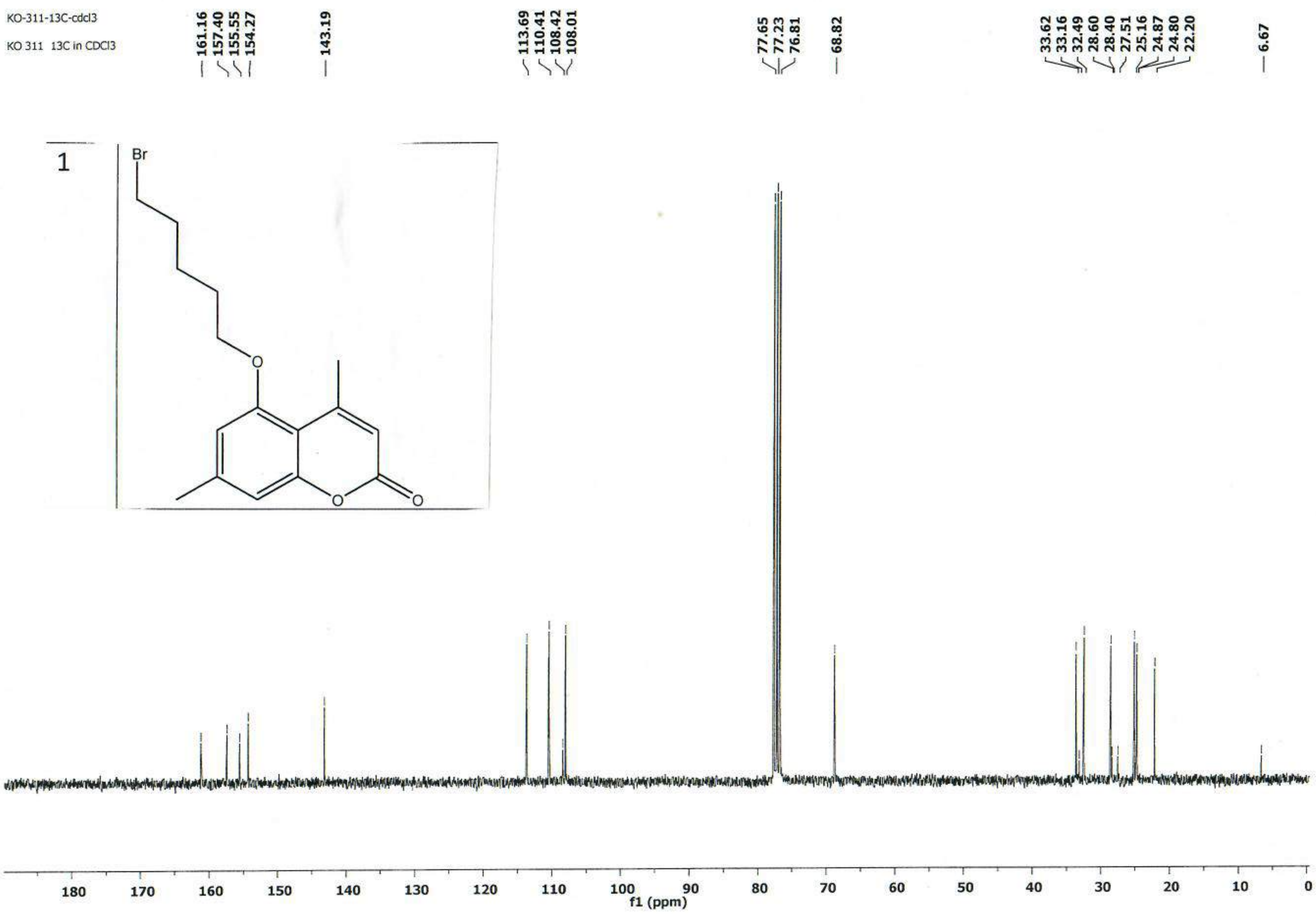
KO-311-13C-cdcl3
KO 311 13C in CDCl3
161.16
157.40
155.55
154.27
143.19
113.69
110.41
108.42
108.01
77.65
77.23
76.81
68.82
33.62
33.16
32.49
28.60
28.40
27.51
25.16
24.87
24.80
22.20
6.67
1
Br
O
O
O
180
170
160
150
140
130
120
110
100
90
80
70
60
50
40
30
20
10
0
f1 (ppm)

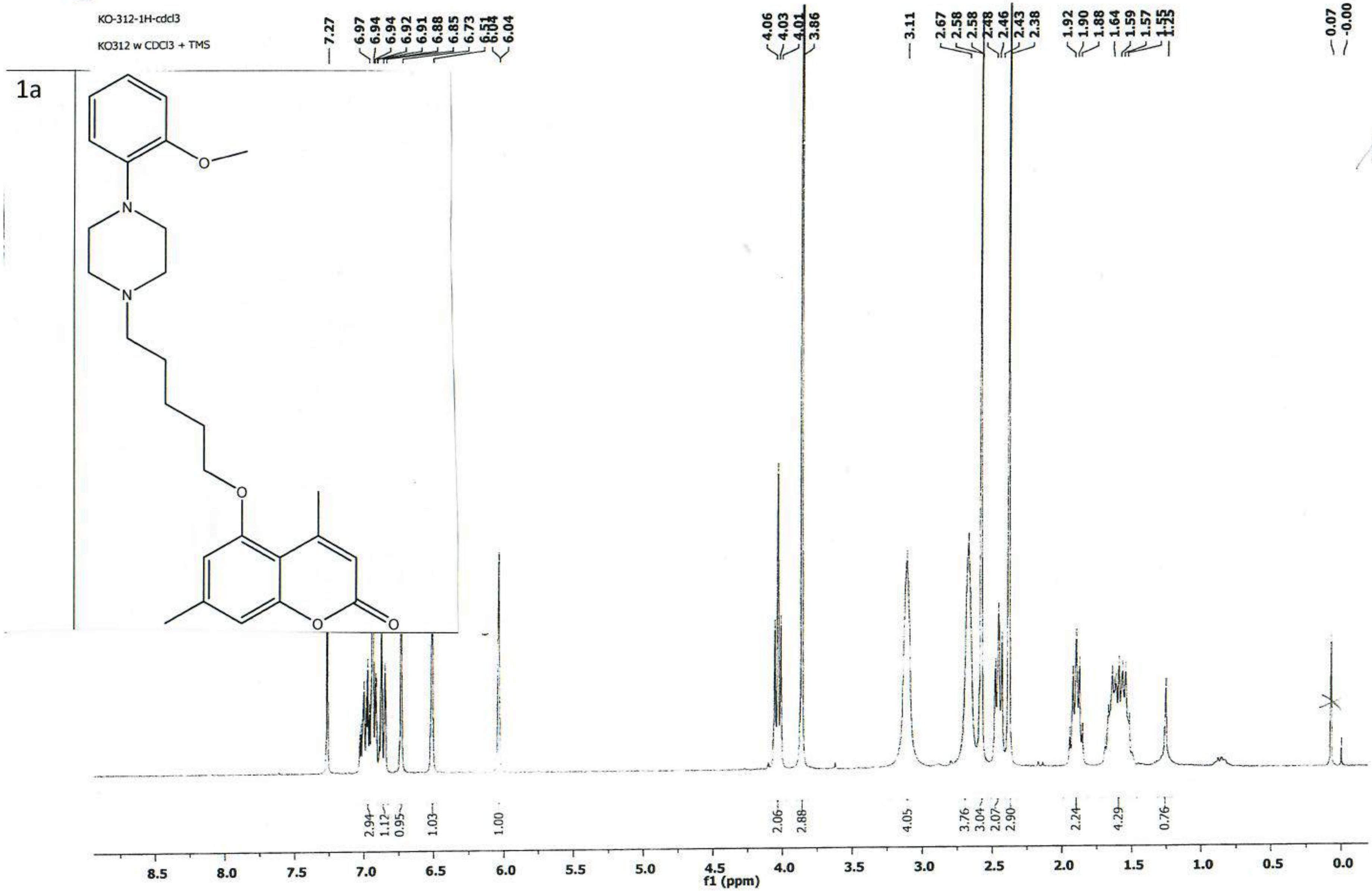
KO-312-1H-cdcl3
KO312 w CDCl3 + TMS
1a
7.27
6.97
6.94
6.94
6.92
6.91
6.88
6.85
6.73
6.51
6.04
6.04
4.06
4.03
4.01
3.86
3.11
2.67
2.58
2.58
2.48
2.46
2.43
2.38
1.92
1.90
1.88
1.64
1.59
1.57
1.55
1.55
0.07
-0.00
2.94
1.12
0.95
1.03
1.00
2.06
2.88
4.05
3.76
3.04
2.07
2.90
2.24
4.29
0.76
8.5
8.0
7.5
7.0
6.5
6.0
5.5
5.0
4.5
f1 (ppm)
4.0
3.5
3.0
2.5
2.0
1.5
1.0
0.5
0.0

1a

1a

1a

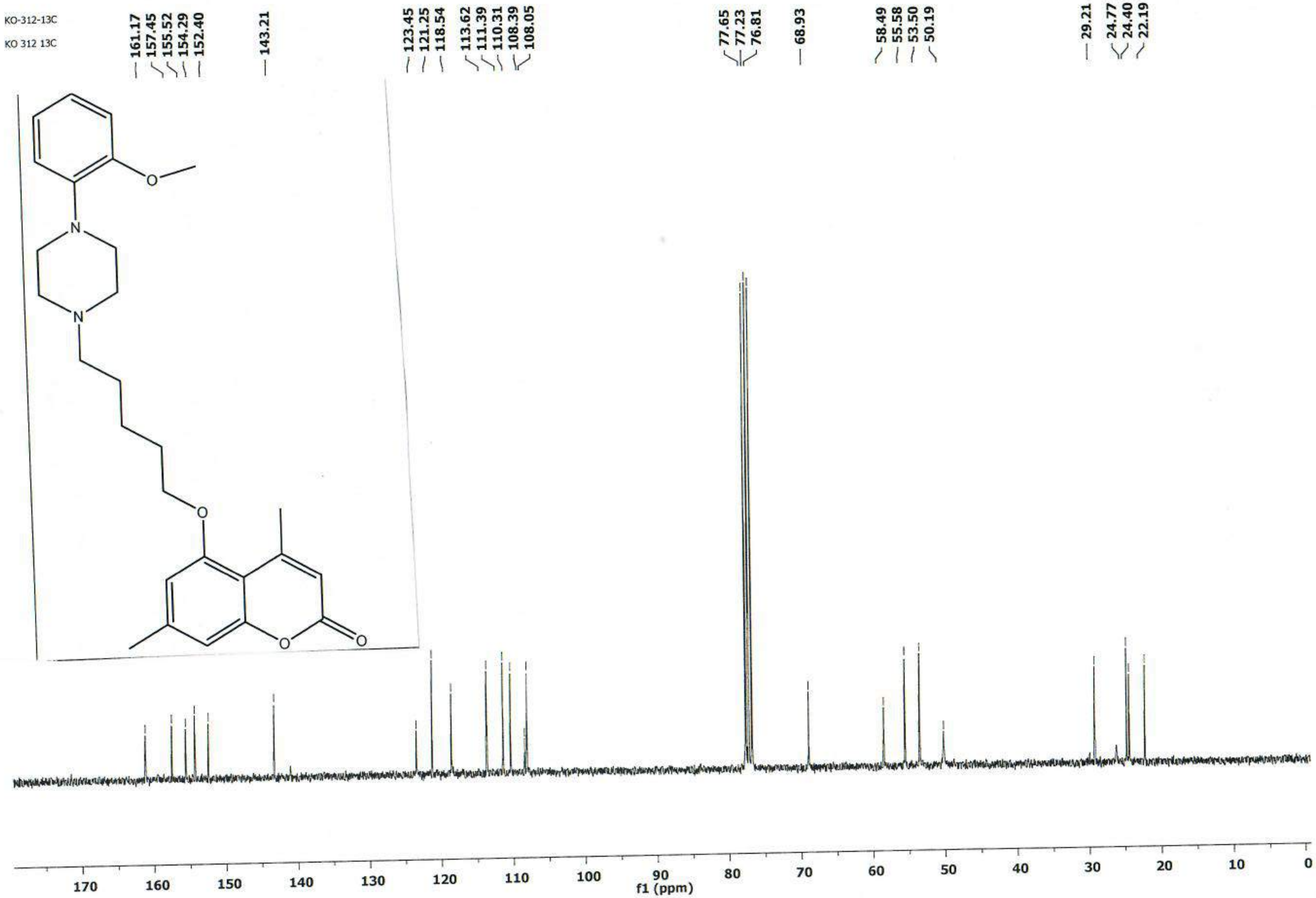
KO-312-13C
KO 312 13C
161.17
157.45
155.52
154.29
152.40
143.21
123.45
121.25
118.54
113.62
111.39
110.31
108.39
108.05
77.65
77.23
76.81
68.93
58.49
55.58
53.50
50.19
29.21
24.77
24.40
22.19
170 160 150 140 130 120 110 100 90 80 70 60 50 40 30 20 10 0
f1 (ppm)

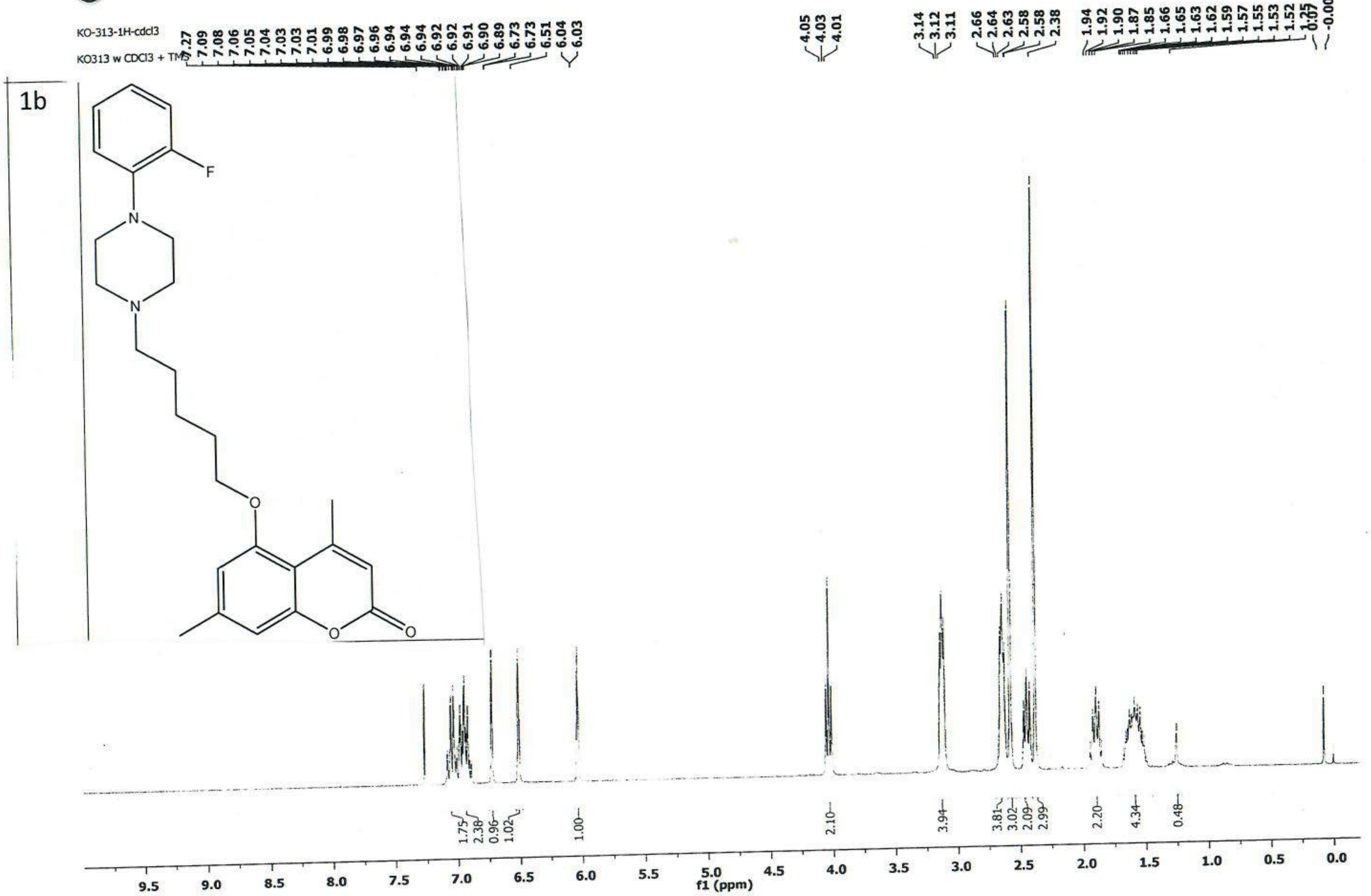
KO-313-1H-cdcl3
KO313 w CDCl3 + TMS
7.27 7.09 7.08 7.06 7.05 7.04 7.03 7.03 7.01 6.99 6.98 6.97 6.96 6.94 6.94 6.94 6.92 6.92 6.91 6.90 6.89 6.73 6.73 6.51 6.04 6.03
4.05 4.03 4.01
3.14 3.12 3.11
2.66 2.64 2.63 2.58 2.58 2.38
1.94 1.92 1.90 1.87 1.85 1.66 1.65 1.63 1.62 1.59 1.57 1.55 1.53 1.52 1.25 0.07 -0.00
1b
F
N
N
O
O
O
1.75 2.38 0.96 1.02 1.00 2.10 3.94 3.81 3.02 2.09 2.99 2.20 4.34 0.48
9.5 9.0 8.5 8.0 7.5 7.0 6.5 6.0 5.5 5.0 4.5 4.0 3.5 3.0 2.5 2.0 1.5 1.0 0.5 0.0
f1 (ppm)

16

16

1b

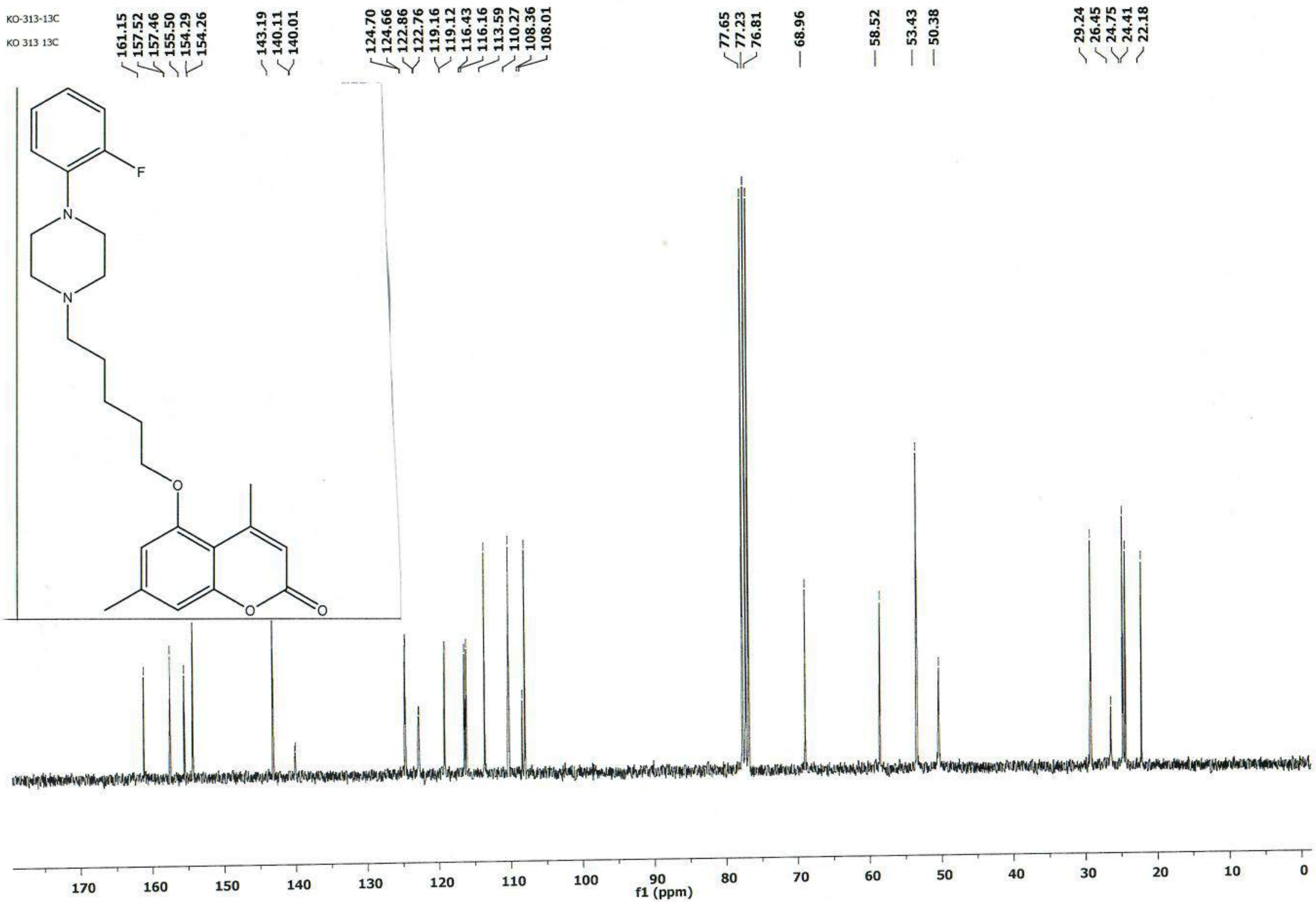
KO-313-13C
KO 313 13C
161.15
157.52
157.46
155.50
154.29
154.26
143.19
140.11
140.01
124.70
124.66
122.86
122.76
119.16
119.12
116.43
116.16
113.59
110.27
108.36
108.01
77.65
77.23
76.81
68.96
58.52
53.43
50.38
29.24
26.45
24.75
24.41
22.18
F
N
N
O
O
O
170
160
150
140
130
120
110
100
90
80
70
60
50
40
30
20
10
0
f1 (ppm)

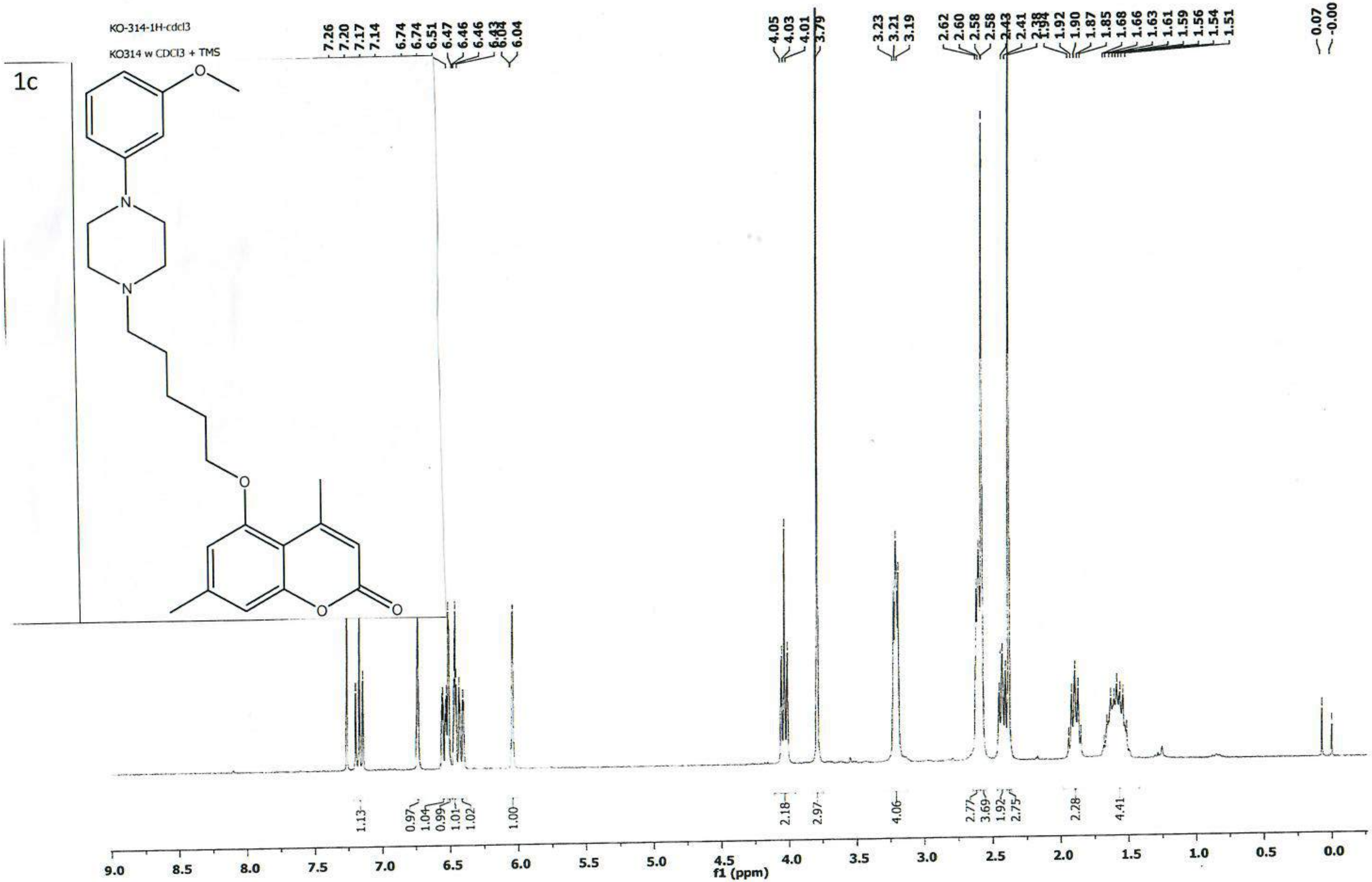
KO-314-1H-cdcl3
KO314 w CDCl3 + TMS
1c
7.26
7.20
7.17
7.14
6.74
6.74
6.51
6.47
6.46
6.46
6.04
4.05
4.03
4.01
3.79
3.23
3.21
3.19
2.62
2.60
2.58
2.58
2.43
2.41
1.92
1.90
1.87
1.85
1.68
1.66
1.63
1.61
1.59
1.56
1.54
1.51
0.07
-0.00
1.13
0.97
1.04
0.99
1.01
1.02
1.00
2.18
2.97
4.06
2.77
3.69
1.92
2.75
2.28
4.41
f1 (ppm)

1c

1c

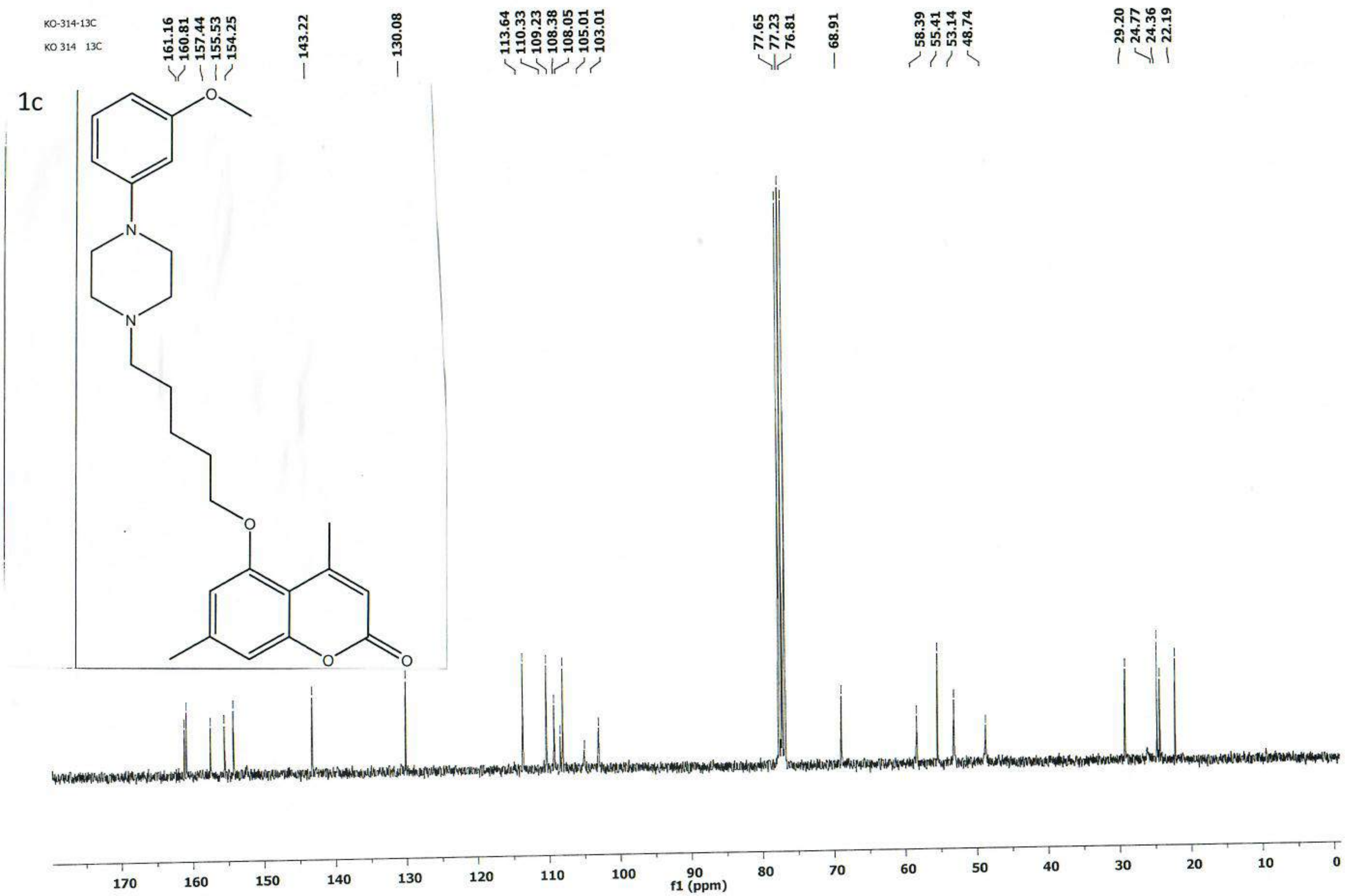
KO-314-13C
KO 314 13C
161.16
160.81
157.44
155.53
154.25
143.22
130.08
113.64
110.33
109.23
108.38
108.05
105.01
103.01
77.65
77.23
76.81
68.91
58.39
55.41
53.14
48.74
29.20
24.77
24.36
22.19
O
N
N
O
O
O
1c
170
160
150
140
130
120
110
100
90
f1 (ppm)
80
70
60
50
40
30
20
10
0

V

KO-315-1H-cdcl3

KO315 w CDCl3 + TMS

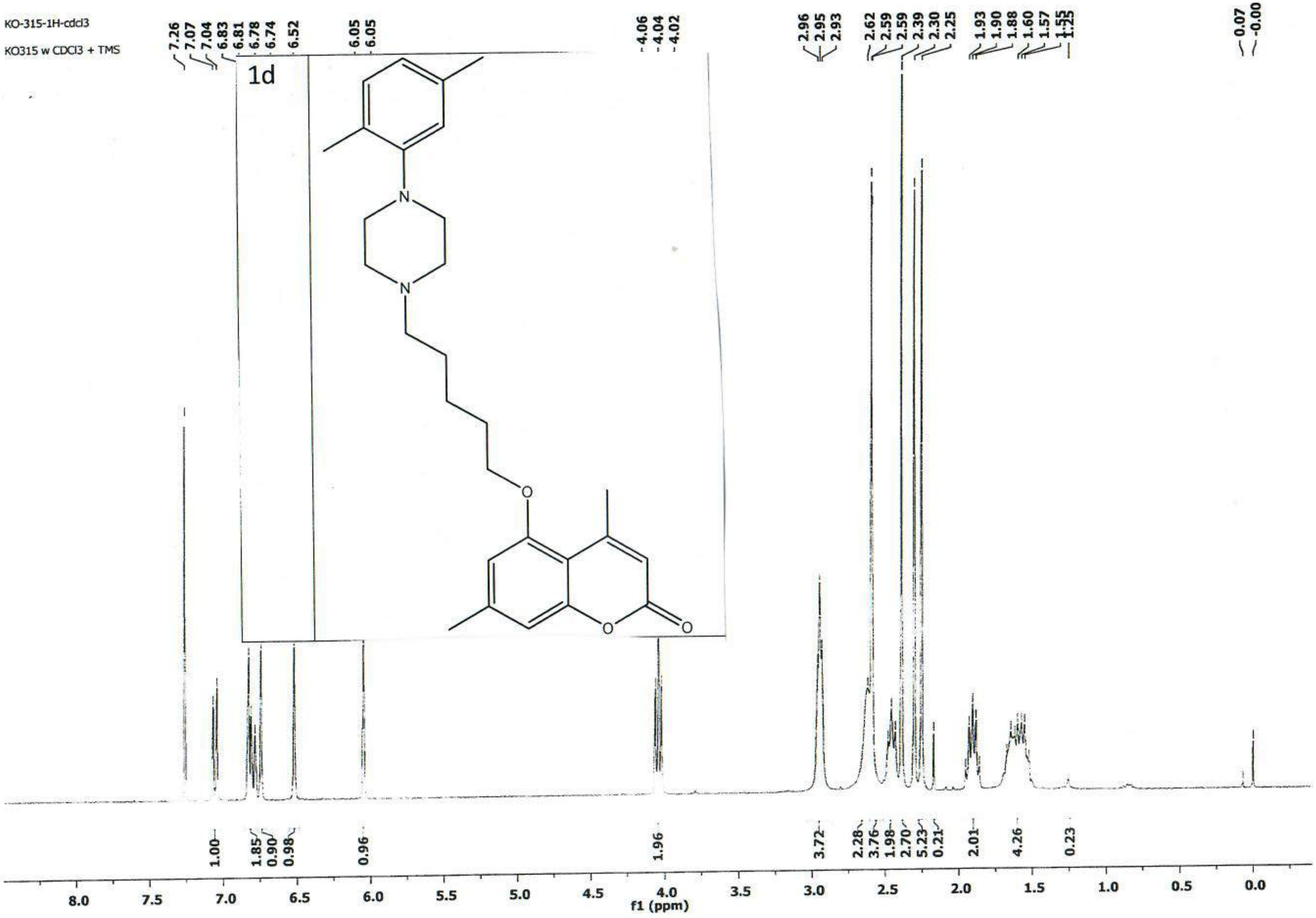

1d

1d

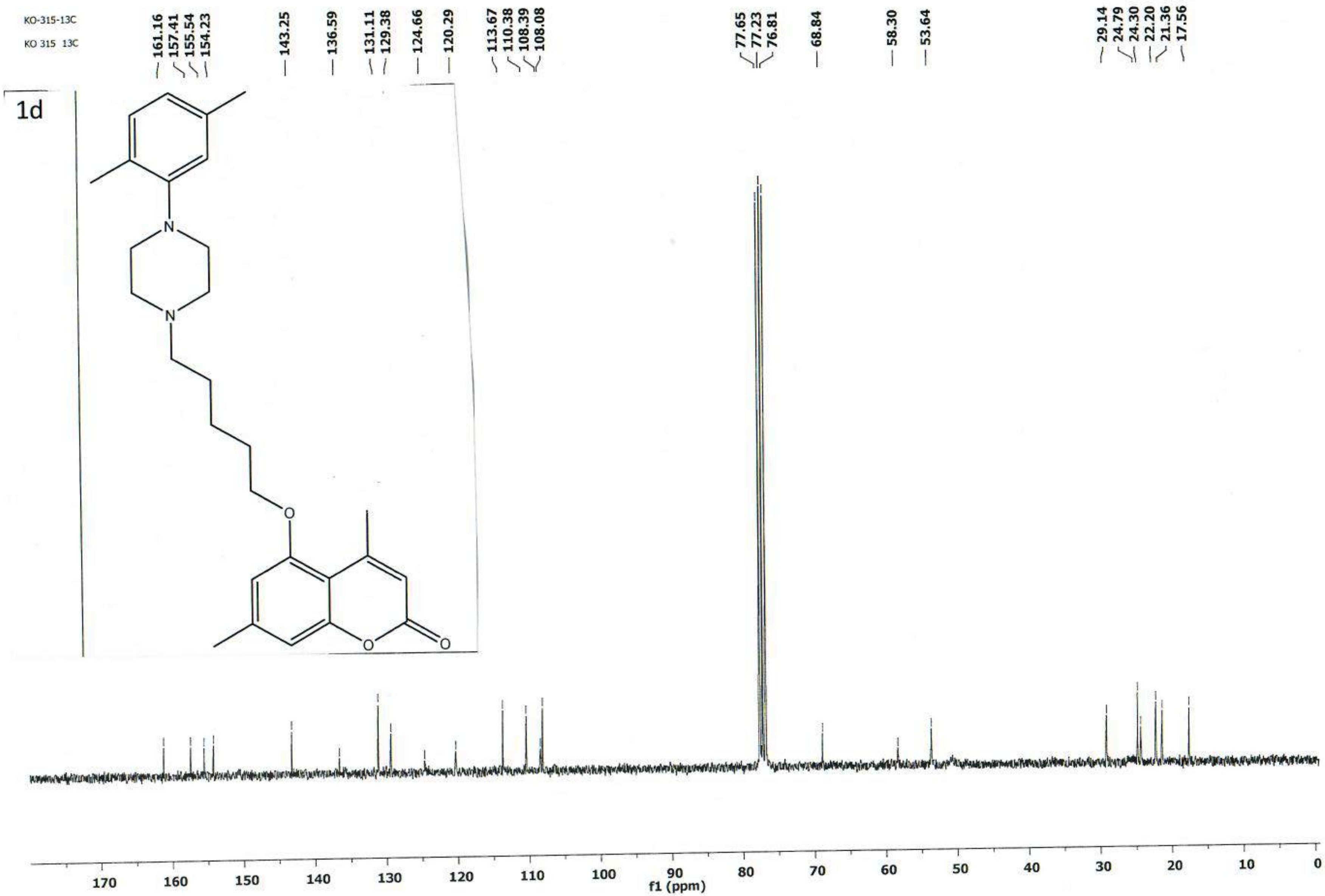
KO-315-13C
KO 315 13C
161.16
157.41
155.54
154.23
143.25
136.59
131.11
129.38
124.66
120.29
113.67
110.38
108.39
108.08
77.65
77.23
76.81
68.84
58.30
53.64
29.14
24.79
24.30
22.20
21.36
17.56
1d
N
N
O
O
O
170
160
150
140
130
120
110
100
90
f1 (ppm)
80
70
60
50
40
30
20
10
0

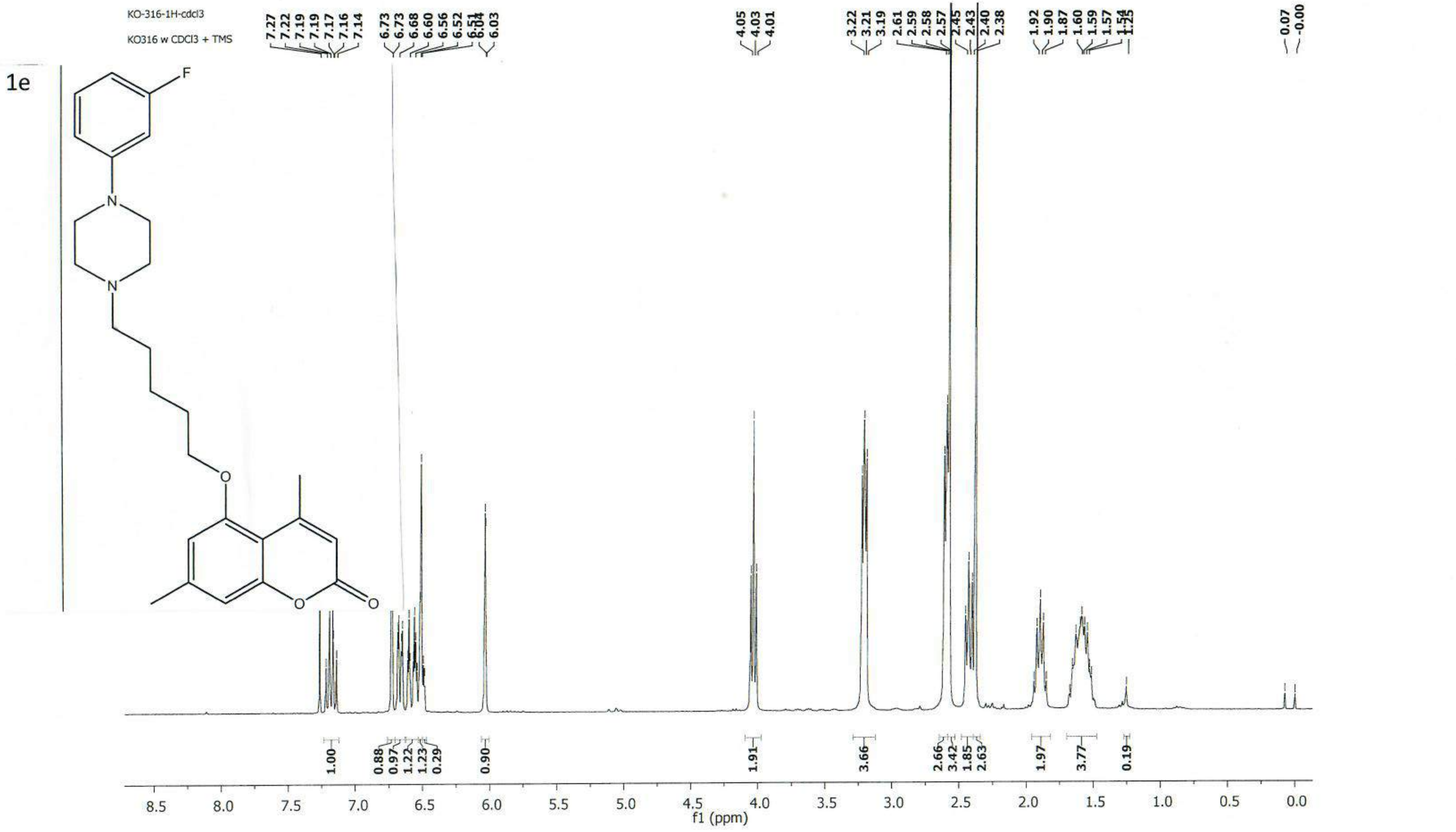
1e
KO-316-1H-cdcl3
KO316 w CDCl3 + TMS
7.27 7.22 7.19 7.19 7.17 7.16 7.14 6.73 6.73 6.68 6.60 6.56 6.52 6.51 6.04 6.03
4.05 4.03 4.01
3.22 3.21 3.19 2.61 2.59 2.58 2.57 2.45 2.43 2.40 2.38
1.92 1.90 1.87 1.60 1.59 1.57 1.54 1.25
0.07 -0.00
1.00 0.88 0.97 1.22 1.23 0.29 0.90 1.91 3.66 2.66 3.42 1.85 2.63 1.97 3.77 0.19
8.5 8.0 7.5 7.0 6.5 6.0 5.5 5.0 4.5 4.0 3.5 3.0 2.5 2.0 1.5 1.0 0.5 0.0
f1 (ppm)
1e

1e

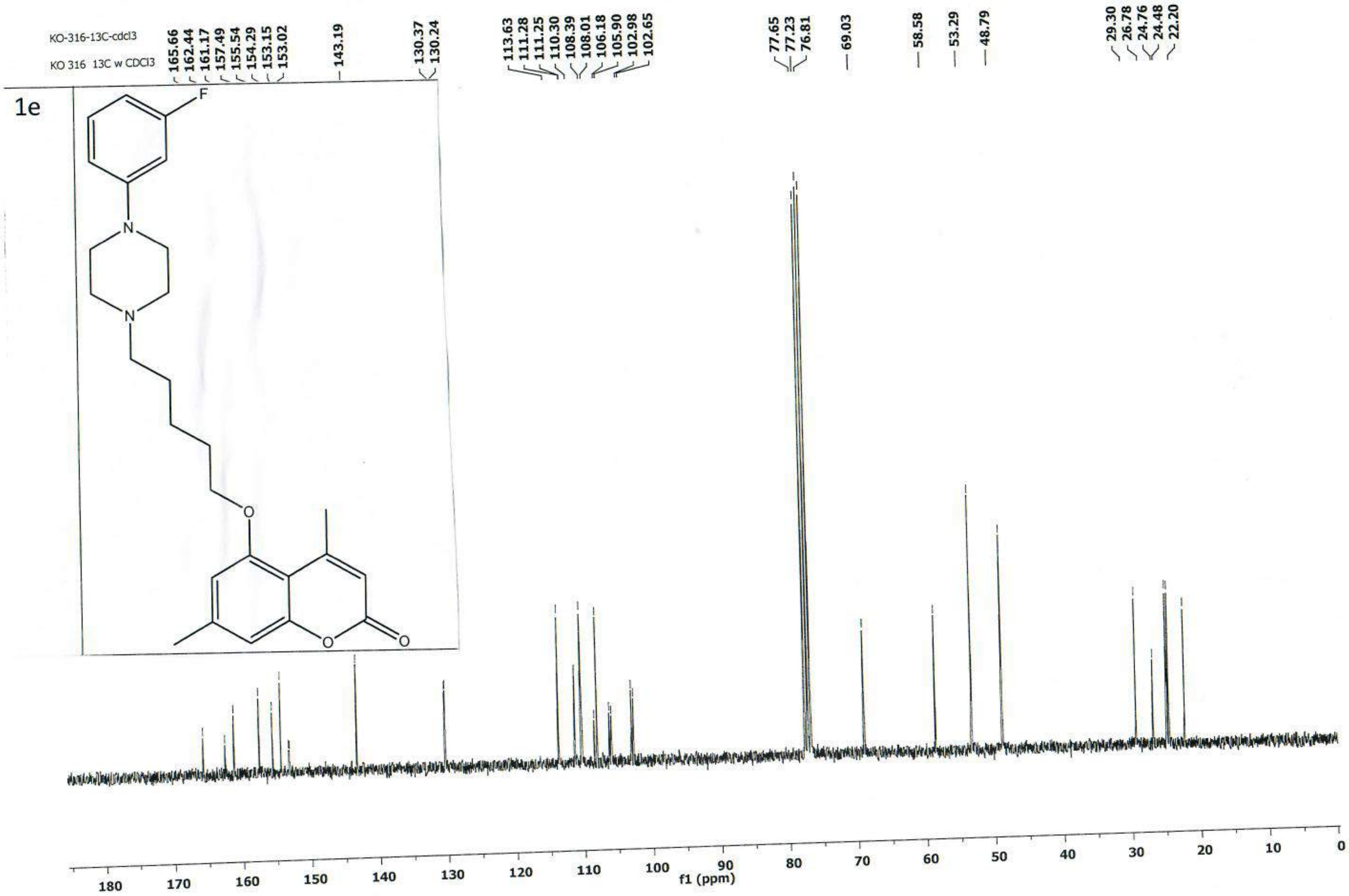
KO-316-13C-cdcl3
KO 316 13C w CDCl3
165.66
162.44
161.17
157.49
155.54
154.29
153.15
153.02
143.19
130.37
130.24
113.63
111.28
111.25
110.30
108.39
108.01
106.18
105.90
102.98
102.65
77.65
77.23
76.81
69.03
58.58
53.29
48.79
29.30
26.78
24.76
24.48
22.20
1e
F
N
N
O
O
O
180
170
160
150
140
130
120
110
100
90
f1 (ppm)
80
70
60
50
40
30
20
10
0

1f

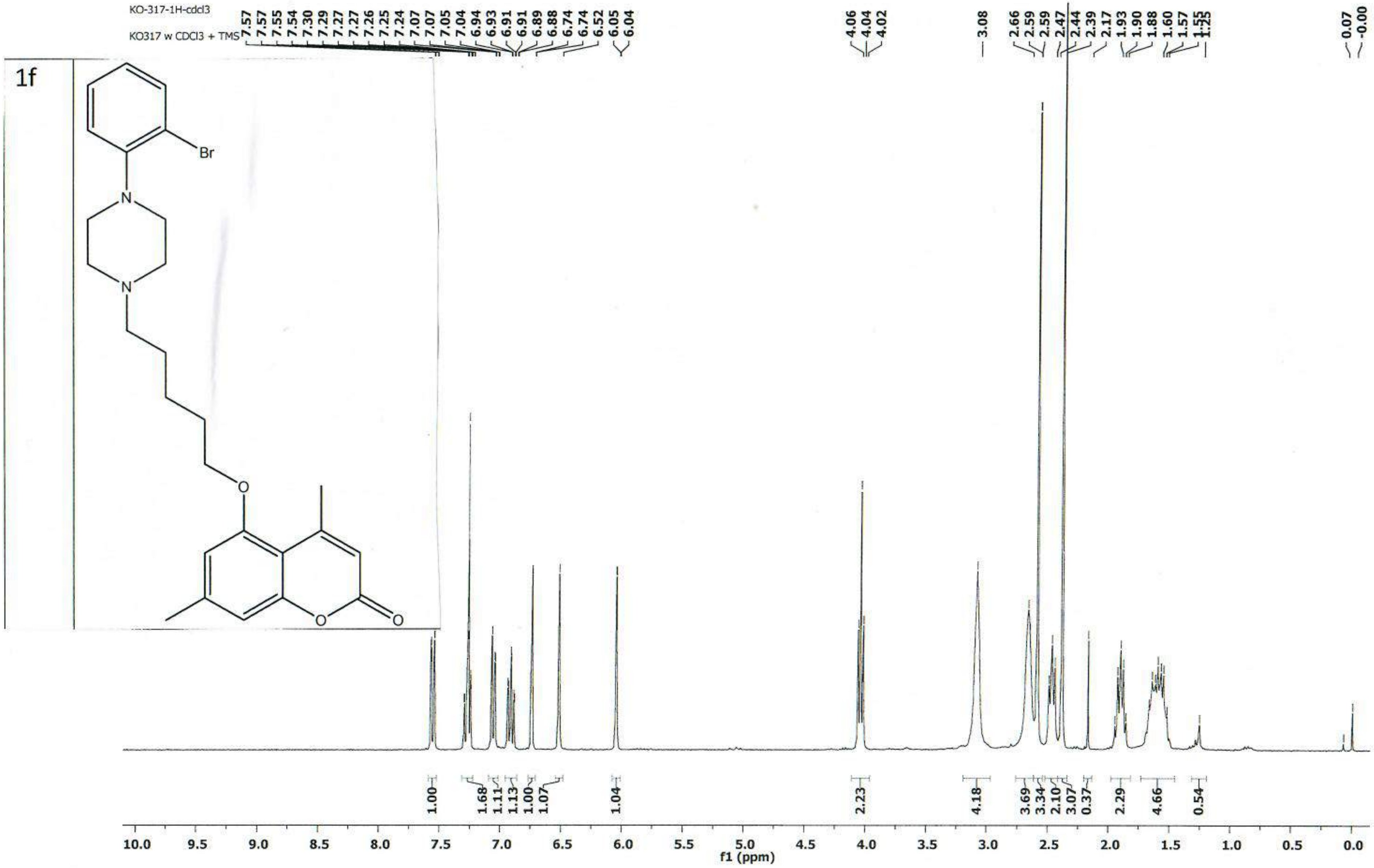
KO-317-1H-cdcl3
KO317 w CDCl3 + TMS
7.57 7.57 7.55 7.54 7.30 7.29 7.27 7.27 7.26 7.25 7.24 7.07 7.07 7.05 7.04 6.94 6.93 6.91 6.91 6.89 6.88 6.74 6.74 6.52 6.05 6.04
4.06 4.04 4.02
3.08
2.66 2.59 2.59 2.47 2.44 2.39 2.17 1.93 1.90 1.88 1.60 1.57 1.55 1.25
0.07 -0.00
1f
Br
N
N
O
O
O
1.00 1.68 1.11 1.13 1.00 1.07 1.04 2.23 4.18 3.69 3.34 2.10 3.07 0.37 2.29 4.66 0.54
10.0 9.5 9.0 8.5 8.0 7.5 7.0 6.5 6.0 5.5 5.0 4.5 4.0 3.5 3.0 2.5 2.0 1.5 1.0 0.5 0.0
f1 (ppm)

KO-317-13C-cdcl3

KO 317 13C w CDCl3

1f

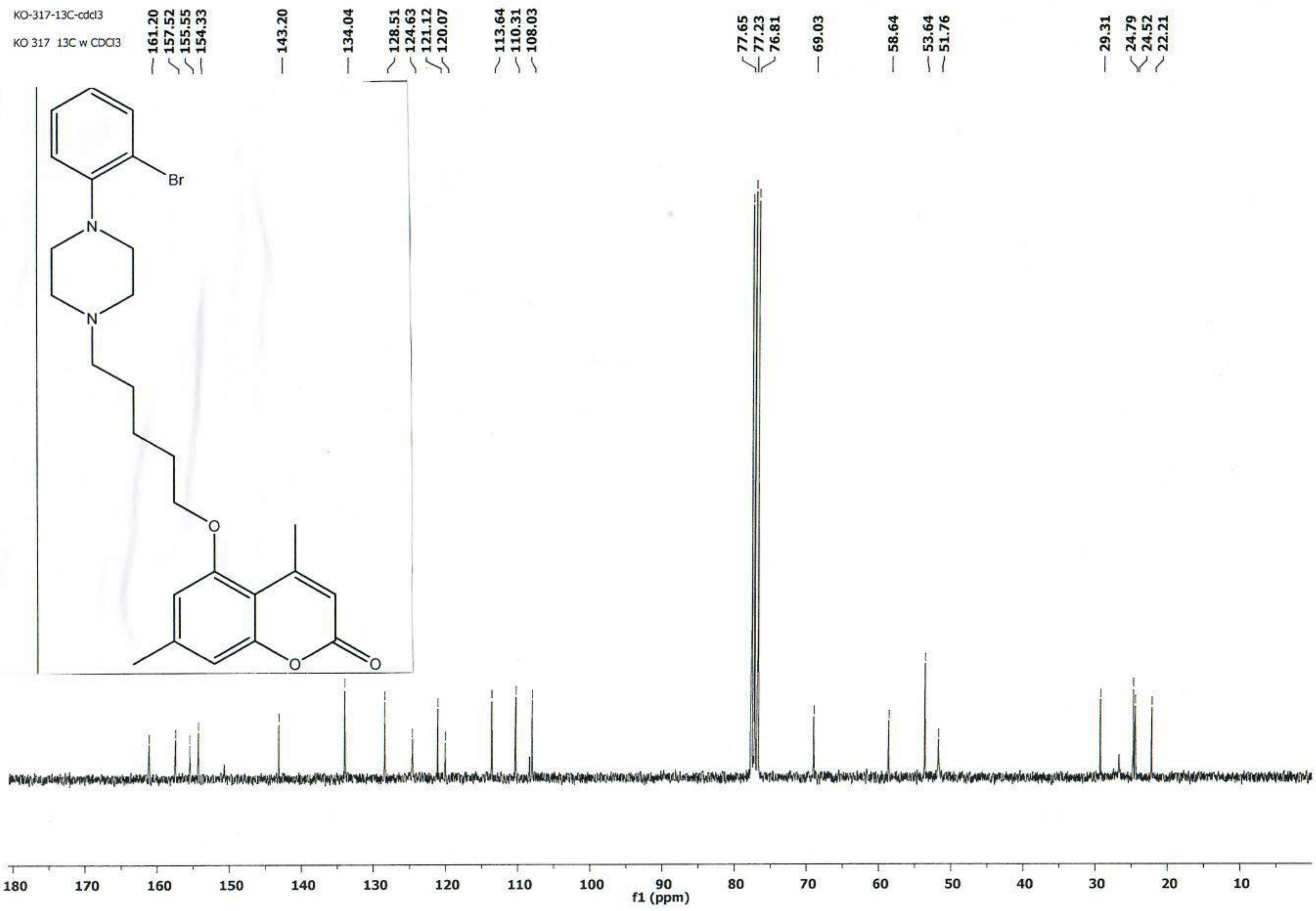

1f

1g

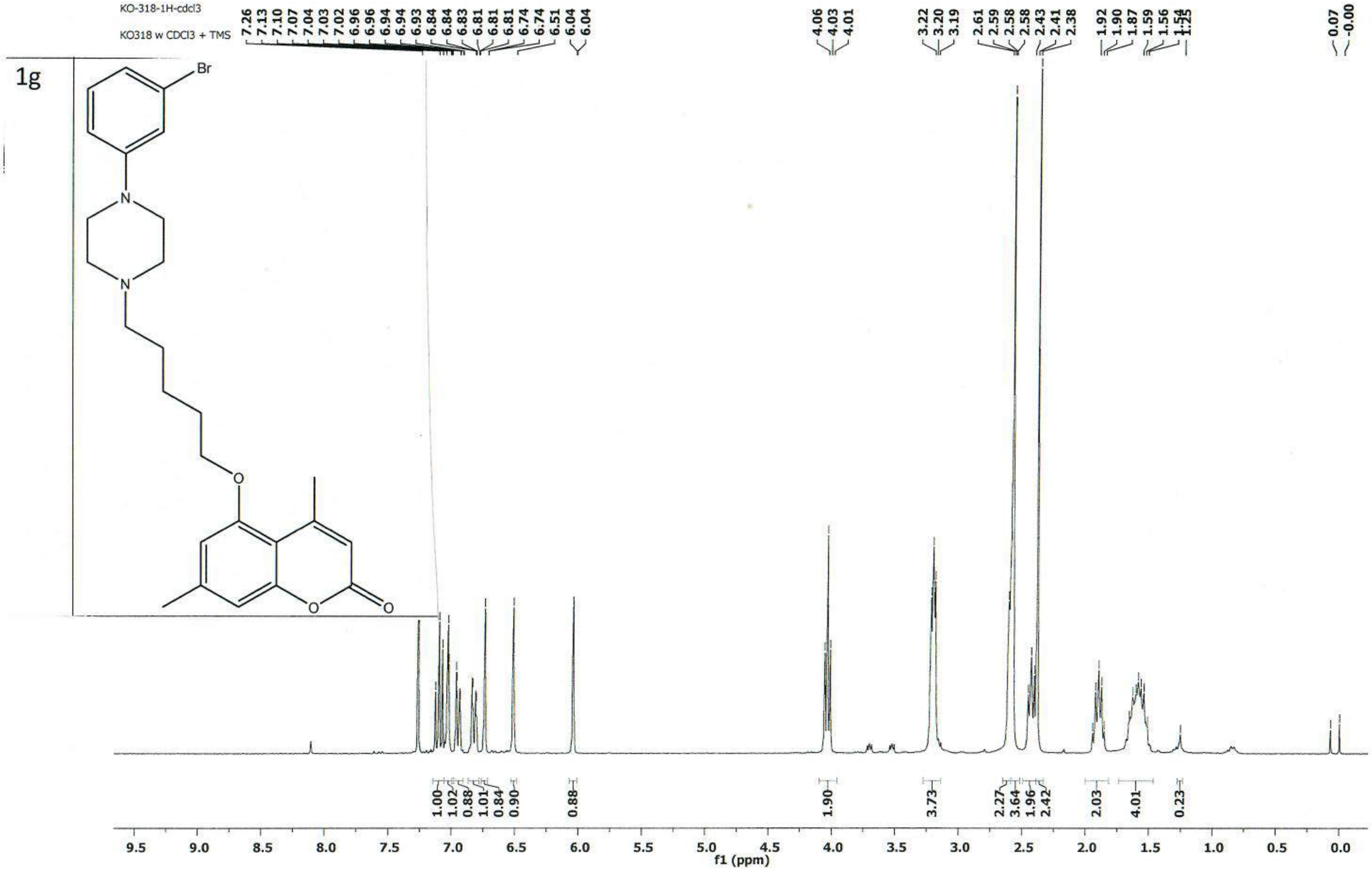

KO-318-1H-cdcl3
KO318 w CDCl3 + TMS
7.26 7.13 7.10 7.07 7.04 7.03 7.02 6.96 6.96 6.94 6.94 6.93 6.84 6.84 6.83 6.81 6.81 6.81 6.74 6.74 6.51 6.04 6.04
4.06 4.03 4.01
3.22 3.20 3.19
2.61 2.59 2.58 2.58 2.43 2.41 2.38
1.92 1.90 1.87 1.59 1.56 1.54 1.25
0.07 -0.00
1.00 1.02 0.88 1.01 0.84 0.90 0.88 1.90 3.73 2.27 3.64 1.96 2.42 2.03 4.01 0.23
9.5 9.0 8.5 8.0 7.5 7.0 6.5 6.0 5.5 5.0 4.5 4.0 3.5 3.0 2.5 2.0 1.5 1.0 0.5 0.0
f1 (ppm)

1g

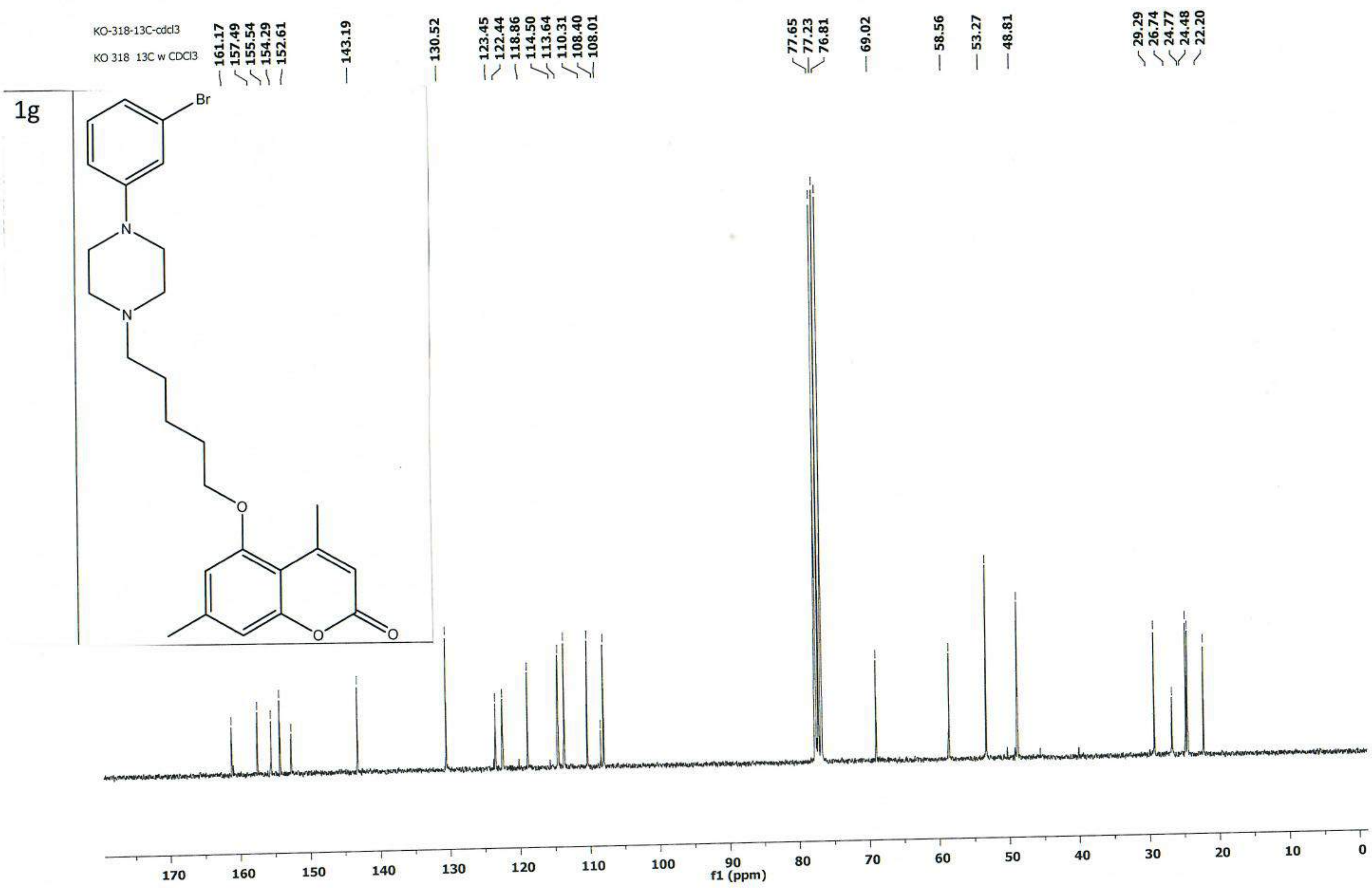

KO-318-13C-cdcl3
KO 318 13C w CDCl3
161.17
157.49
155.54
154.29
152.61
143.19
130.52
123.45
122.44
118.86
114.50
113.64
110.31
108.40
108.01
77.65
77.23
76.81
69.02
58.56
53.27
48.81
29.29
26.74
24.77
24.48
22.20
1g
Br
N
N
O
O
O
170
160
150
140
130
120
110
100
90
f1 (ppm)
80
70
60
50
40
30
20
10
0

1h

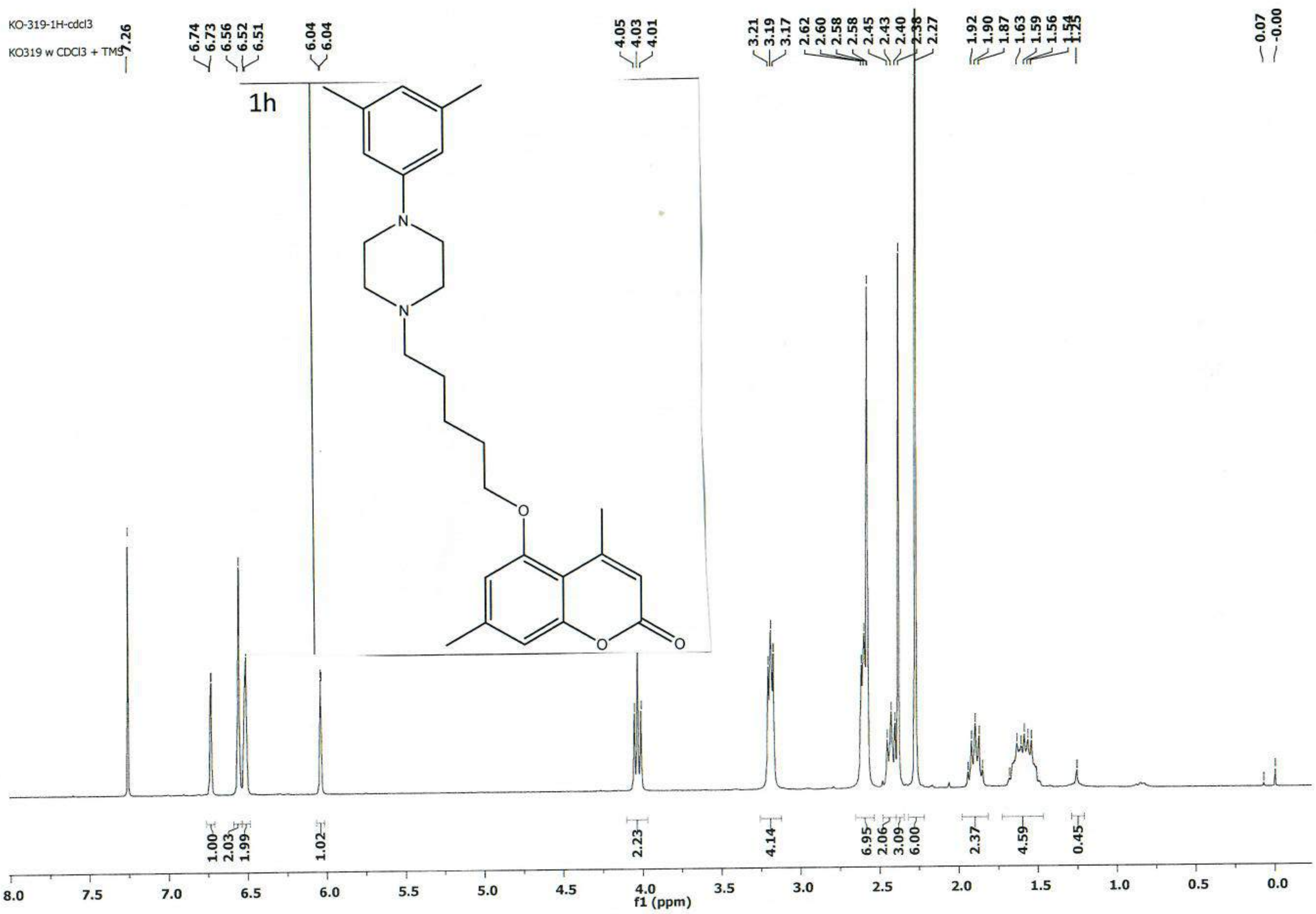
KO-319-1H-cdcl3
KO319 w CDCl3 + TMS
7.26
6.74 6.73 6.56 6.52 6.51
6.04 6.04
4.05 4.03 4.01
3.21 3.19 3.17 2.62 2.60 2.58 2.58 2.45 2.43 2.40 2.38 2.27
1.92 1.90 1.87 1.63 1.59 1.56 1.54 1.25
0.07 -0.00
1h
N
N
O
O
O
1.00 2.03 1.99 1.02 2.23 4.14 6.95 2.06 3.09 6.00 2.37 4.59 0.45
8.0 7.5 7.0 6.5 6.0 5.5 5.0 4.5 4.0 3.5 3.0 2.5 2.0 1.5 1.0 0.5 0.0
f1 (ppm)

1h

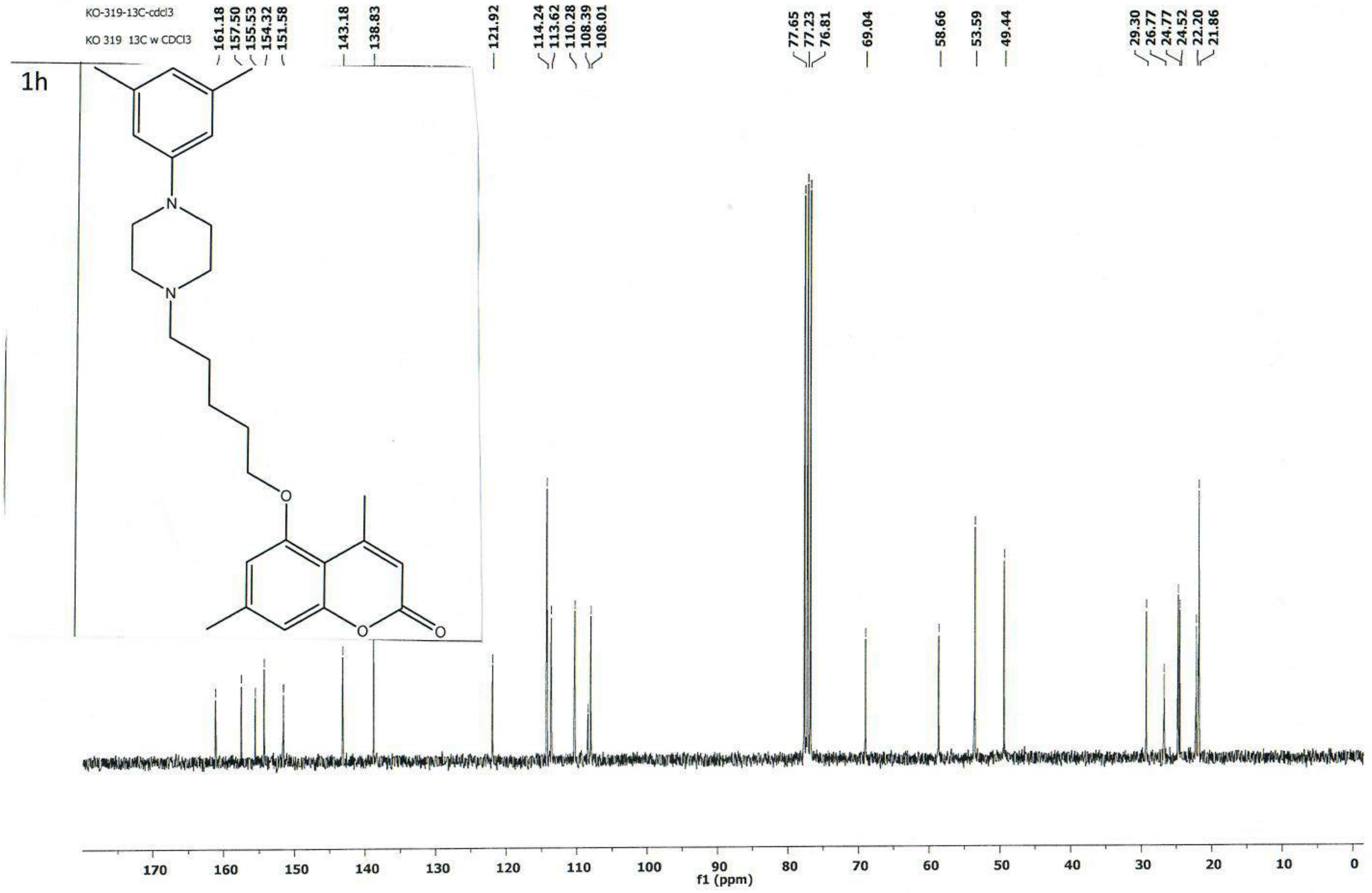
KO-319-13C-cdcl3
KO 319 13C w CDCl3
1h
161.18
157.50
155.53
154.32
151.58
143.18
138.83
121.92
114.24
113.62
110.28
108.39
108.01
77.65
77.23
76.81
69.04
58.66
53.59
49.44
29.30
26.77
24.77
24.52
22.20
21.86
170 160 150 140 130 120 110 100 90 80 70 60 50 40 30 20 10 0
f1 (ppm)

1i

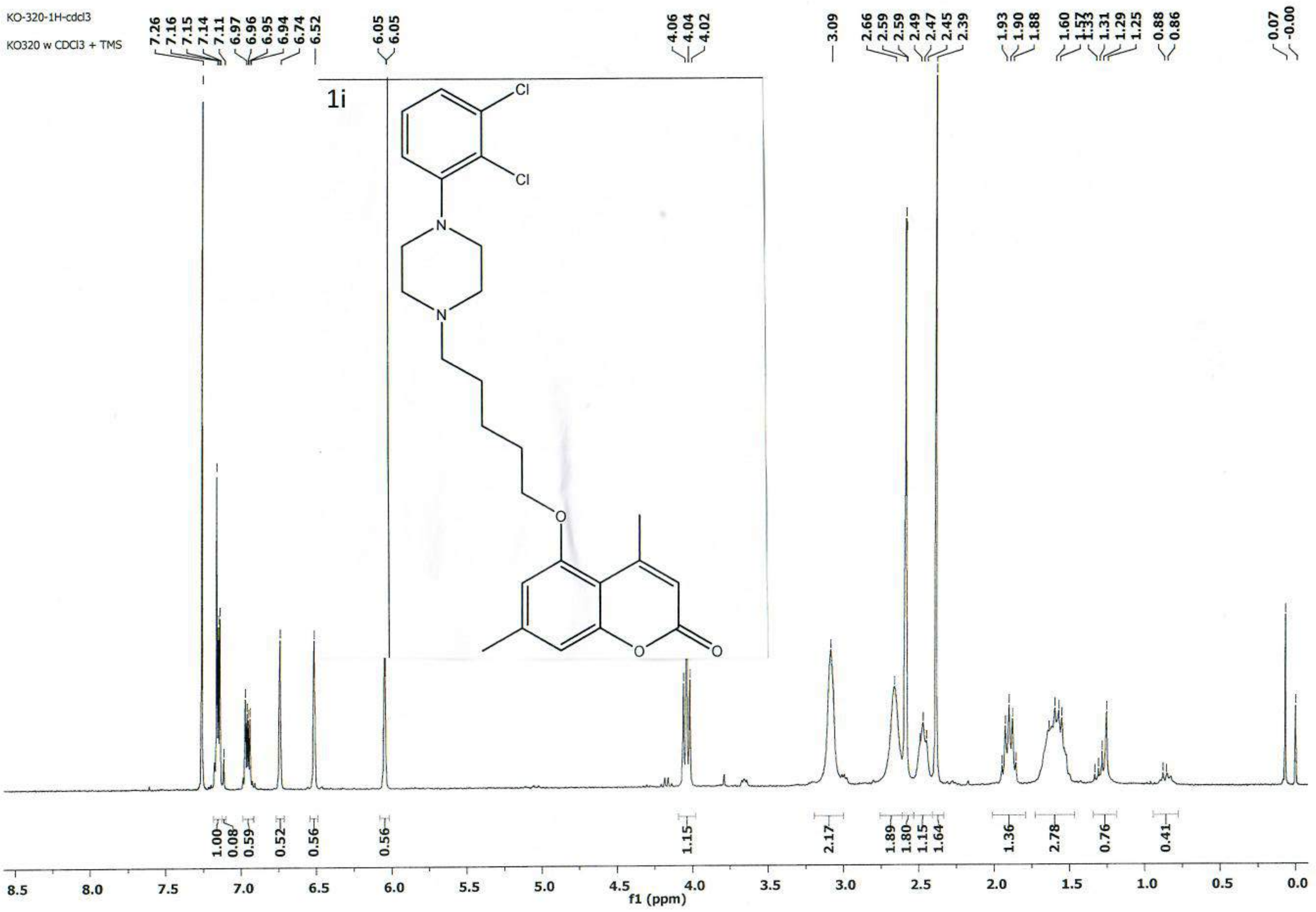

KO-320-1H-cdcl3
KO320 w CDCl3 + TMS
7.26 7.16 7.15 7.14 7.11 6.97 6.96 6.95 6.94 6.74 6.52
6.05 6.05
4.06 4.04 4.02
3.09
2.66 2.59 2.59 2.49 2.47 2.45 2.39
1.93 1.90 1.88
1.60 1.57 1.33 1.31 1.29 1.25
0.88 0.86
0.07 -0.00
1i
Cl
Cl
N
N
O
O
O
1.00 0.08 0.59 0.52 0.56 0.56 1.15 2.17 1.89 1.80 1.15 1.64 1.36 2.78 0.76 0.41
8.5 8.0 7.5 7.0 6.5 6.0 5.5 5.0 4.5 4.0 3.5 3.0 2.5 2.0 1.5 1.0 0.5 0.0
f1 (ppm)

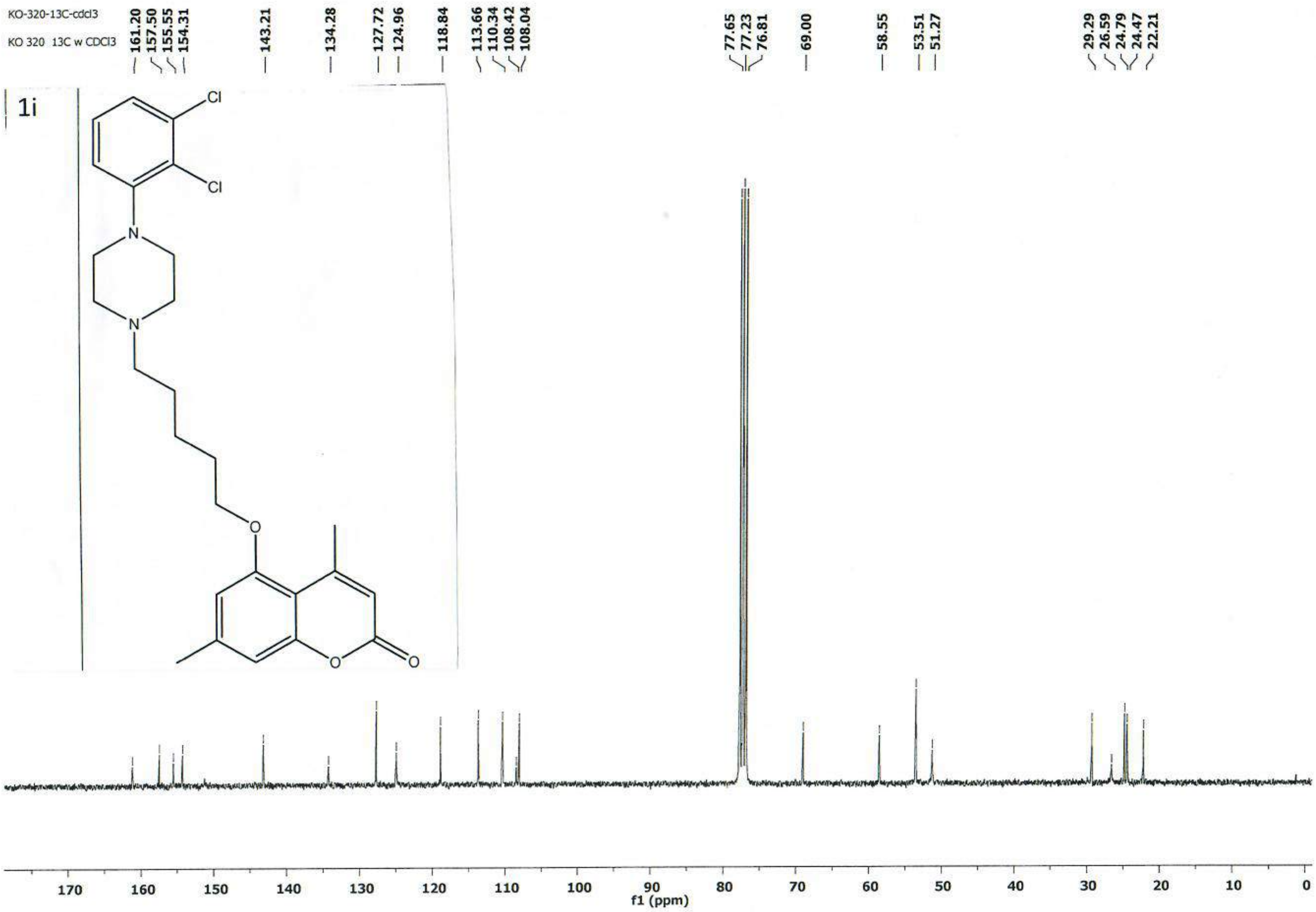
KO-320-13C-cdcl3
KO 320 13C w CDCl3
161.20
157.50
155.55
154.31
143.21
134.28
127.72
124.96
118.84
113.66
110.34
108.42
108.04
77.65
77.23
76.81
69.00
58.55
53.51
51.27
29.29
26.59
24.79
24.47
22.21
1i
Cl
Cl
N
N
O
O
O
170
160
150
140
130
120
110
100
90
80
70
60
50
40
30
20
10
0
f1 (ppm)

1i

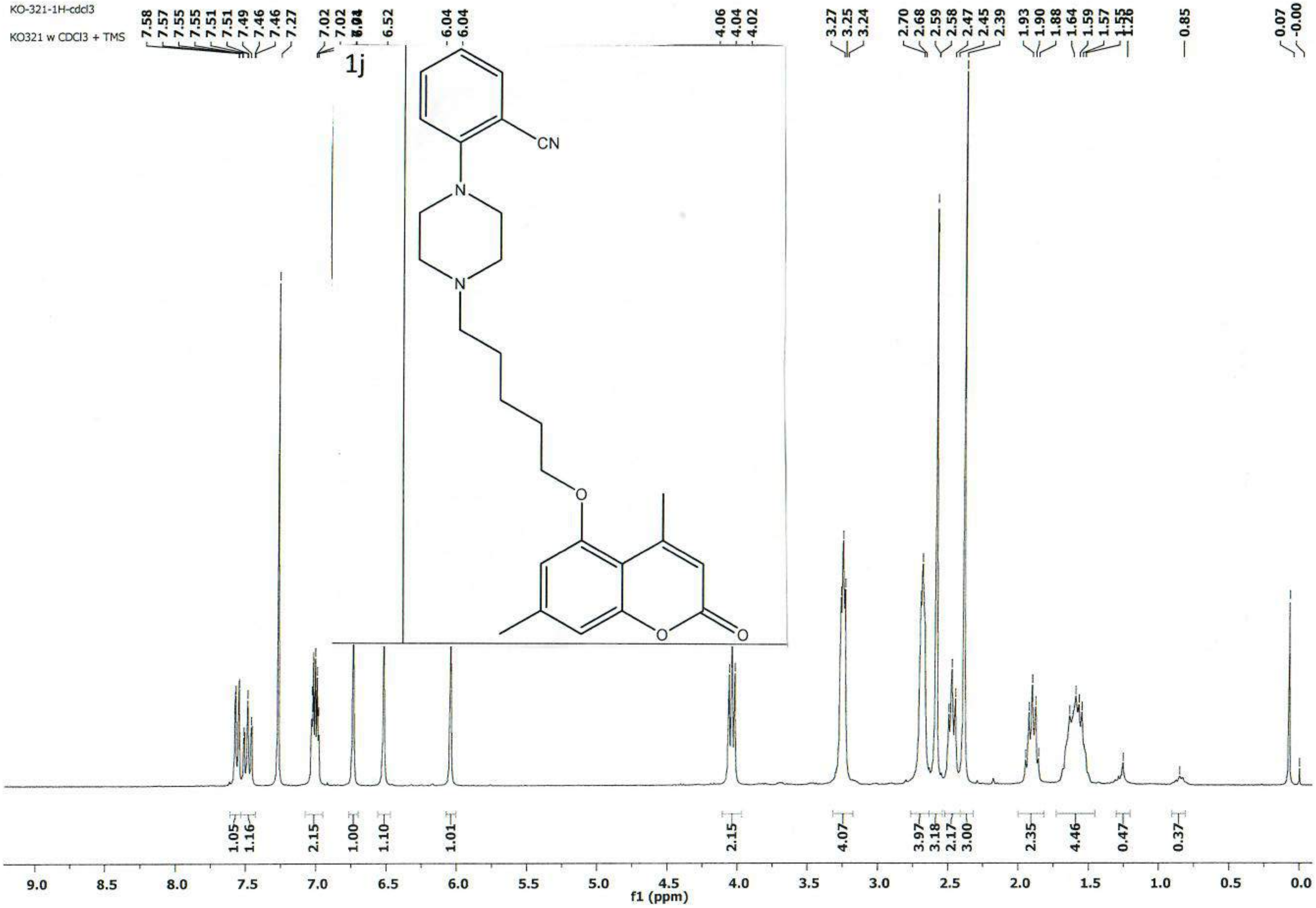
KO-321-1H-cdcl3
KO321 w CDCl3 + TMS
7.58 7.57 7.55 7.55 7.51 7.51 7.49 7.46 7.46 7.27
7.02 7.02
6.52
6.04 6.04
4.06 4.04 4.02
3.27 3.25 3.24
2.70 2.68 2.59 2.58 2.47 2.45 2.39
1.93 1.90 1.88 1.64 1.59 1.57
0.85
0.07 -0.00
1j
CN
N
N
O
O
O
1.05 1.16 2.15 1.00 1.10 1.01 2.15 4.07 3.97 3.18 2.17 3.00 2.35 4.46 0.47 0.37
9.0 8.5 8.0 7.5 7.0 6.5 6.0 5.5 5.0 4.5 4.0 3.5 3.0 2.5 2.0 1.5 1.0 0.5 0.0
f1 (ppm)

1j

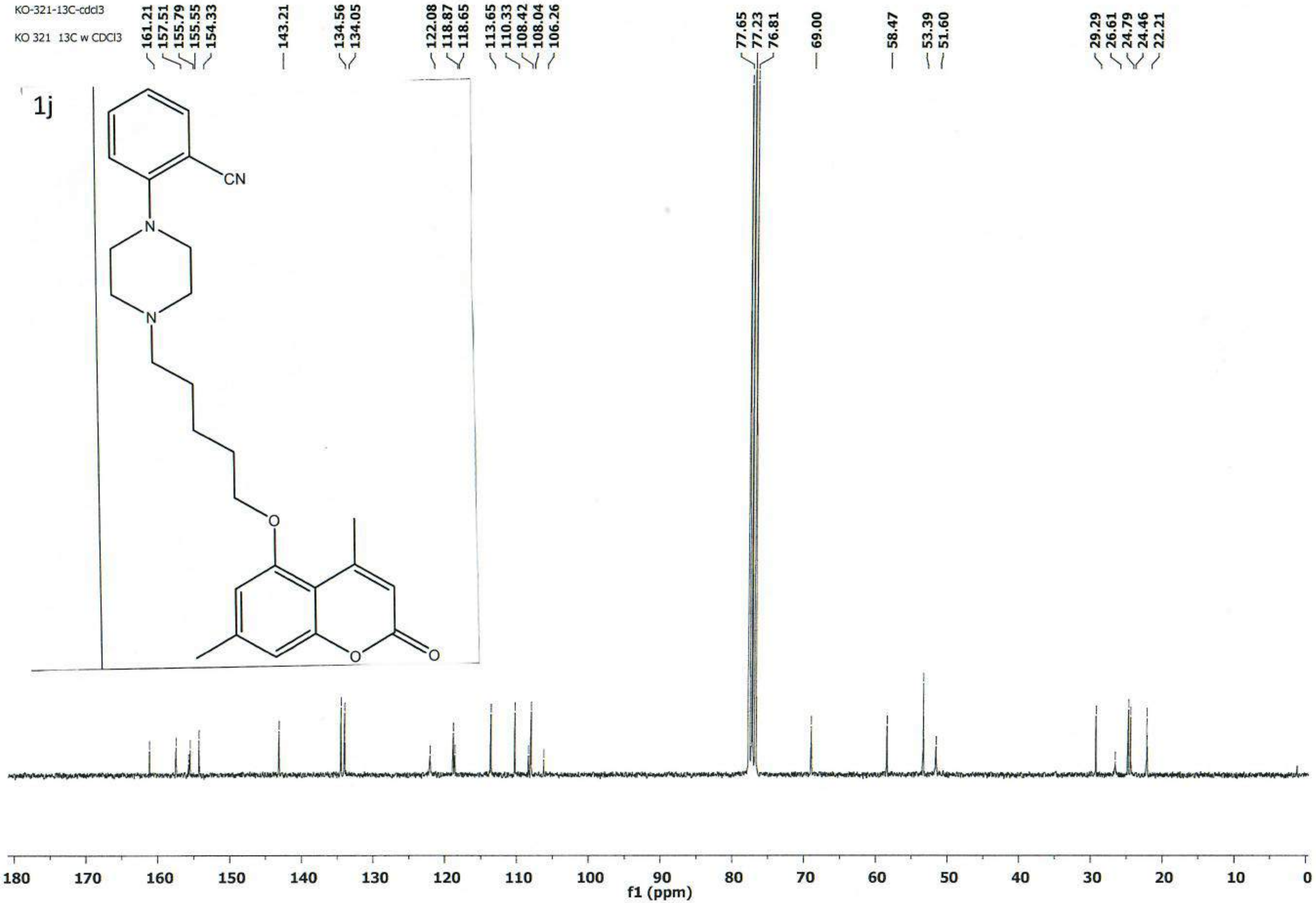
KO-321-13C-cdcl3
KO 321 13C w CDCl3
161.21
157.51
155.79
155.55
154.33
143.21
134.56
134.05
122.08
118.87
118.65
113.65
110.33
108.42
108.04
106.26
77.65
77.23
76.81
69.00
58.47
53.39
51.60
29.29
26.61
24.79
24.46
22.21
1j
CN
N
N
O
O
O
180 170 160 150 140 130 120 110 100 90 80 70 60 50 40 30 20 10 0
f1 (ppm)

KO-333-1H

KO 333 1H w CDCl3

2

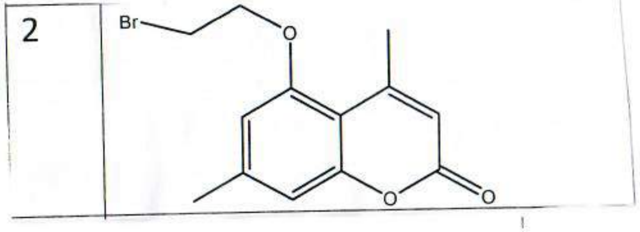

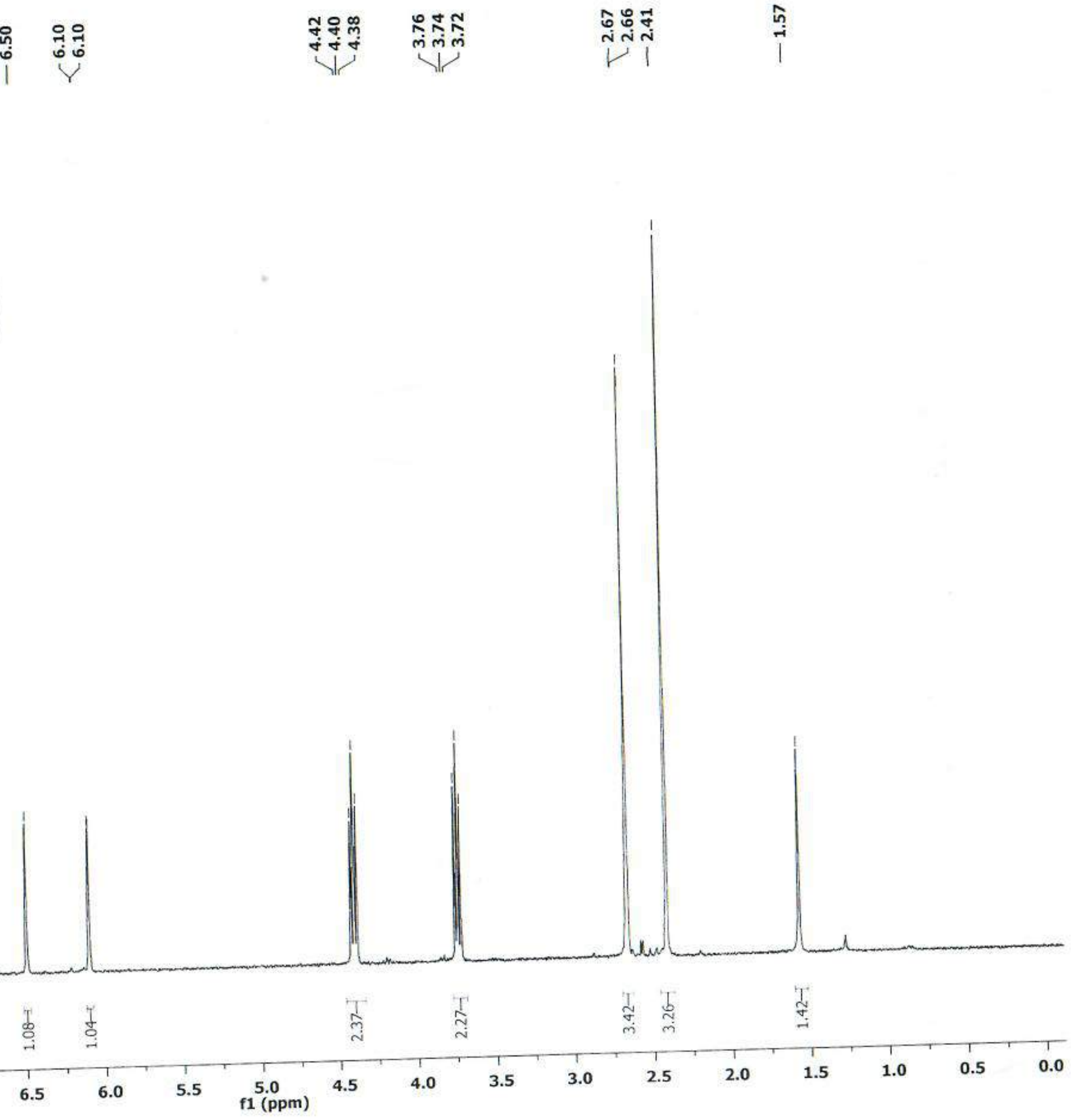

2

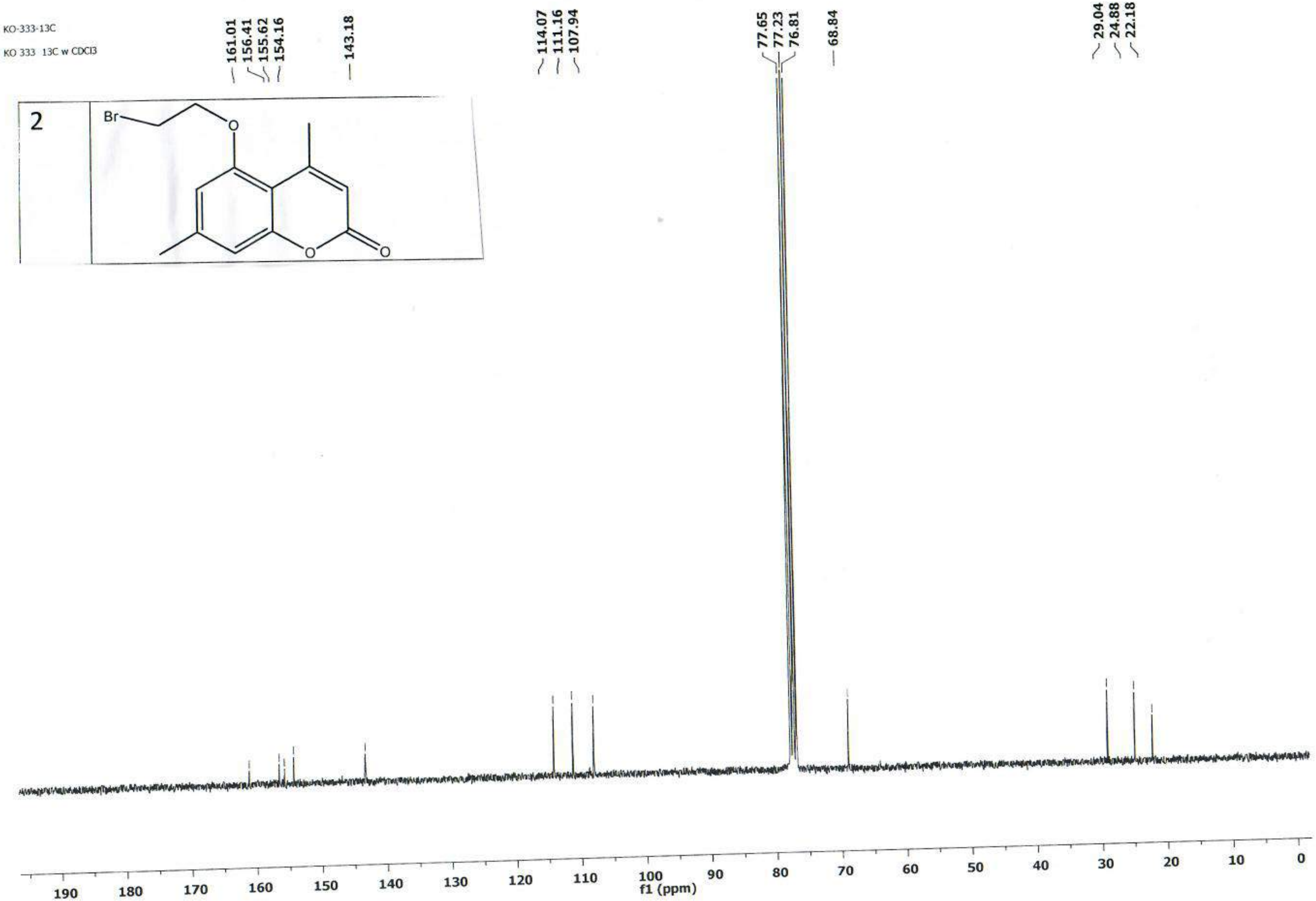
KO-333-13C
KO 333 13C w CDCl3
161.01
156.41
155.62
154.16
143.18
114.07
111.16
107.94
77.65
77.23
76.81
68.84
29.04
24.88
22.18
2
Br
O
O
O
190 180 170 160 150 140 130 120 110 100 90 80 70 60 50 40 30 20 10 0
f1 (ppm)

2e

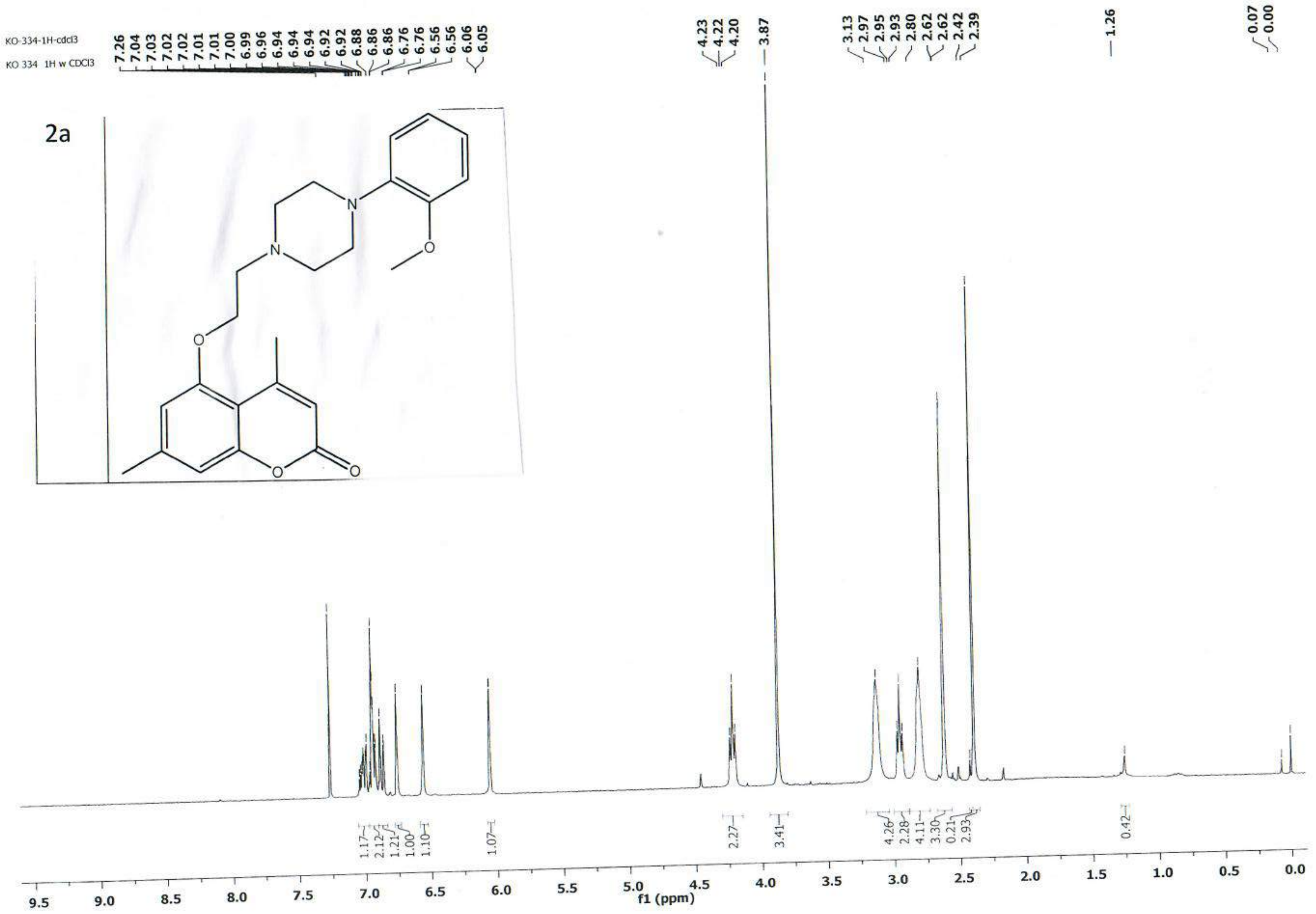
KO-334-1H-cdcl3
KO 334 1H w CDCl3
7.26
7.04
7.03
7.02
7.02
7.01
7.01
7.00
6.99
6.96
6.94
6.94
6.94
6.92
6.92
6.88
6.86
6.86
6.76
6.76
6.56
6.56
6.06
6.05
4.23
4.22
4.20
3.87
3.13
2.97
2.95
2.93
2.80
2.62
2.62
2.42
2.39
1.26
0.07
0.00
2a
N
N
O
O
O
O
1.17
2.12
1.21
1.00
1.10
1.07
2.27
3.41
4.26
2.28
4.11
3.30
0.21
2.93
0.42
9.5
9.0
8.5
8.0
7.5
7.0
6.5
6.0
5.5
5.0
4.5
4.0
3.5
3.0
2.5
2.0
1.5
1.0
0.5
0.0
f1 (ppm)

2a

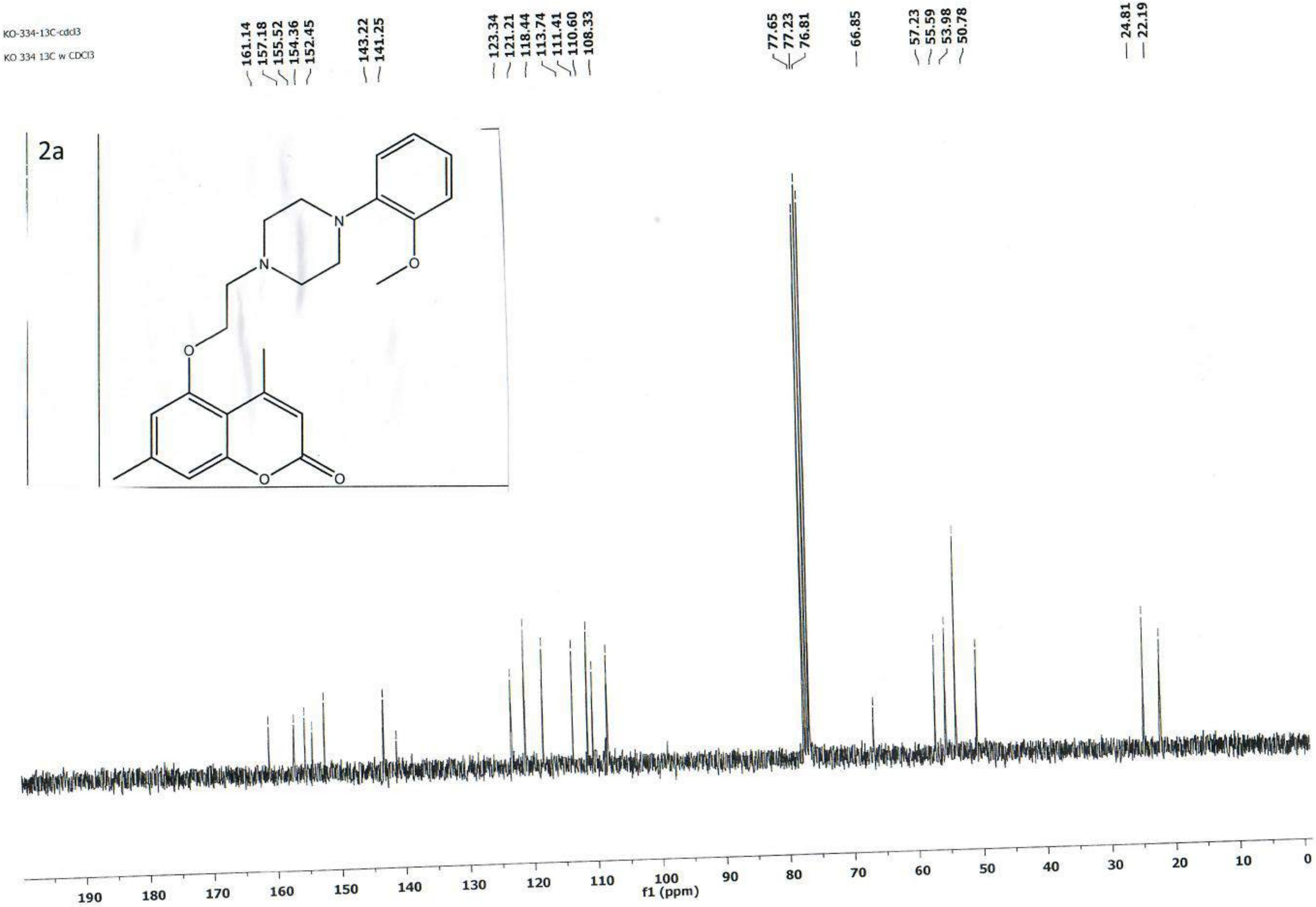
KO-334-13C-cdcl3
KO 334 13C w CDCl3
161.14
157.18
155.52
154.36
152.45
143.22
141.25
123.34
121.21
118.44
113.74
111.41
110.60
108.33
77.65
77.23
76.81
66.85
57.23
55.59
53.98
50.78
24.81
22.19
2a
N
N
O
O
O
O
190 180 170 160 150 140 130 120 110 100 90 80 70 60 50 40 30 20 10 0
f1 (ppm)

2b

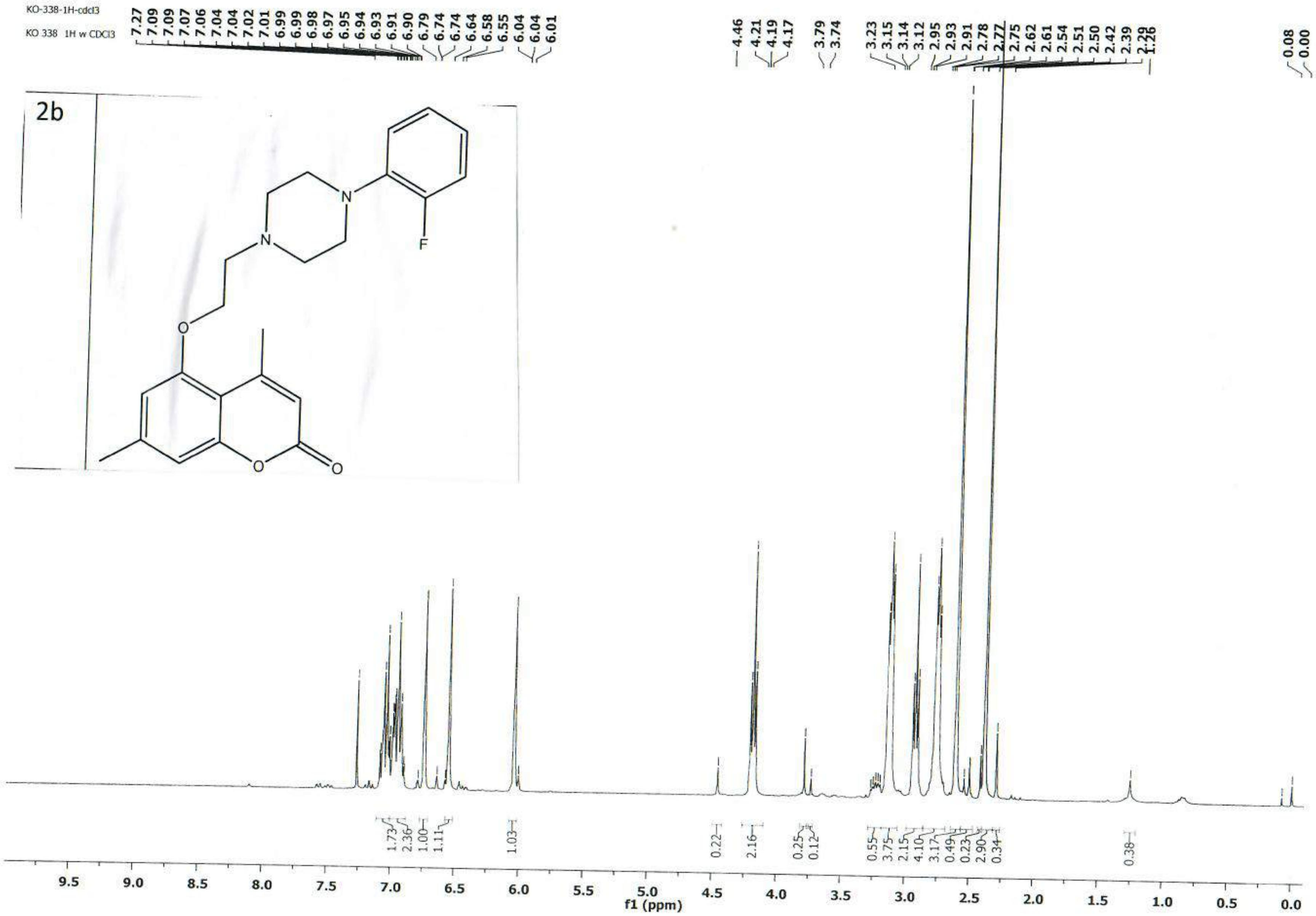
KO-338-1H-cdcl3
KO 338 1H w CDCl3
7.27 7.09 7.09 7.07 7.06 7.04 7.04 7.02 7.01 6.99 6.99 6.98 6.97 6.95 6.94 6.93 6.91 6.90 6.79 6.74 6.74 6.64 6.58 6.55 6.04 6.04 6.01
4.46 4.21 4.19 4.17 3.79 3.74 3.23 3.15 3.14 3.12 2.95 2.93 2.91 2.78 2.77 2.75 2.62 2.61 2.54 2.51 2.50 2.42 2.39 2.29 1.26
0.08 0.00
2b
N
N
F
O
O
O
1.73 2.36 1.00 1.11 1.03 0.22 2.16 0.25 0.12 0.55 3.75 2.15 4.10 3.17 0.49 0.23 2.90 0.34 0.38
9.5 9.0 8.5 8.0 7.5 7.0 6.5 6.0 5.5 5.0 4.5 4.0 3.5 3.0 2.5 2.0 1.5 1.0 0.5 0.0
f1 (ppm)

2b

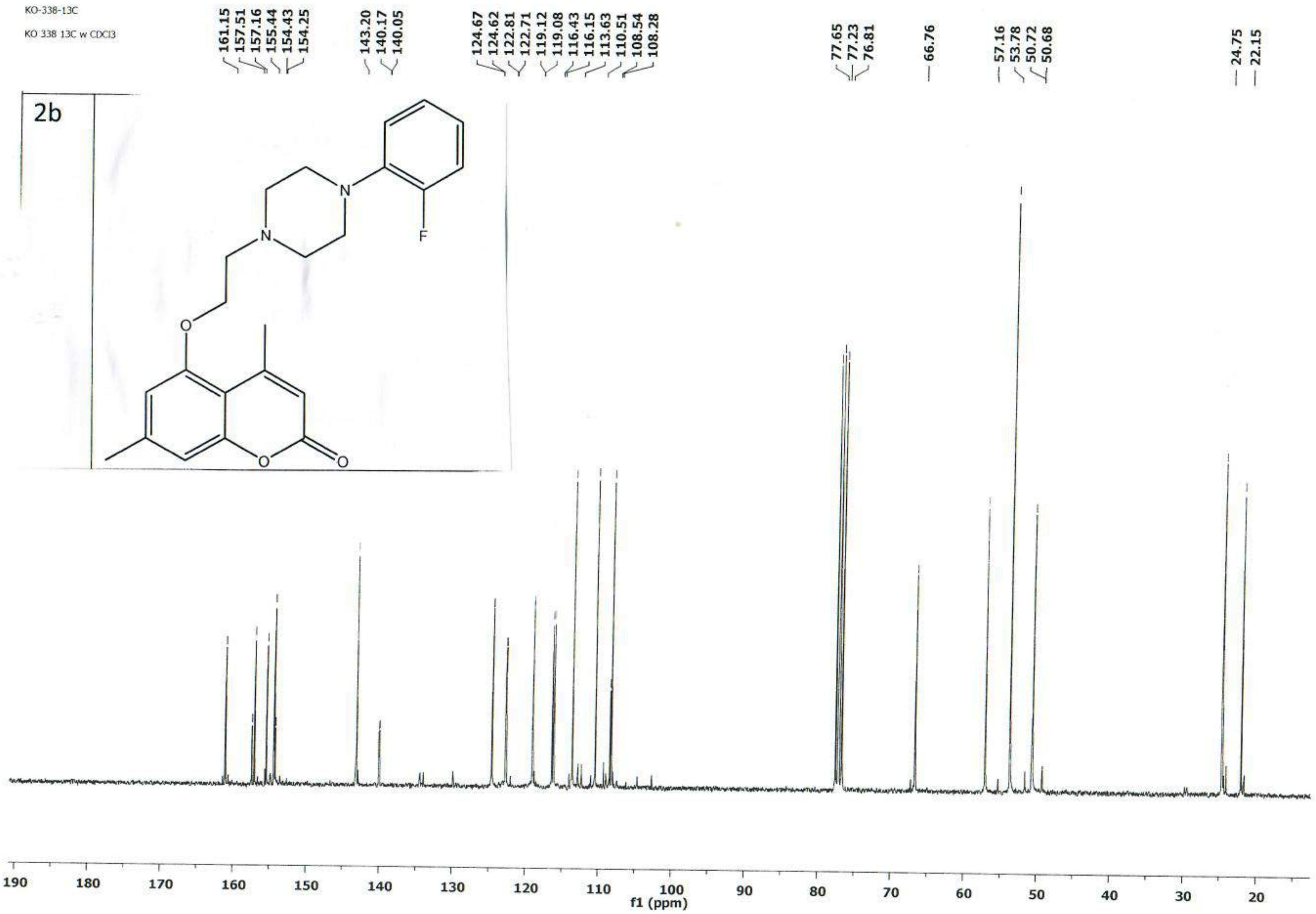
KO-338-13C
KO 338 13C w CDCl3
161.15
157.51
157.16
155.44
154.43
154.25
143.20
140.17
140.05
124.67
124.62
122.81
122.71
119.12
119.08
116.43
116.15
113.63
110.51
108.54
108.28
77.65
77.23
76.81
66.76
57.16
53.78
50.72
50.68
24.75
22.15
N
N
F
O
O
O
190
180
170
160
150
140
130
120
110
100
f1 (ppm)
90
80
70
60
50
40
30
20

2c

KO-337-1H

KO 337 1H w CDCl3

2c

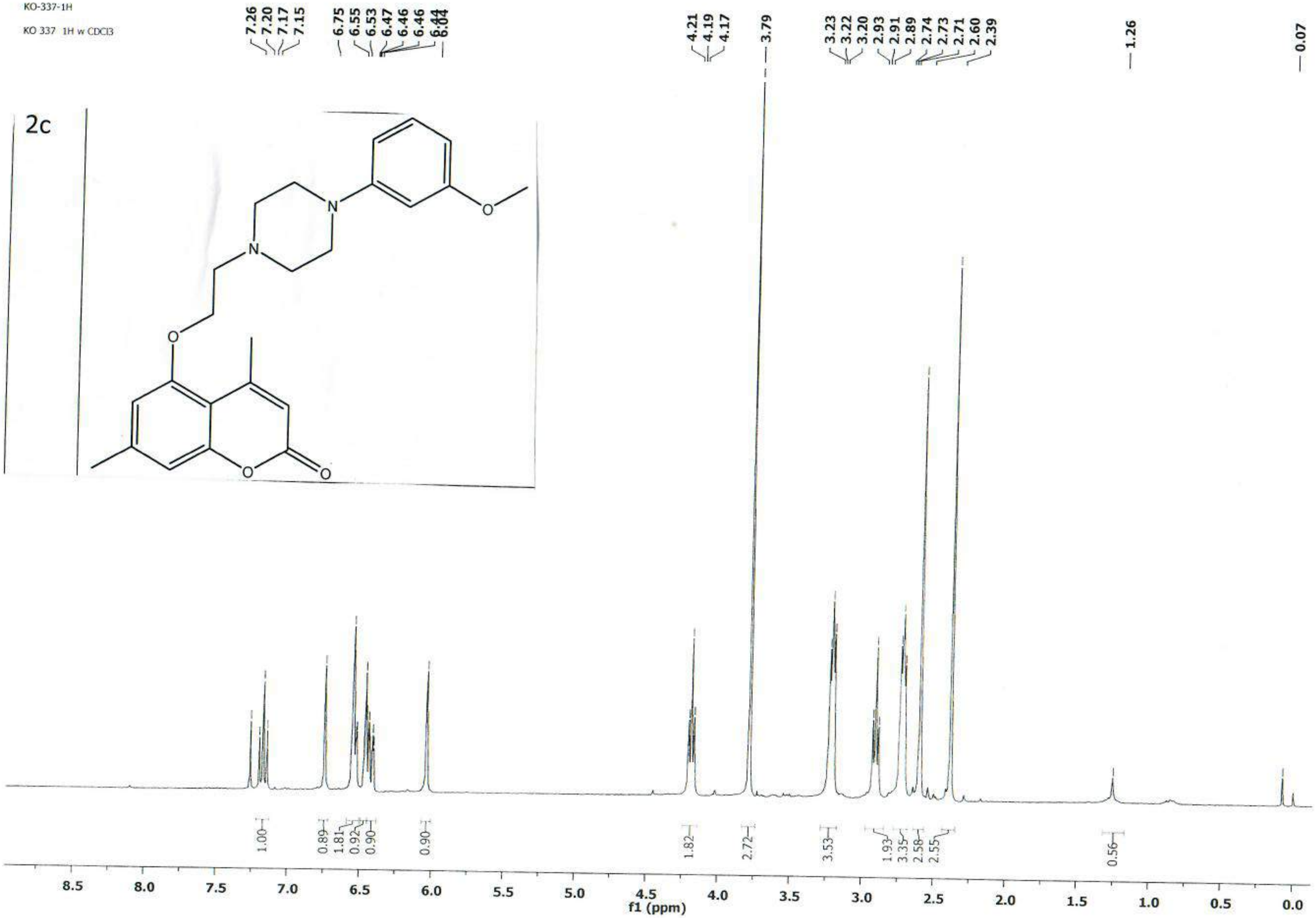

2c

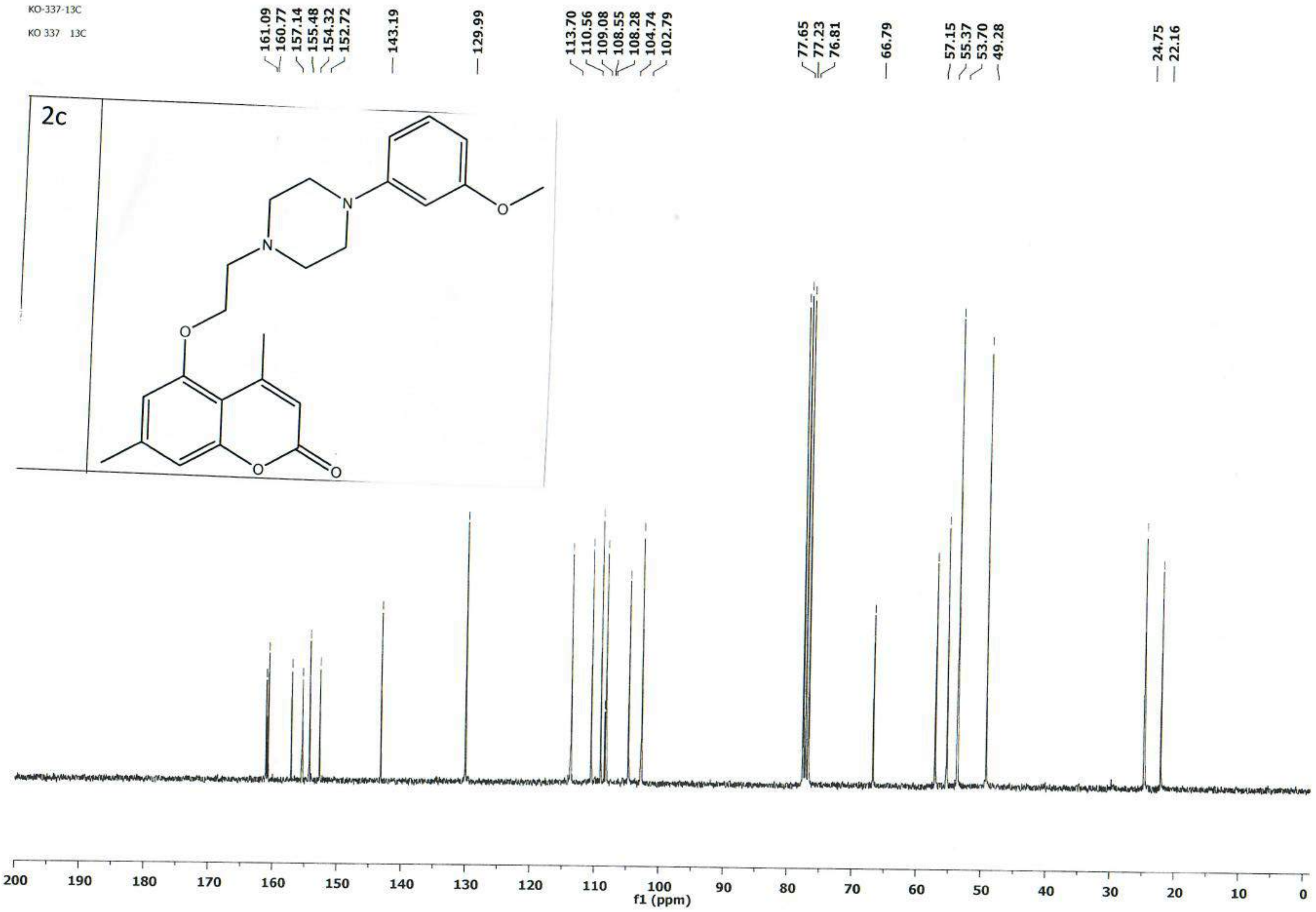
KO-337-13C
KO 337 13C
161.09
160.77
157.14
155.48
154.32
152.72
143.19
129.99
113.70
110.56
109.08
108.55
108.28
104.74
102.79
77.65
77.23
76.81
66.79
57.15
55.37
53.70
49.28
24.75
22.16
2c
N
N
O
O
O
O
200
190
180
170
160
150
140
130
120
110
100
90
80
70
60
50
40
30
20
10
0
f1 (ppm)

2d

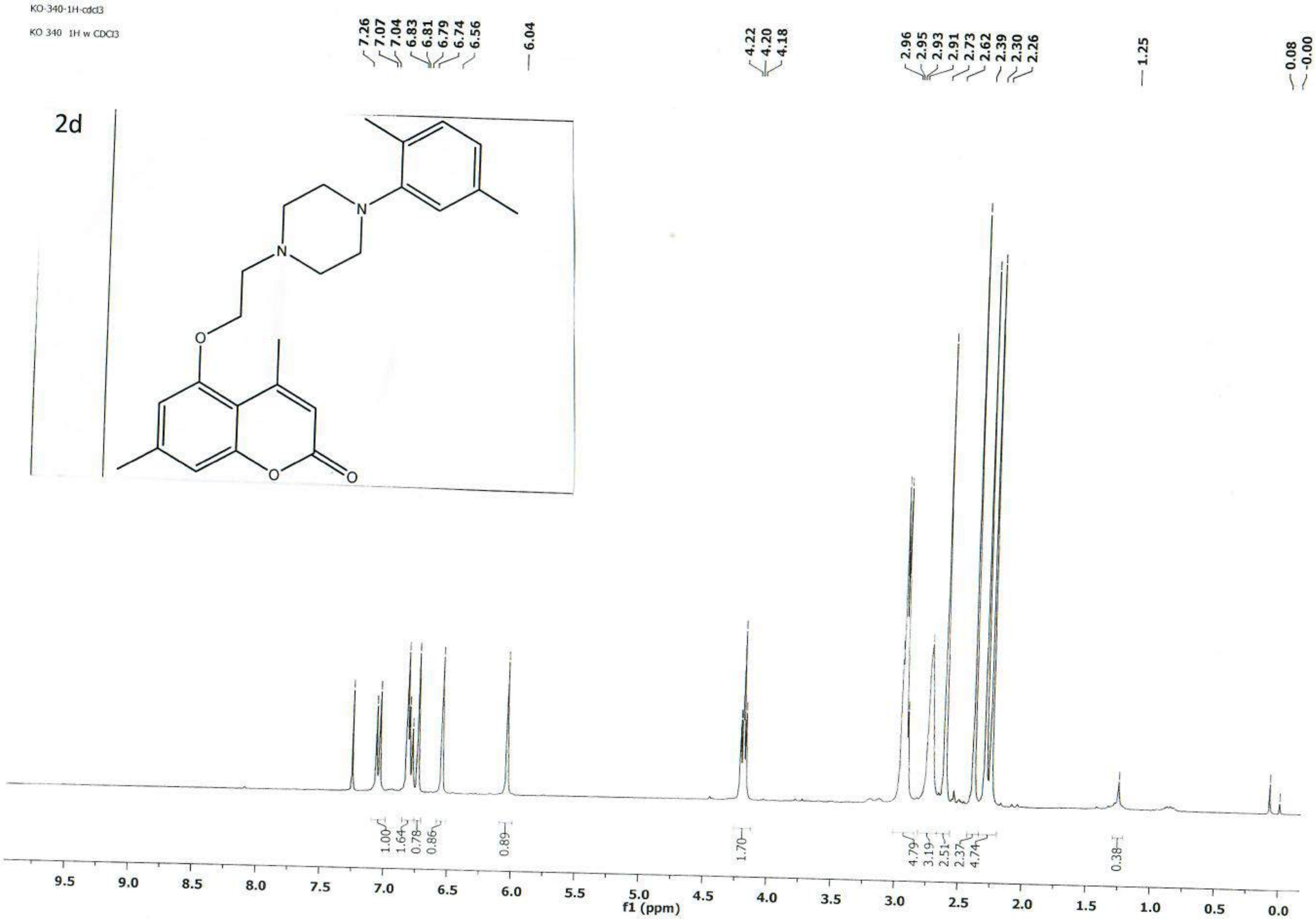
KO-340-1H-cdcl3
KO 340 1H w CDCl3
2d
N
N
O
O
O
7.26
7.07
7.04
6.83
6.81
6.79
6.74
6.56
6.04
4.22
4.20
4.18
2.96
2.95
2.93
2.91
2.73
2.62
2.39
2.30
2.26
1.25
0.08
-0.00
1.00
1.64
0.78
0.86
0.89
1.70
4.79
3.19
2.51
2.37
4.74
0.38
9.5
9.0
8.5
8.0
7.5
7.0
6.5
6.0
5.5
5.0
4.5
4.0
3.5
3.0
2.5
2.0
1.5
1.0
0.5
0.0
f1 (ppm)

2d

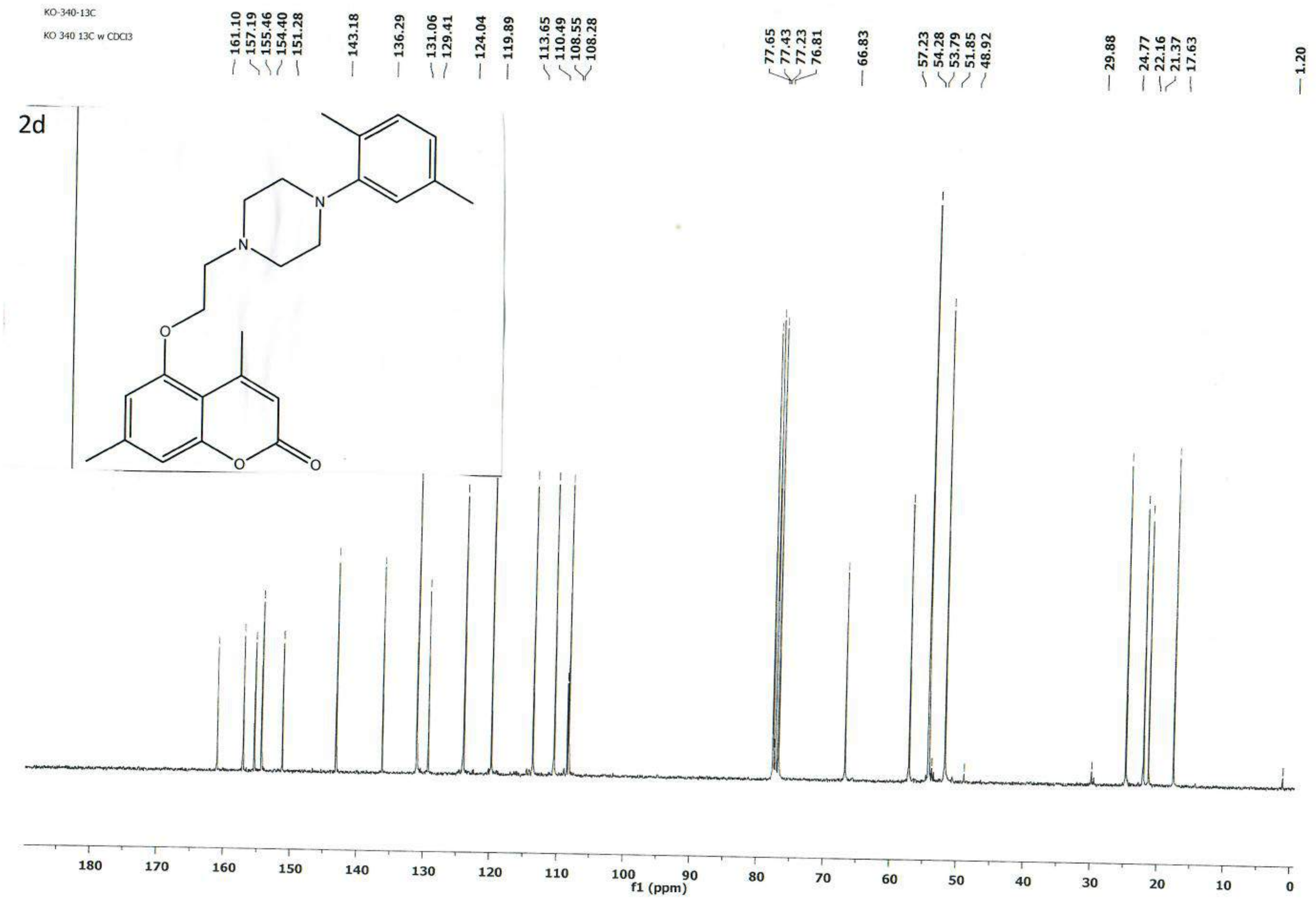
KO-340-13C
KO 340 13C w CDCl3
2d
161.10
157.19
155.46
154.40
151.28
143.18
136.29
131.06
129.41
124.04
119.89
113.65
110.49
108.55
108.28
77.65
77.43
77.23
76.81
66.83
57.23
54.28
53.79
51.85
48.92
29.88
24.77
22.16
21.37
17.63
1.20
180
170
160
150
140
130
120
110
100
90
80
70
60
50
40
30
20
10
0
f1 (ppm)

2e

KO-341-1H-cdcl3

KO 341 1H w CDCl3

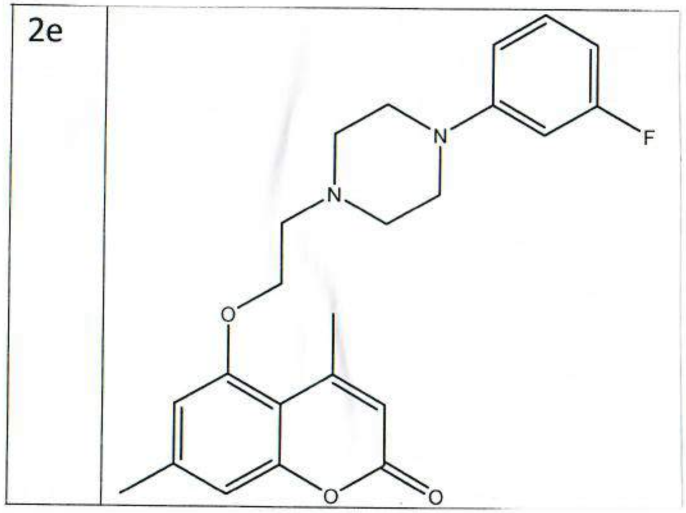

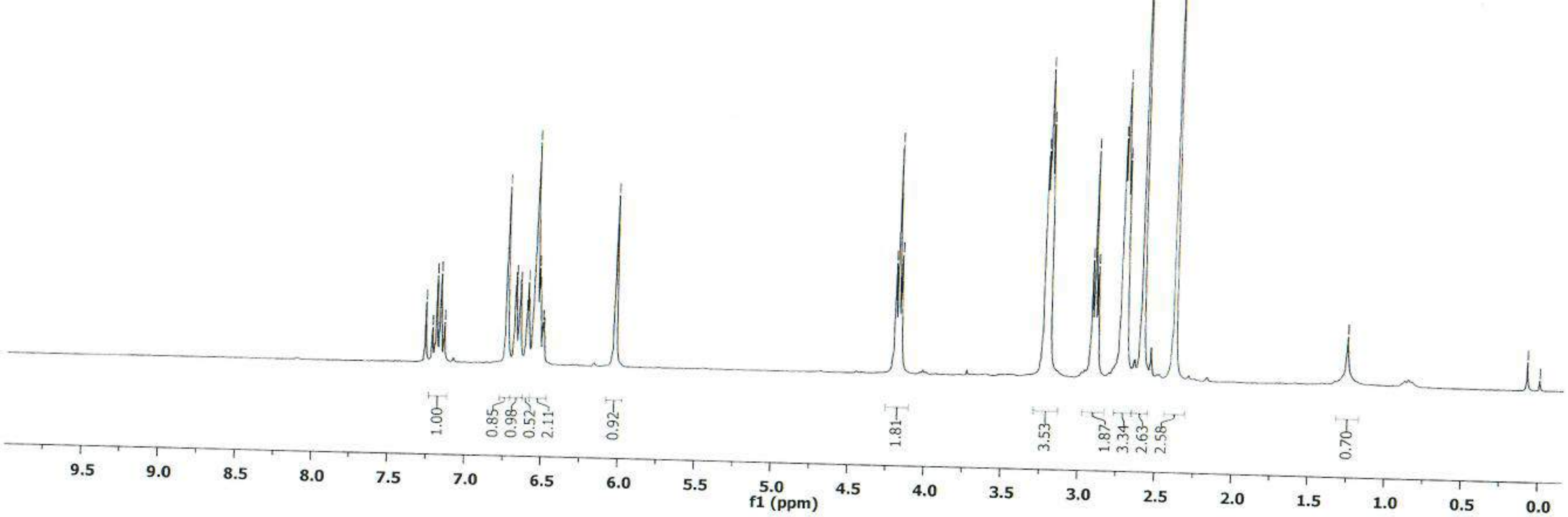

2e

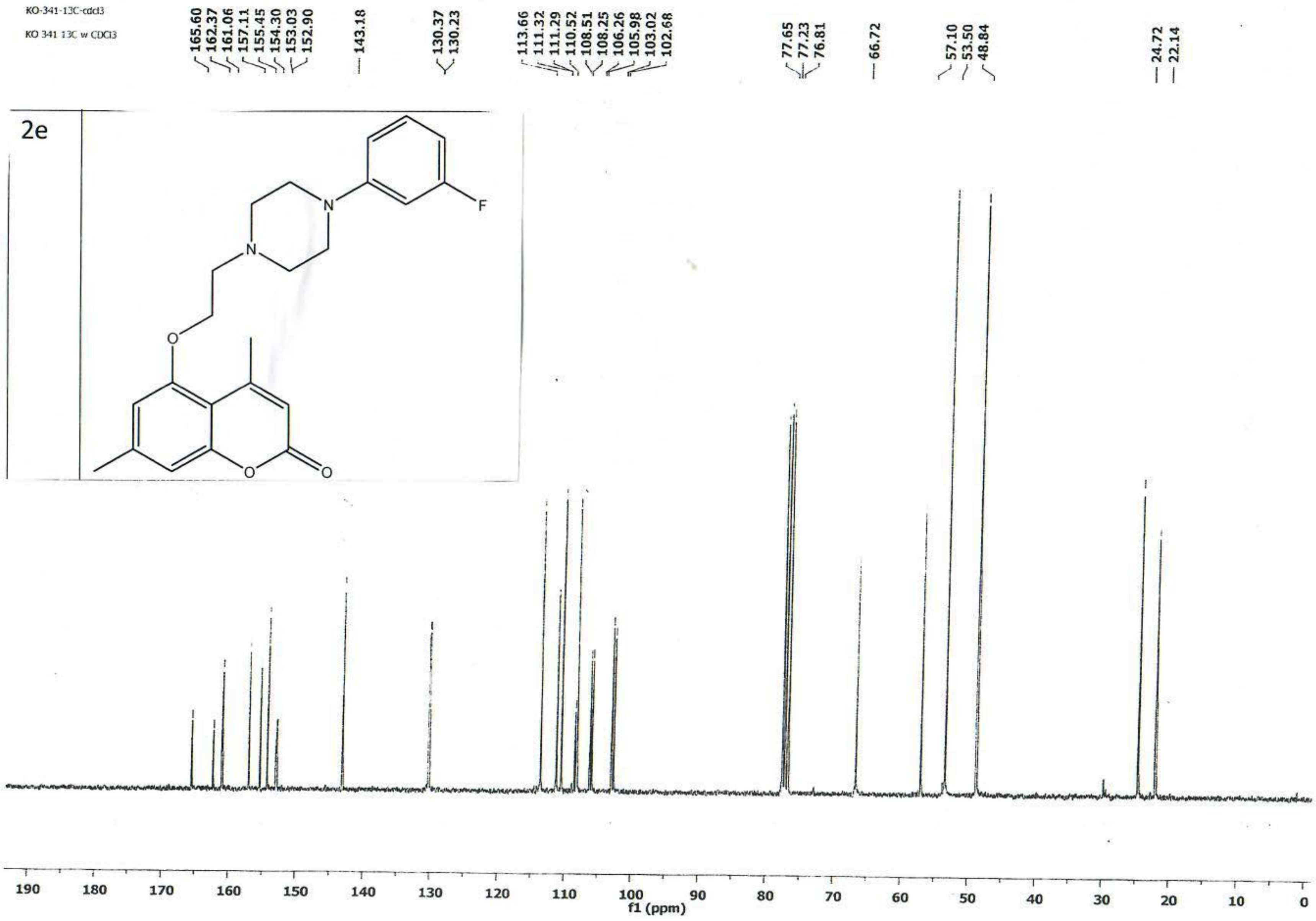
KO-341-13C-cdcl3
KO 341 13C w CDCl3
165.60
162.37
161.06
157.11
155.45
154.30
153.03
152.90
143.18
130.37
130.23
113.66
111.32
111.29
110.52
108.51
108.25
106.26
105.98
103.02
102.68
77.65
77.23
76.81
66.72
57.10
53.50
48.84
24.72
22.14
2e
N
N
F
O
O
O
190 180 170 160 150 140 130 120 110 100 90 80 70 60 50 40 30 20 10 0
f1 (ppm)

2f

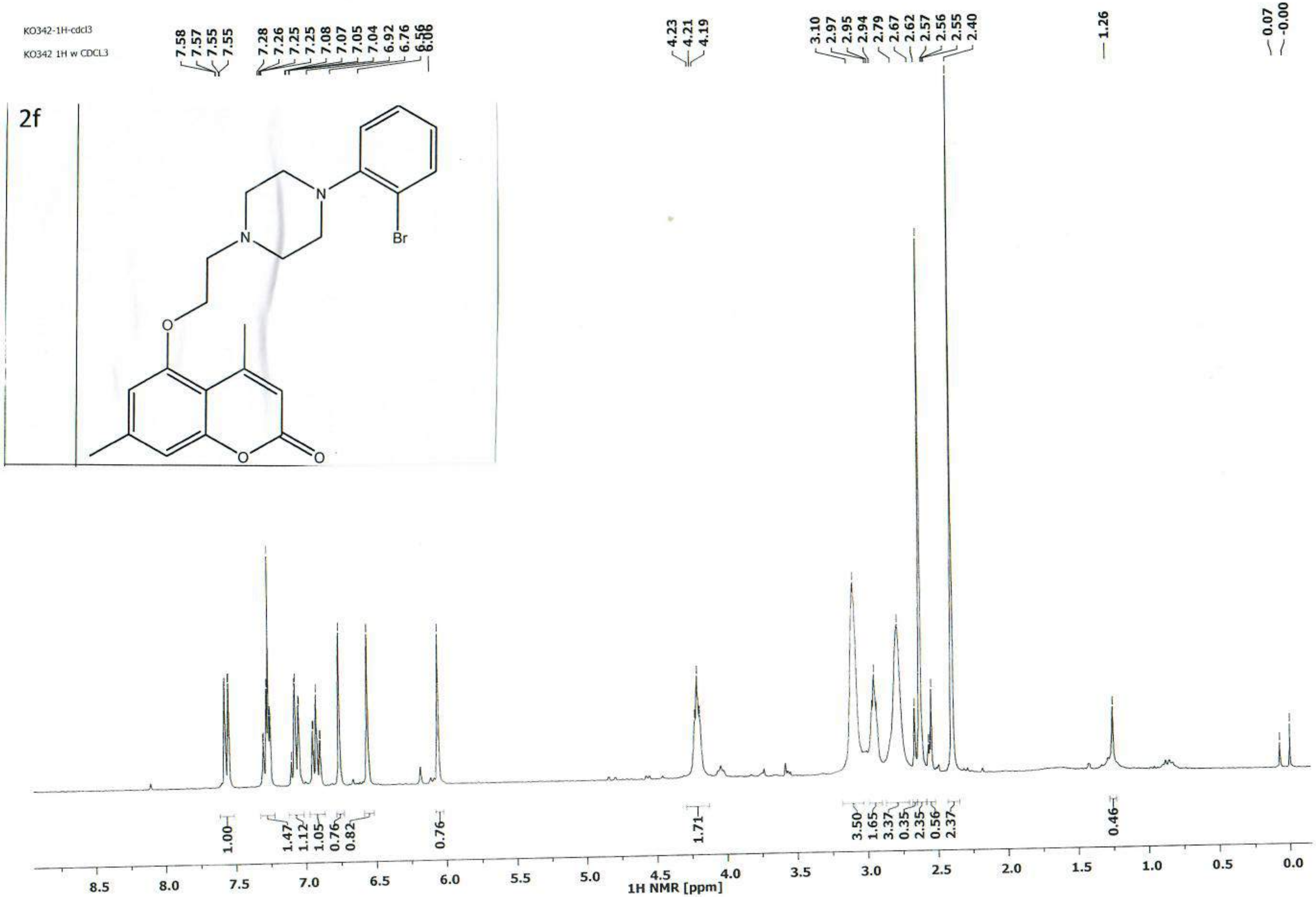

KO342-1H-cdcl3
KO342 1H w CDCL3
7.58 7.57 7.55 7.55 7.28 7.26 7.25 7.25 7.08 7.07 7.05 7.04 6.92 6.76 6.56 6.06
4.23 4.21 4.19
3.10 2.97 2.95 2.94 2.79 2.67 2.62 2.57 2.56 2.55 2.40
1.26
0.07 -0.00
2f
N
N
Br
O
O
O
1.00 1.47 1.12 1.05 0.76 0.82 0.76 1.71 3.50 1.65 3.37 0.35 2.35 0.56 2.37 0.46
8.5 8.0 7.5 7.0 6.5 6.0 5.5 5.0 4.5 4.0 3.5 3.0 2.5 2.0 1.5 1.0 0.5 0.0
1H NMR [ppm]

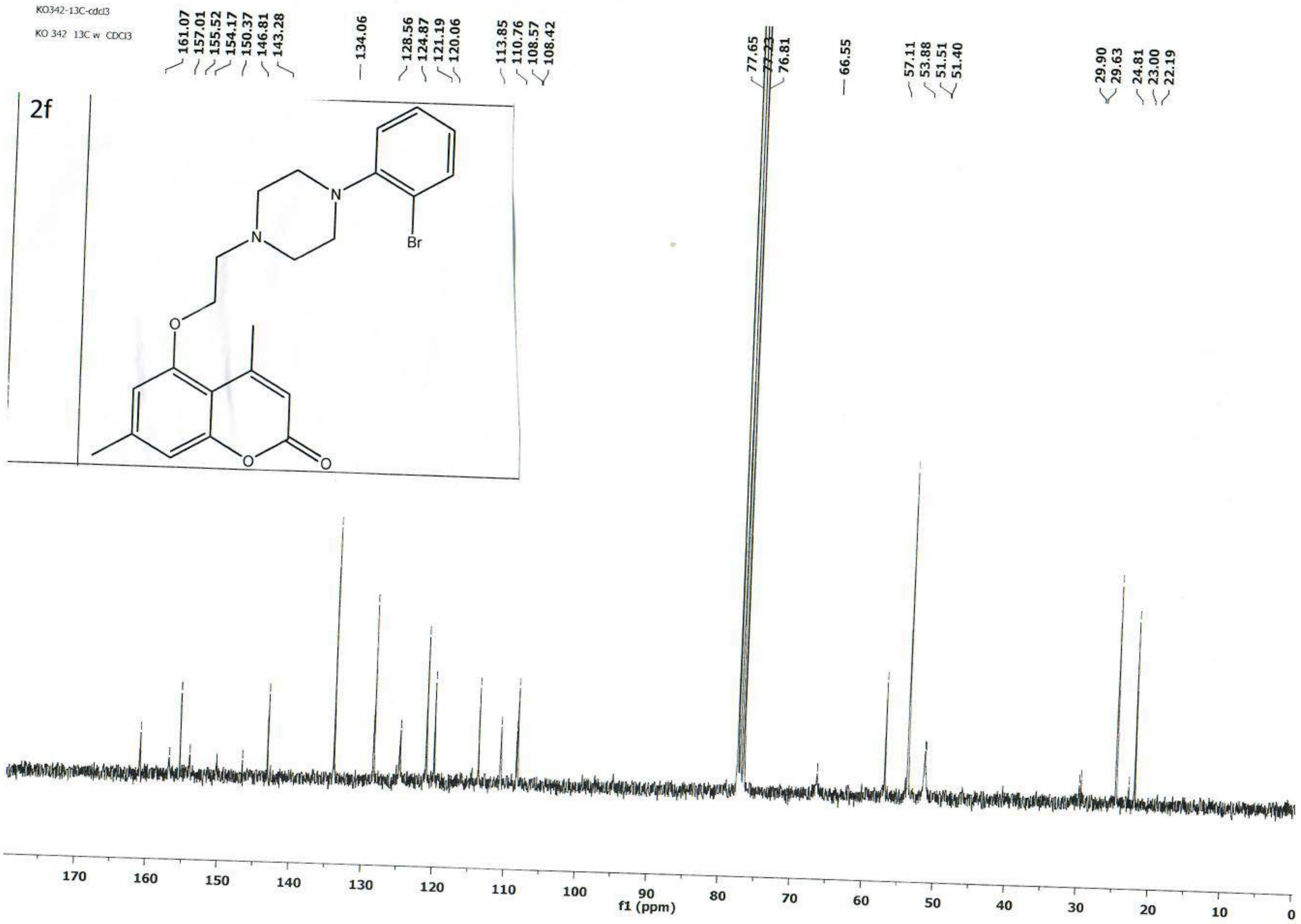
KO342-13C-cdcl3
KO 342 13C w CDCl3
161.07
157.01
155.52
154.17
150.37
146.81
143.28
134.06
128.56
124.87
121.19
120.06
113.85
110.76
108.57
108.42
77.65
77.23
76.81
66.55
57.11
53.88
51.51
51.40
29.90
29.63
24.81
23.00
22.19
2f
N
N
Br
O
O
O
170 160 150 140 130 120 110 100 90 80 70 60 50 40 30 20 10 0
f1 (ppm)

2g

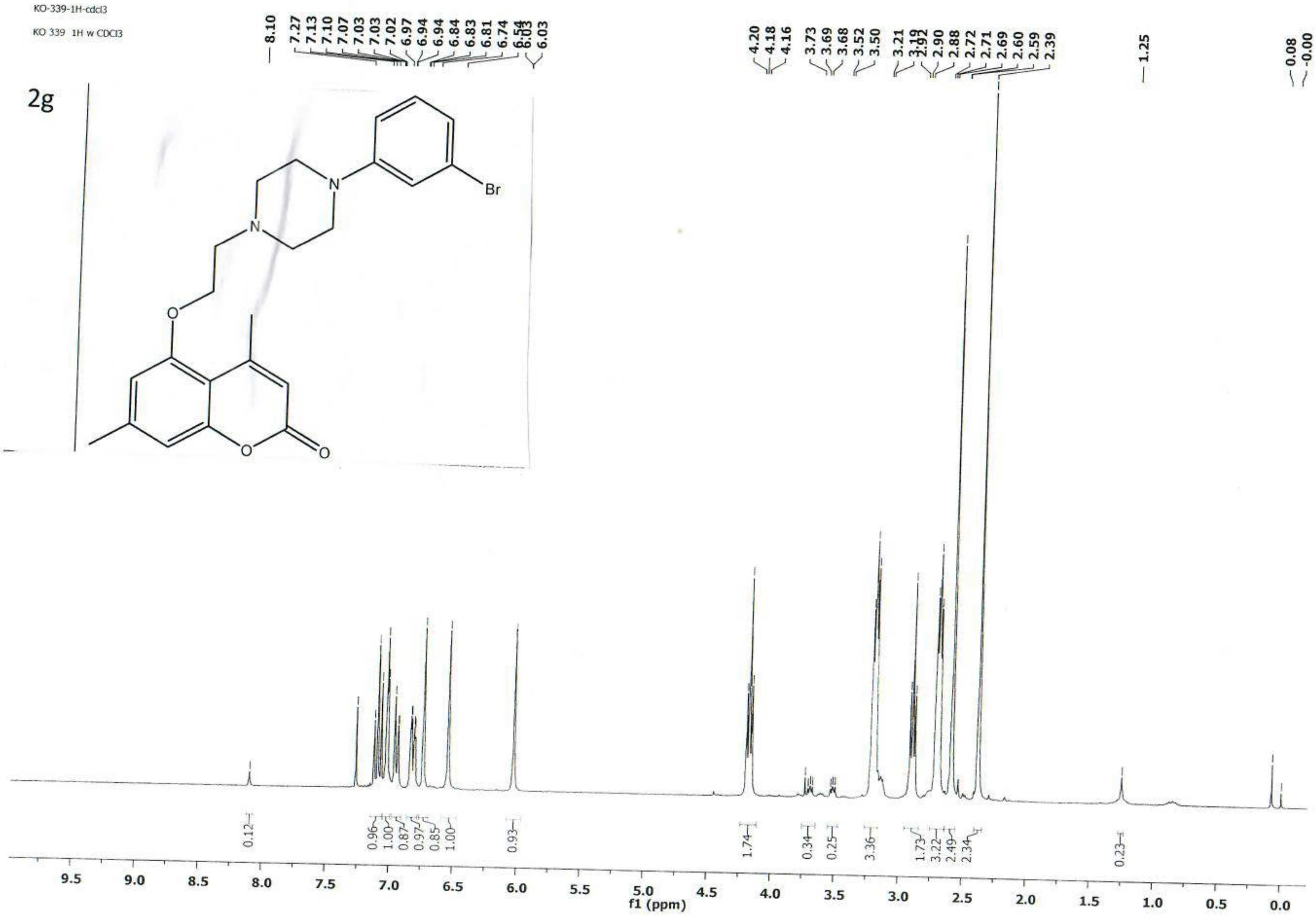

KO-339-1H-cdcl3
KO 339 1H w CDCl3
2g
N
N
Br
O
O
O
8.10
7.27
7.13
7.10
7.07
7.03
7.03
7.02
6.97
6.94
6.94
6.84
6.83
6.81
6.74
6.54
6.53
6.03
4.20
4.18
4.16
3.73
3.69
3.68
3.52
3.50
3.21
3.19
2.92
2.90
2.88
2.72
2.71
2.69
2.60
2.59
2.39
1.25
0.08
-0.00
0.12
0.96
1.00
0.87
0.97
0.85
1.00
0.93
1.74
0.34
0.25
3.36
1.73
3.22
2.49
2.34
0.23
9.5
9.0
8.5
8.0
7.5
7.0
6.5
6.0
5.5
5.0
4.5
4.0
3.5
3.0
2.5
2.0
1.5
1.0
0.5
0.0
f1 (ppm)

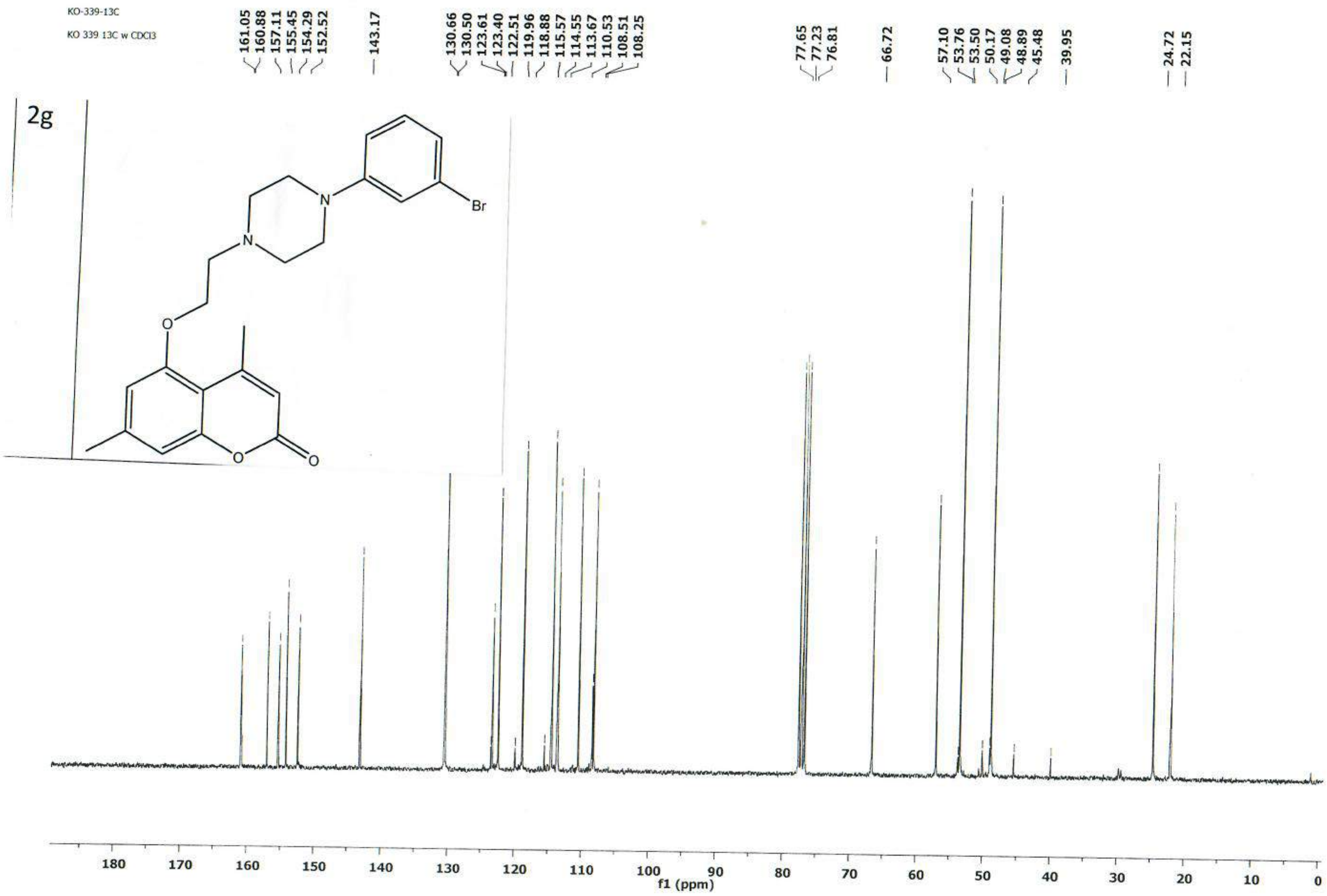
KO-339-13C
KO 339 13C w CDCl3
2g
Br
N
N
O
O
O
161.05
160.88
157.11
155.45
154.29
152.52
143.17
130.66
130.50
123.61
123.40
122.51
119.96
118.88
115.57
114.55
113.67
110.53
108.51
108.25
77.65
77.23
76.81
66.72
57.10
53.76
53.50
50.17
49.08
48.89
45.48
39.95
24.72
22.15
180
170
160
150
140
130
120
110
100
90
80
70
60
50
40
30
20
10
0
f1 (ppm)

2h

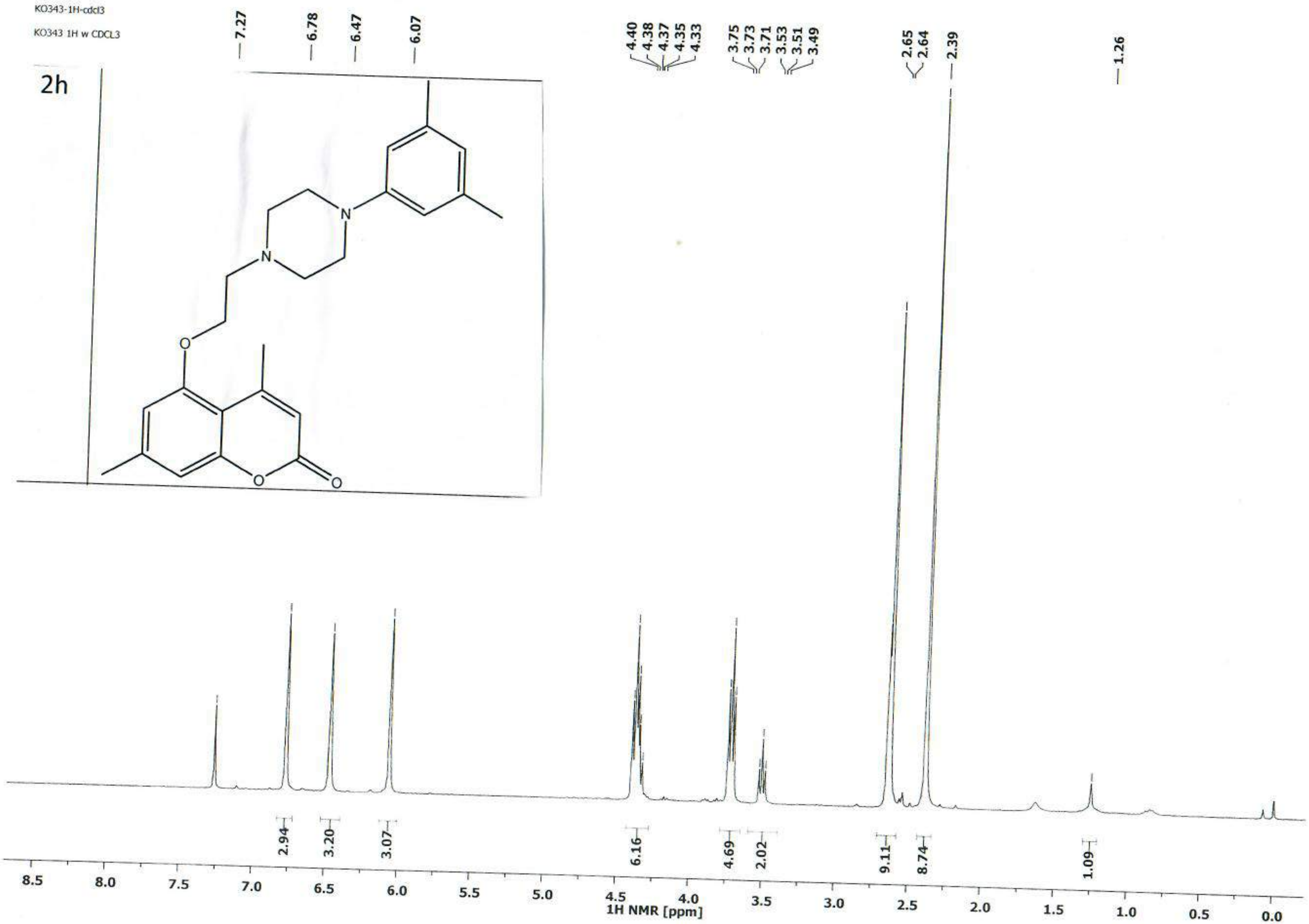

KO343-1H-cdcl3
KO343 1H w CDCL3
2h
7.27
6.78
6.47
6.07
4.40
4.38
4.37
4.35
4.33
3.75
3.73
3.71
3.53
3.51
3.49
2.65
2.64
2.39
1.26
2.94
3.20
3.07
6.16
4.69
2.02
9.11
8.74
1.09
8.5
8.0
7.5
7.0
6.5
6.0
5.5
5.0
4.5
4.0
3.5
3.0
2.5
2.0
1.5
1.0
0.5
0.0
1H NMR [ppm]

2h

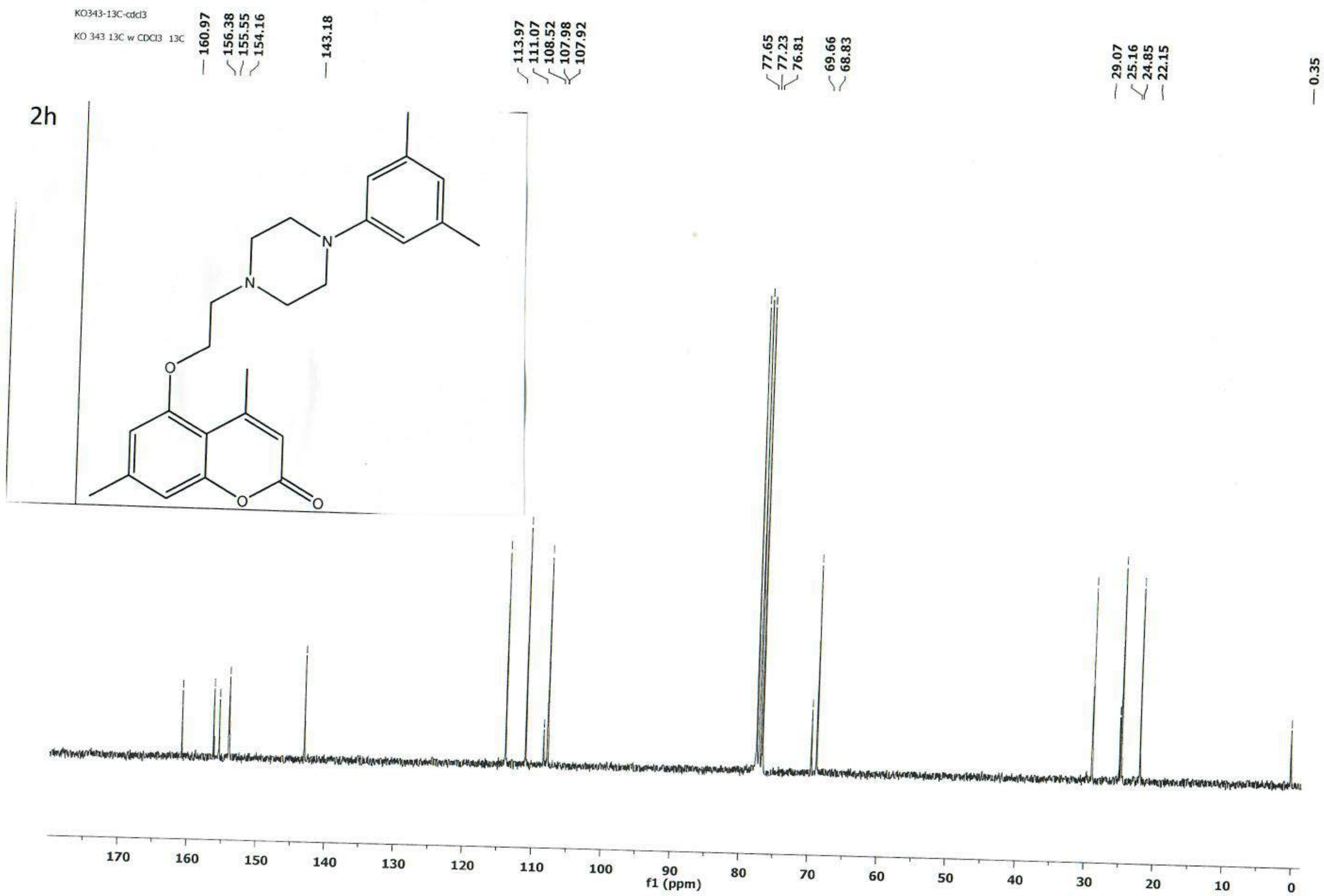
KO343-13C-cdcl3
KO 343 13C w CDCl3 13C
160.97
156.38
155.55
154.16
143.18
113.97
111.07
108.52
107.98
107.92
77.65
77.23
76.81
69.66
68.83
29.07
25.16
24.85
22.15
0.35
2h
N
N
O
O
O
170
160
150
140
130
120
110
100
90
f1 (ppm)
80
70
60
50
40
30
20
10
0

2i

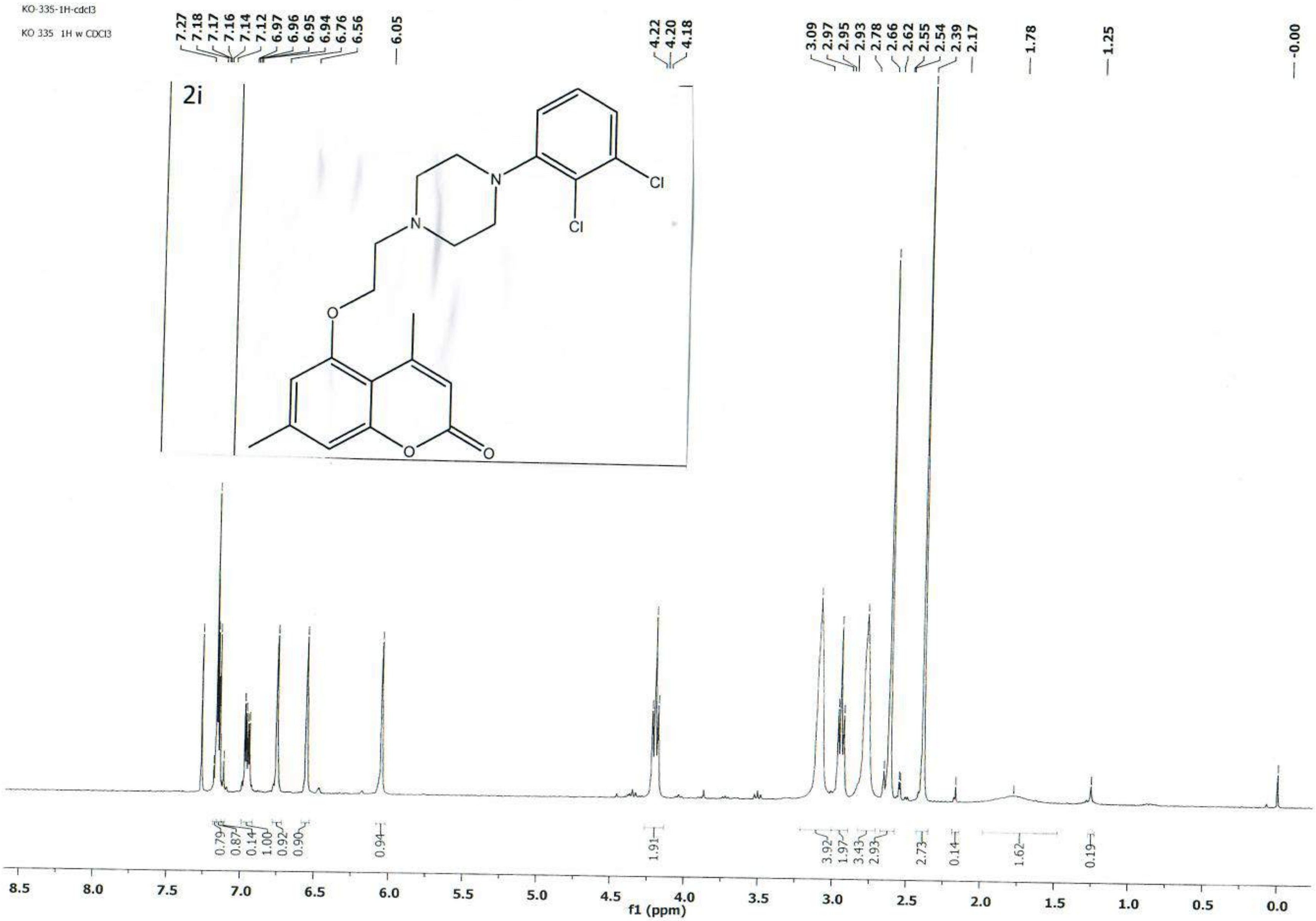

KO-335-1H-cdcl3
KO 335 1H w CDCl3
7.27
7.18
7.17
7.16
7.14
7.12
6.97
6.96
6.95
6.94
6.76
6.56
6.05
4.22
4.20
4.18
3.09
2.97
2.95
2.93
2.78
2.66
2.62
2.55
2.54
2.39
2.17
1.78
1.25
-0.00
2i
N
N
Cl
Cl
O
O
O
0.79
0.87
0.14
1.00
0.92
0.90
0.94
1.91
3.92
1.97
3.43
2.93
2.73
0.14
1.62
0.19
8.5
8.0
7.5
7.0
6.5
6.0
5.5
5.0
4.5
4.0
3.5
3.0
2.5
2.0
1.5
1.0
0.5
0.0
f1 (ppm)

2i

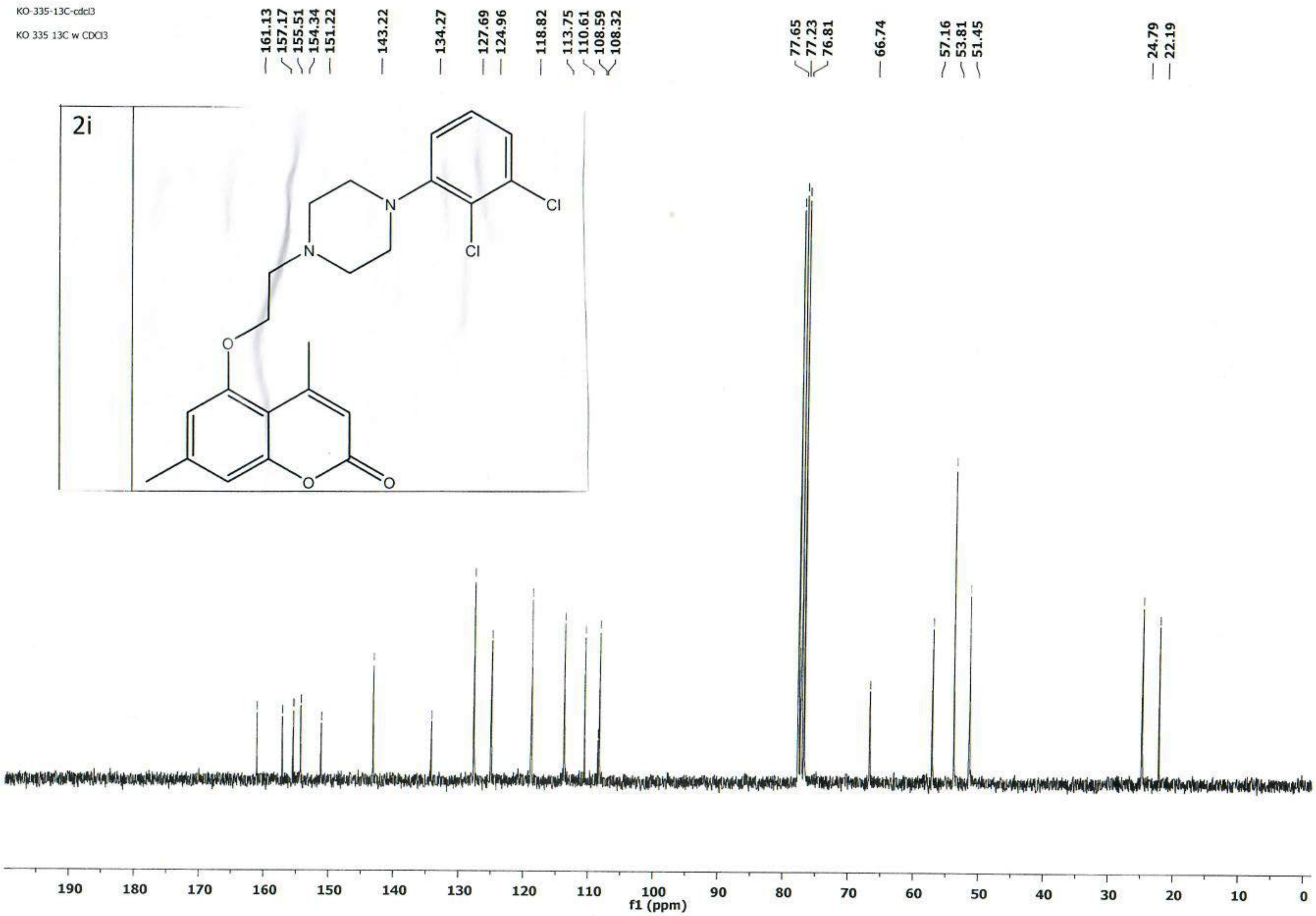
KO-335-13C-cdcl3
KO 335 13C w CDCl3
161.13
157.17
155.51
154.34
151.22
143.22
134.27
127.69
124.96
118.82
113.75
110.61
108.59
108.32
77.65
77.23
76.81
66.74
57.16
53.81
51.45
24.79
22.19
2i
N
N
Cl
Cl
O
O
O
190
180
170
160
150
140
130
120
110
100
90
80
70
60
50
40
30
20
10
0
f1 (ppm)

2j

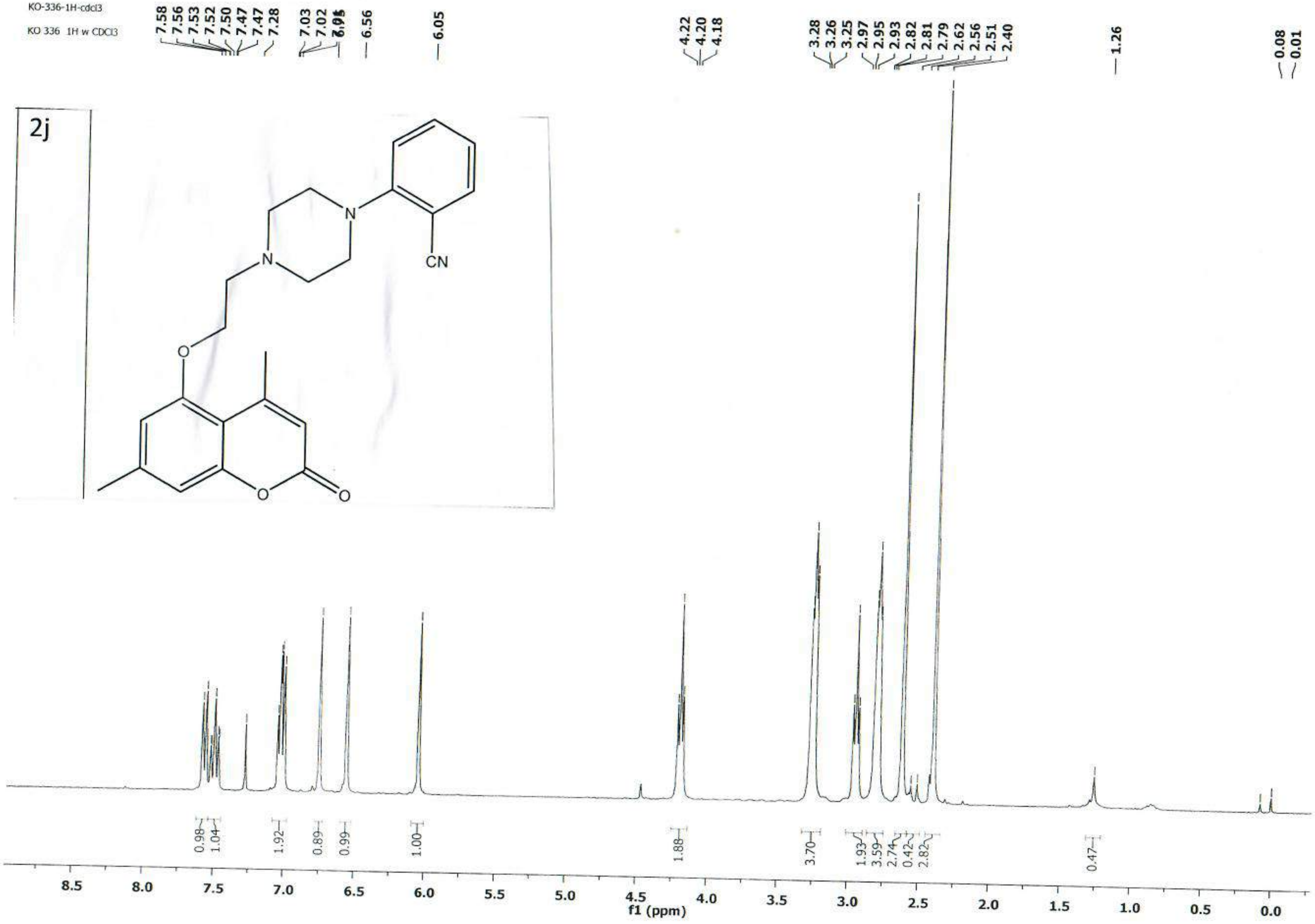
KO-336-1H-cdcl3
KO 336 1H w CDCl3
7.58
7.56
7.53
7.52
7.50
7.47
7.47
7.28
7.03
7.02
6.56
6.05
4.22
4.20
4.18
3.28
3.26
3.25
2.97
2.95
2.93
2.82
2.81
2.79
2.62
2.56
2.51
2.40
1.26
0.08
0.01
2j
N
N
CN
O
O
O
0.98
1.04
1.92
0.89
0.99
1.00
1.88
3.70
1.93
3.59
2.74
0.42
2.82
0.47
8.5
8.0
7.5
7.0
6.5
6.0
5.5
5.0
4.5
4.0
3.5
3.0
2.5
2.0
1.5
1.0
0.5
0.0
f1 (ppm)

2j

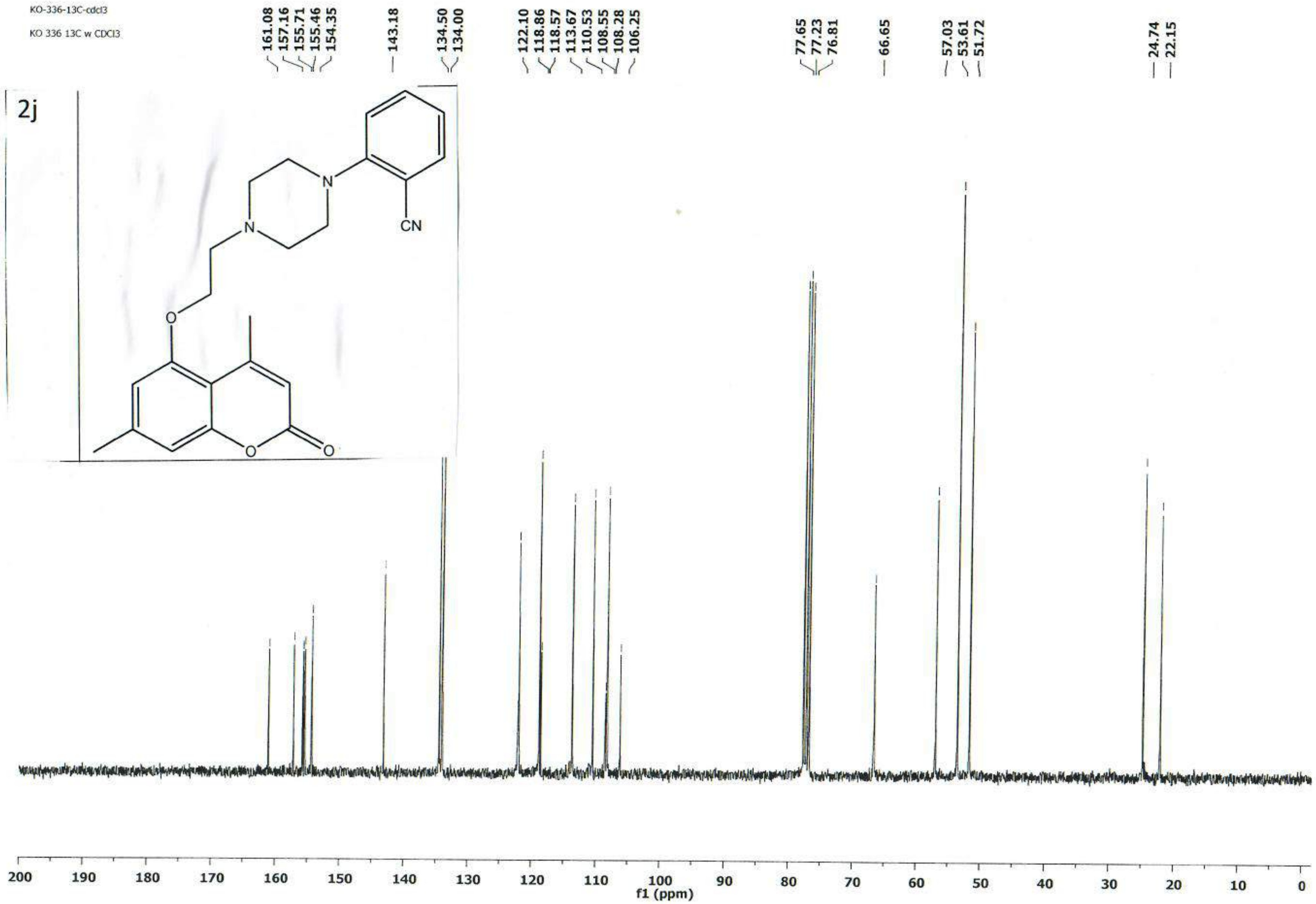

KO-336-13C-cdcl3
KO 336 13C w CDCl3
161.08
157.16
155.71
155.46
154.35
143.18
134.50
134.00
122.10
118.86
118.57
113.67
110.53
108.55
108.28
106.25
77.65
77.23
76.81
66.65
57.03
53.61
51.72
24.74
22.15
2j
N
N
CN
O
O
O
200 190 180 170 160 150 140 130 120 110 100 90 80 70 60 50 40 30 20 10 0
f1 (ppm)

3

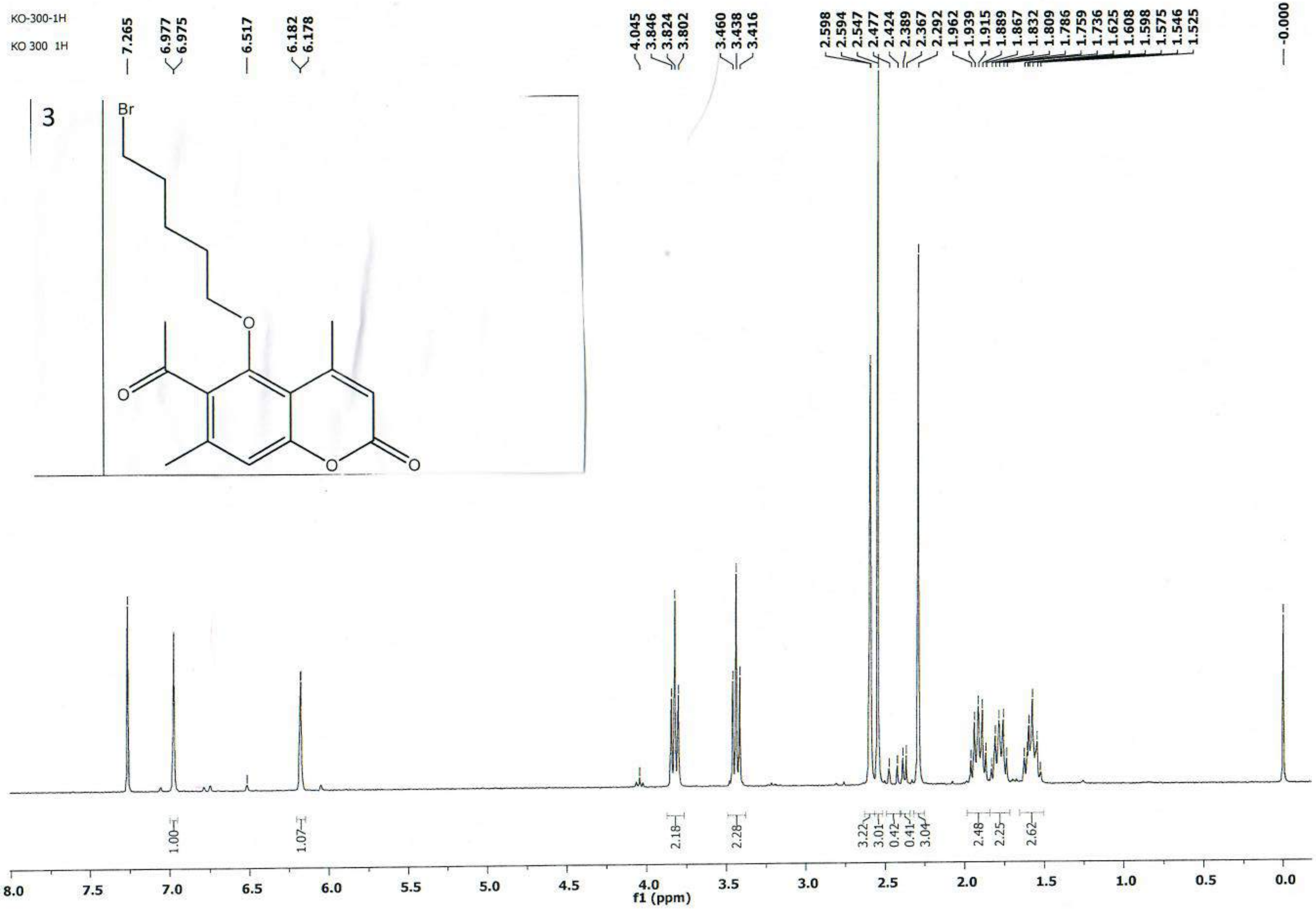
KO-300-1H
KO 300 1H
7.265
6.977
6.975
6.517
6.182
6.178
4.045
3.846
3.824
3.802
3.460
3.438
3.416
2.598
2.594
2.547
2.477
2.424
2.389
2.367
2.292
1.962
1.939
1.915
1.889
1.867
1.832
1.809
1.786
1.759
1.736
1.625
1.608
1.598
1.575
1.546
1.525
-0.000
3
Br
O
O
O
O
1.00
1.07
2.18
2.28
3.22
3.01
0.42
0.41
3.04
2.48
2.25
2.62
8.0
7.5
7.0
6.5
6.0
5.5
5.0
4.5
4.0
3.5
3.0
2.5
2.0
1.5
1.0
0.5
0.0
f1 (ppm)

3

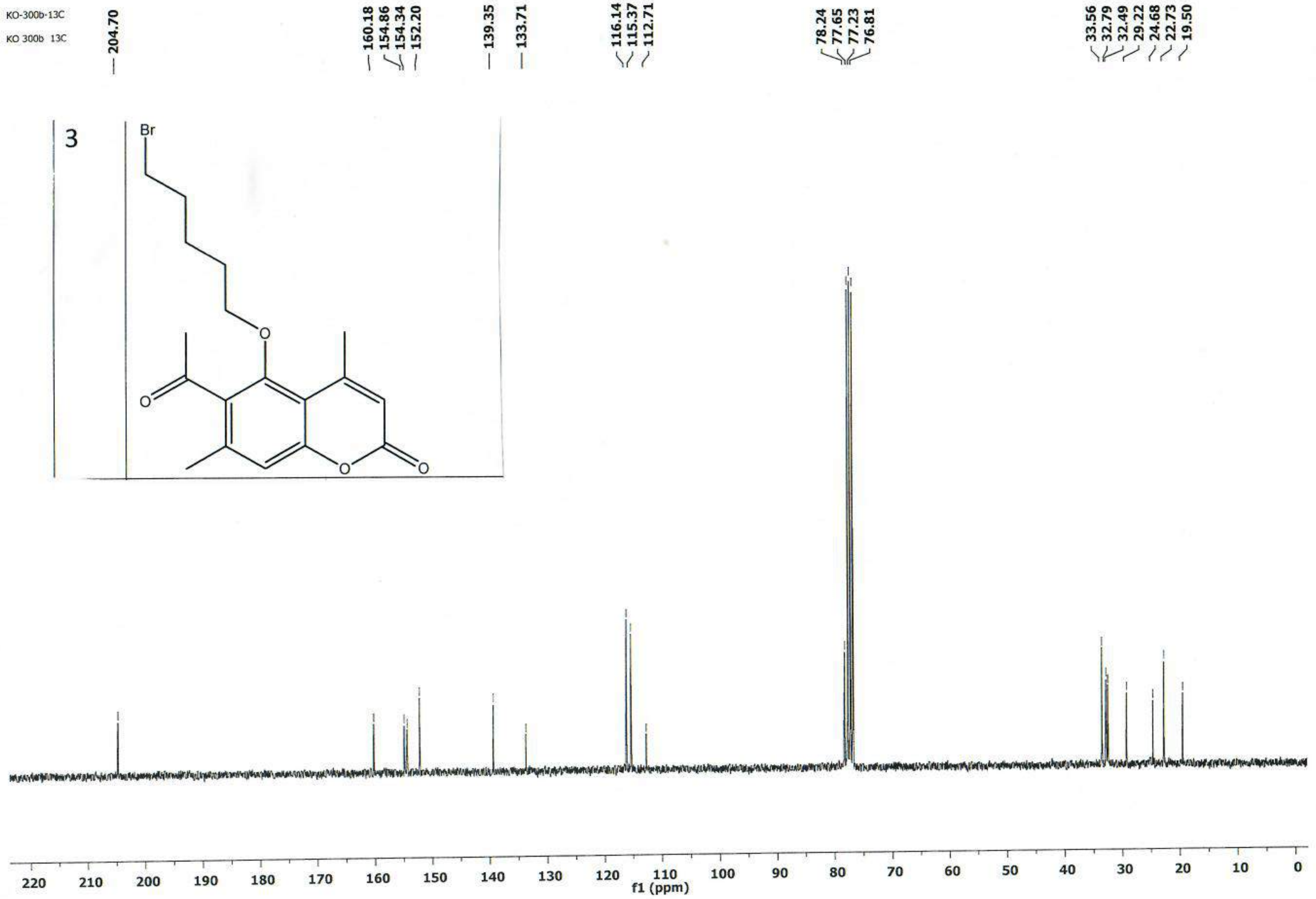
KO-300b-13C
KO 300b 13C
204.70
160.18
154.86
154.34
152.20
139.35
133.71
116.14
115.37
112.71
78.24
77.65
77.23
76.81
33.56
32.79
32.49
29.22
24.68
22.73
19.50
3
Br
O
O
O
O
220 210 200 190 180 170 160 150 140 130 120 110 100 90 80 70 60 50 40 30 20 10 0
f1 (ppm)

3a

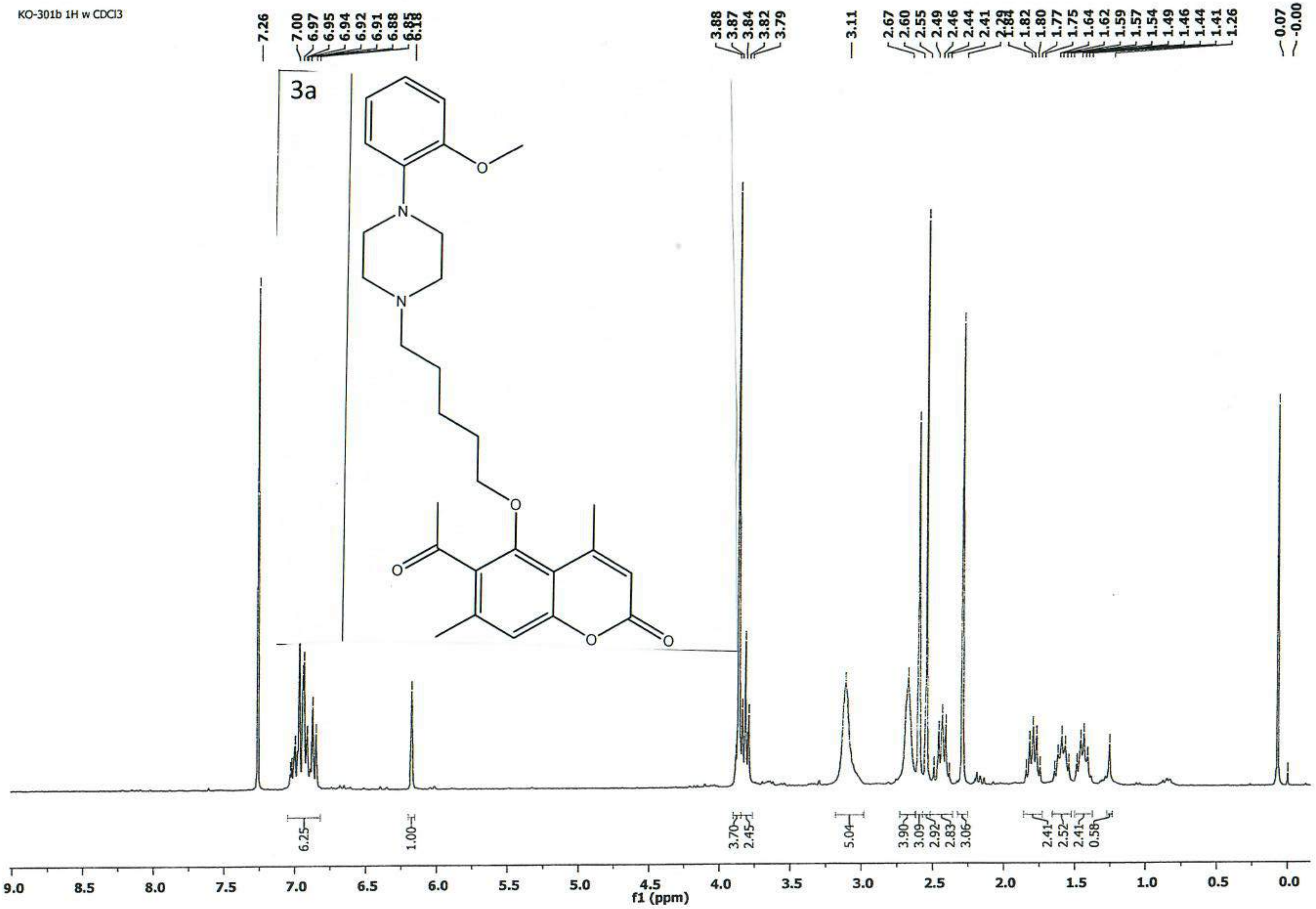

3a

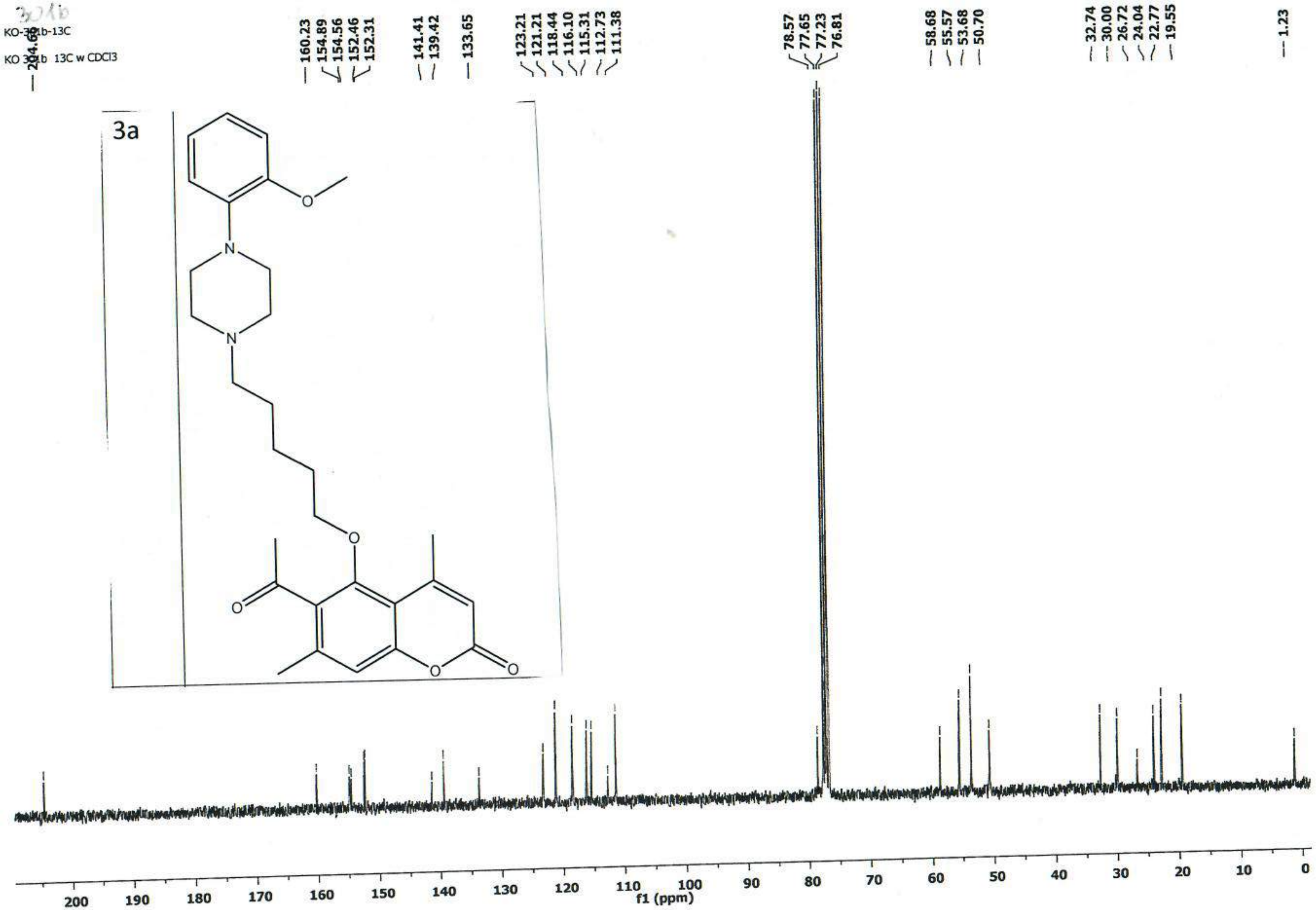
13C w CDCl3
204.60
160.23
154.89
154.56
152.46
152.31
141.41
139.42
133.65
123.21
121.21
118.44
116.10
115.31
112.73
111.38
78.57
77.65
77.23
76.81
58.68
55.57
53.68
50.70
32.74
30.00
26.72
24.04
22.77
19.55
1.23
3a
N
N
O
O
O
O
O
200 190 180 170 160 150 140 130 120 110 100 90 80 70 60 50 40 30 20 10 0
f1 (ppm)

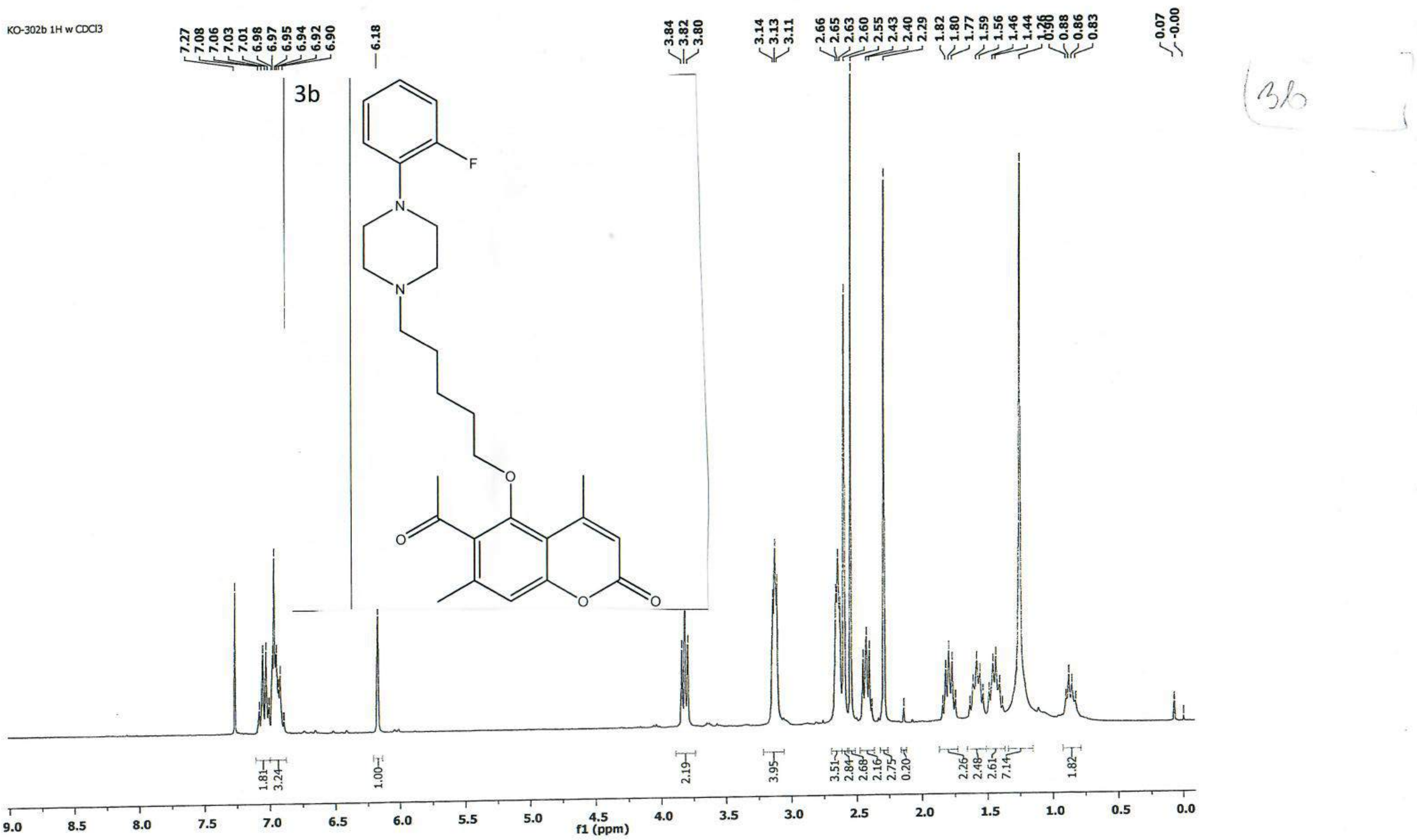
KO-302b 1H w CDCl3
3b
F
N
N
O
O
O
O
f1 (ppm)
(3b)

KO-302b-13C

KO 302b 13C w CDCl3

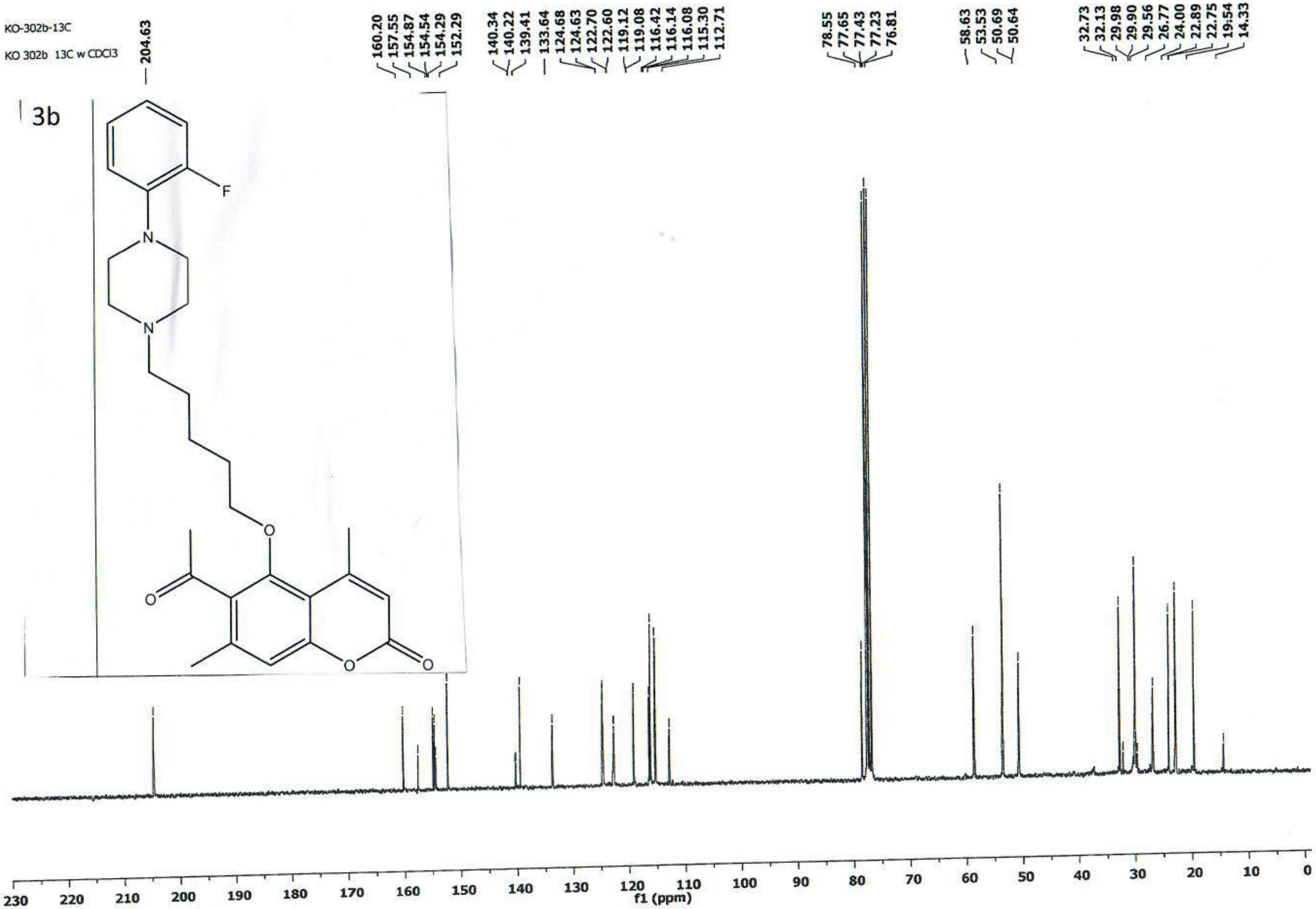

KO-303

KO303 w CDCl3 + TMS

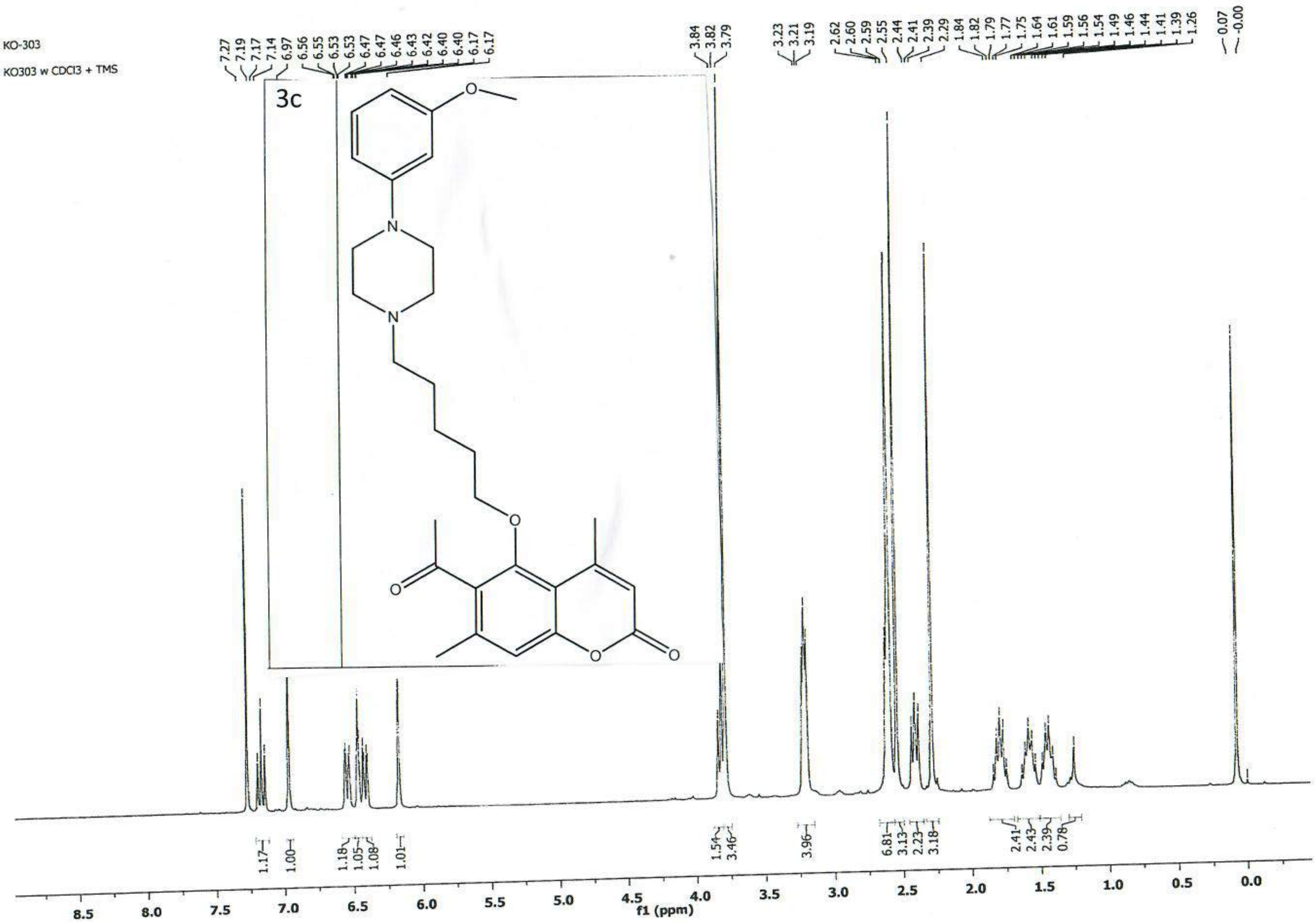

3c

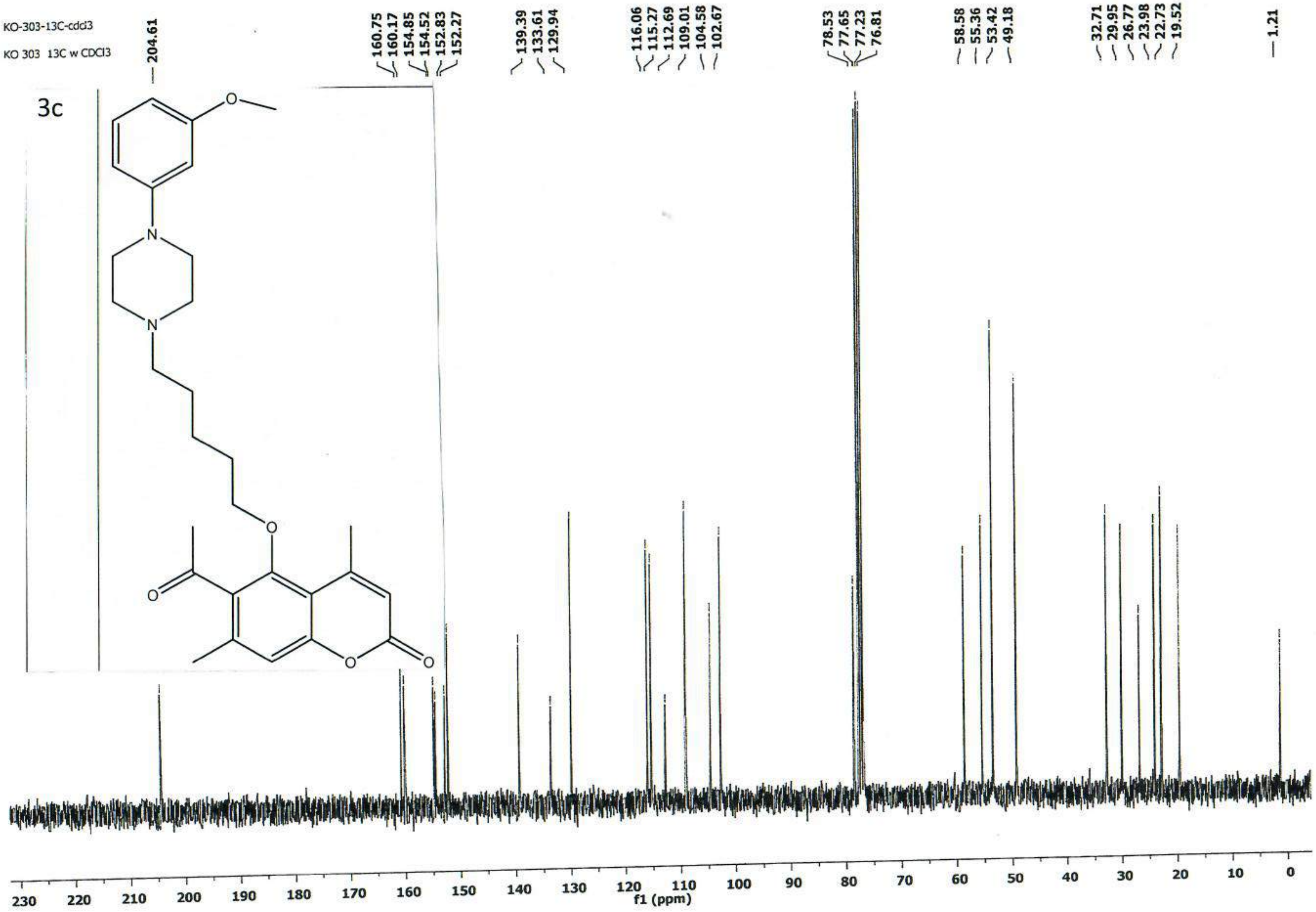

KO-303-13C-cdcl3
KO 303 13C w CDCl3
3c
204.61
160.75
160.17
154.85
154.52
152.83
152.27
139.39
133.61
129.94
116.06
115.27
112.69
109.01
104.58
102.67
78.53
77.65
77.23
76.81
58.58
55.36
53.42
49.18
32.71
29.95
26.77
23.98
22.73
19.52
1.21
230 220 210 200 190 180 170 160 150 140 130 120 110 100 90 80 70 60 50 40 30 20 10 0
f1 (ppm)

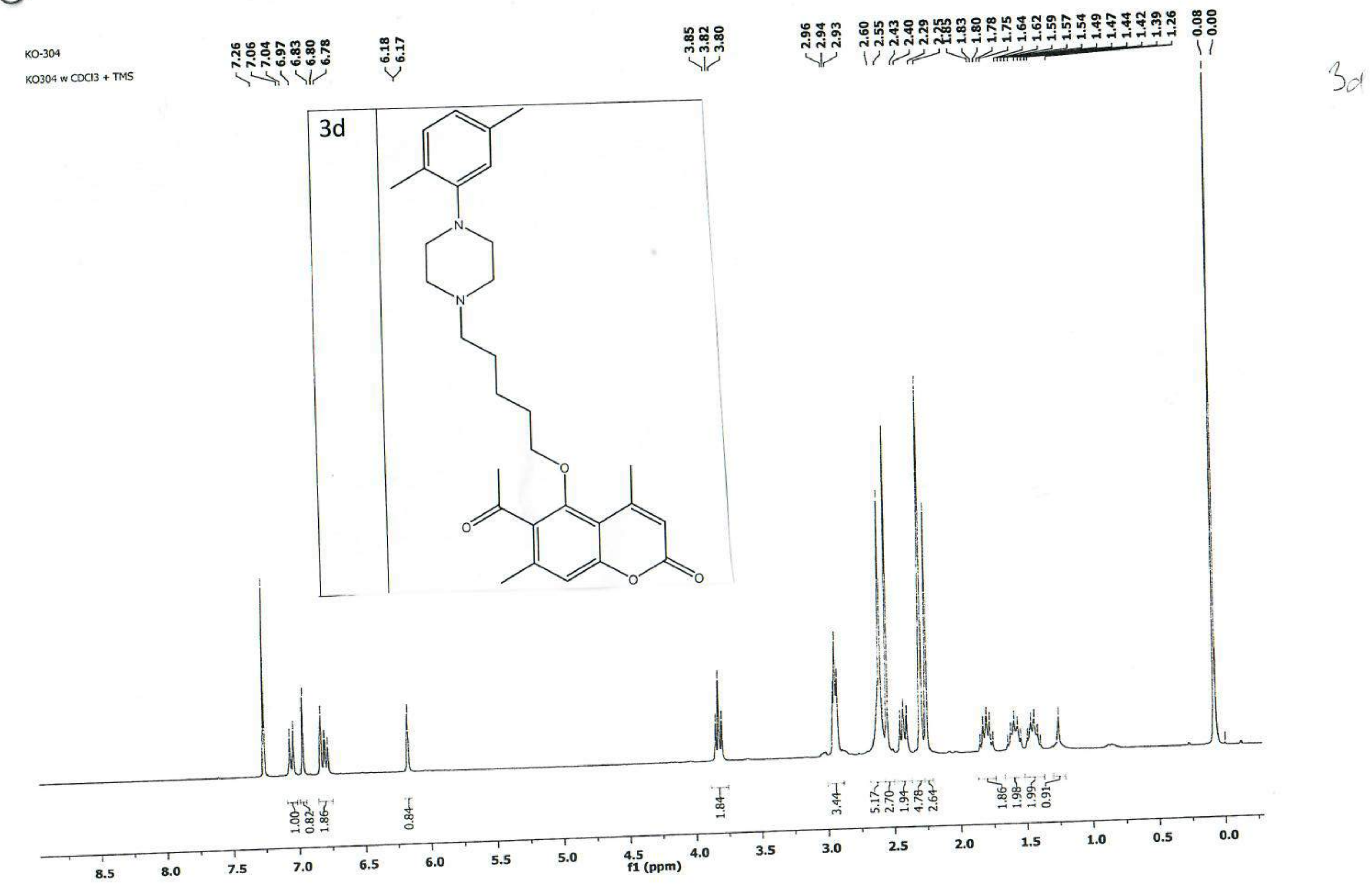
KO-304
KO304 w CDCl3 + TMS
3d
O
N
f1 (ppm)

3d

KO-304-13C-cdcl3

KO 304 13C w CDCl3

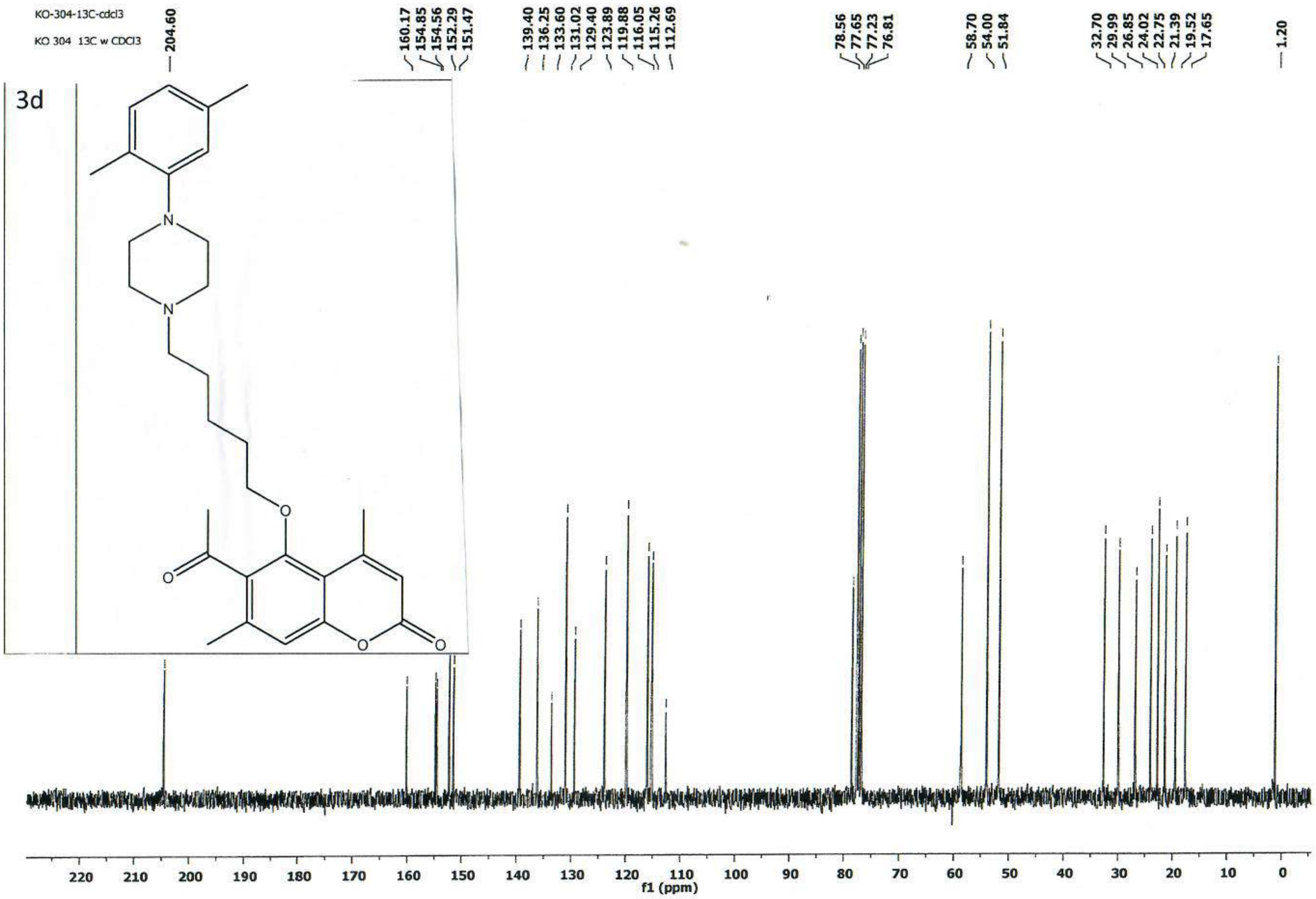

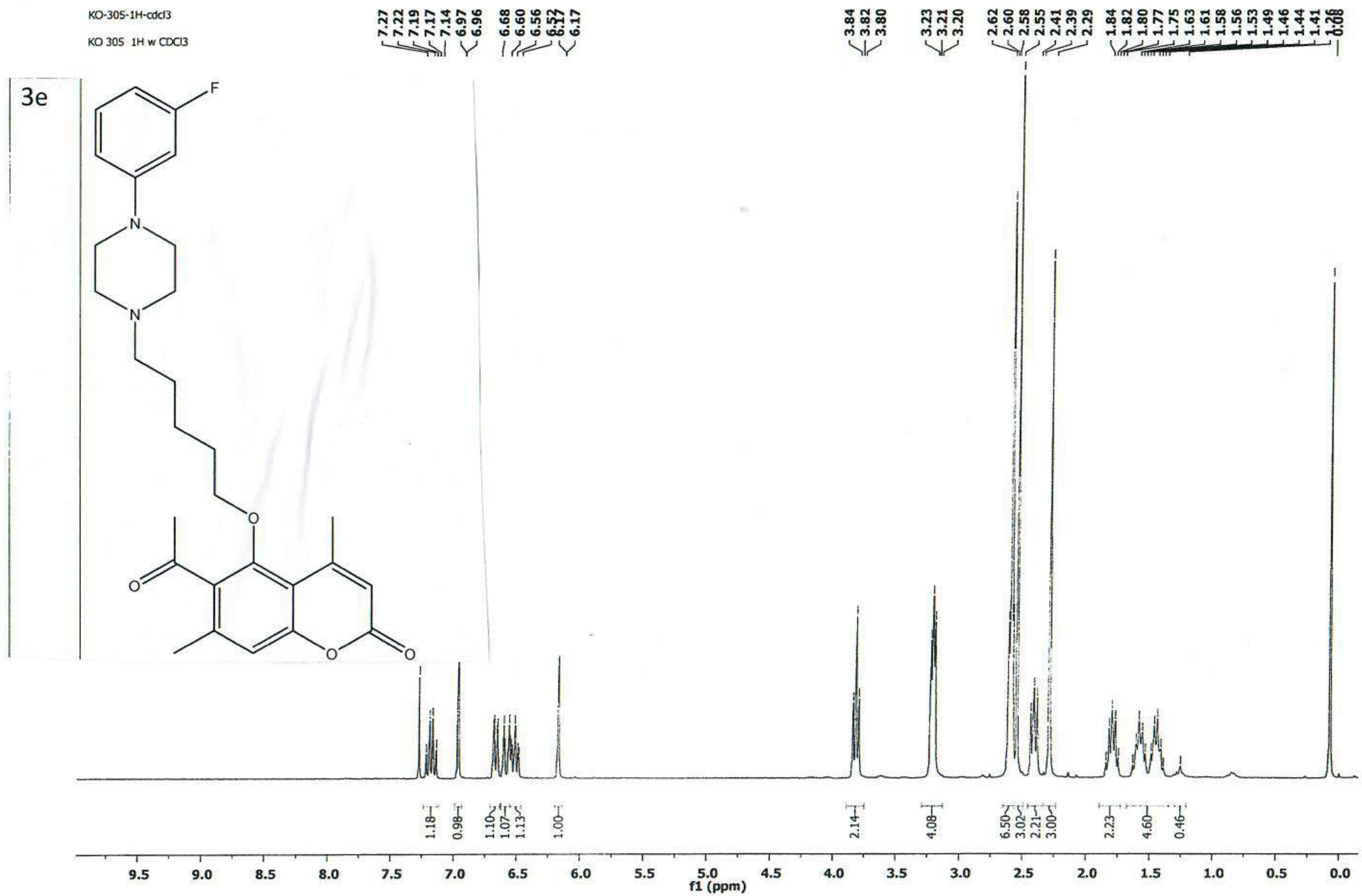
KO-305-1H-cdcl3
KO 305 1H w CDCl3
3e
F
N
N
O
O
O
O
7.27
7.22
7.19
7.17
7.14
6.97
6.96
6.68
6.60
6.56
6.52
6.17
6.17
3.84
3.82
3.80
3.23
3.21
3.20
2.62
2.60
2.58
2.55
2.41
2.39
2.29
1.84
1.82
1.80
1.77
1.75
1.63
1.61
1.58
1.56
1.53
1.49
1.46
1.44
1.41
1.26
0.08
1.18
0.98
1.10
1.07
1.13
1.00
2.14
4.08
6.50
3.02
2.21
3.00
2.23
4.60
0.46
9.5
9.0
8.5
8.0
7.5
7.0
6.5
6.0
5.5
5.0
4.5
4.0
3.5
3.0
2.5
2.0
1.5
1.0
0.5
0.0
f1 (ppm)

3e

3e

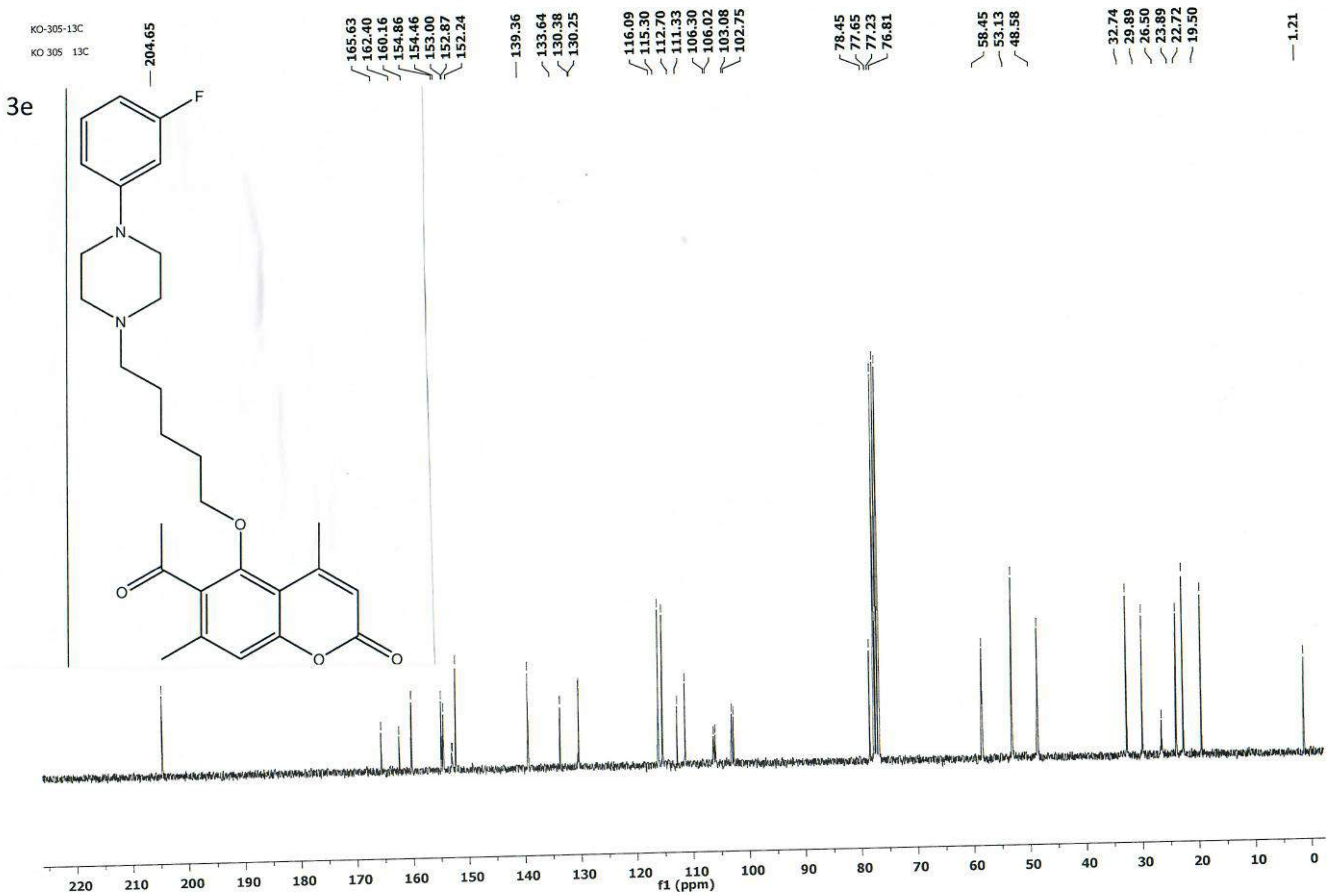
KO-305-13C
KO 305 13C
3e
F
N
N
O
O
O
O
204.65
165.63
162.40
160.16
154.86
154.46
153.00
152.87
152.24
139.36
133.64
130.38
130.25
116.09
115.30
112.70
111.33
106.30
106.02
103.08
102.75
78.45
77.65
77.23
76.81
58.45
53.13
48.58
32.74
29.89
26.50
23.89
22.72
19.50
1.21
220 210 200 190 180 170 160 150 140 130 120 110 100 90 80 70 60 50 40 30 20 10 0
f1 (ppm)

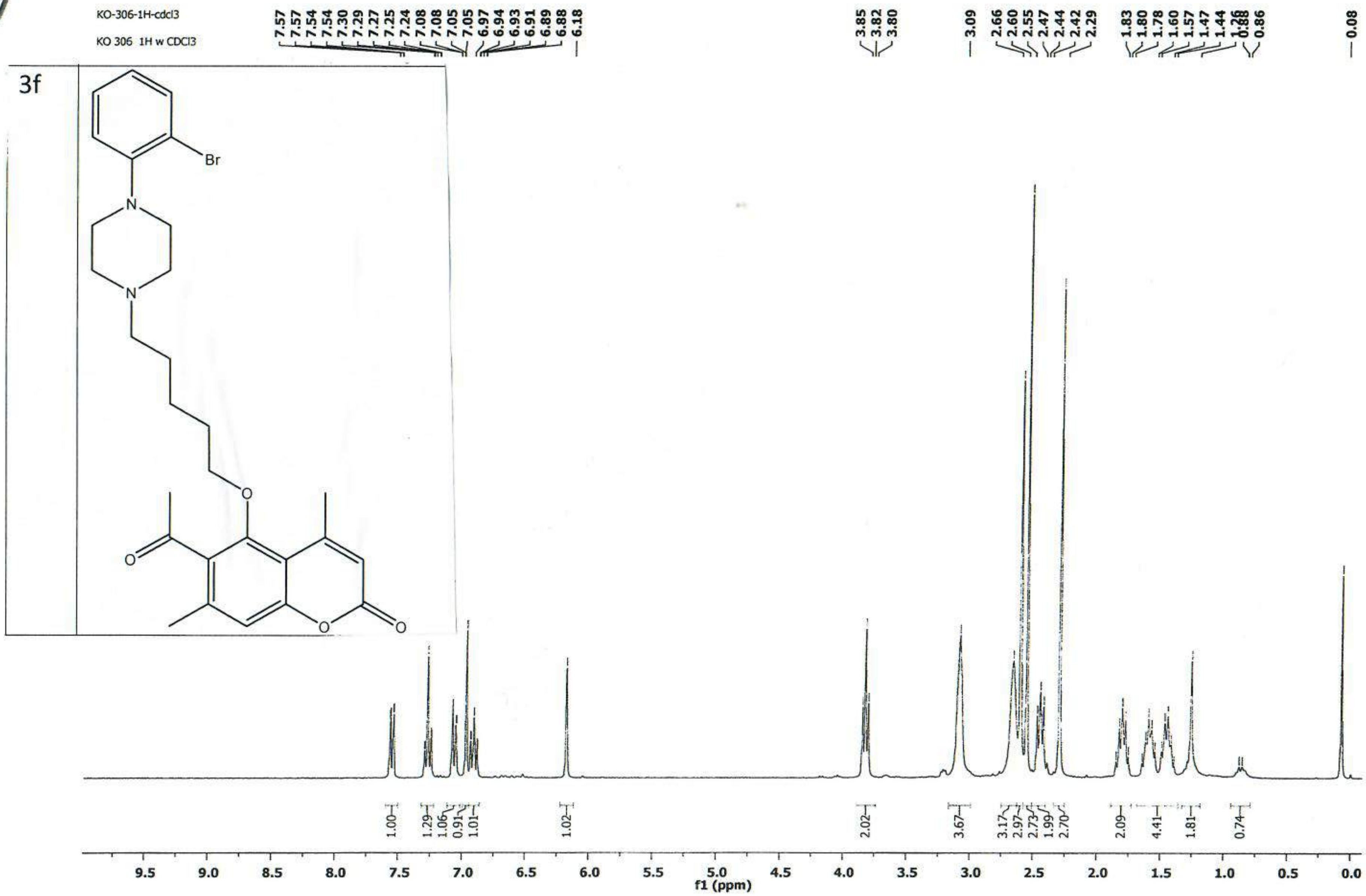

KO-306-1H-cdcl3
KO 306 1H w CDCl3
7.57 7.57 7.54 7.54 7.30 7.29 7.27 7.25 7.24 7.08 7.08 7.05 7.05 6.97 6.94 6.93 6.91 6.89 6.88 6.18
3.85 3.82 3.80
3.09
2.66 2.60 2.55 2.47 2.44 2.42 2.29
1.83 1.80 1.78 1.60 1.57 1.47 1.44 1.26 0.88 0.86
0.08
3f
Br
N
N
O
O
O
O
1.00 1.29 1.06 0.91 1.01 1.02 2.02 3.67 3.17 2.97 2.73 1.99 2.70 2.09 4.41 1.81 0.74
9.5 9.0 8.5 8.0 7.5 7.0 6.5 6.0 5.5 5.0 4.5 4.0 3.5 3.0 2.5 2.0 1.5 1.0 0.5 0.0
f1 (ppm)

3f

3f

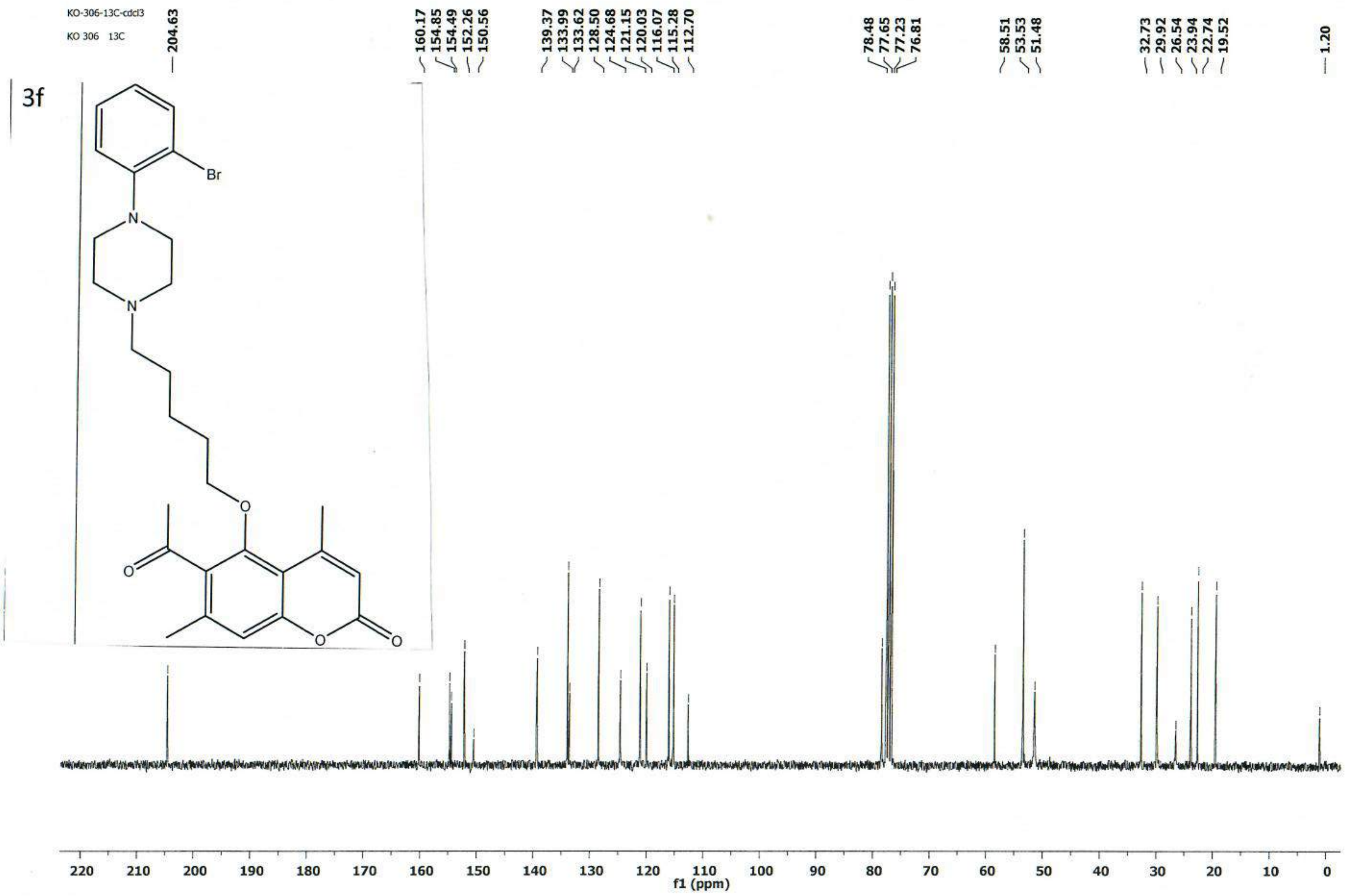
KO-306-13C-cdcl3
KO 306 13C
204.63
160.17
154.85
154.49
152.26
150.56
139.37
133.99
133.62
128.50
124.68
121.15
120.03
116.07
115.28
112.70
78.48
77.65
77.23
76.81
58.51
53.53
51.48
32.73
29.92
26.54
23.94
22.74
19.52
1.20
3f
Br
N
N
O
O
O
O
220 210 200 190 180 170 160 150 140 130 120 110 100 90 80 70 60 50 40 30 20 10 0
f1 (ppm)

3g

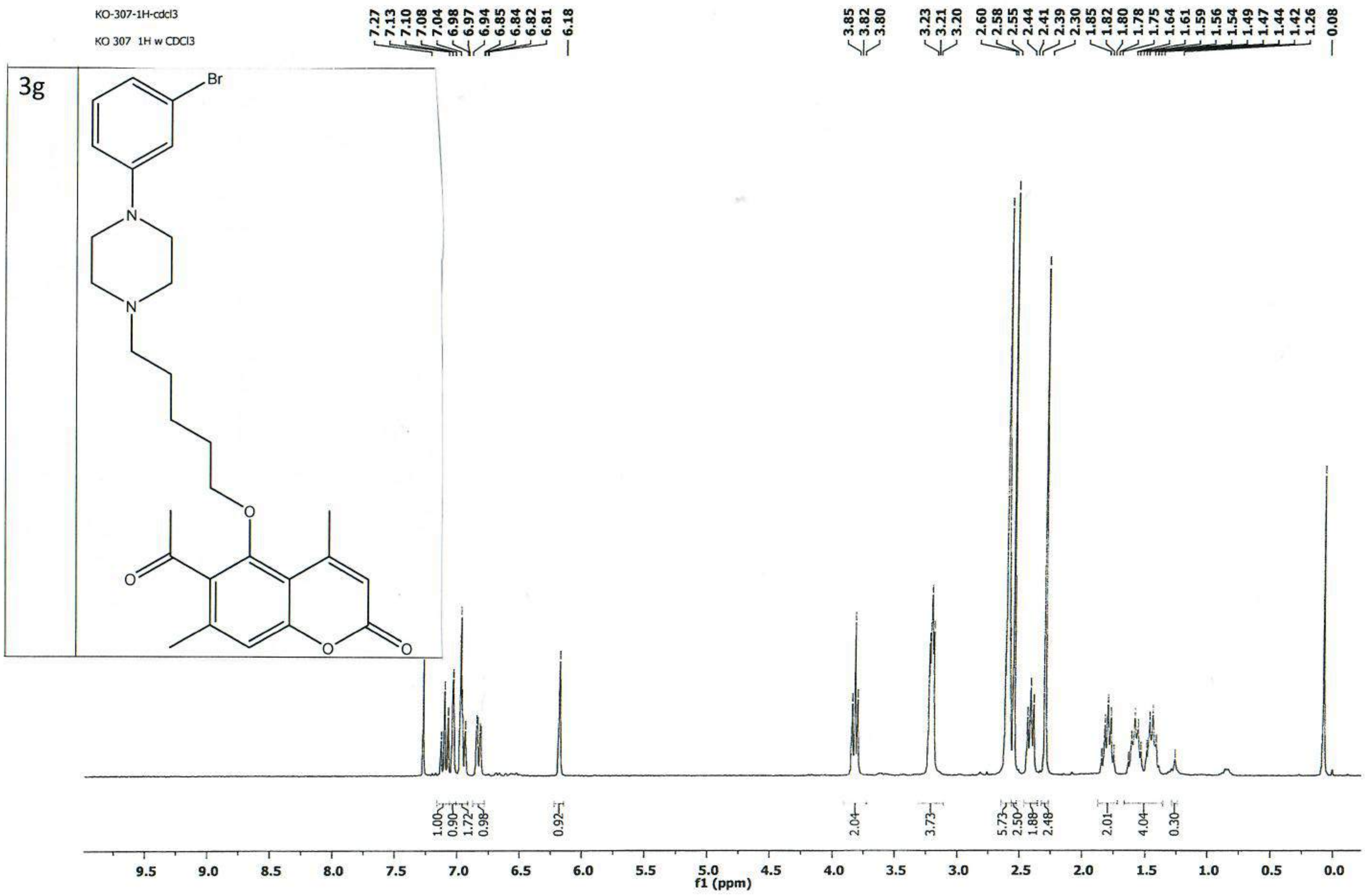
KO-307-1H-cdcl3
KO 307 1H w CDCl3
3g
Br
N
N
O
O
O
O
7.27
7.13
7.10
7.08
7.04
6.98
6.97
6.94
6.85
6.84
6.82
6.81
6.18
3.85
3.82
3.80
3.23
3.21
3.20
2.60
2.58
2.55
2.44
2.41
2.39
2.30
1.85
1.82
1.80
1.78
1.75
1.64
1.61
1.59
1.56
1.54
1.49
1.47
1.44
1.42
1.26
0.08
1.00
0.90
1.72
0.98
0.92
2.04
3.73
5.73
2.50
1.88
2.48
2.01
4.04
0.30
9.5
9.0
8.5
8.0
7.5
7.0
6.5
6.0
5.5
5.0
4.5
4.0
3.5
3.0
2.5
2.0
1.5
1.0
0.5
0.0
f1 (ppm)

3g

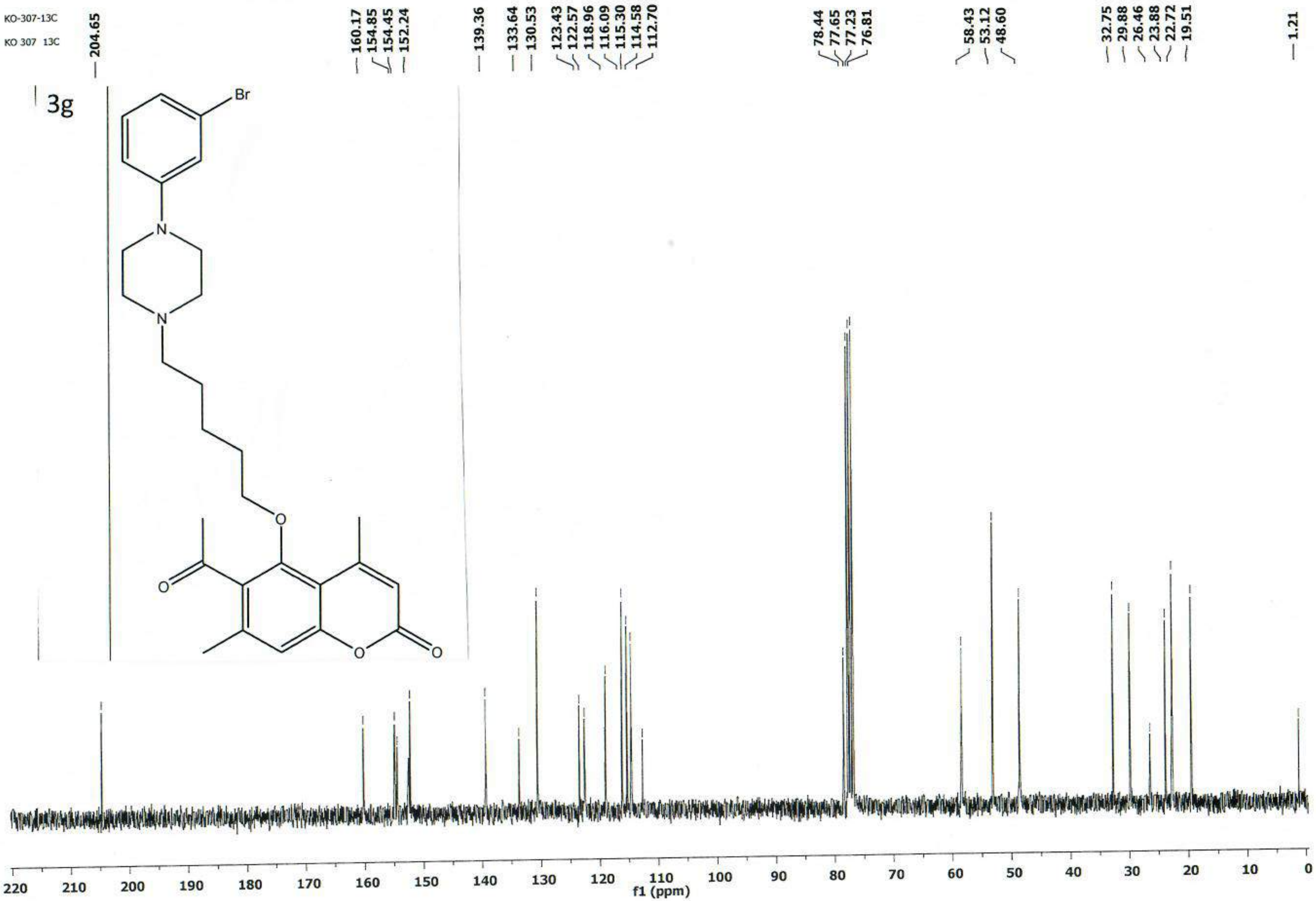

KO-307-13C
KO 307 13C
204.65
160.17
154.85
154.45
152.24
139.36
133.64
130.53
123.43
122.57
118.96
116.09
115.30
114.58
112.70
78.44
77.65
77.23
76.81
58.43
53.12
48.60
32.75
29.88
26.46
23.88
22.72
19.51
1.21
3g
Br
N
N
O
O
O
O
220
210
200
190
180
170
160
150
140
130
120
110
100
90
80
70
60
50
40
30
20
10
0
f1 (ppm)

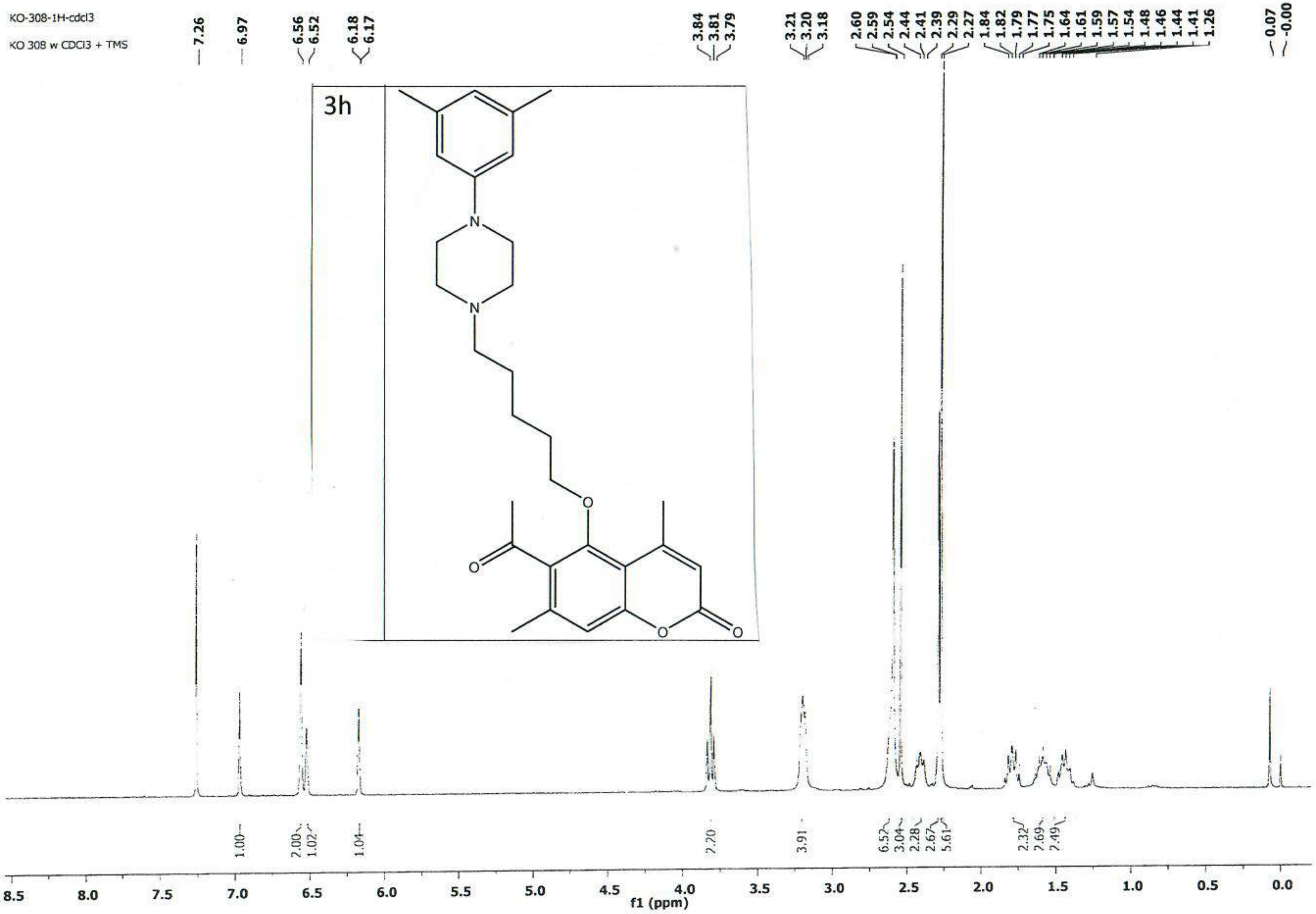
KO-308-1H-cdcl3
KO 308 w CDCl3 + TMS
7.26
6.97
6.56
6.52
6.18
6.17
3.84
3.81
3.79
3.21
3.20
3.18
2.60
2.59
2.54
2.44
2.41
2.39
2.29
2.27
1.84
1.82
1.79
1.77
1.75
1.64
1.61
1.59
1.57
1.54
1.48
1.46
1.44
1.41
1.26
0.07
-0.00
3h
N
N
O
O
O
O
1.00
2.00
1.02
1.04
2.20
3.91
6.52
3.04
2.28
2.67
5.61
2.32
2.69
2.49
8.5
8.0
7.5
7.0
6.5
6.0
5.5
5.0
4.5
4.0
3.5
3.0
2.5
2.0
1.5
1.0
0.5
0.0
f1 (ppm)

3h

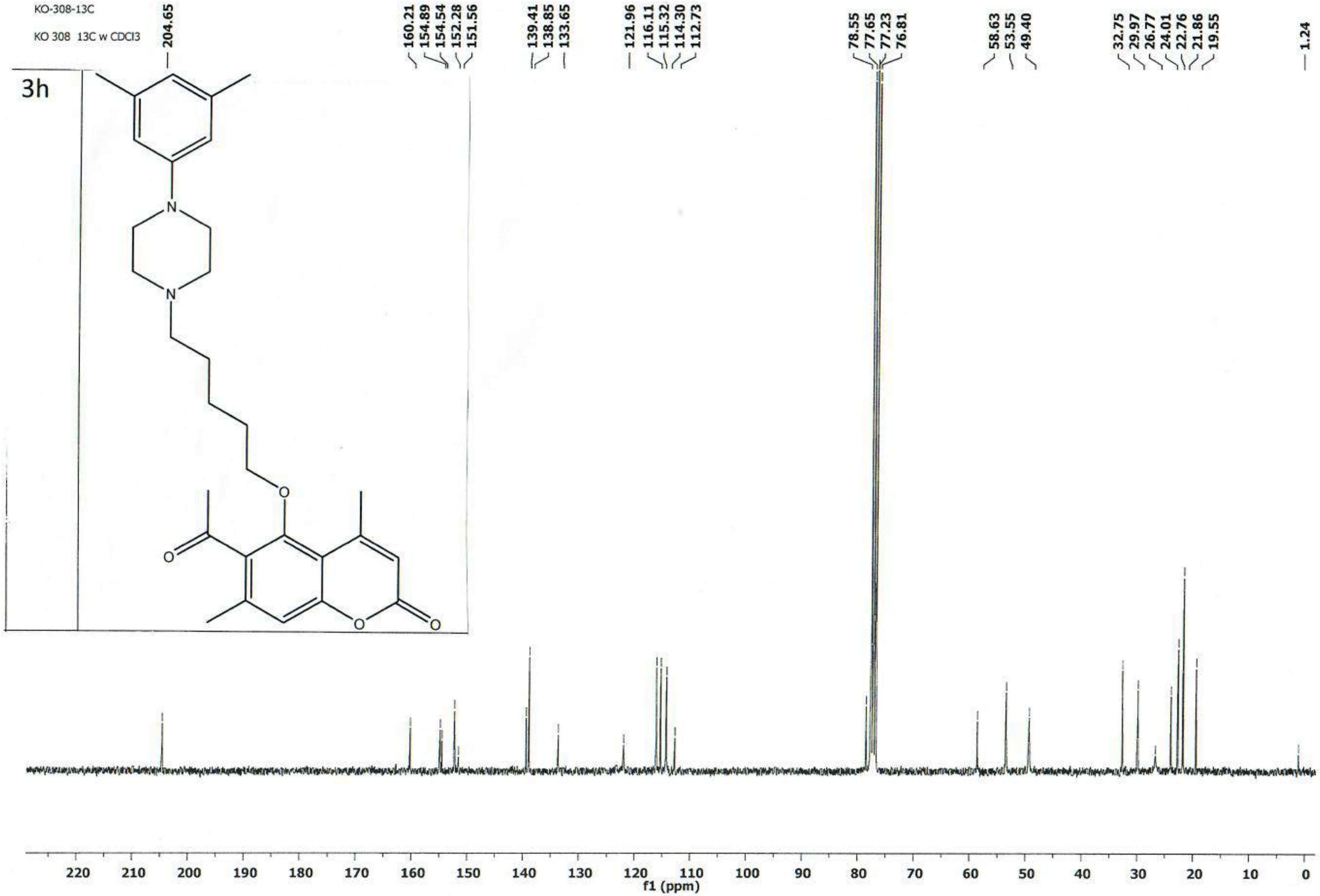
KO-308-13C
KO 308 13C w CDCl3
3h
204.65
160.21
154.89
154.54
152.28
151.56
139.41
138.85
133.65
121.96
116.11
115.32
114.30
112.73
78.55
77.65
77.23
76.81
58.63
53.55
49.40
32.75
29.97
26.77
24.01
22.76
21.86
19.55
1.24
220 210 200 190 180 170 160 150 140 130 120 110 100 90 80 70 60 50 40 30 20 10 0
f1 (ppm)

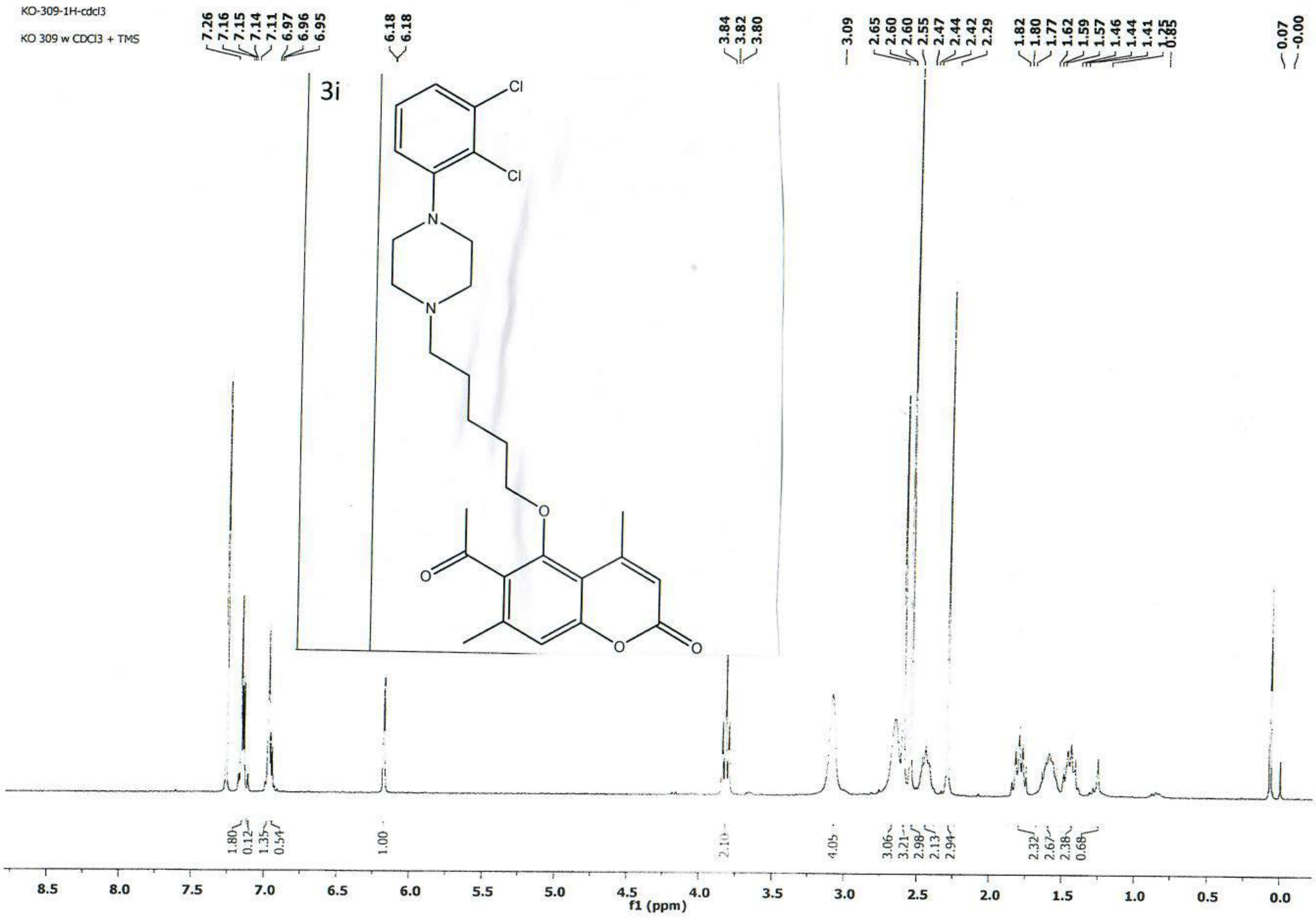
KO-309-1H-cdcl3
KO 309 w CDCl3 + TMS
7.26 7.16 7.15 7.14 7.11 6.97 6.96 6.95
6.18 6.18
3.84 3.82 3.80
3.09
2.65 2.60 2.60 2.55 2.47 2.44 2.42 2.29
1.82 1.80 1.77 1.62 1.59 1.57 1.46 1.44 1.41 1.25 0.85
0.07 -0.00
3i
Cl
Cl
N
N
O
O
O
O
1.80 0.12 1.35 0.54
1.00
2.10
4.05
3.06 3.21 2.98 2.13 2.94
2.32 2.67 2.38 0.68
8.5 8.0 7.5 7.0 6.5 6.0 5.5 5.0 4.5 4.0 3.5 3.0 2.5 2.0 1.5 1.0 0.5 0.0
f1 (ppm)

3i

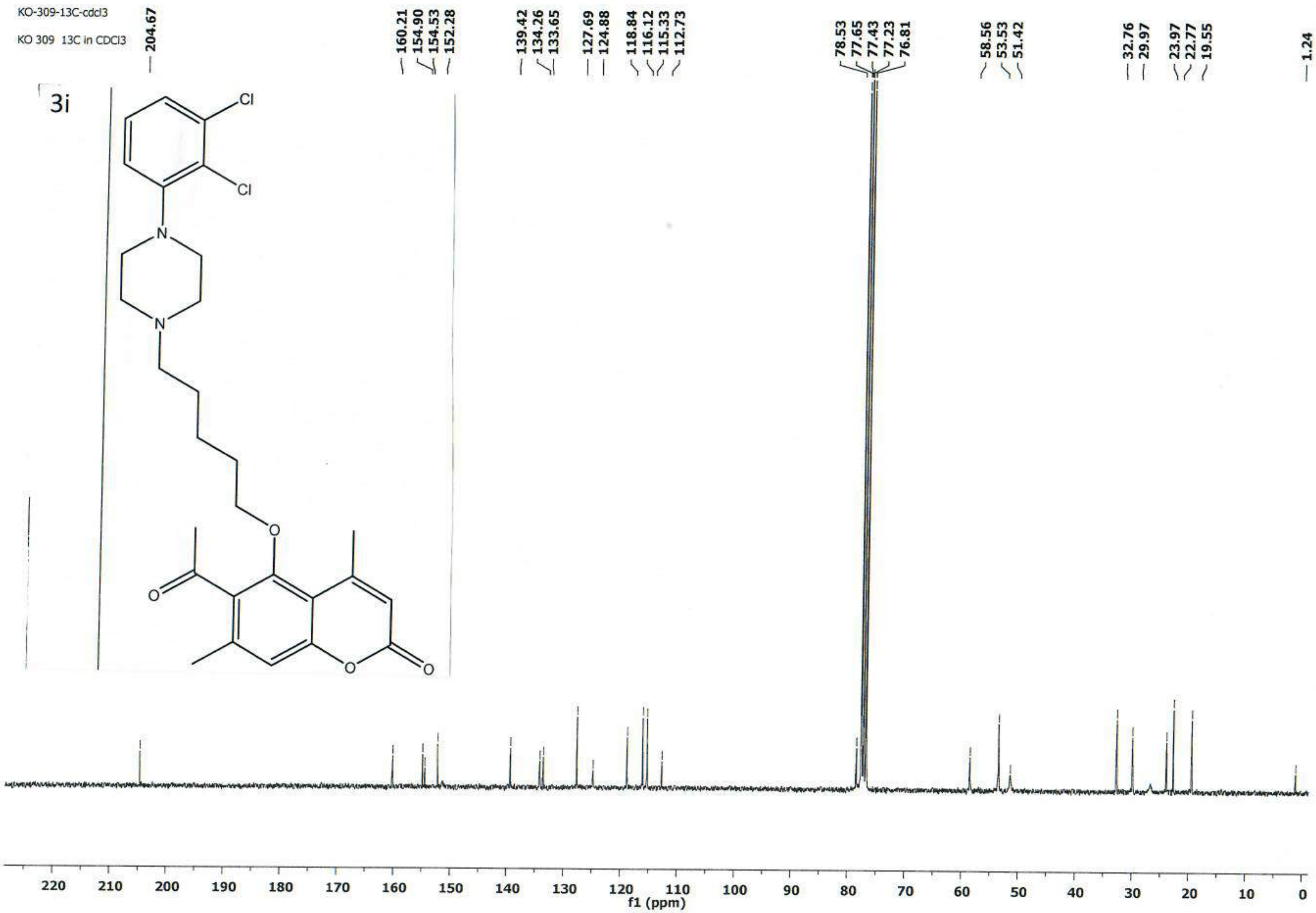

KO-309-13C-cdcl3
KO 309 13C in CDCl3
204.67
160.21
154.90
154.53
152.28
139.42
134.26
133.65
127.69
124.88
118.84
116.12
115.33
112.73
78.53
77.65
77.43
77.23
76.81
58.56
53.53
51.42
32.76
29.97
23.97
22.77
19.55
1.24
3i
Cl
Cl
N
N
O
O
O
O
220 210 200 190 180 170 160 150 140 130 120 110 100 90 80 70 60 50 40 30 20 10 0
f1 (ppm)

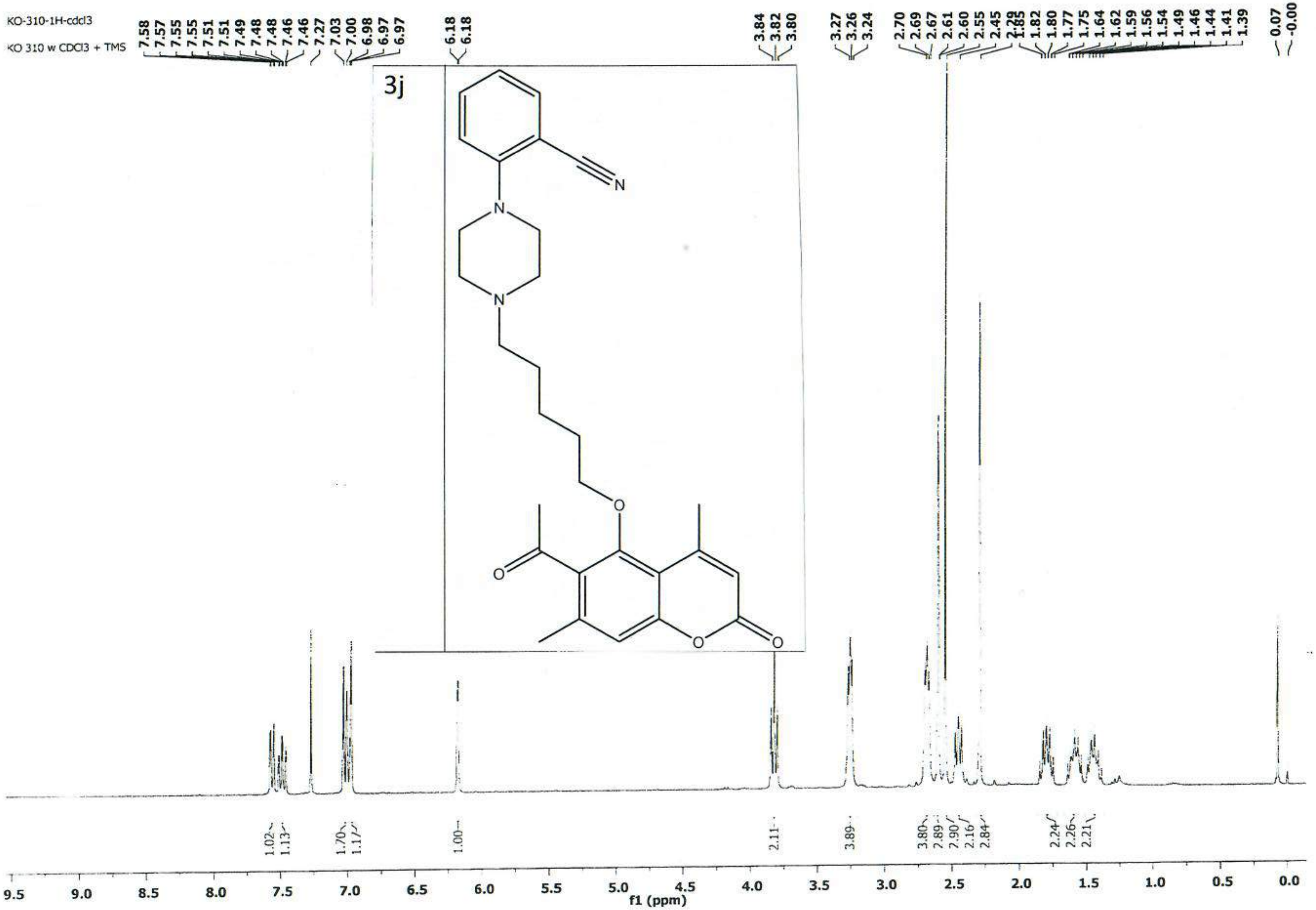

KO-310-1H-cdcl3
KO 310 w CDCl3 + TMS
7.58 7.57 7.55 7.55 7.51 7.49 7.48 7.48 7.46 7.46 7.27 7.03 7.00 6.98 6.97 6.97
6.18 6.18
3.84 3.82 3.80
3.27 3.26 3.24
2.70 2.69 2.67 2.61 2.60 2.55 2.45 2.29 1.85 1.82 1.80 1.77 1.75 1.64 1.62 1.59 1.56 1.54 1.49 1.46 1.44 1.41 1.39
0.07 -0.00
3j
N
O
1.02 1.13 1.70 1.17 1.00 2.11 3.89 3.80 2.89 7.90 2.16 2.84 2.24 2.26 2.21
9.5 9.0 8.5 8.0 7.5 7.0 6.5 6.0 5.5 5.0 4.5 4.0 3.5 3.0 2.5 2.0 1.5 1.0 0.5 0.0
f1 (ppm)

3j

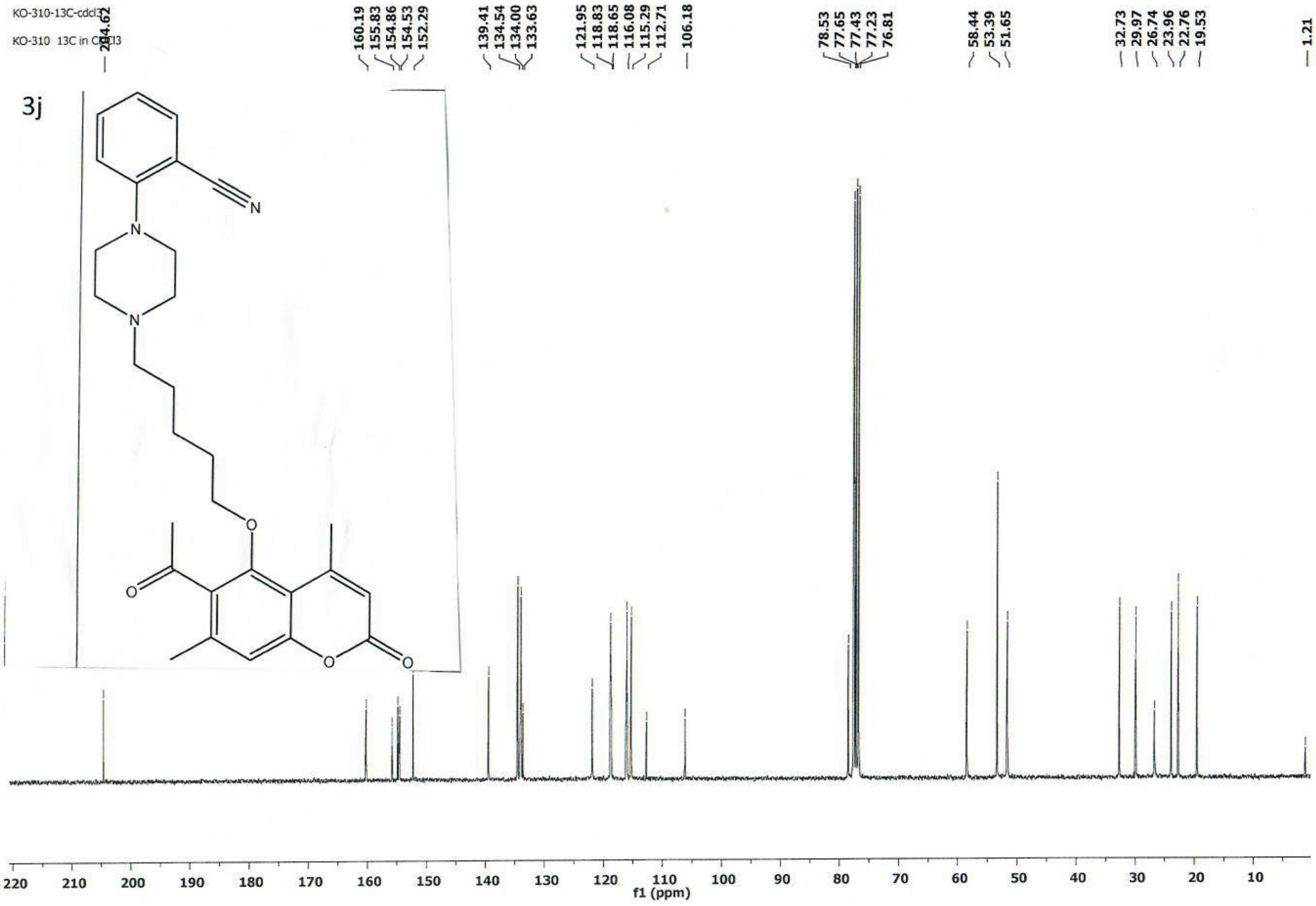
KO-310-13C-cdcl3
KO-310 13C in CDCl3
204.62
160.19
155.83
154.86
154.53
152.29
139.41
134.54
134.00
133.63
121.95
118.83
118.65
116.08
115.29
112.71
106.18
78.53
77.65
77.43
77.23
76.81
58.44
53.39
51.65
32.73
29.97
26.74
23.96
22.76
19.53
1.21
3j
N
N
N
O
O
O
O
220 210 200 190 180 170 160 150 140 130 120 110 100 90 80 70 60 50 40 30 20 10
f1 (ppm)

4

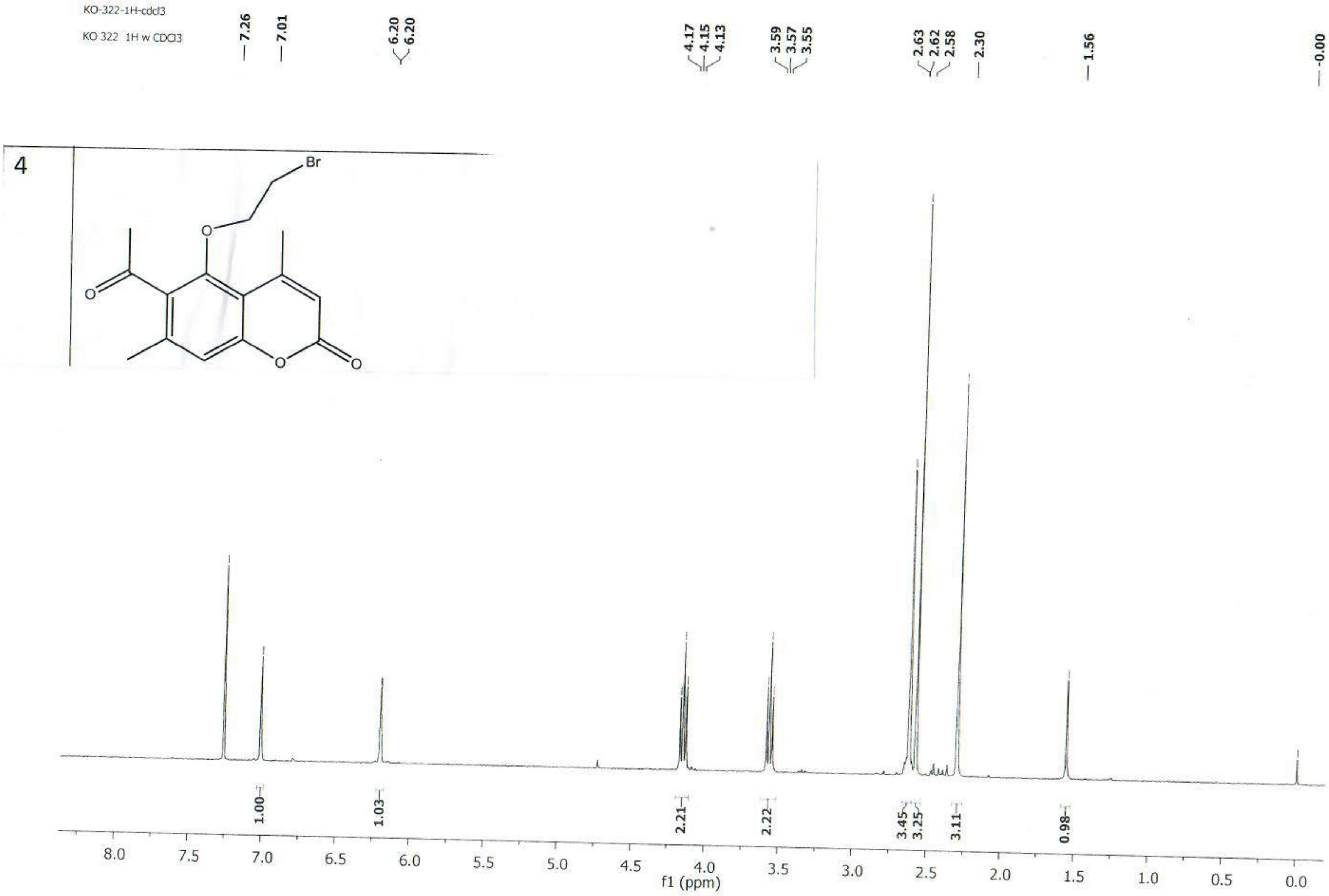
KO-322-1H-cdcl3
KO 322 1H w CDCl3
7.26
7.01
6.20
6.20
4.17
4.15
4.13
3.59
3.57
3.55
2.63
2.62
2.58
2.30
1.56
-0.00
4
Br
O
O
O
O
1.00
1.03
2.21
2.22
3.45
3.25
3.11
0.98
8.0
7.5
7.0
6.5
6.0
5.5
5.0
4.5
4.0
3.5
3.0
2.5
2.0
1.5
1.0
0.5
0.0
f1 (ppm)

4

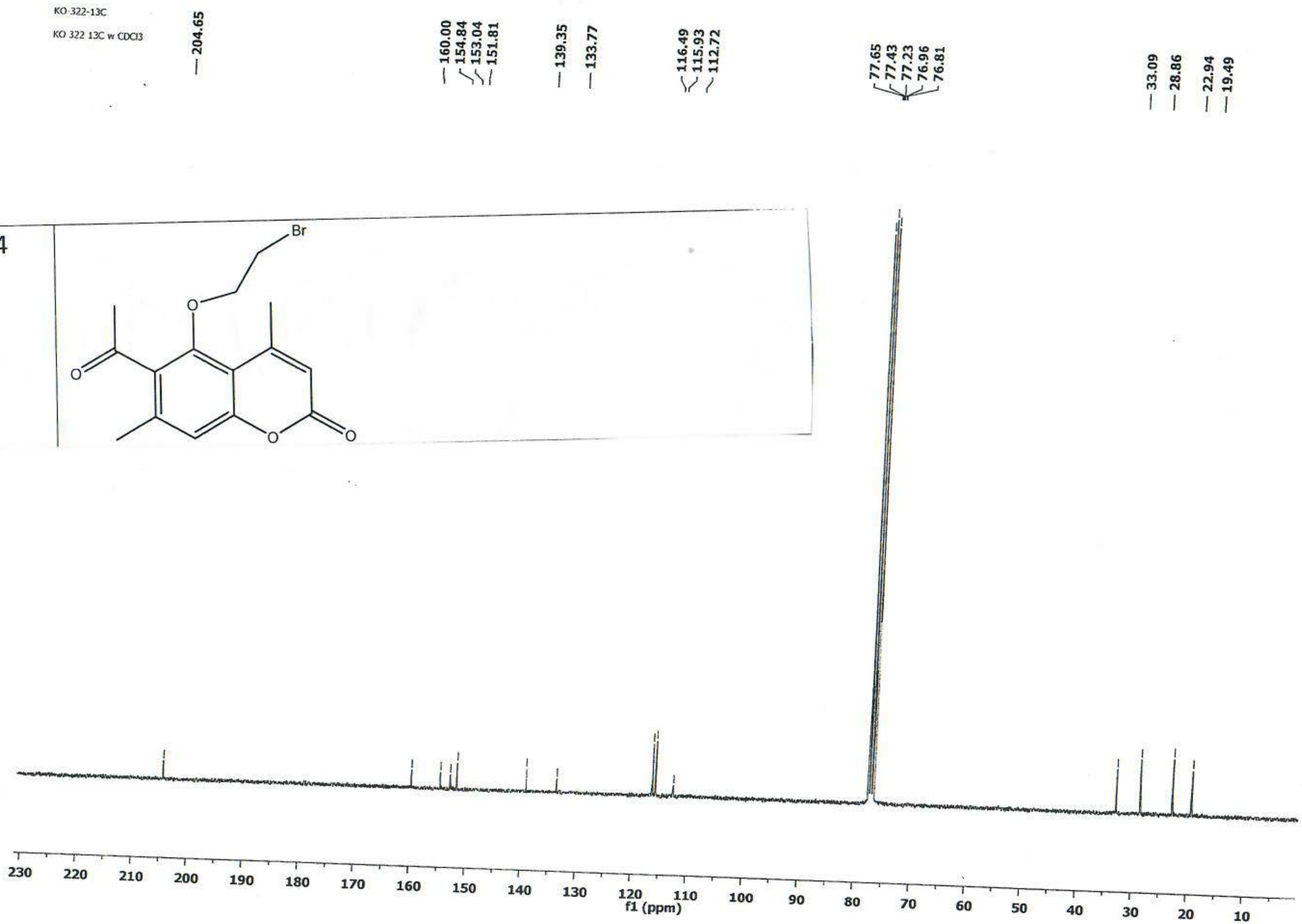
KO-322-13C
KO 322 13C w CDCl3
204.65
160.00
154.84
153.04
151.81
139.35
133.77
116.49
115.93
112.72
77.65
77.43
77.23
76.96
76.81
33.09
28.86
22.94
19.49
Br
O
O
O
O
230 220 210 200 190 180 170 160 150 140 130 120 110 100 90 80 70 60 50 40 30 20 10
f1 (ppm)

4a

KO-323-1h

KO 323 1H

4a

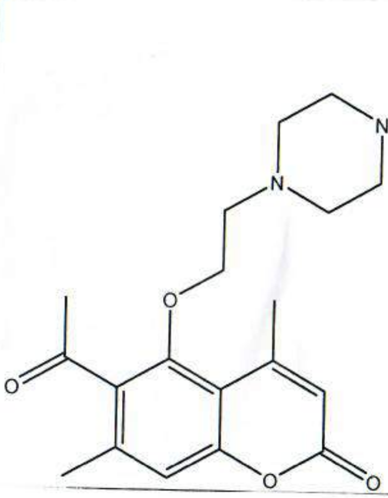

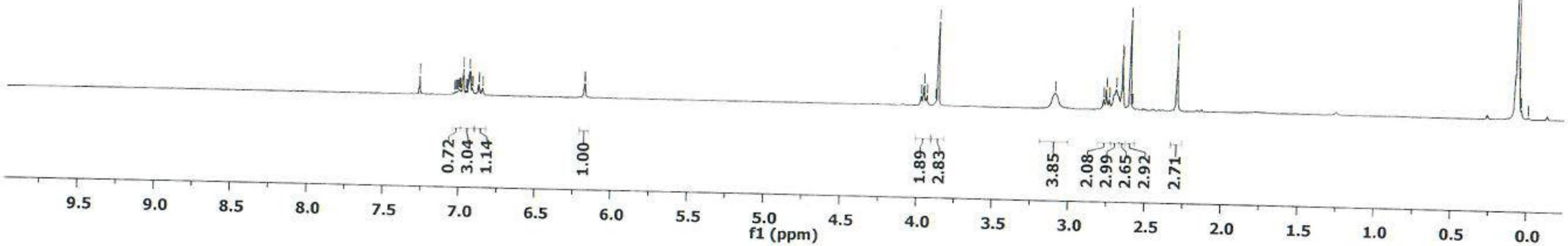

4a

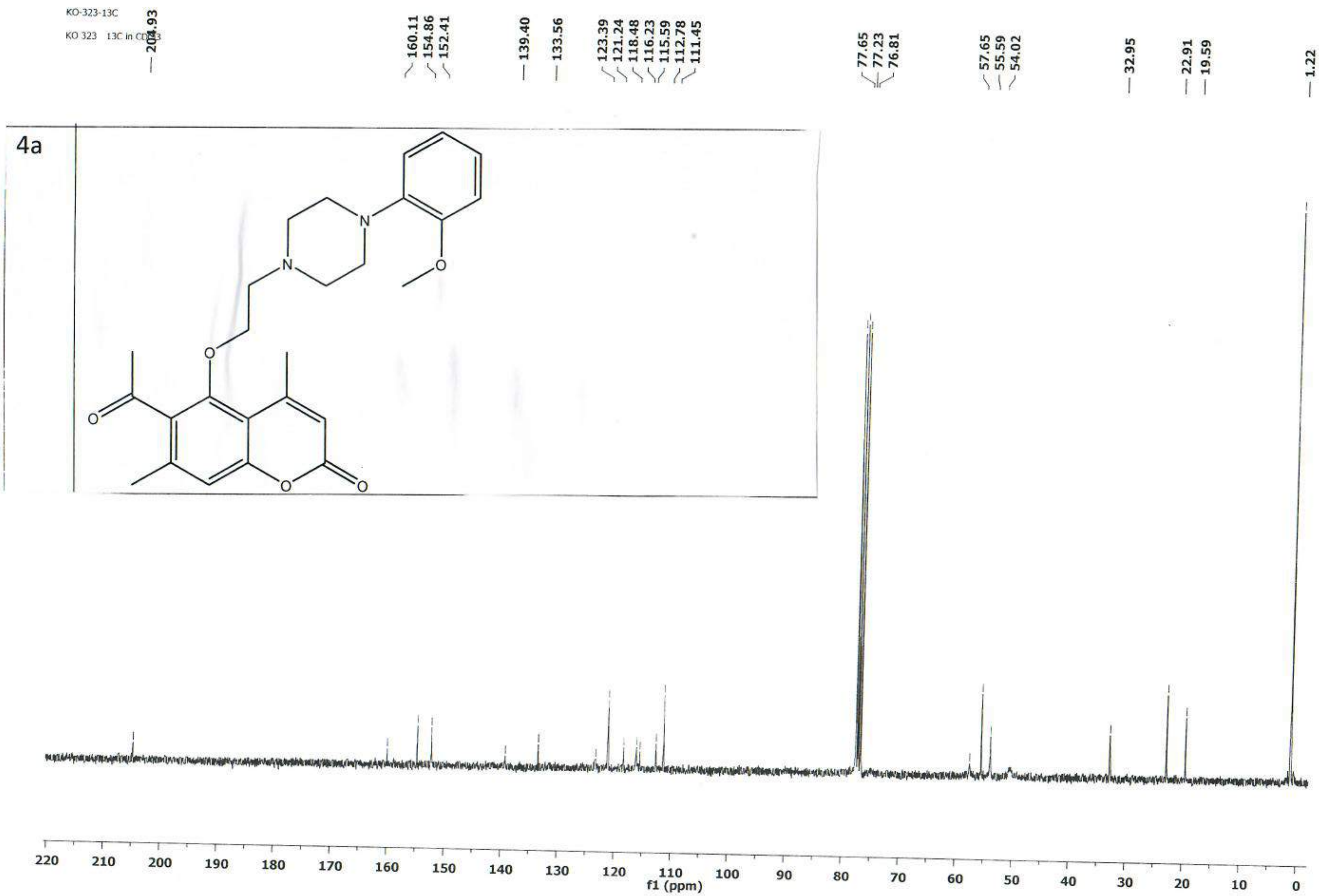

KO-323-13C
KO 323 13C in CDCl3
204.93
160.11
154.86
152.41
139.40
133.56
123.39
121.24
118.48
116.23
115.59
112.78
111.45
77.65
77.23
76.81
57.65
55.59
54.02
32.95
22.91
19.59
1.22
4a
220 210 200 190 180 170 160 150 140 130 120 110 100 90 80 70 60 50 40 30 20 10 0
f1 (ppm)

4b

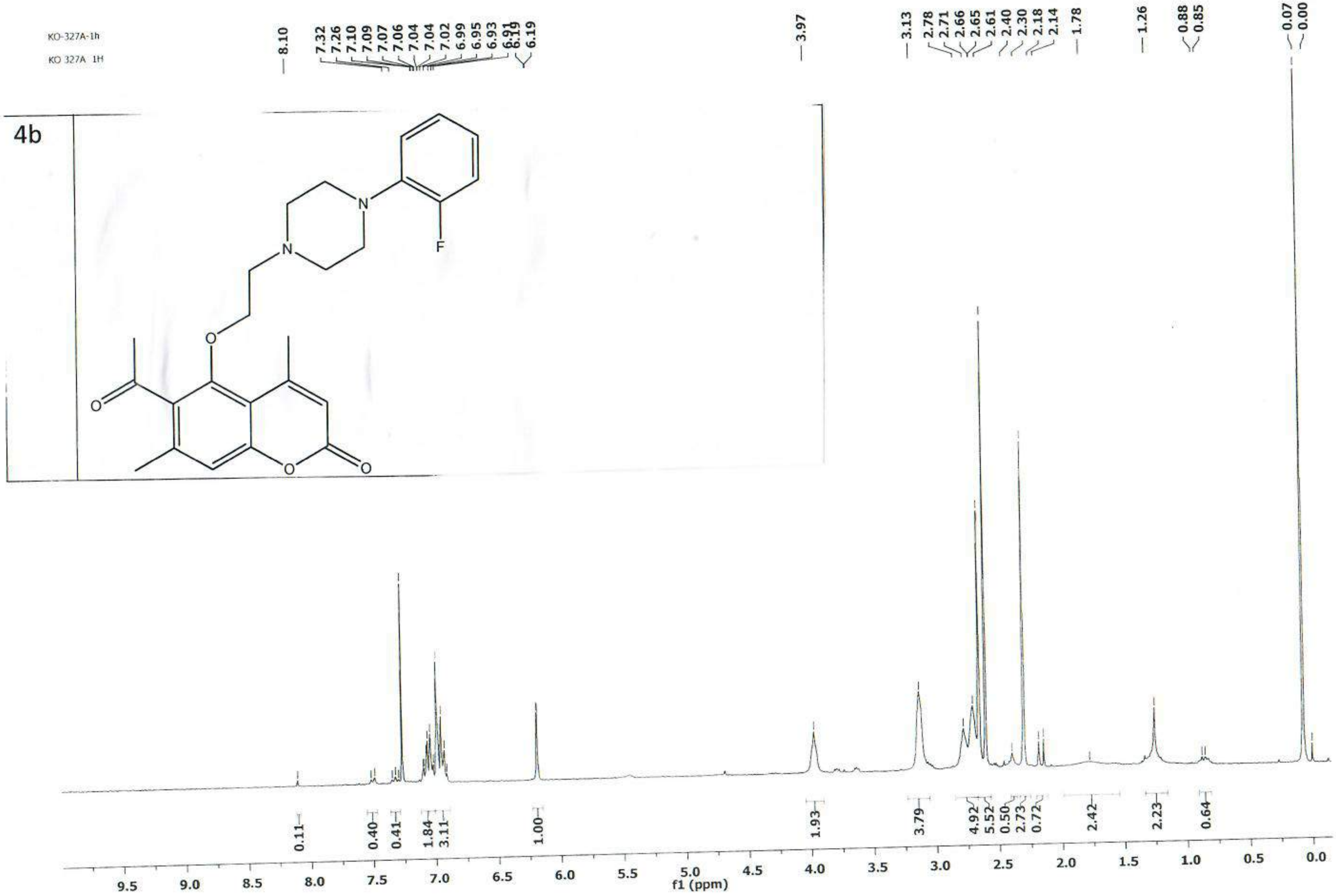
KO-327A-1h
KO 327A 1H
8.10
7.32 7.26 7.10 7.09 7.07 7.06 7.04 7.04 7.02 6.99 6.95 6.93 6.91 6.19 6.19
3.97
3.13 2.78 2.71 2.66 2.65 2.61 2.40 2.30 2.18 2.14 1.78
1.26
0.88 0.85
0.07 0.00
4b
O
N
N
F
O
O
0.11 0.40 0.41 1.84 3.11 1.00 1.93 3.79 4.92 5.52 0.50 2.73 0.72 2.42 2.23 0.64
9.5 9.0 8.5 8.0 7.5 7.0 6.5 6.0 5.5 5.0 4.5 4.0 3.5 3.0 2.5 2.0 1.5 1.0 0.5 0.0
f1 (ppm)

4b

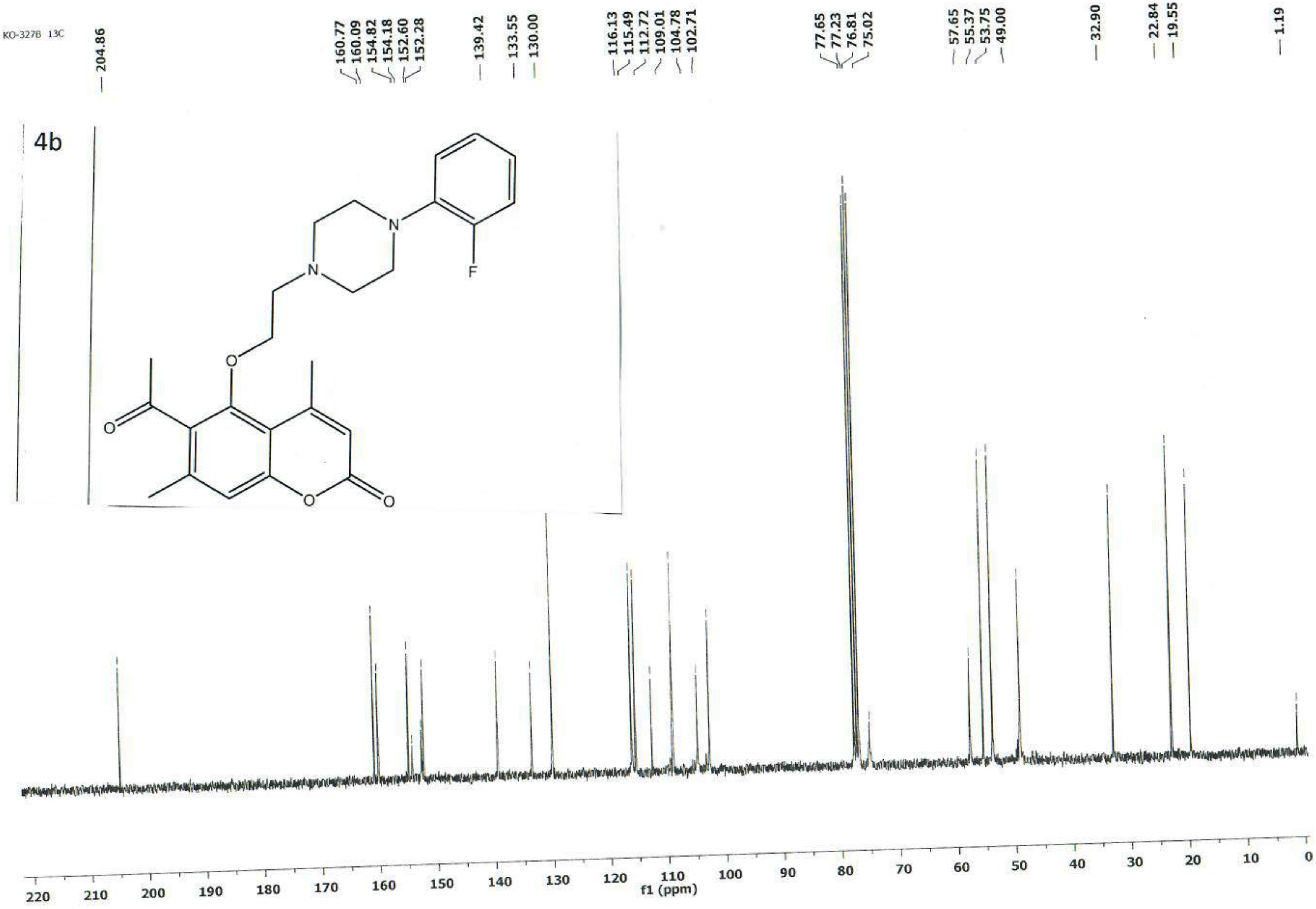

KO-327B 13C
204.86
160.77
160.09
154.82
154.18
152.60
152.28
139.42
133.55
130.00
116.13
115.49
112.72
109.01
104.78
102.71
77.65
77.23
76.81
75.02
57.65
55.37
53.75
49.00
32.90
22.84
19.55
1.19
4b
N
N
F
O
O
O
O
220
210
200
190
180
170
160
150
140
130
120
110
100
90
80
70
60
50
40
30
20
10
0
f1 (ppm)

4c

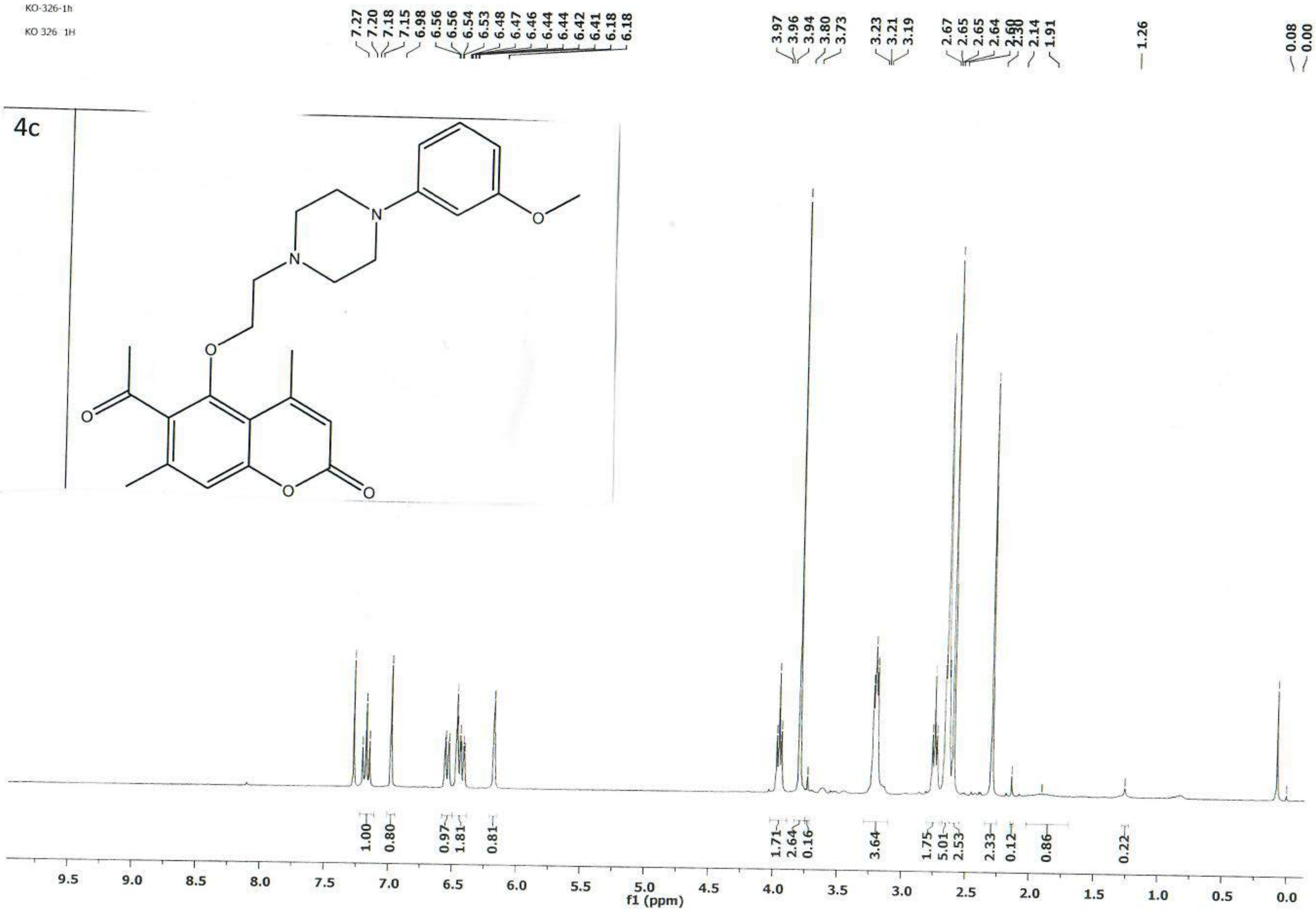
KO-326-1h
KO 326 1H
7.27
7.20
7.18
7.15
6.98
6.56
6.56
6.54
6.53
6.48
6.47
6.46
6.44
6.44
6.42
6.41
6.18
6.18
3.97
3.96
3.94
3.80
3.73
3.23
3.21
3.19
2.67
2.65
2.65
2.64
2.60
2.30
2.14
1.91
1.26
0.08
0.00
4c
N
N
O
O
O
O
O
1.00
0.80
0.97
1.81
0.81
1.71
2.64
0.16
3.64
1.75
5.01
2.53
2.33
0.12
0.86
0.22
9.5
9.0
8.5
8.0
7.5
7.0
6.5
6.0
5.5
5.0
4.5
4.0
3.5
3.0
2.5
2.0
1.5
1.0
0.5
0.0
f1 (ppm)

4c

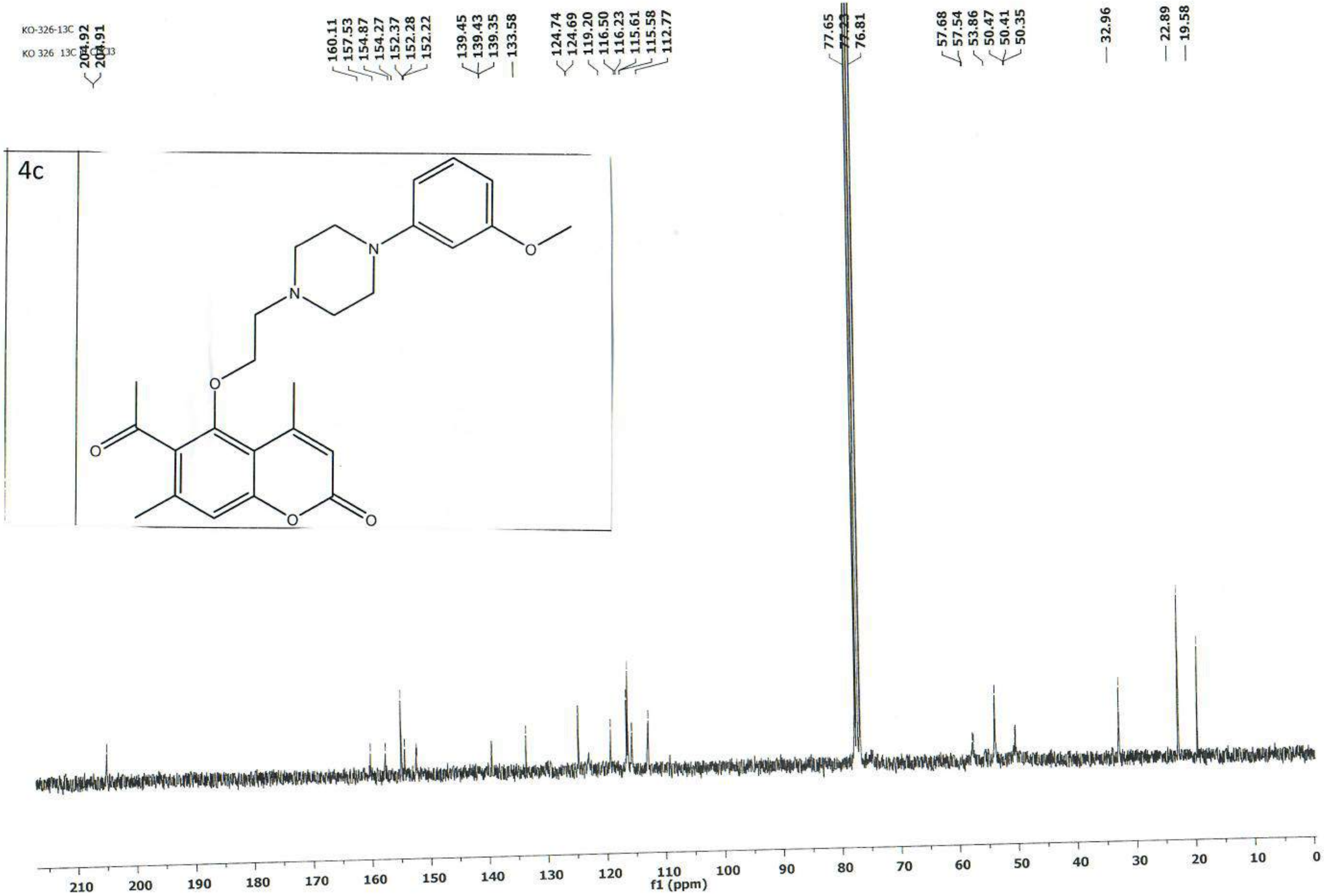
KO-326-13C
KO 326 13C CDCl3
204.92
204.91
160.11
157.53
154.87
154.27
152.37
152.28
152.22
139.45
139.43
139.35
133.58
124.74
124.69
119.20
116.50
116.23
115.61
115.58
112.77
77.65
77.23
76.81
57.68
57.54
53.86
50.47
50.41
50.35
32.96
22.89
19.58
4c
N
N
O
O
O
O
O
210
200
190
180
170
160
150
140
130
120
110
100
90
80
70
60
50
40
30
20
10
0
f1 (ppm)

4d

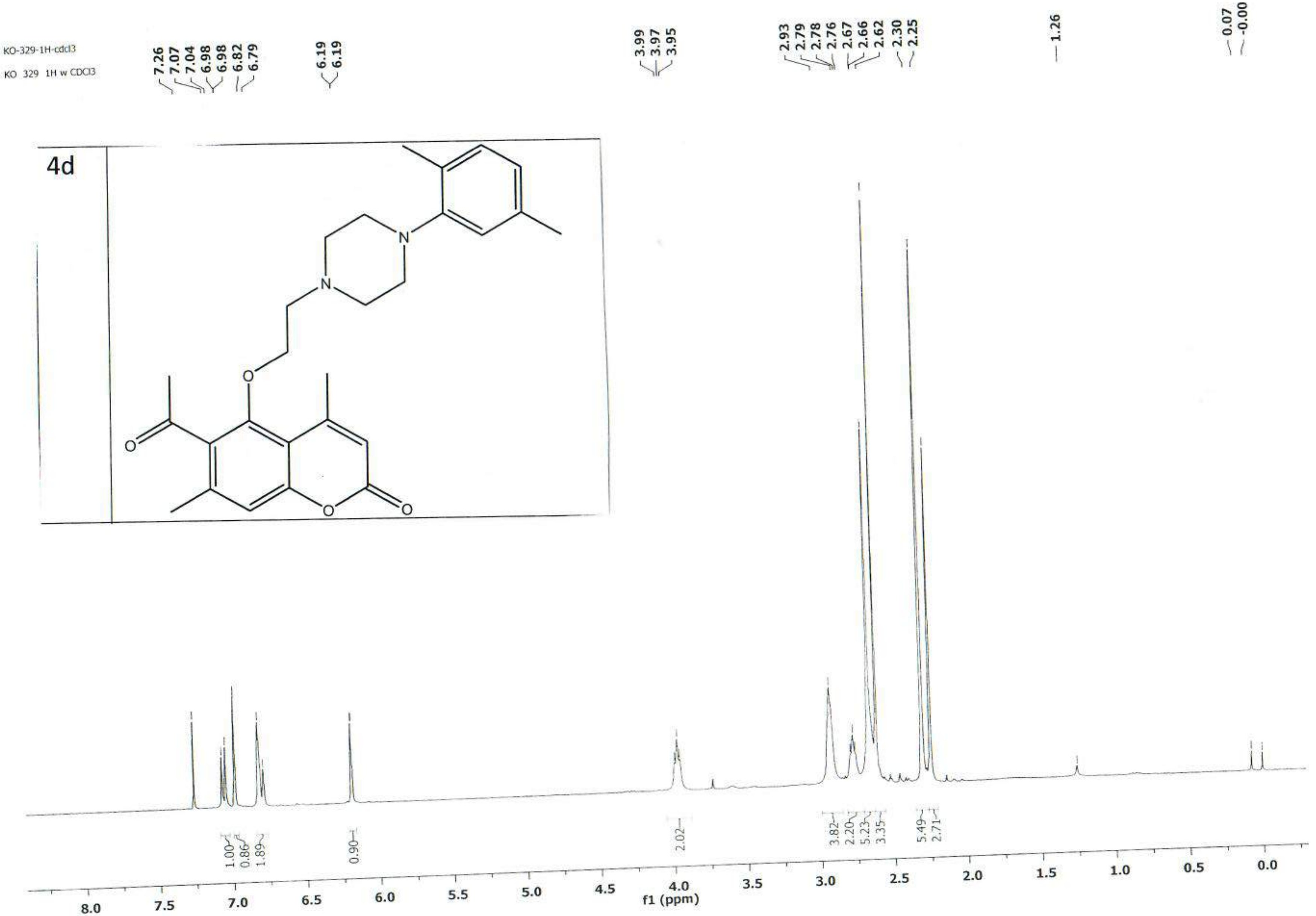

KO-329-1H-cdcl3
KO 329 1H w CDCl3
7.26
7.07
7.04
6.98
6.98
6.82
6.79
6.19
6.19
3.99
3.97
3.95
2.93
2.79
2.78
2.76
2.67
2.66
2.62
2.30
2.25
1.26
0.07
-0.00
4d
N
N
O
O
O
O
O
1.00
0.86
1.89
0.90
2.02
3.82
2.20
5.23
3.35
5.49
2.71
8.0
7.5
7.0
6.5
6.0
5.5
5.0
4.5
4.0
3.5
3.0
2.5
2.0
1.5
1.0
0.5
0.0
f1 (ppm)

4d

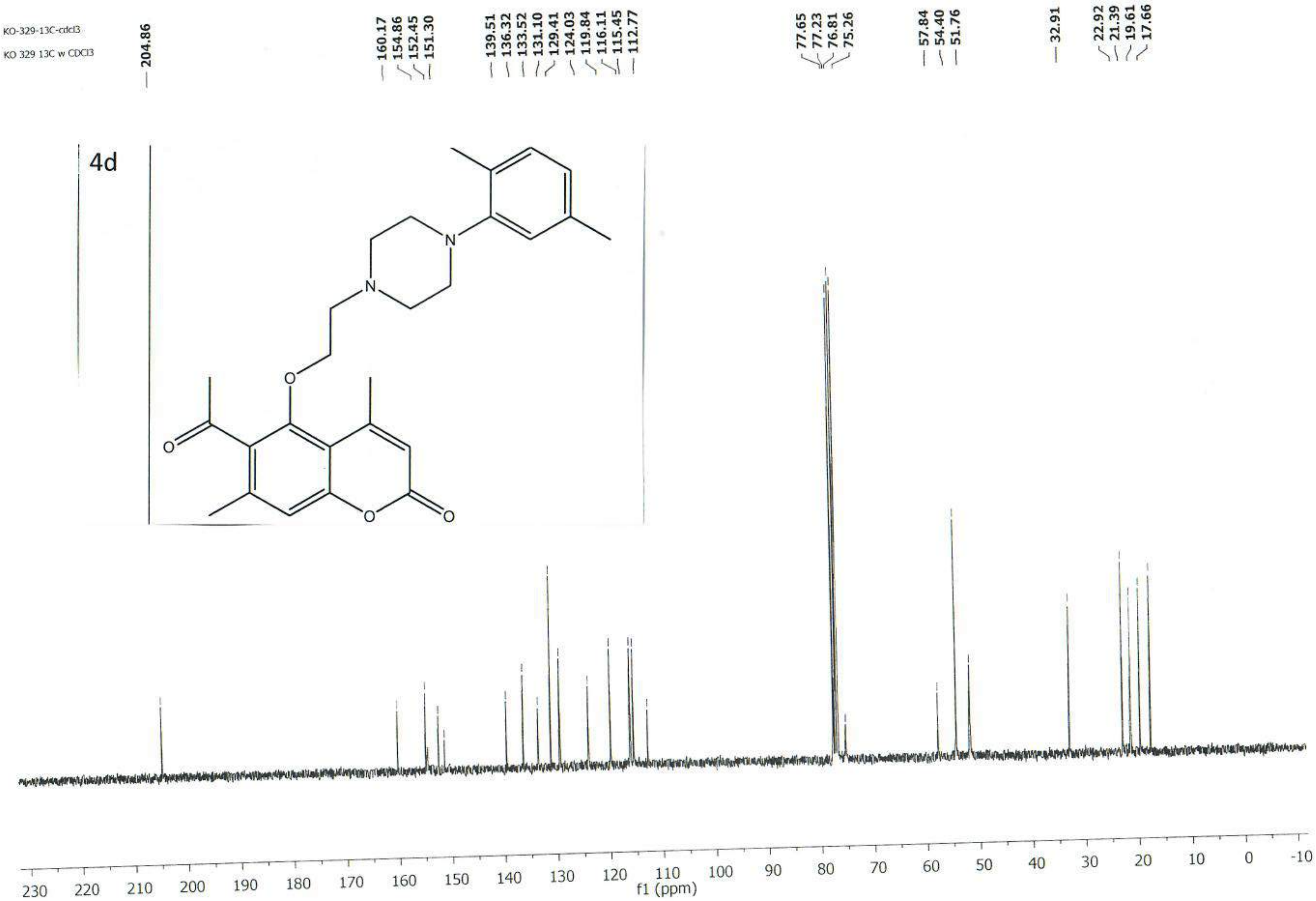

KO-329-13C-cdcl3
KO 329 13C w CDCl3
204.86
160.17
154.86
152.45
151.30
139.51
136.32
133.52
131.10
129.41
124.03
119.84
116.11
115.45
112.77
77.65
77.23
76.81
75.26
57.84
54.40
51.76
32.91
22.92
21.39
19.61
17.66
4d
230 220 210 200 190 180 170 160 150 140 130 120 110 100 90 80 70 60 50 40 30 20 10 0 -10
f1 (ppm)

4e

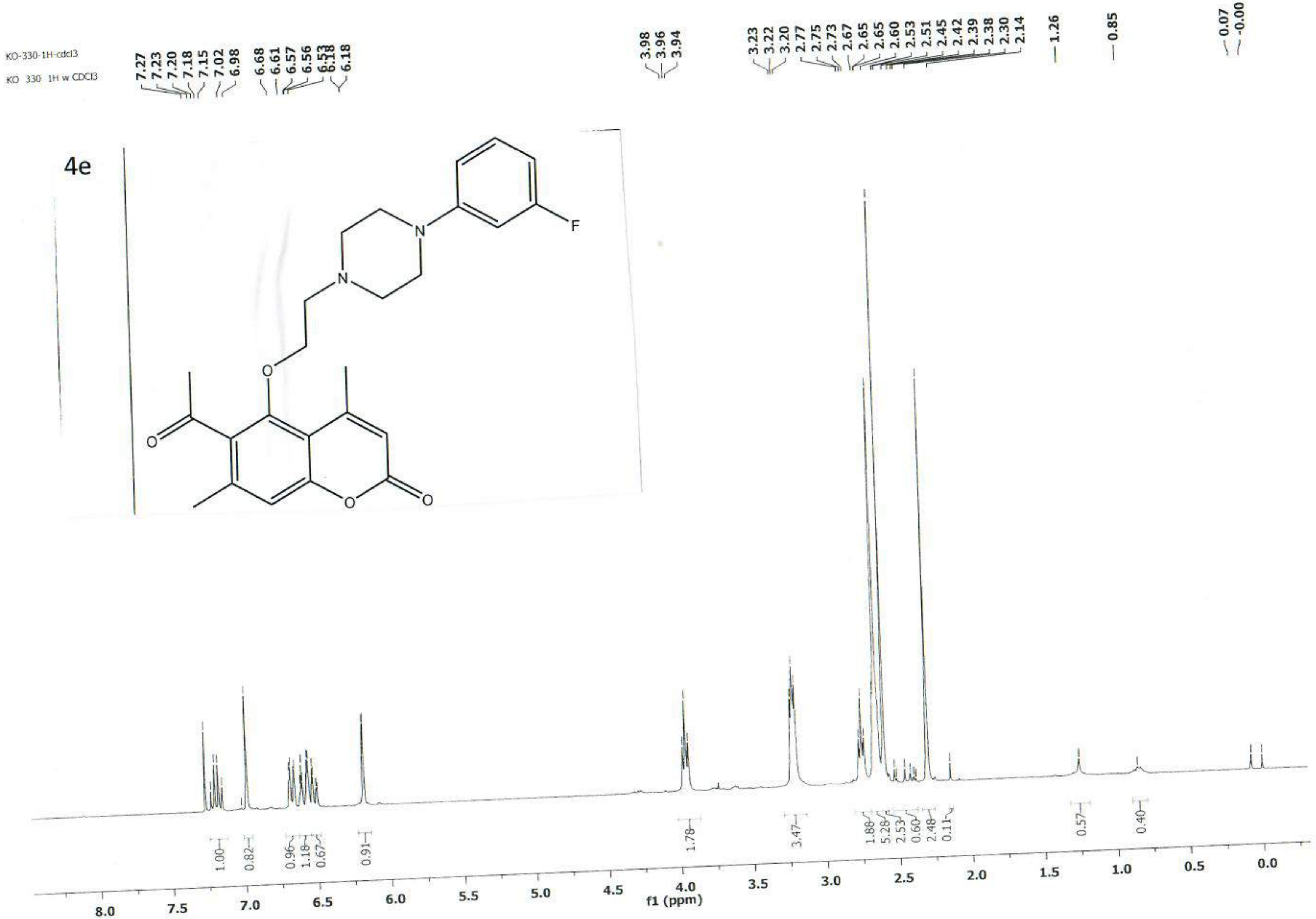

KO-330-1H-cdcl3
KO 330 1H w CDCl3
7.27
7.23
7.20
7.18
7.15
7.02
6.98
6.68
6.61
6.57
6.56
6.53
6.18
3.98
3.96
3.94
3.23
3.22
3.20
2.77
2.75
2.73
2.67
2.65
2.65
2.60
2.53
2.51
2.45
2.42
2.39
2.38
2.30
2.14
1.26
0.85
0.07
-0.00
4e
N
N
F
O
O
O
O
1.00
0.82
0.96
1.18
0.67
0.91
1.78
3.47
1.88
5.28
2.53
0.60
2.48
0.11
0.57
0.40
8.0
7.5
7.0
6.5
6.0
5.5
5.0
4.5
4.0
3.5
3.0
2.5
2.0
1.5
1.0
0.5
0.0
f1 (ppm)

4e

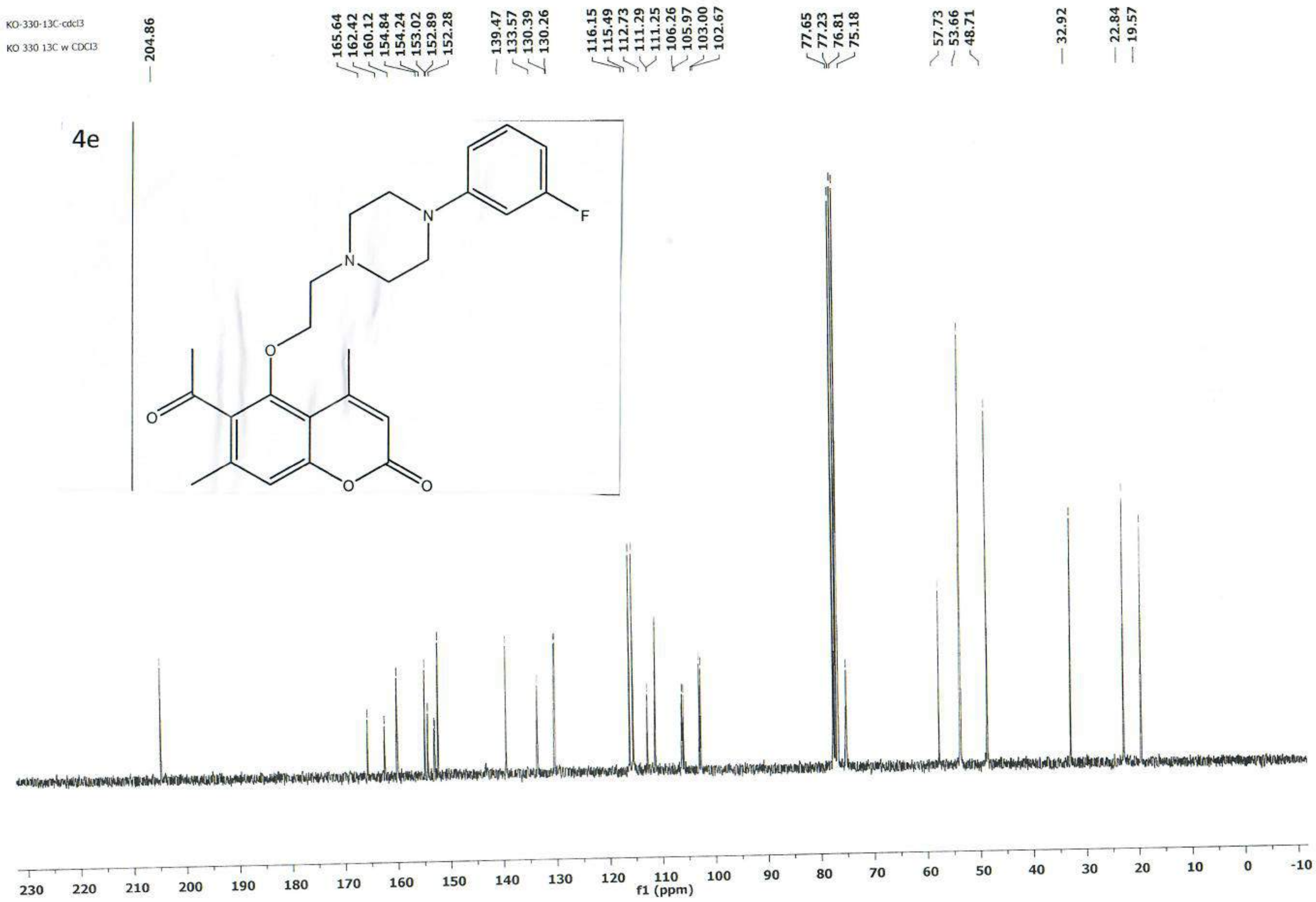
KO-330-13C-cdcl3
KO 330 13C w CDCl3
204.86
165.64
162.42
160.12
154.84
154.24
153.02
152.89
152.28
139.47
133.57
130.39
130.26
116.15
115.49
112.73
111.29
111.25
106.26
105.97
103.00
102.67
77.65
77.23
76.81
75.18
57.73
53.66
48.71
32.92
22.84
19.57
4e
N
N
F
O
O
O
O
230
220
210
200
190
180
170
160
150
140
130
120
110
100
90
80
70
60
50
40
30
20
10
0
-10
f1 (ppm)

4f

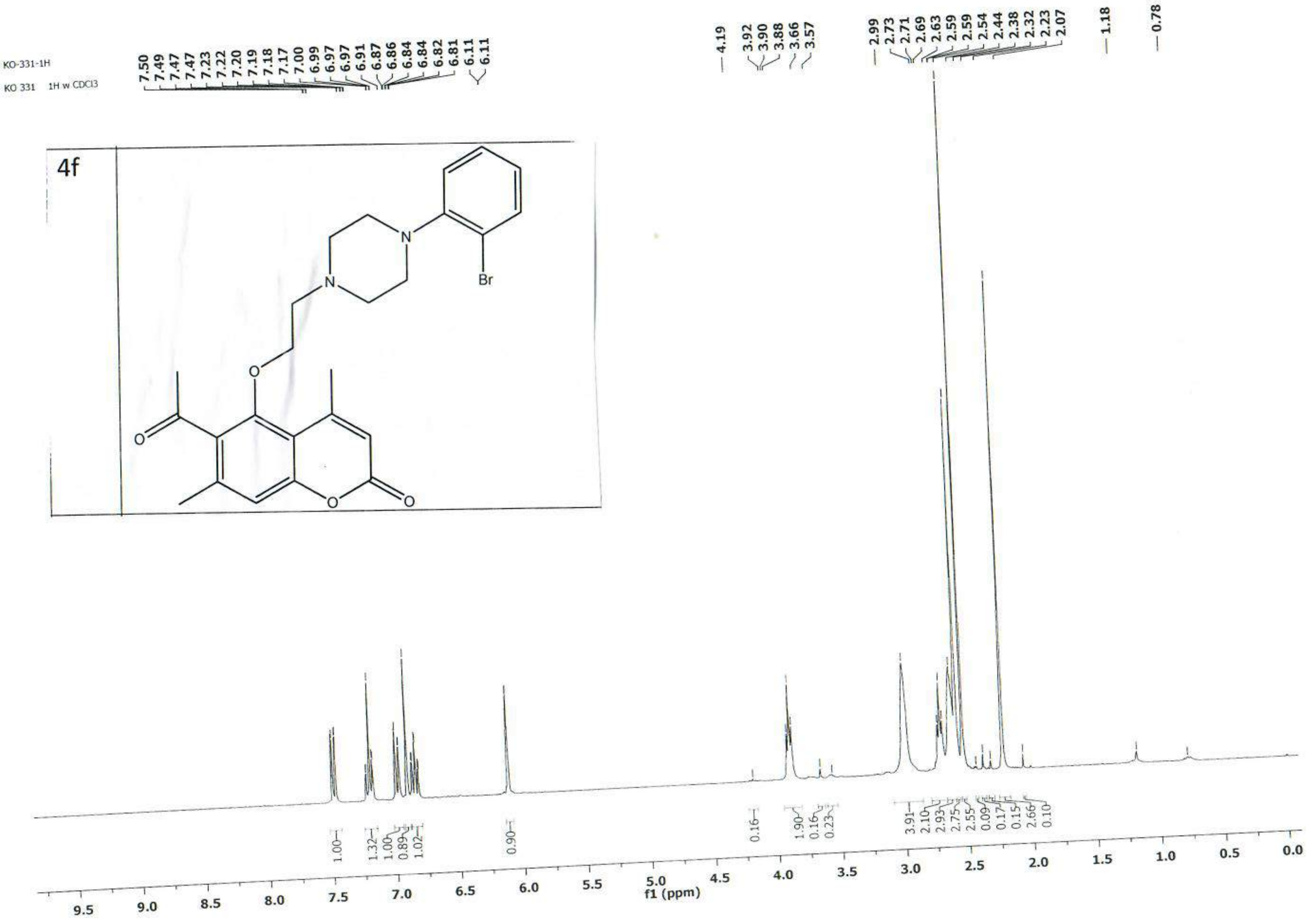
KO-331-1H
KO 331 1H w CDCl3
7.50 7.49 7.47 7.47 7.23 7.22 7.20 7.19 7.18 7.17 7.00 6.99 6.97 6.97 6.91 6.87 6.86 6.84 6.84 6.82 6.81 6.11 6.11
4.19 3.92 3.90 3.88 3.66 3.57
2.99 2.73 2.71 2.69 2.63 2.59 2.59 2.54 2.44 2.38 2.32 2.23 2.07
1.18
0.78
4f
N
N
Br
O
O
O
O
1.00 1.32 1.00 0.89 1.02 0.90 0.16 1.90 0.16 0.23 3.91 2.10 2.93 2.75 2.55 0.09 0.17 0.15 2.66 0.10
9.5 9.0 8.5 8.0 7.5 7.0 6.5 6.0 5.5 5.0 4.5 4.0 3.5 3.0 2.5 2.0 1.5 1.0 0.5 0.0
f1 (ppm)

KO-331-13C

KO 331 13C w CDCl3

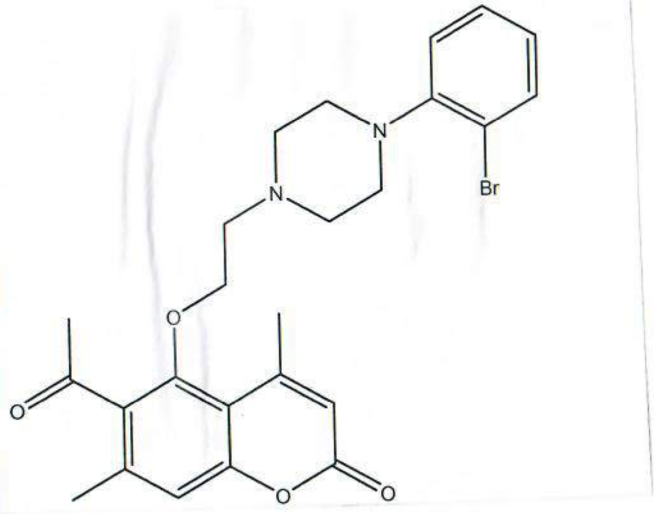
4f

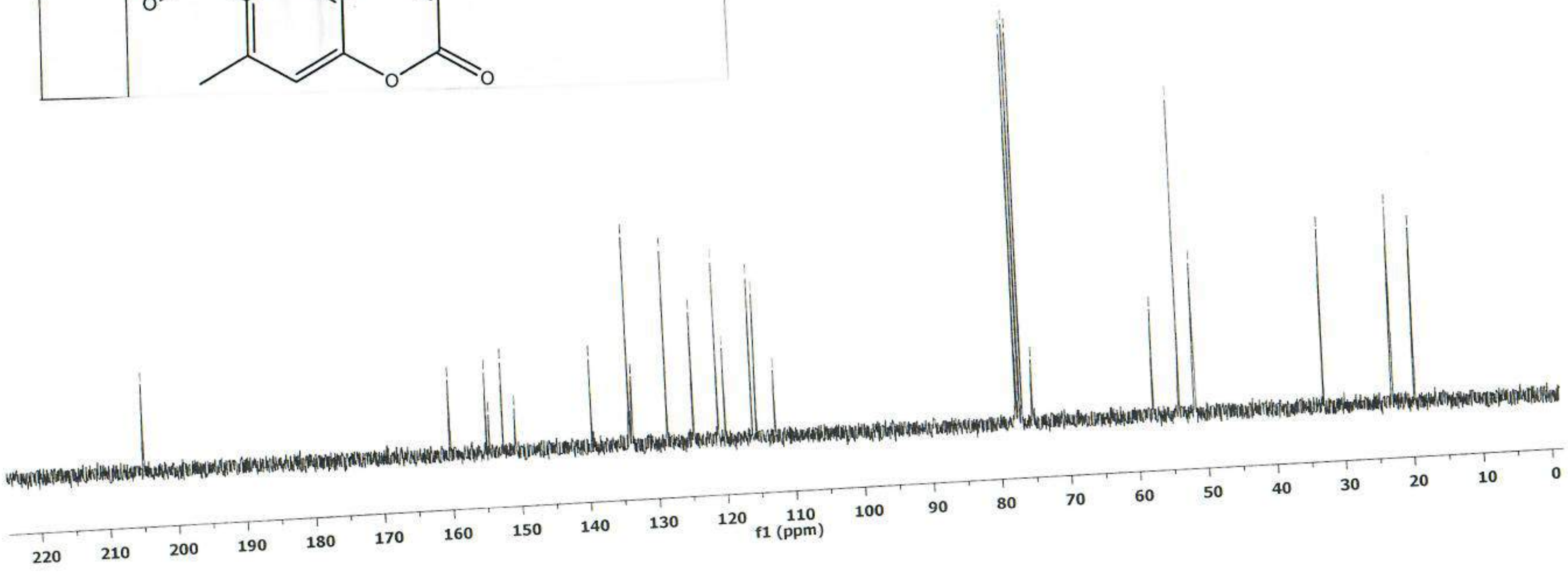

48

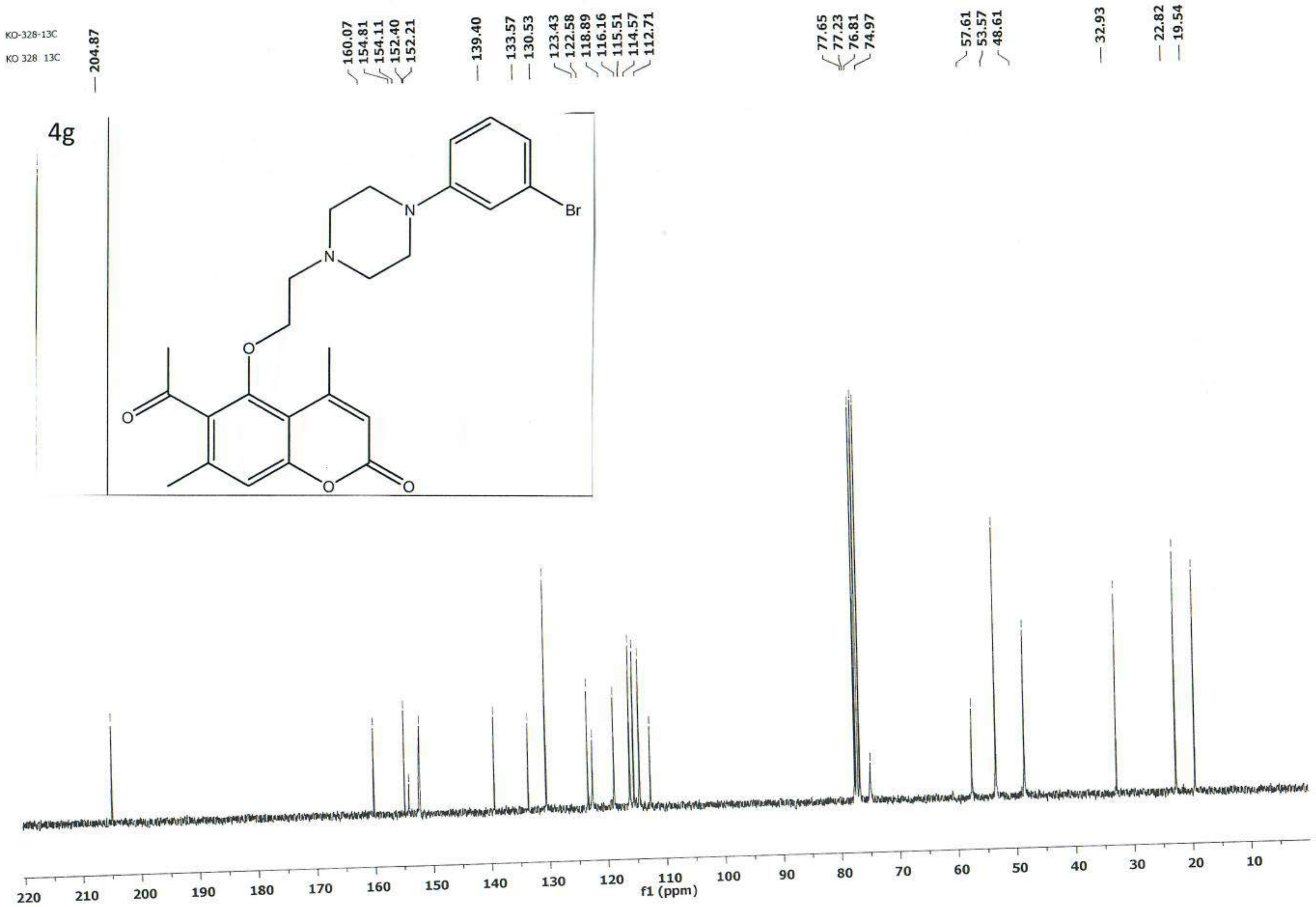

KO-328-13C
KO 328 13C
204.87
160.07
154.81
154.11
152.40
152.21
139.40
133.57
130.53
123.43
122.58
118.89
116.16
115.51
114.57
112.71
77.65
77.23
76.81
74.97
57.61
53.57
48.61
32.93
22.82
19.54
4g
Br
220 210 200 190 180 170 160 150 140 130 120 110 100 90 80 70 60 50 40 30 20 10
f1 (ppm)

4g

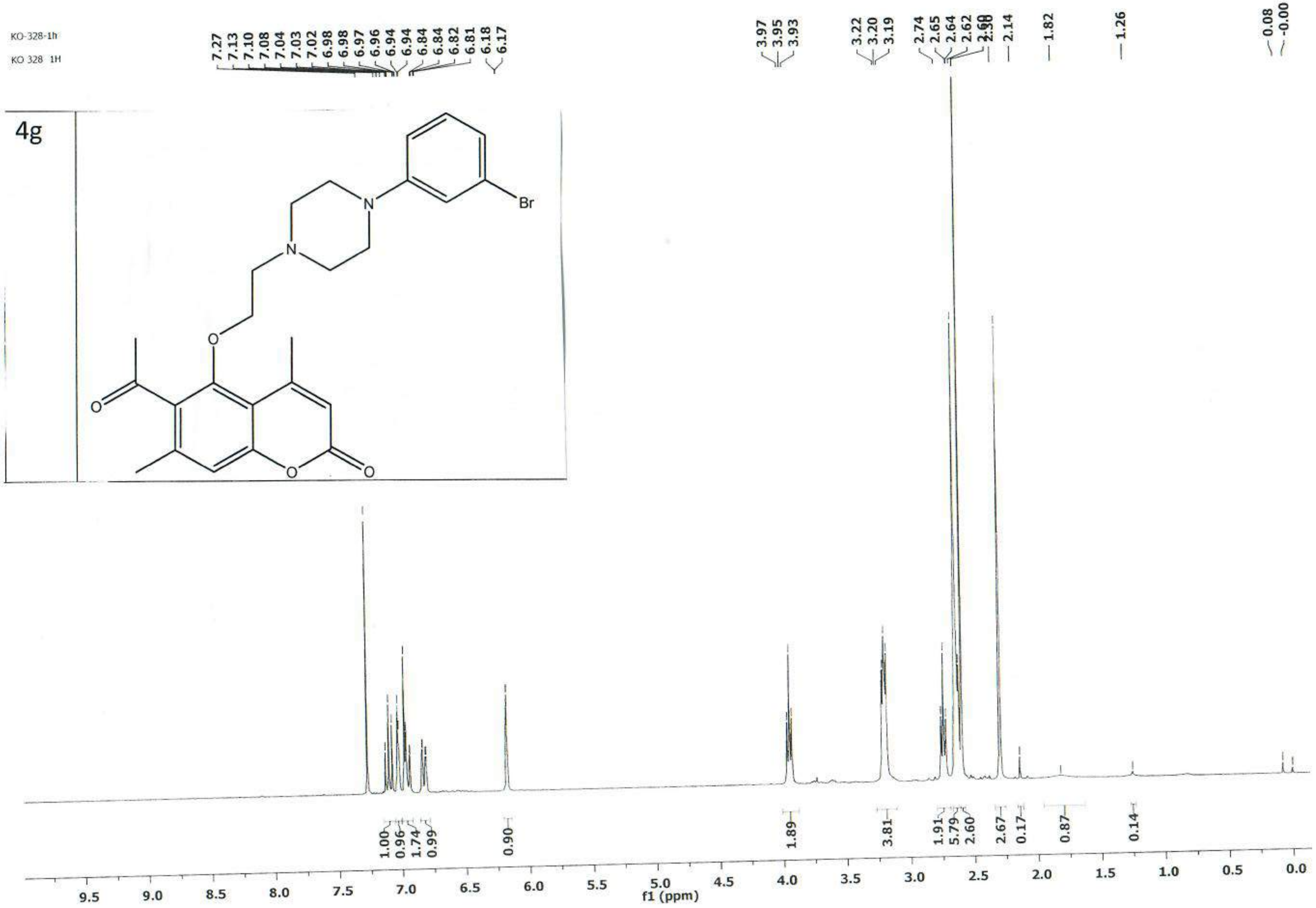
KO-328-1h
KO 328 1H
7.27
7.13
7.10
7.08
7.04
7.03
7.02
6.98
6.98
6.97
6.96
6.94
6.94
6.84
6.84
6.82
6.81
6.18
6.17
3.97
3.95
3.93
3.22
3.20
3.19
2.74
2.65
2.64
2.62
2.14
1.82
1.26
0.08
-0.00
4g
N
N
Br
O
O
O
O
1.00
0.96
1.74
0.99
0.90
1.89
3.81
1.91
5.79
2.60
2.67
0.17
0.87
0.14
9.5
9.0
8.5
8.0
7.5
7.0
6.5
6.0
5.5
5.0
4.5
4.0
3.5
3.0
2.5
2.0
1.5
1.0
0.5
0.0
f1 (ppm)

4h

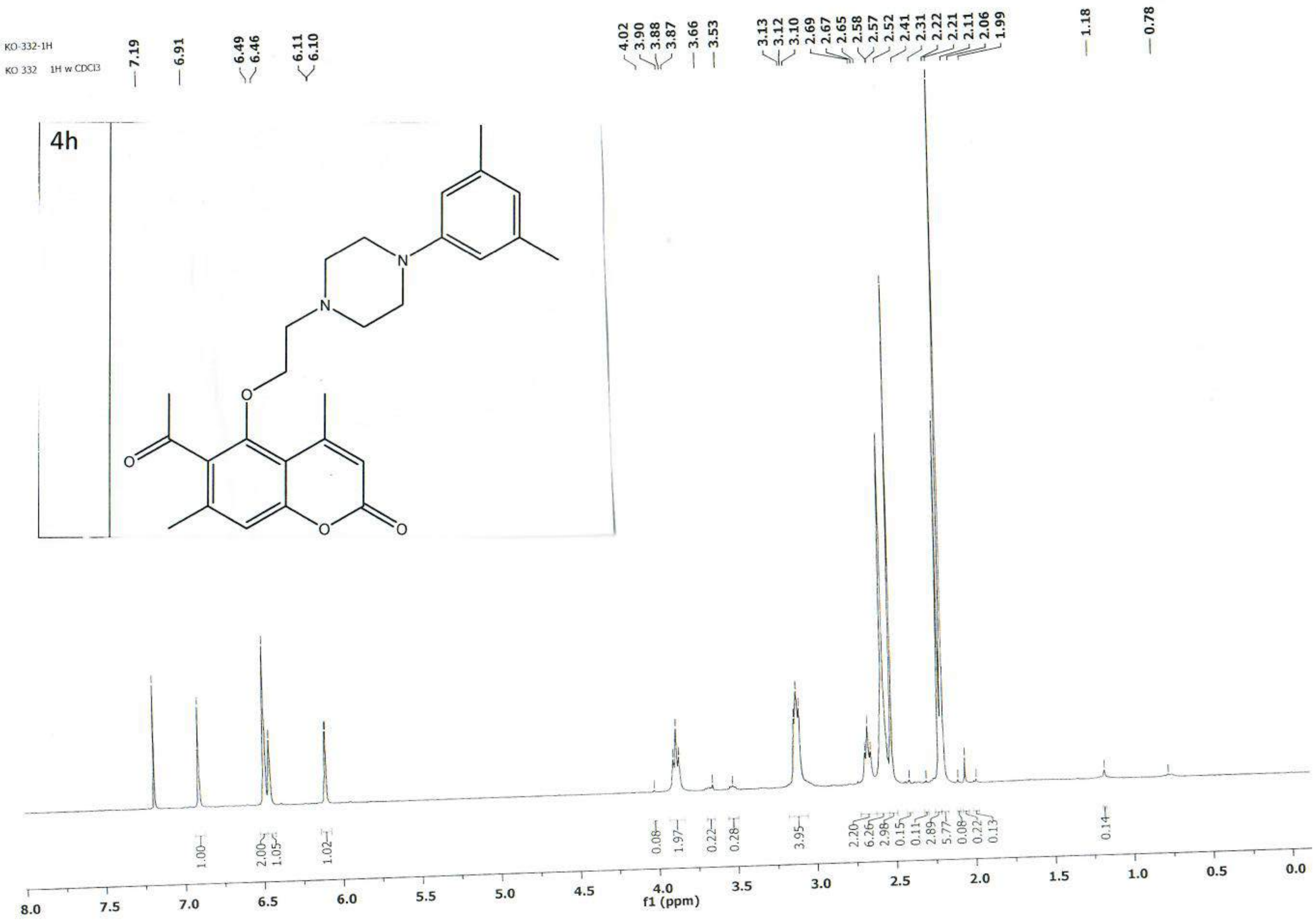
KO-332-1H
KO 332 1H w CDCl3
7.19
6.91
6.49
6.46
6.11
6.10
4.02
3.90
3.88
3.87
3.66
3.53
3.13
3.12
3.10
2.69
2.67
2.65
2.58
2.57
2.52
2.41
2.31
2.22
2.21
2.11
2.06
1.99
1.18
0.78
4h
1.00
2.00
1.05
1.02
0.08
1.97
0.22
0.28
3.95
2.20
6.26
2.98
0.15
0.11
2.89
5.77
0.08
0.22
0.13
0.14
f1 (ppm)

4h

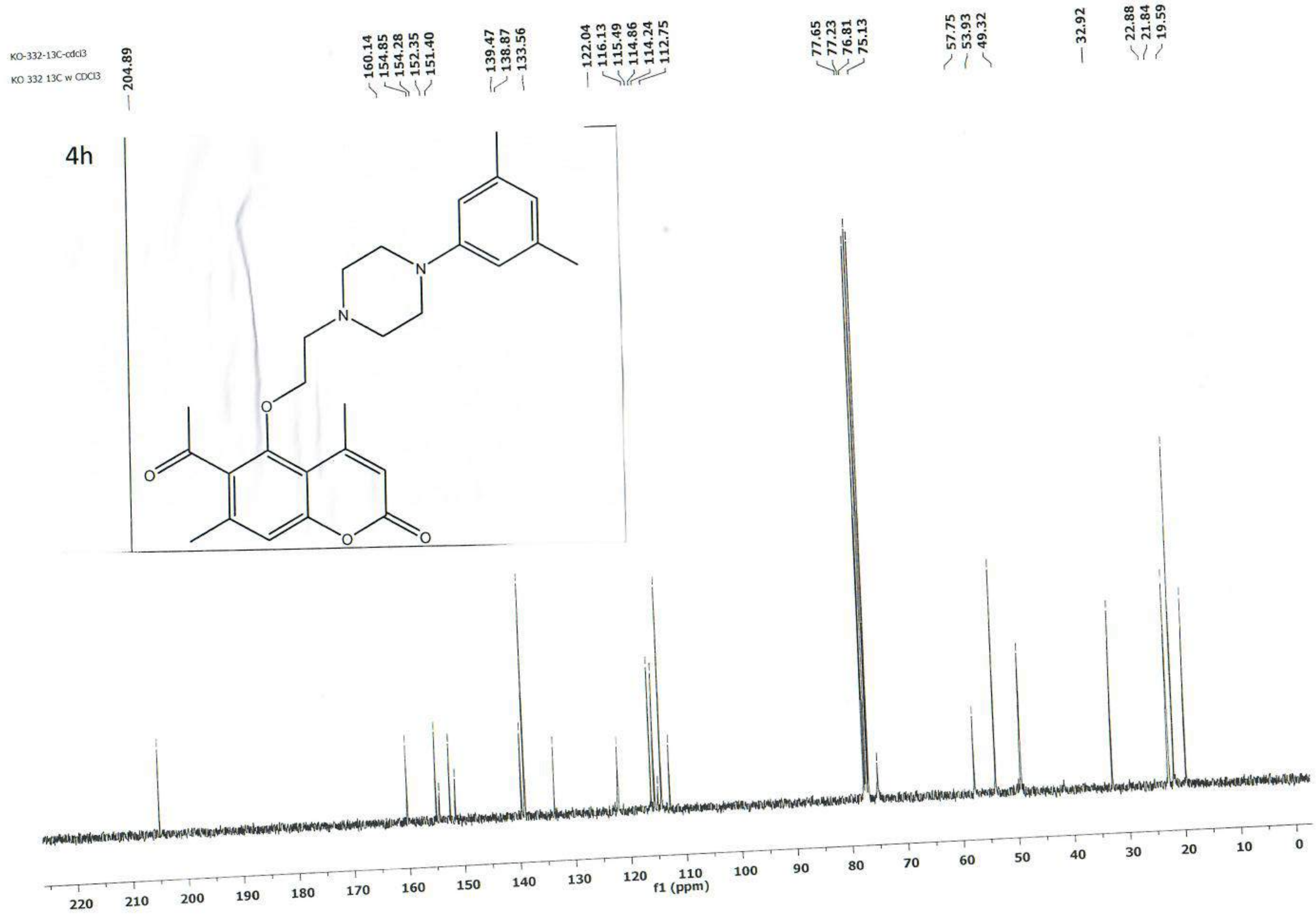
KO-332-13C-cdcl3
KO 332 13C w CDCl3
204.89
160.14
154.85
154.28
152.35
151.40
139.47
138.87
133.56
122.04
116.13
115.49
114.86
114.24
112.75
77.65
77.23
76.81
75.13
57.75
53.93
49.32
32.92
22.88
21.84
19.59
f1 (ppm)

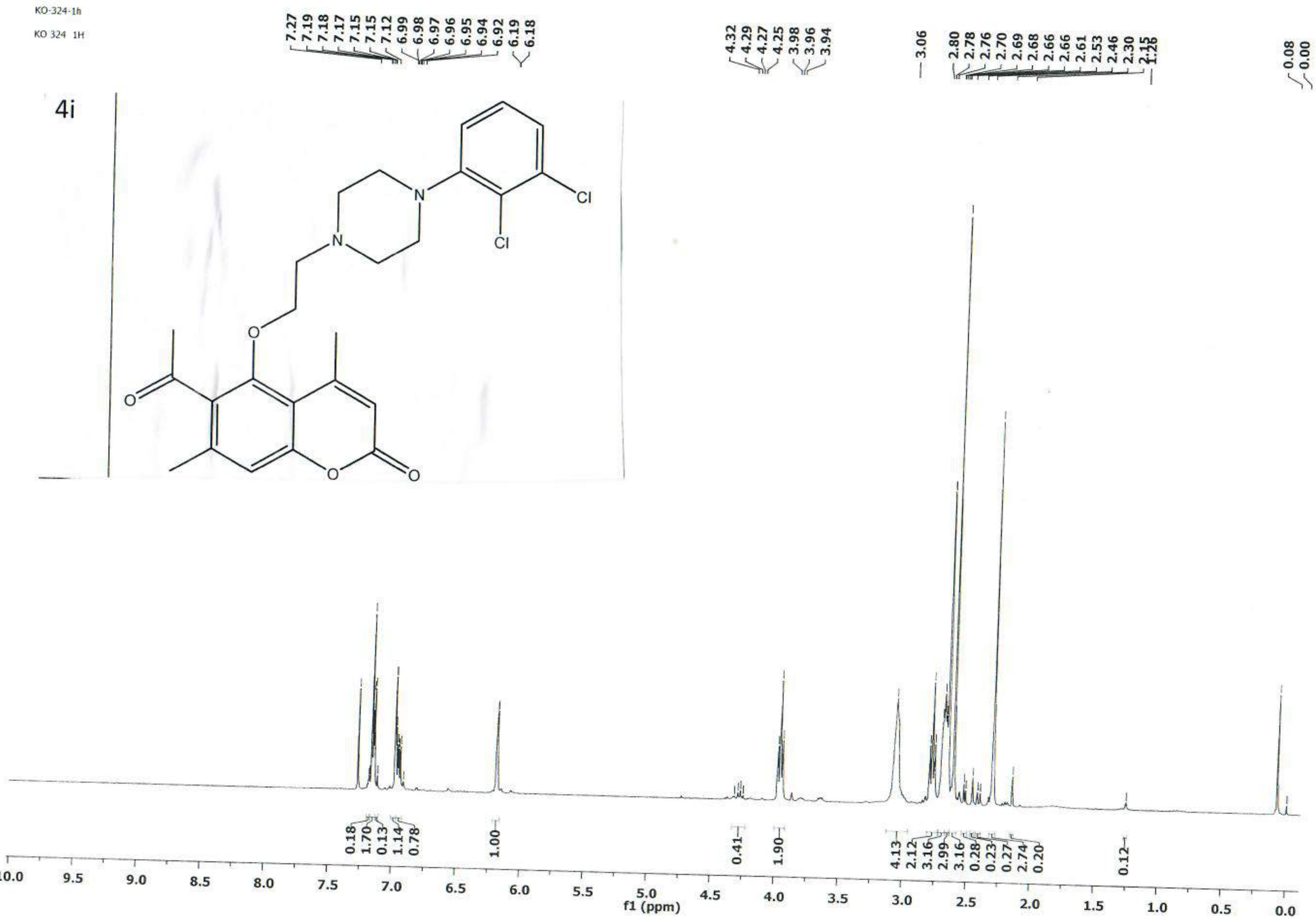

KO-324-1h
KO 324 1H
7.27 7.19 7.18 7.17 7.15 7.15 7.12 6.99 6.98 6.97 6.96 6.95 6.94 6.92 6.19 6.18
4.32 4.29 4.27 4.25 3.98 3.96 3.94
3.06
2.80 2.78 2.76 2.70 2.69 2.68 2.66 2.66 2.61 2.53 2.46 2.30 2.15 1.26
0.08 0.00
4i
N
N
Cl
Cl
O
O
O
O
0.18 1.70 0.13 1.14 0.78
1.00
0.41
1.90
4.13 2.12 3.16 2.99 3.16 0.28 0.23 0.27 2.74 0.20
0.12
10.0 9.5 9.0 8.5 8.0 7.5 7.0 6.5 6.0 5.5 5.0 4.5 4.0 3.5 3.0 2.5 2.0 1.5 1.0 0.5 0.0
f1 (ppm)

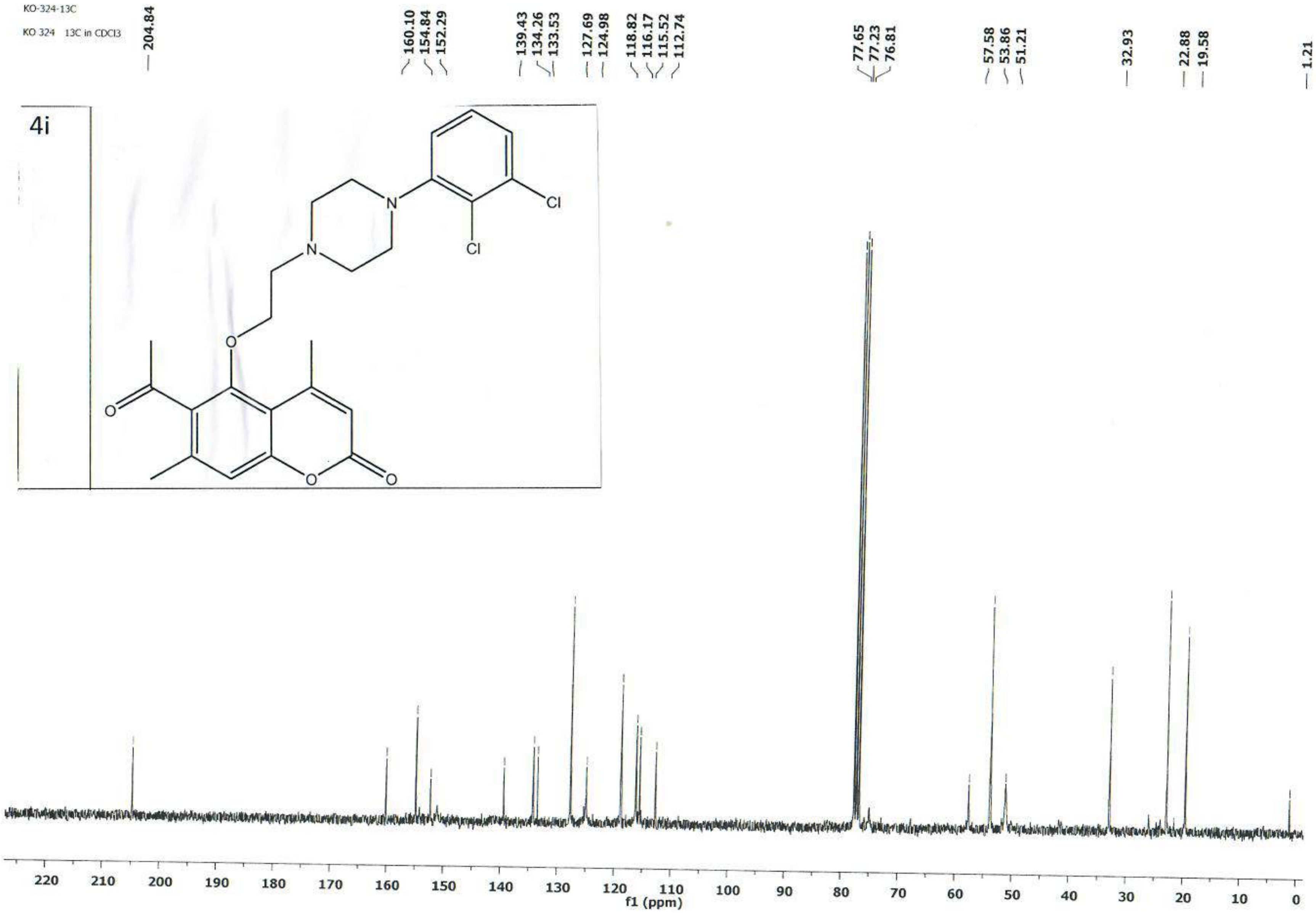

KO-324-13C
KO 324 13C in CDCl3
204.84
160.10
154.84
152.29
139.43
134.26
133.53
127.69
124.98
118.82
116.17
115.52
112.74
77.65
77.23
76.81
57.58
53.86
51.21
32.93
22.88
19.58
1.21
4i
N
N
Cl
Cl
O
O
O
O
220 210 200 190 180 170 160 150 140 130 120 110 100 90 80 70 60 50 40 30 20 10 0
f1 (ppm)

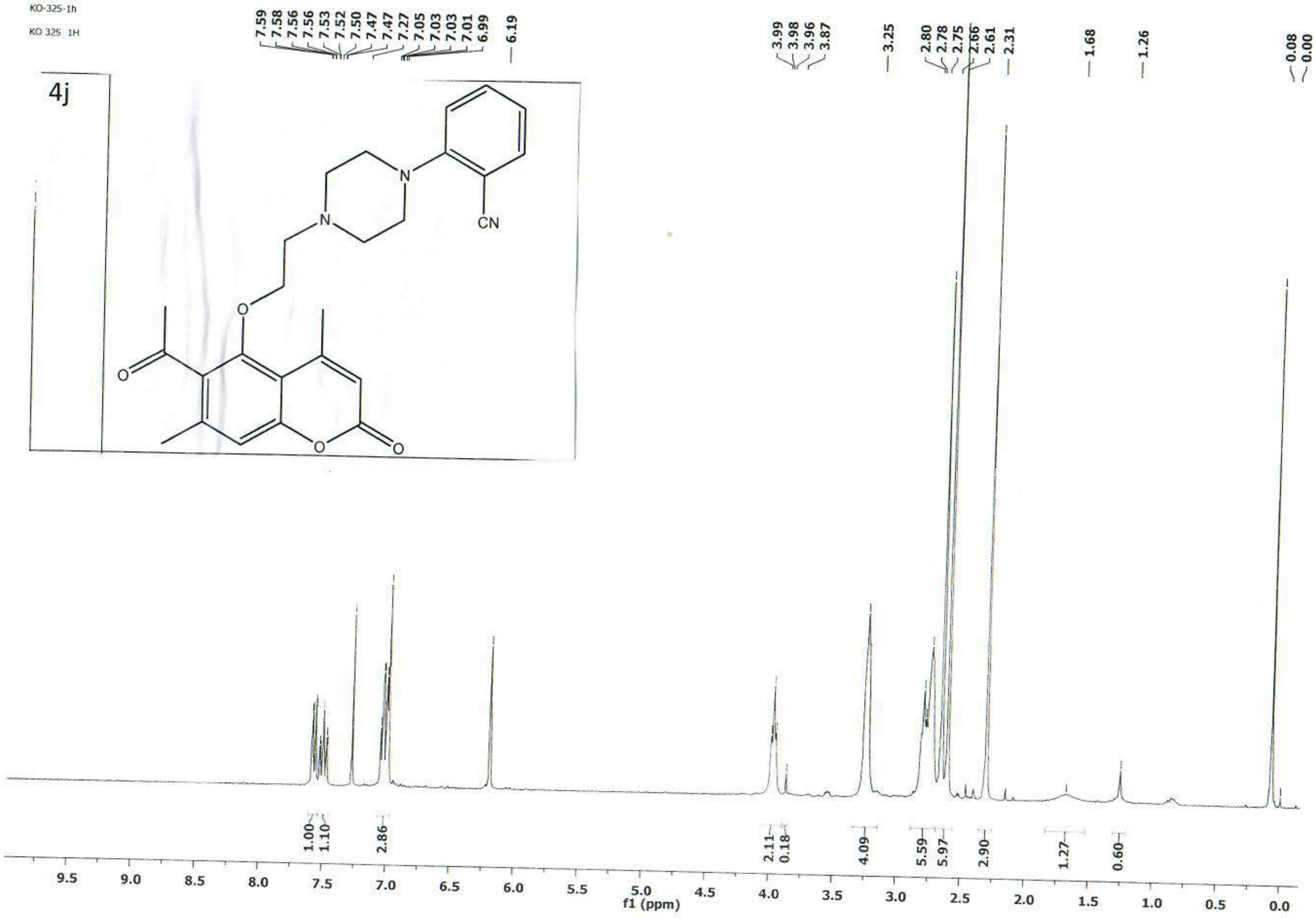

KO-325-1h
KO 325 1H
4j
CN
7.59 7.58 7.56 7.56 7.53 7.52 7.50 7.47 7.47 7.27 7.05 7.03 7.03 7.01 6.99
6.19
3.99 3.98 3.96 3.87
3.25
2.80 2.78 2.75 2.66 2.61 2.31
1.68
1.26
0.08 0.00
1.00 1.10 2.86 2.11 0.18 4.09 5.59 5.97 2.90 1.27 0.60
f1 (ppm)

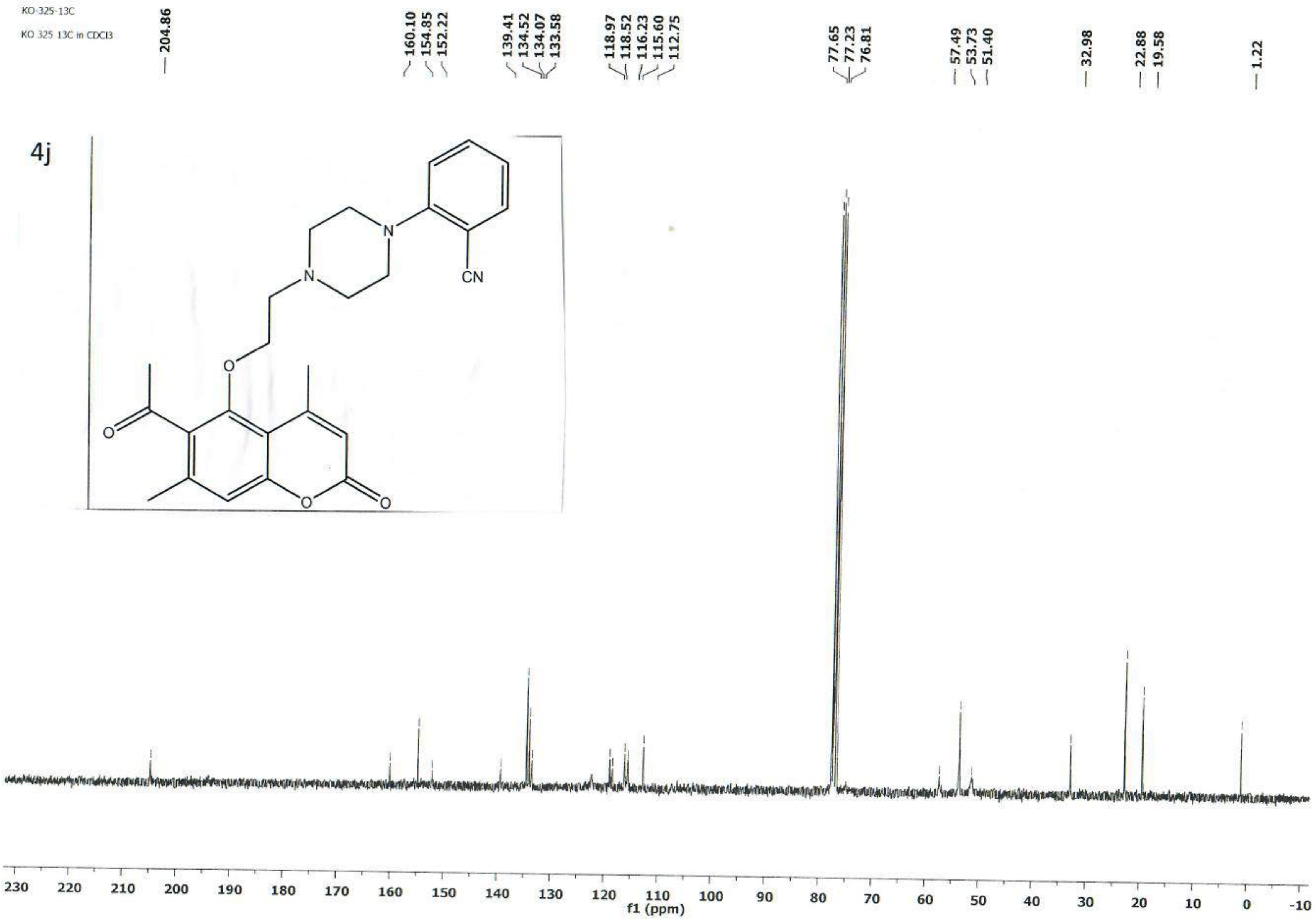

KO-325-13C
KO 325 13C in CDCl3
204.86
160.10
154.85
152.22
139.41
134.52
134.07
133.58
118.97
118.52
116.23
115.60
112.75
77.65
77.23
76.81
57.49
53.73
51.40
32.98
22.88
19.58
1.22
4j
N
N
CN
O
O
O
O
230 220 210 200 190 180 170 160 150 140 130 120 110 100 90 80 70 60 50 40 30 20 10 0 -10
f1 (ppm)

5

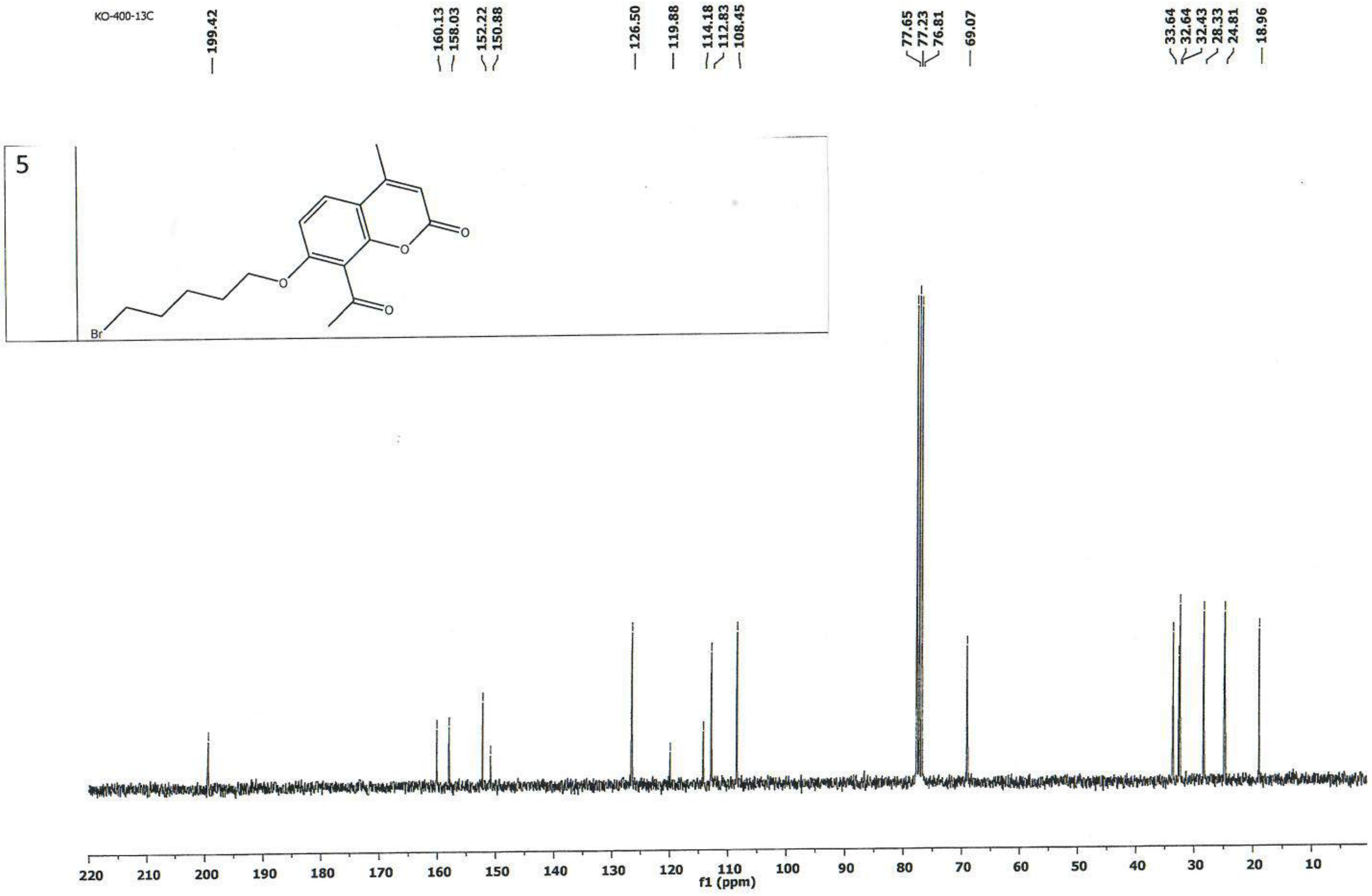
KO-400-13C
199.42
160.13
158.03
152.22
150.88
126.50
119.88
114.18
112.83
108.45
77.65
77.23
76.81
69.07
33.64
32.64
32.43
28.33
24.81
18.96
5
Br
O
O
O
O
220 210 200 190 180 170 160 150 140 130 120 110 100 90 80 70 60 50 40 30 20 10
f1 (ppm)

5

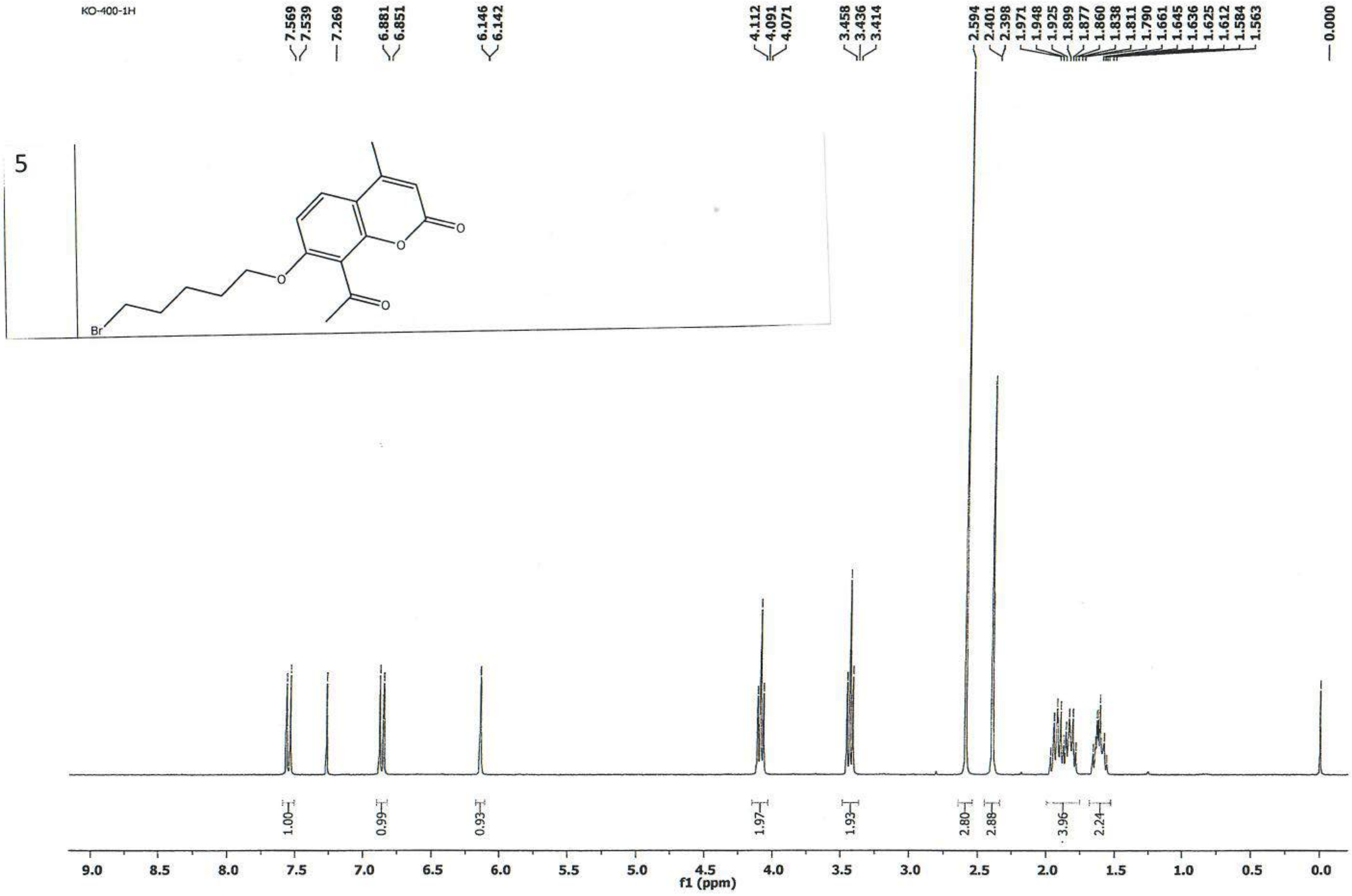
KO-400-1H
7.569
7.539
7.269
6.881
6.851
6.146
6.142
4.112
4.091
4.071
3.458
3.436
3.414
2.594
2.401
2.398
1.971
1.948
1.925
1.899
1.877
1.860
1.838
1.811
1.790
1.661
1.645
1.636
1.625
1.612
1.584
1.563
0.000
5
Br
O
O
O
O
1.00
0.99
0.93
1.97
1.93
2.80
2.88
3.96
2.24
9.0 8.5 8.0 7.5 7.0 6.5 6.0 5.5 5.0 4.5 4.0 3.5 3.0 2.5 2.0 1.5 1.0 0.5 0.0
f1 (ppm)

5a

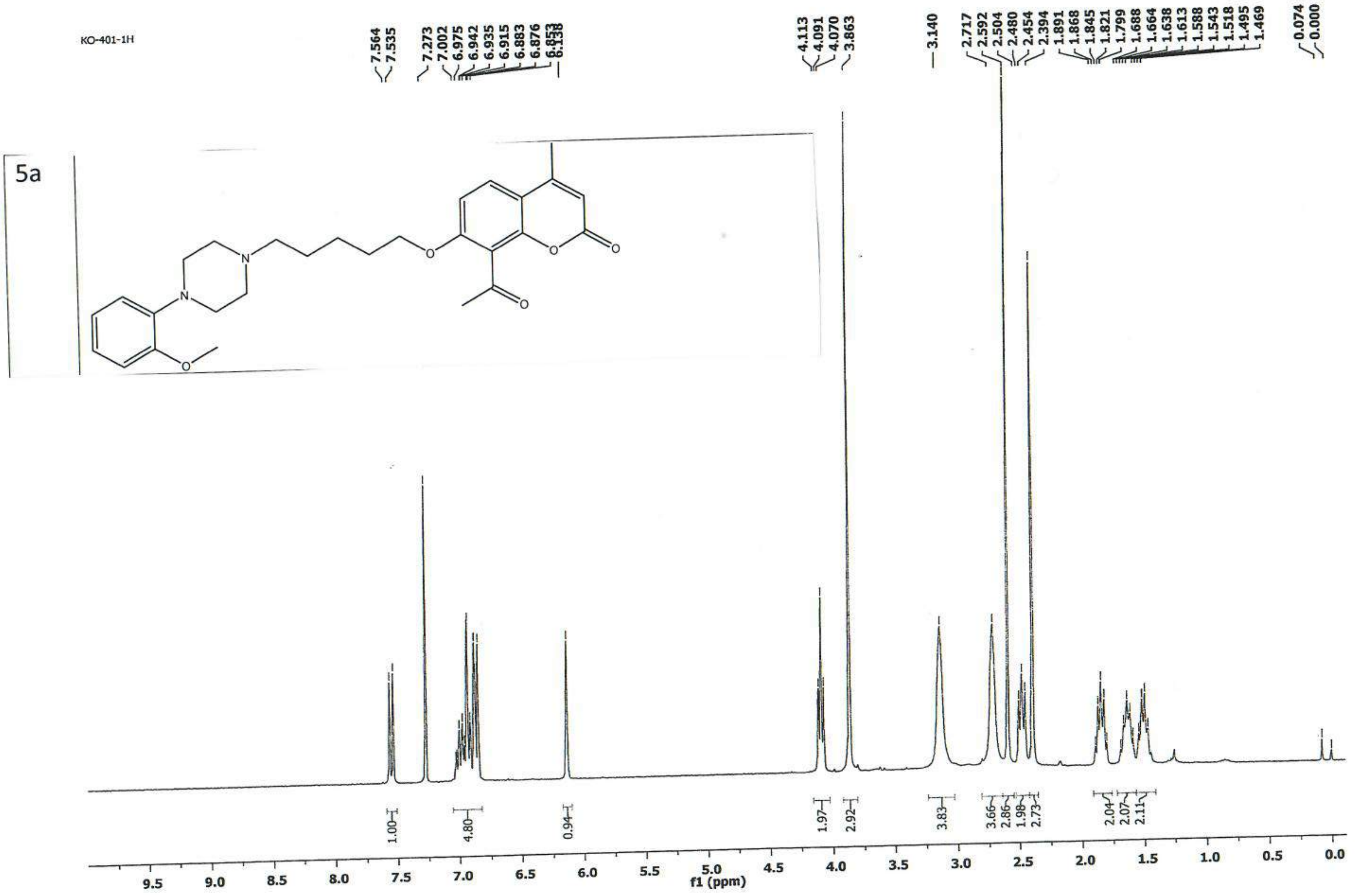
KO-401-1H
7.564
7.535
7.273
7.002
6.975
6.942
6.935
6.915
6.883
6.876
6.853
6.138
4.113
4.091
4.070
3.863
3.140
2.717
2.592
2.504
2.480
2.454
2.394
1.891
1.868
1.845
1.821
1.799
1.688
1.664
1.638
1.613
1.588
1.543
1.518
1.495
1.469
0.074
0.000
5a
N
N
O
O
O
O
O
1.00
4.80
0.94
1.97
2.92
3.83
3.66
2.86
1.98
2.73
2.04
2.07
2.11
9.5
9.0
8.5
8.0
7.5
7.0
6.5
6.0
5.5
5.0
4.5
4.0
3.5
3.0
2.5
2.0
1.5
1.0
0.5
0.0
f1 (ppm)

5a

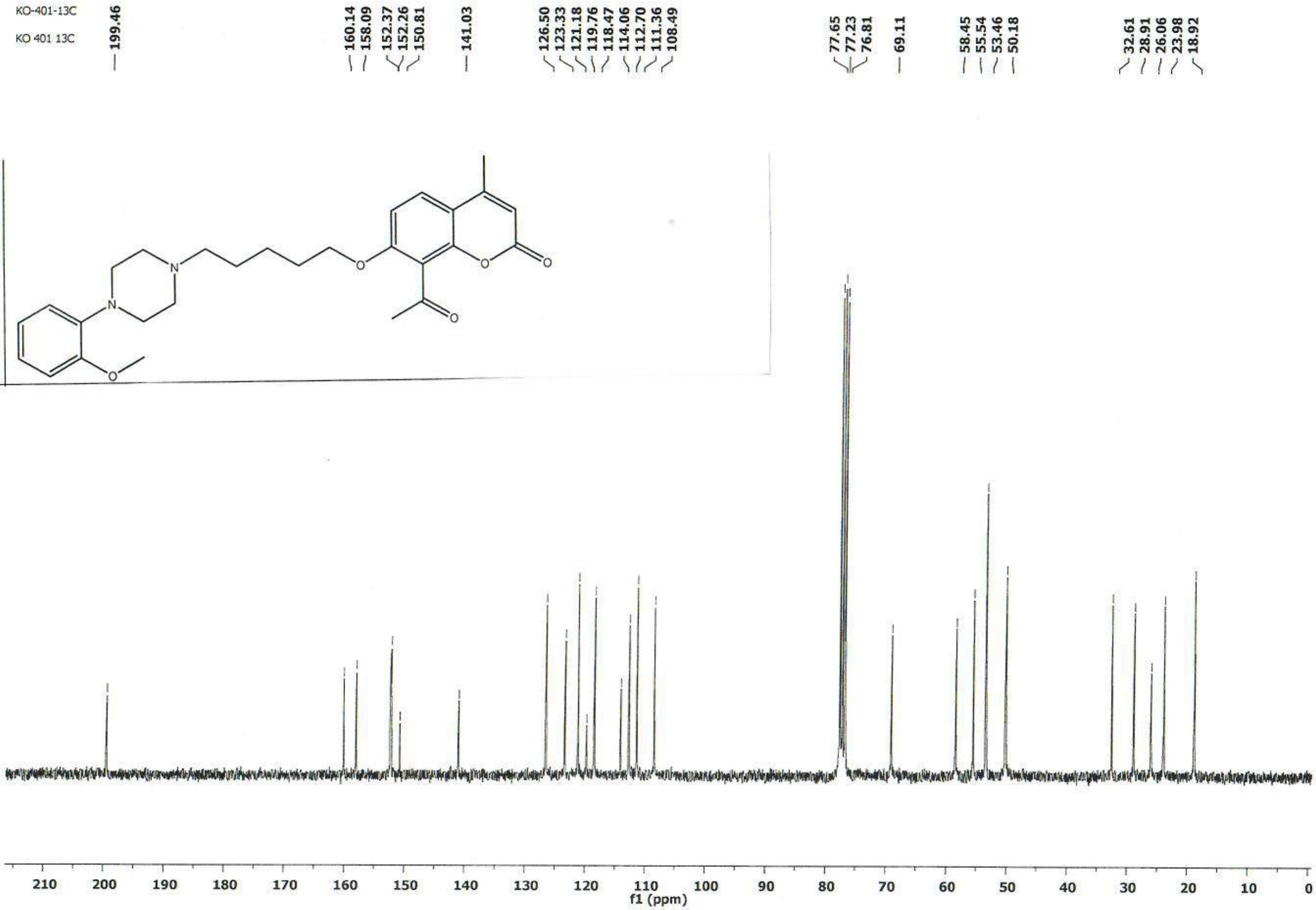
KO-401-13C
KO 401 13C
199.46
160.14
158.09
152.37
152.26
150.81
141.03
126.50
123.33
121.18
119.76
118.47
114.06
112.70
111.36
108.49
77.65
77.23
76.81
69.11
58.45
55.54
53.46
50.18
32.61
28.91
26.06
23.98
18.92
210 200 190 180 170 160 150 140 130 120 110 100 90 80 70 60 50 40 30 20 10 0
f1 (ppm)
N
N
O
O
O
O
O

5a

56

5b

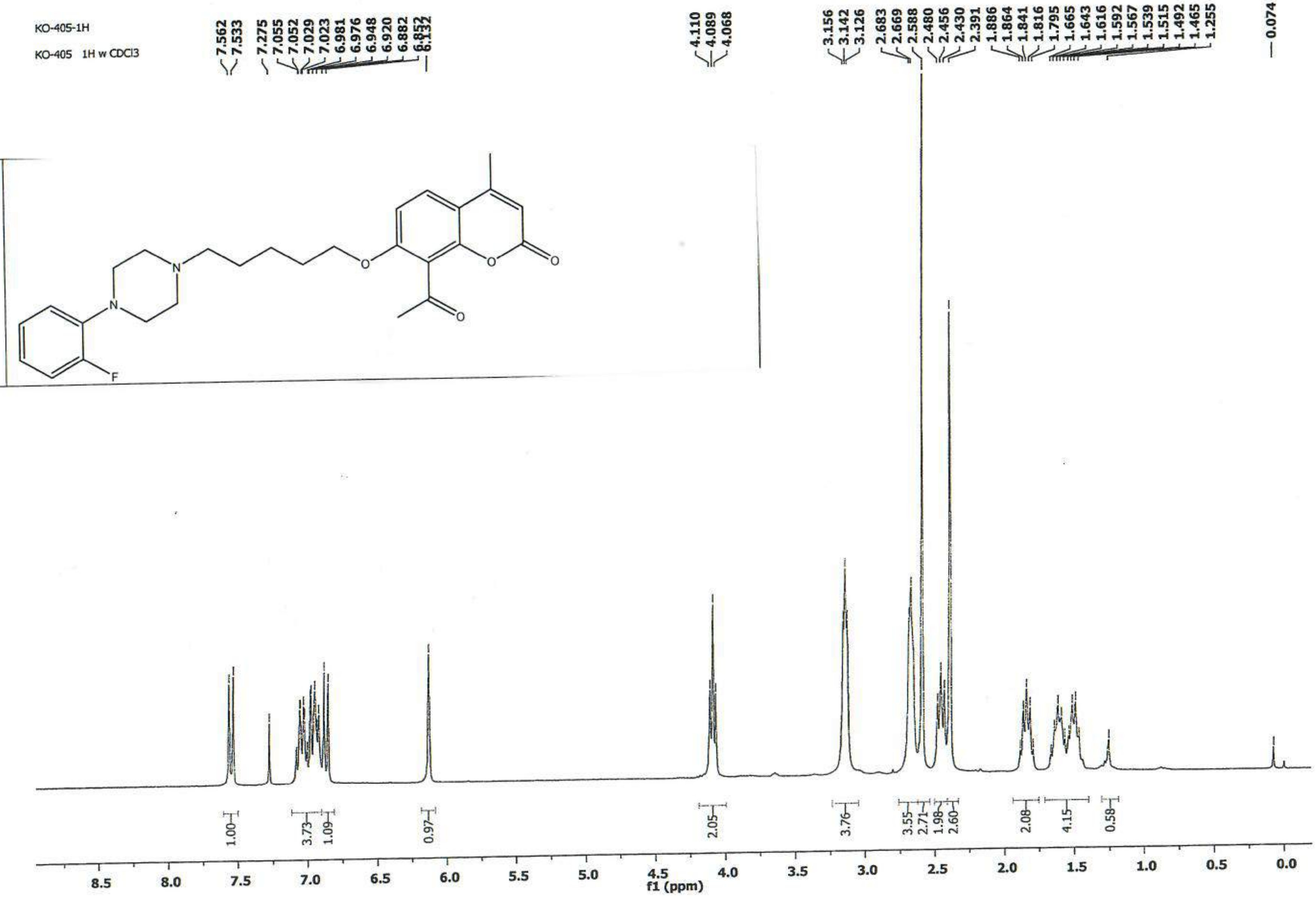
KO-405-1H
KO-405 1H w CDCl3
7.562
7.533
7.275
7.055
7.052
7.029
7.023
6.981
6.976
6.948
6.920
6.882
6.852
6.132
4.110
4.089
4.068
3.156
3.142
3.126
2.683
2.669
2.588
2.480
2.456
2.430
2.391
1.886
1.864
1.841
1.816
1.795
1.665
1.643
1.616
1.592
1.567
1.539
1.515
1.492
1.465
1.255
0.074
1.00
3.73
1.09
0.97
2.05
3.76
3.55
2.71
1.98
2.60
2.08
4.15
0.58
f1 (ppm)

5b

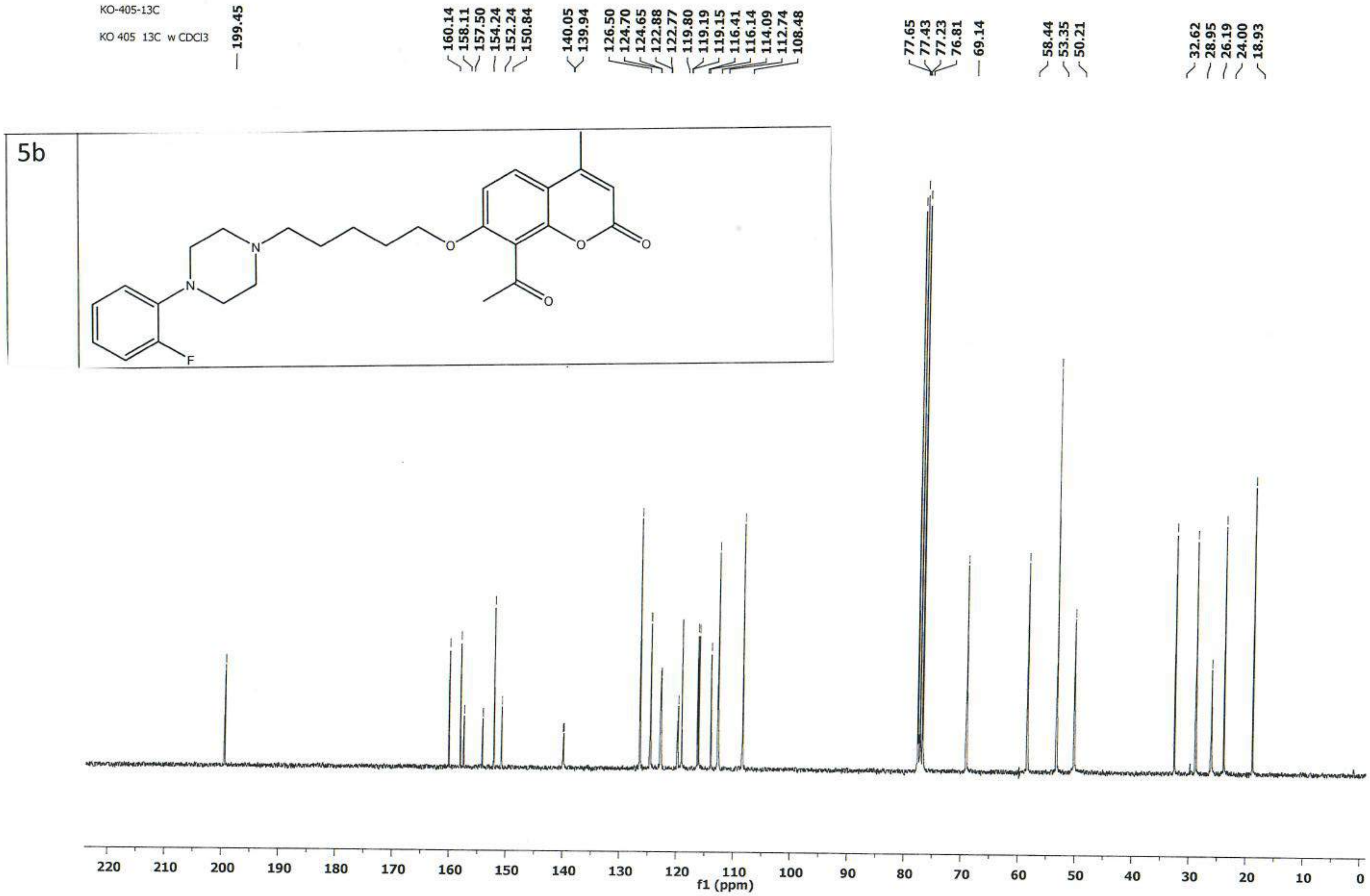
KO-405-13C
KO 405 13C w CDCl3
199.45
160.14
158.11
157.50
154.24
152.24
150.84
140.05
139.94
126.50
124.70
124.65
122.88
122.77
119.80
119.19
119.15
116.41
116.14
114.09
112.74
108.48
77.65
77.43
77.23
76.81
69.14
58.44
53.35
50.21
32.62
28.95
26.19
24.00
18.93
5b
N
N
O
O
O
O
F
220
210
200
190
180
170
160
150
140
130
120
110
100
90
80
70
60
50
40
30
20
10
0
f1 (ppm)

5c

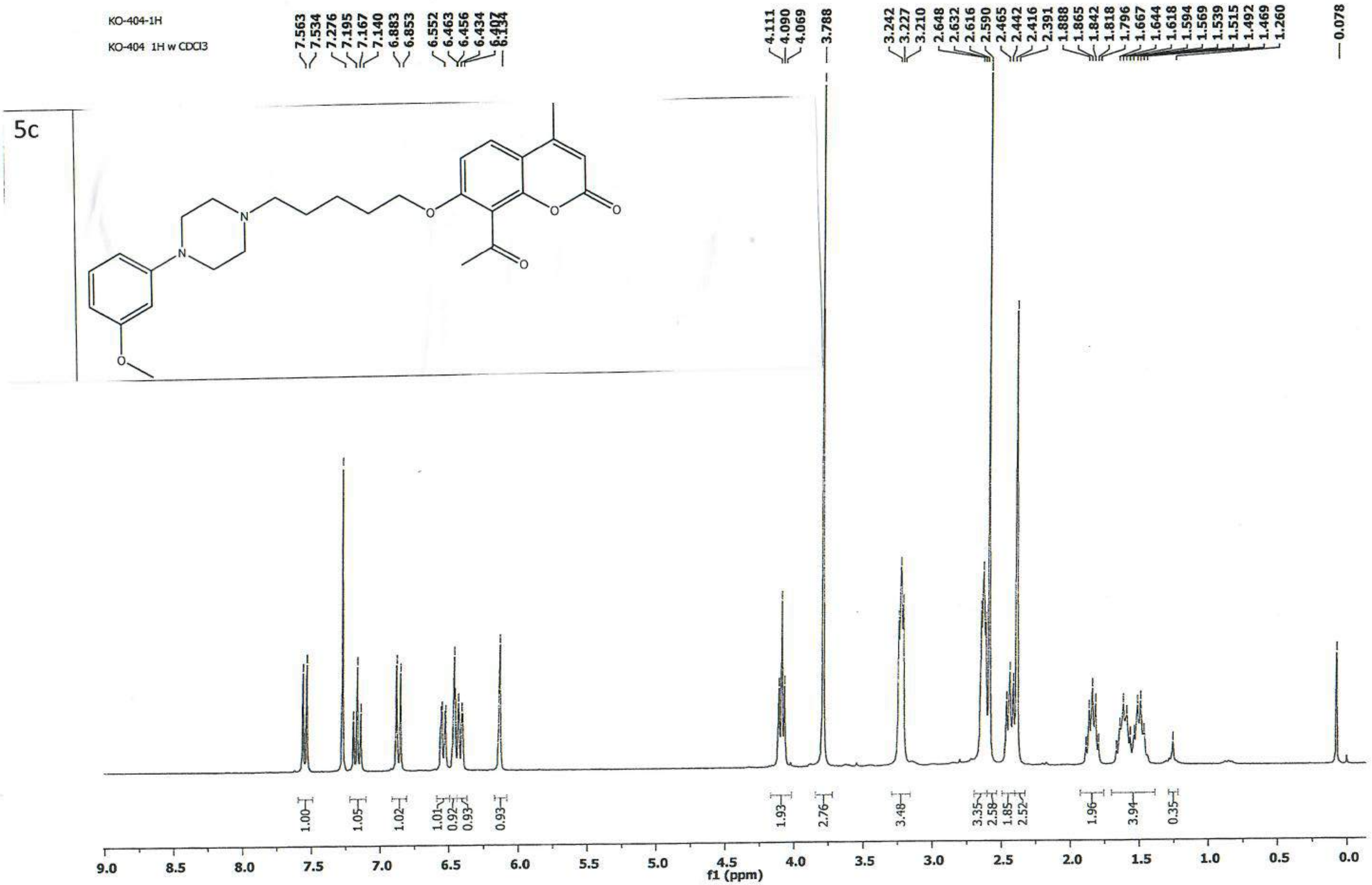
KO-404-1H
KO-404 1H w CDCl3
5c
7.563
7.534
7.276
7.195
7.167
7.140
6.883
6.853
6.552
6.463
6.456
6.434
6.407
6.134
4.111
4.090
4.069
3.788
3.242
3.227
3.210
2.648
2.632
2.616
2.590
2.465
2.442
2.416
2.391
1.888
1.865
1.842
1.818
1.796
1.667
1.644
1.618
1.594
1.569
1.539
1.515
1.492
1.469
1.260
0.078
1.00
1.05
1.02
1.01
0.92
0.93
0.93
1.93
2.76
3.48
3.35
2.58
1.85
2.52
1.96
3.94
0.35
9.0
8.5
8.0
7.5
7.0
6.5
6.0
5.5
5.0
4.5
4.0
3.5
3.0
2.5
2.0
1.5
1.0
0.5
0.0
f1 (ppm)

5c

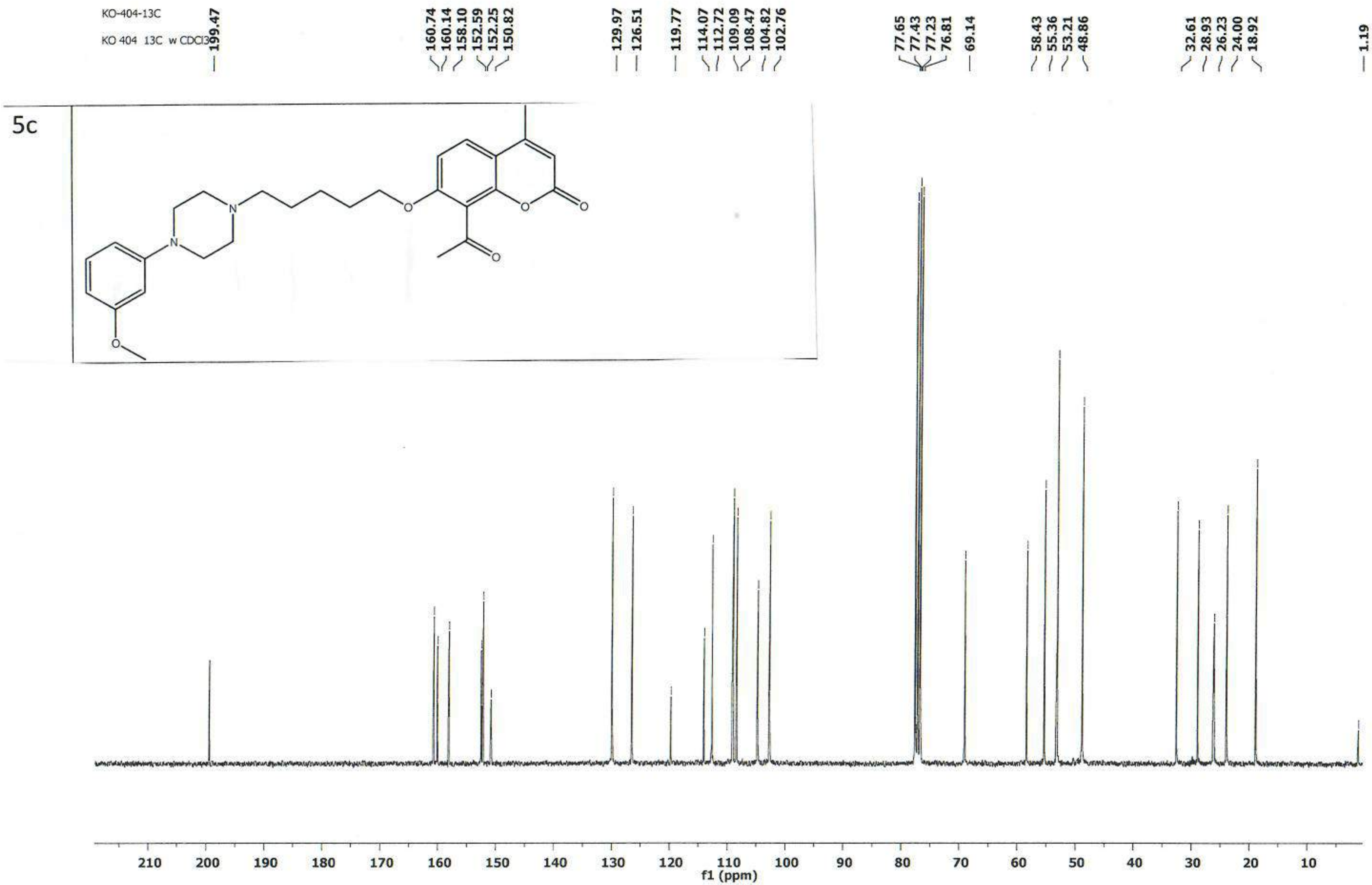
KO-404-13C
KO 404 13C w CDCl3
199.47
160.74
160.14
158.10
152.59
152.25
150.82
129.97
126.51
119.77
114.07
112.72
109.09
108.47
104.82
102.76
77.65
77.43
77.23
76.81
69.14
58.43
55.36
53.21
48.86
32.61
28.93
26.23
24.00
18.92
1.19
5c
210
200
190
180
170
160
150
140
130
120
110
100
90
80
70
60
50
40
30
20
10
f1 (ppm)

5d

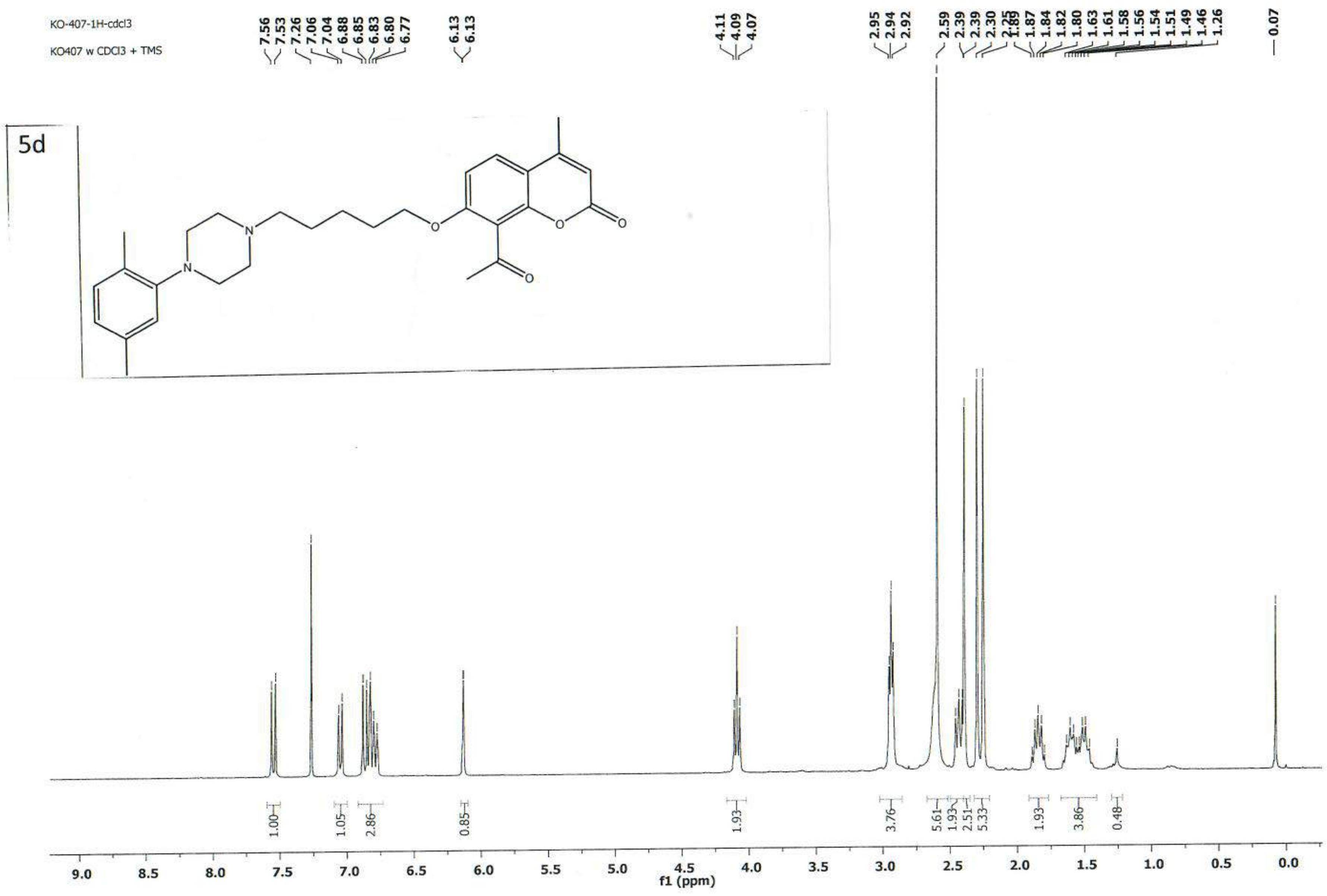
KO-407-1H-cdcl3
KO407 w CDCl3 + TMS
7.56 7.53 7.26 7.06 7.04 6.88 6.85 6.83 6.80 6.77
6.13 6.13
4.11 4.09 4.07
2.95 2.94 2.92
2.59 2.39 2.39 2.30 1.87 1.84 1.82 1.80 1.63 1.61 1.58 1.56 1.54 1.51 1.49 1.46 1.26
0.07
5d
1.00 1.05 2.86 0.85 1.93 3.76 5.61 1.93 2.51 5.33 1.93 3.86 0.48
9.0 8.5 8.0 7.5 7.0 6.5 6.0 5.5 5.0 4.5 4.0 3.5 3.0 2.5 2.0 1.5 1.0 0.5 0.0
f1 (ppm)

5d

KO-407-13C-cdcl3

KO-407 w CDCl3 13C-NMR

5d

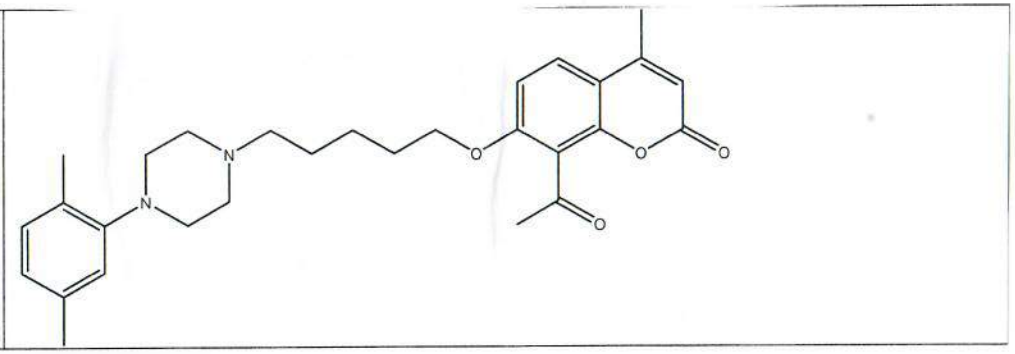

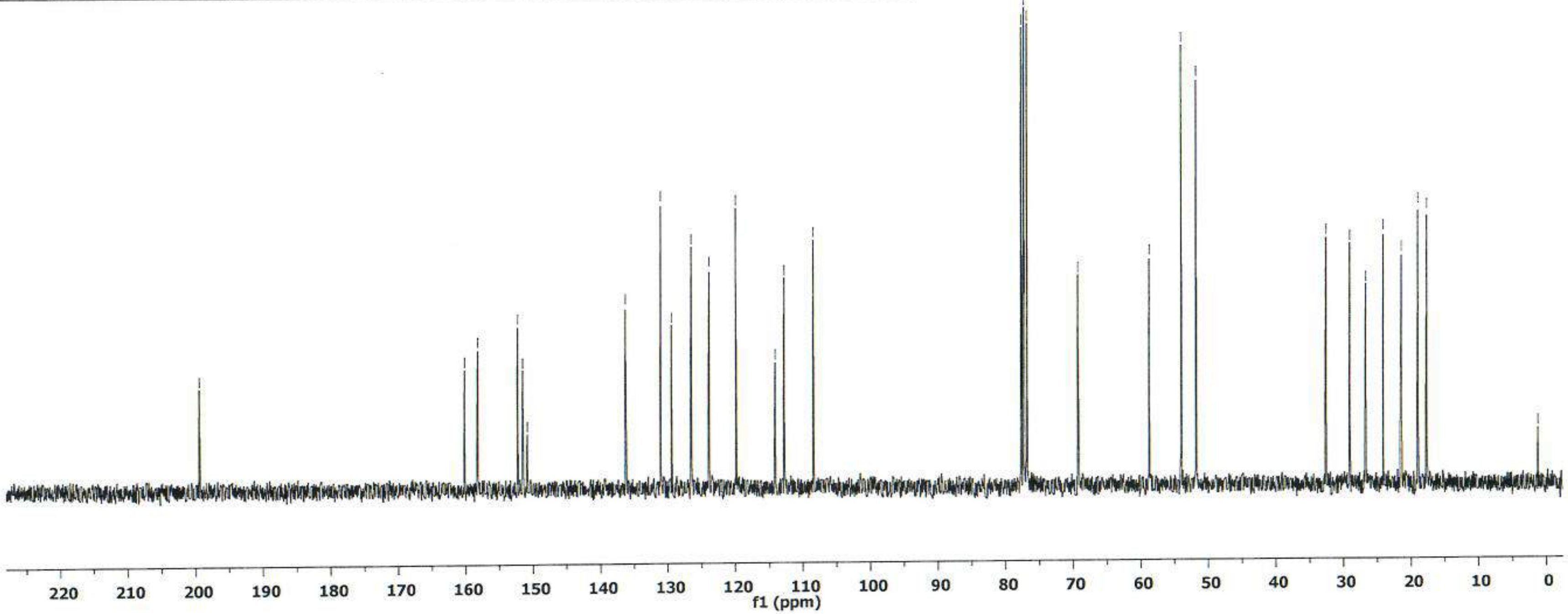

5e

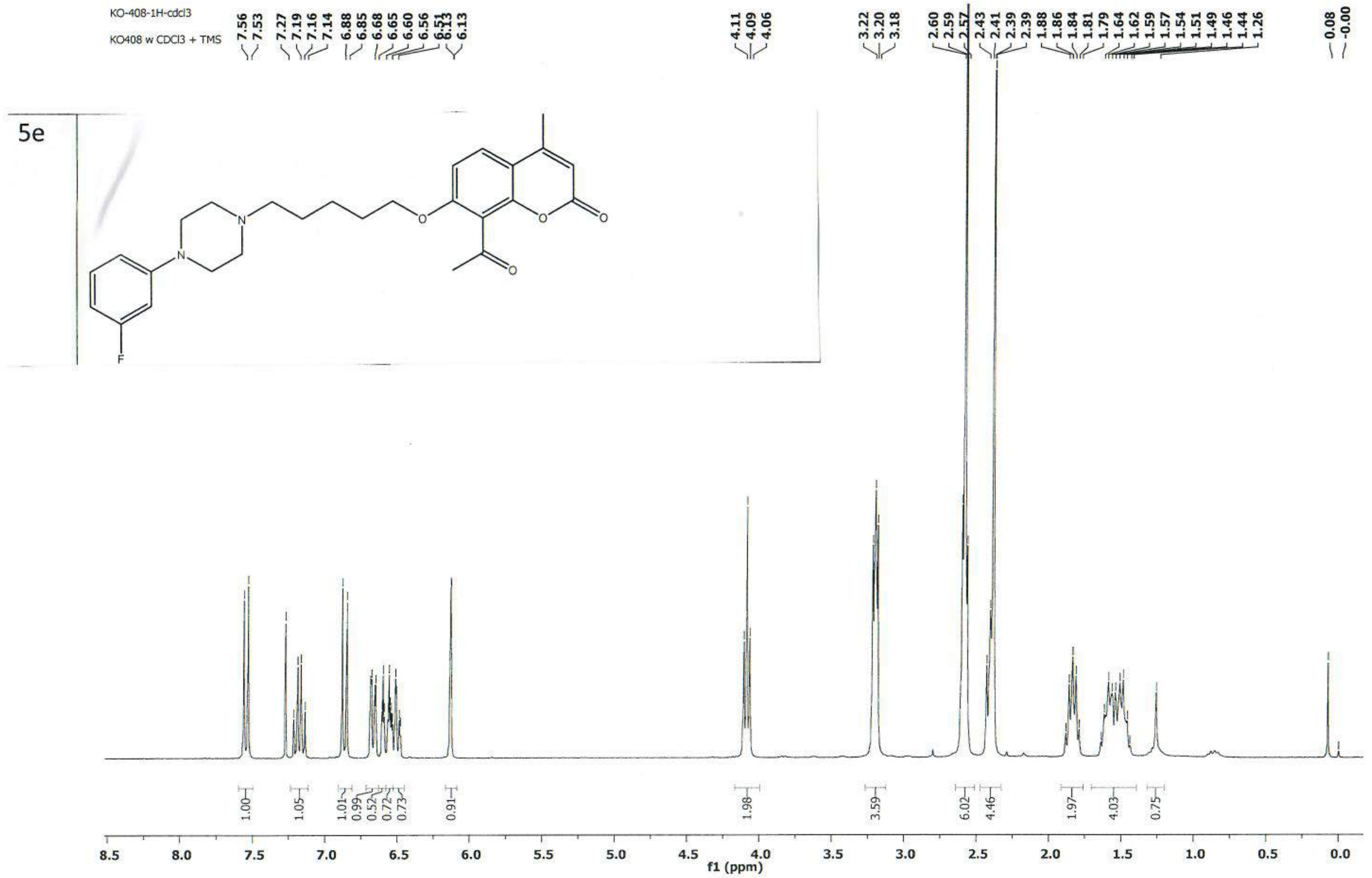

KO-408-1H-cdcl3
KO408 w CDCl3 + TMS
7.56 7.53 7.27 7.19 7.16 7.14 6.88 6.85 6.68 6.65 6.60 6.56 6.51 6.13 6.13
4.11 4.09 4.06
3.22 3.20 3.18
2.60 2.59 2.57 2.43 2.41 2.39 2.39 1.88 1.86 1.84 1.81 1.79 1.64 1.62 1.59 1.57 1.54 1.51 1.49 1.46 1.44 1.26
0.08 -0.00
5e
O
O
O
N
N
O
F
1.00 1.05 1.01 0.99 0.52 0.72 0.73 0.91 1.98 3.59 6.02 4.46 1.97 4.03 0.75
8.5 8.0 7.5 7.0 6.5 6.0 5.5 5.0 4.5 4.0 3.5 3.0 2.5 2.0 1.5 1.0 0.5 0.0
f1 (ppm)

5e

5e

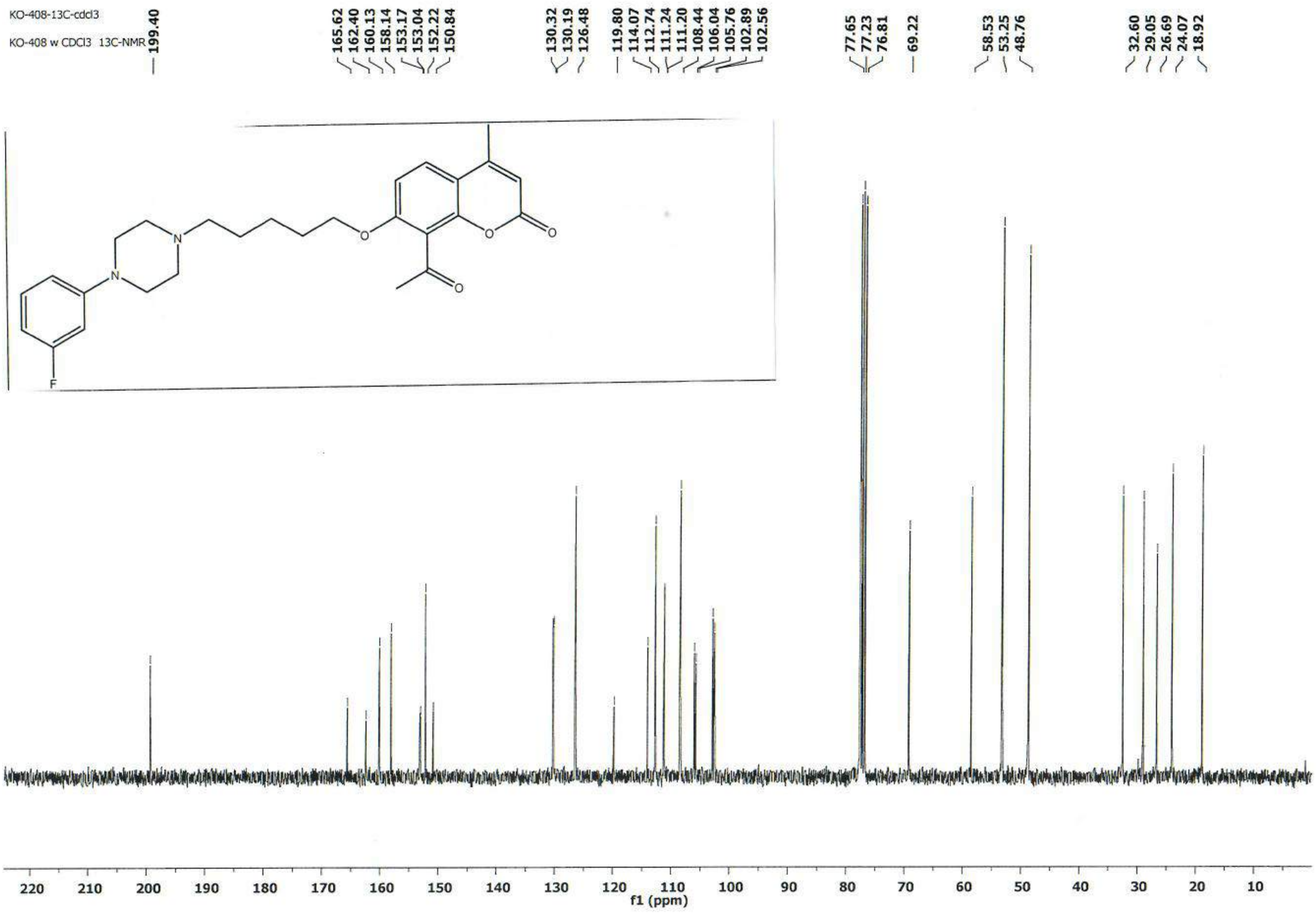
KO-408-13C-cdcl3
KO-408 w CDCl3 13C-NMR
199.40
165.62
162.40
160.13
158.14
153.17
153.04
152.22
150.84
130.32
130.19
126.48
119.80
114.07
112.74
111.24
111.20
108.44
106.04
105.76
102.89
102.56
77.65
77.23
76.81
69.22
58.53
53.25
48.76
32.60
29.05
26.69
24.07
18.92
N
N
O
O
O
O
F
220
210
200
190
180
170
160
150
140
130
120
110
100
90
80
70
60
50
40
30
20
10
f1 (ppm)

5f

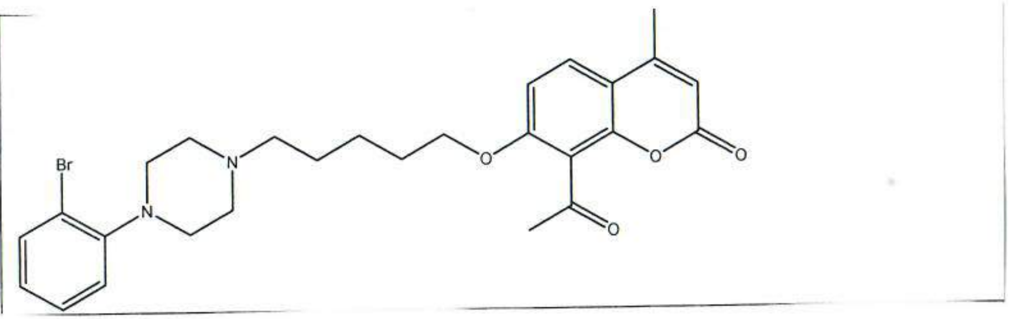

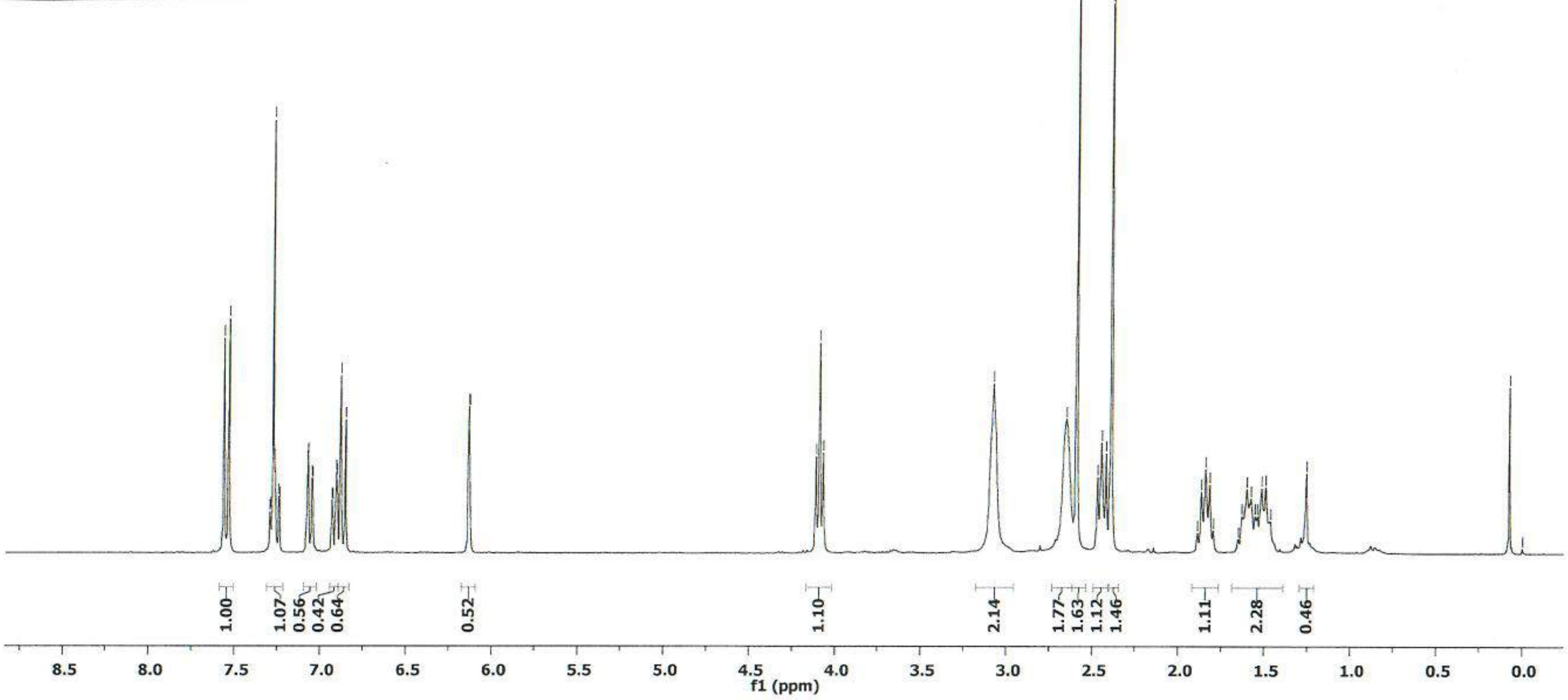
KO-409-1H-cdcl3
KO409 w CDCl3 + TMS
7.56
7.53
7.27
7.27
7.07
7.07
7.05
7.04
6.90
6.90
6.88
6.85
6.13
6.13
4.11
4.09
4.07
3.08
2.65
2.59
2.47
2.45
2.42
2.39
2.39
1.89
1.87
1.84
1.82
1.80
1.65
1.63
1.60
1.58
1.55
1.54
1.51
1.49
1.46
1.26
0.08
-0.00
1.00
1.07
0.56
0.42
0.64
0.52
1.10
2.14
1.77
1.63
1.12
1.46
1.11
2.28
0.46
8.5
8.0
7.5
7.0
6.5
6.0
5.5
5.0
4.5
4.0
3.5
3.0
2.5
2.0
1.5
1.0
0.5
0.0
f1 (ppm)

5f

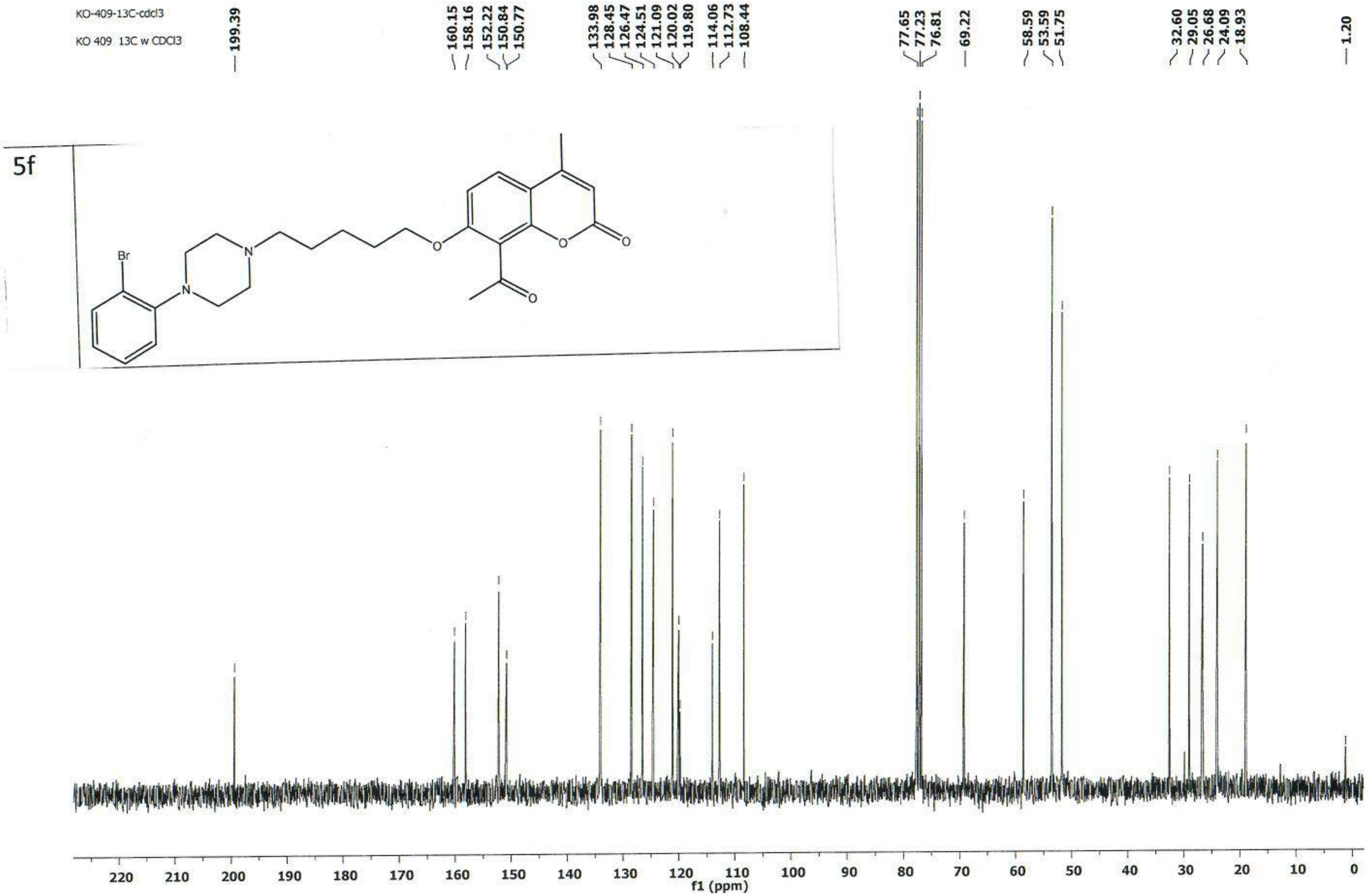
KO-409-13C-cdcl3
KO 409 13C w CDCl3
199.39
160.15
158.16
152.22
150.84
150.77
133.98
128.45
126.47
124.51
121.09
120.02
119.80
114.06
112.73
108.44
77.65
77.23
76.81
69.22
58.59
53.59
51.75
32.60
29.05
26.68
24.09
18.93
1.20
Br
N
N
O
O
O
O
220 210 200 190 180 170 160 150 140 130 120 110 100 90 80 70 60 50 40 30 20 10 0
f1 (ppm)

5g

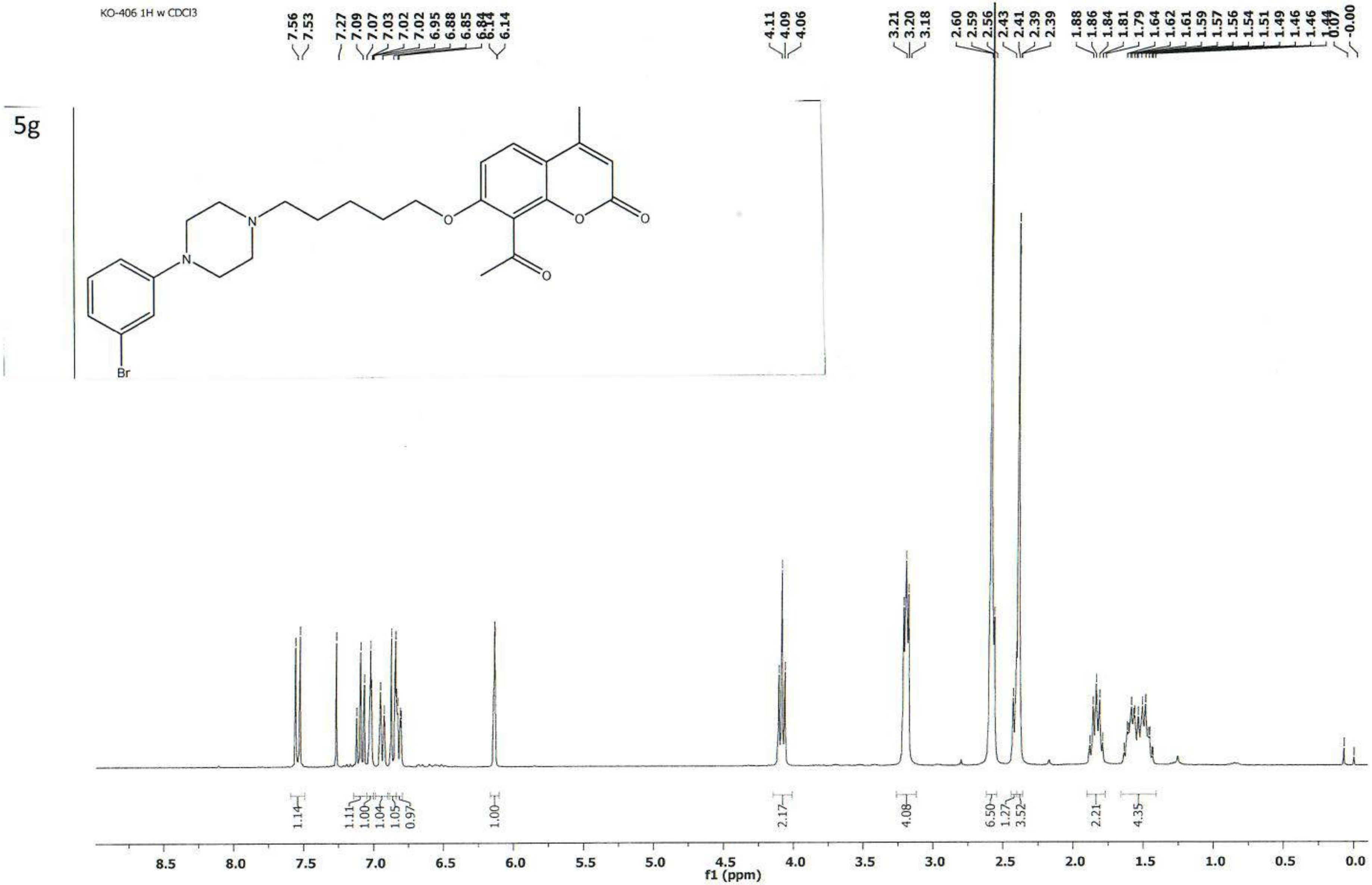
KO-406 1H w CDCl3
5g
Br
N
N
O
O
O
O
7.56
7.53
7.27
7.09
7.07
7.03
7.02
7.02
6.95
6.88
6.85
6.84
6.14
6.14
4.11
4.09
4.06
3.21
3.20
3.18
2.60
2.59
2.56
2.43
2.41
2.39
2.39
1.88
1.86
1.84
1.81
1.79
1.64
1.62
1.61
1.59
1.57
1.56
1.54
1.51
1.49
1.46
1.44
0.07
-0.00
1.14
1.11
1.00
1.04
1.05
0.97
1.00
2.17
4.08
6.50
1.27
3.52
2.21
4.35
f1 (ppm)

5g

KO-406 13C

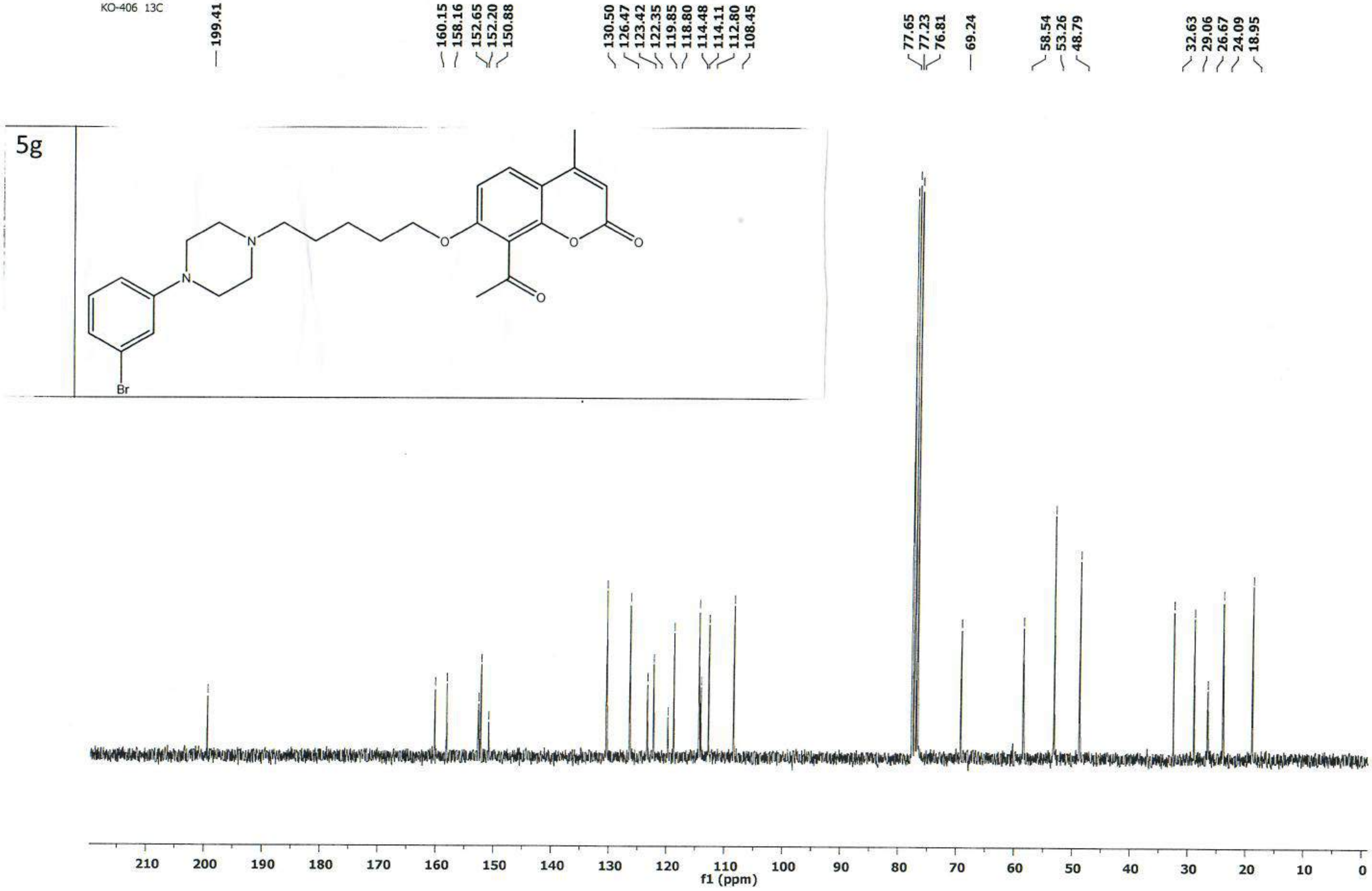

199.41
160.15
158.16
152.65
152.20
150.88
130.50
126.47
123.42
122.35
119.85
118.80
114.48
114.11
112.80
108.45
77.65
77.23
76.81
69.24
58.54
53.26
48.79
32.63
29.06
26.67
24.09
18.95
5g
N
N
O
O
O
O
Br
210
200
190
180
170
160
150
140
130
120
110
100
90
80
70
60
50
40
30
20
10
0
f1 (ppm)

5h

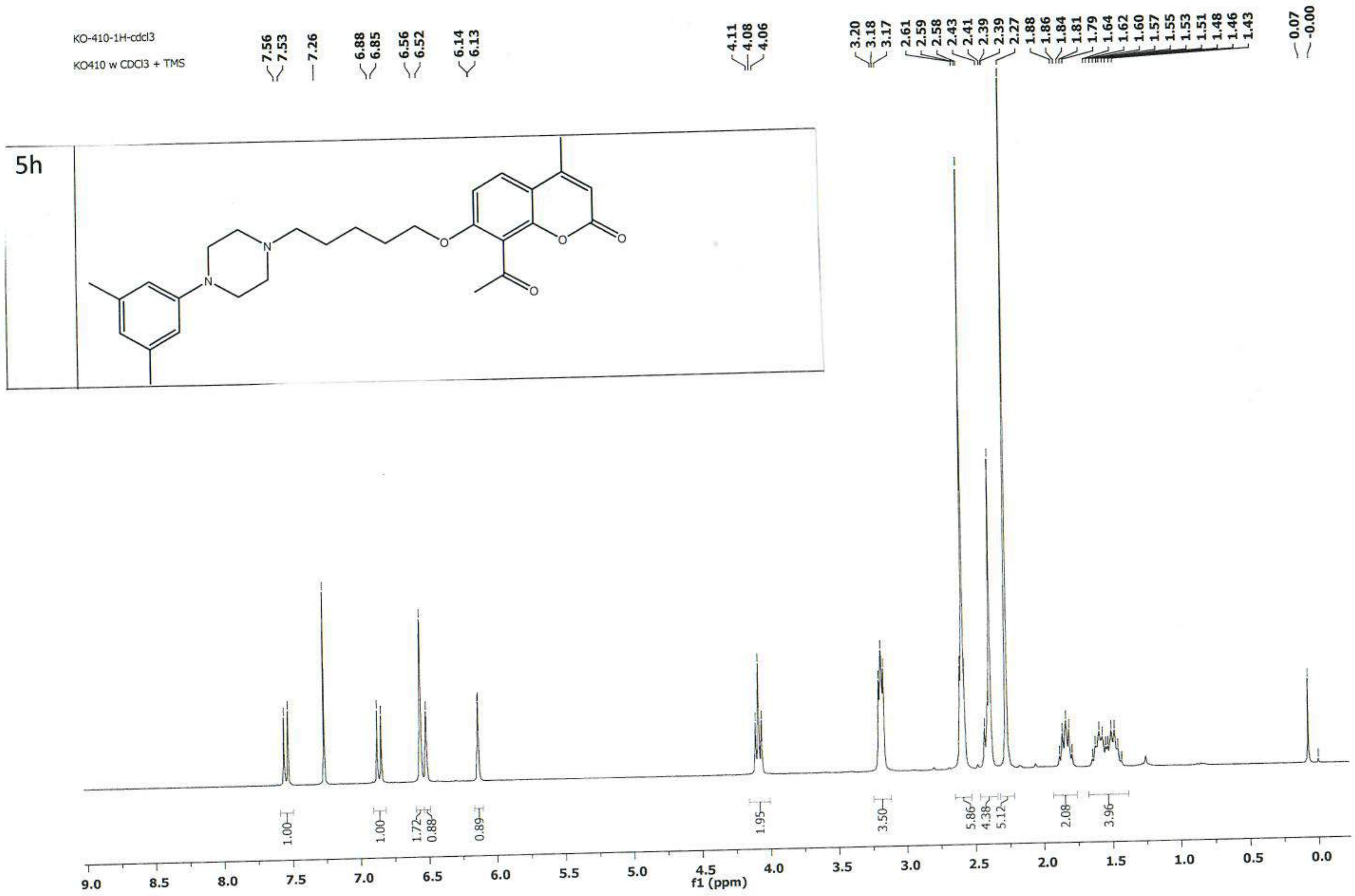
KO-410-1H-cdcl3
KO410 w CDCl3 + TMS
7.56
7.53
7.26
6.88
6.85
6.56
6.52
6.14
6.13
4.11
4.08
4.06
3.20
3.18
3.17
2.61
2.59
2.58
2.43
2.41
2.39
2.39
2.27
1.88
1.86
1.84
1.81
1.79
1.64
1.62
1.60
1.57
1.55
1.53
1.51
1.48
1.46
1.43
0.07
-0.00
5h
N
N
O
O
O
O
1.00
1.00
1.72
0.88
0.89
1.95
3.50
5.86
4.38
5.12
2.08
3.96
9.0
8.5
8.0
7.5
7.0
6.5
6.0
5.5
5.0
4.5
4.0
3.5
3.0
2.5
2.0
1.5
1.0
0.5
0.0
f1 (ppm)

5h

KO-410-13C-cdcl3

KO 410 13C w CDCl3

5h

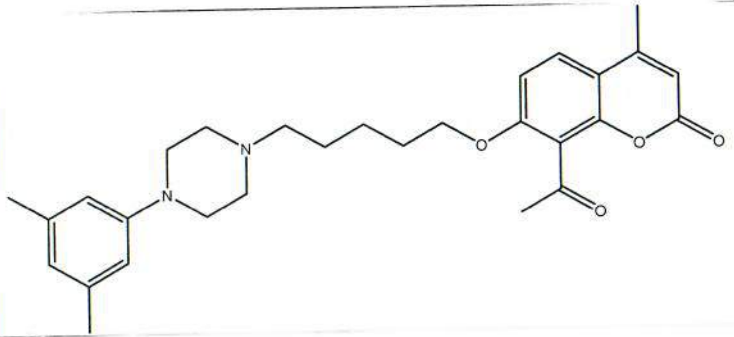

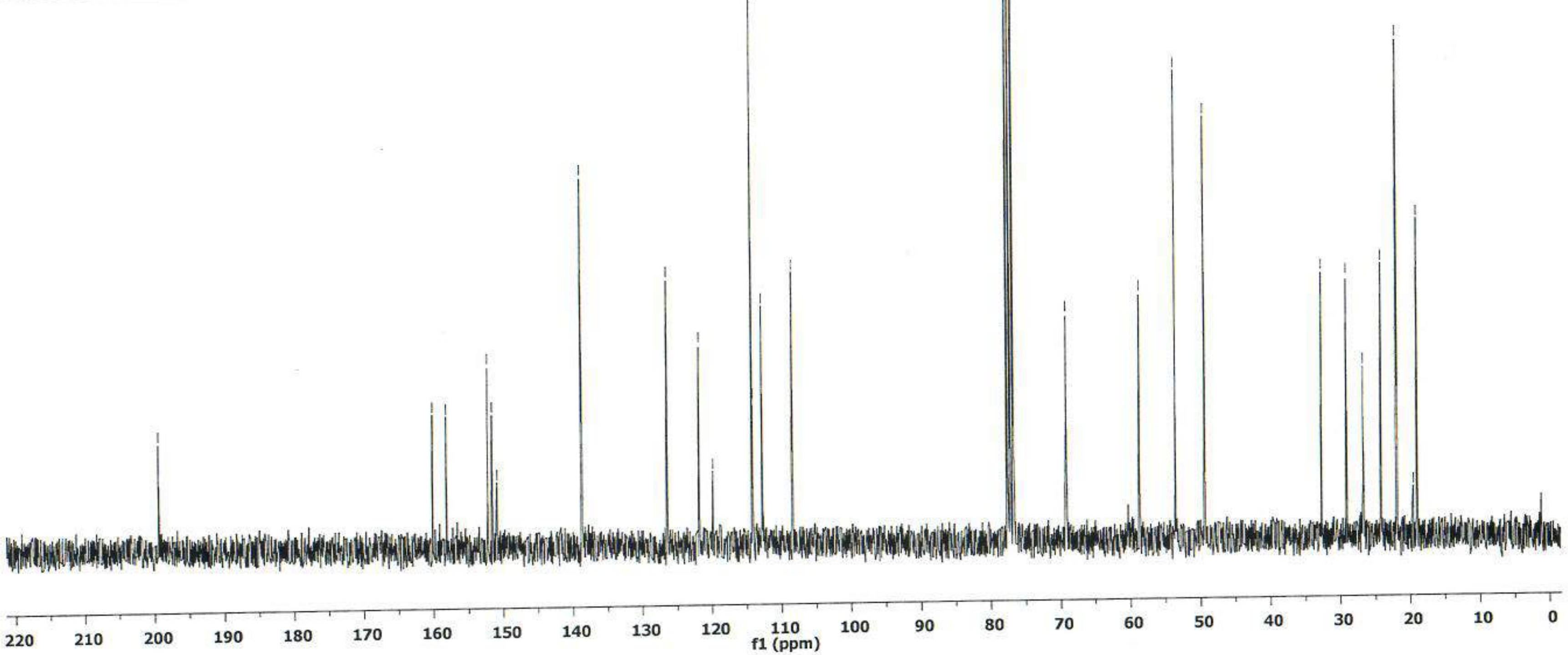

5i

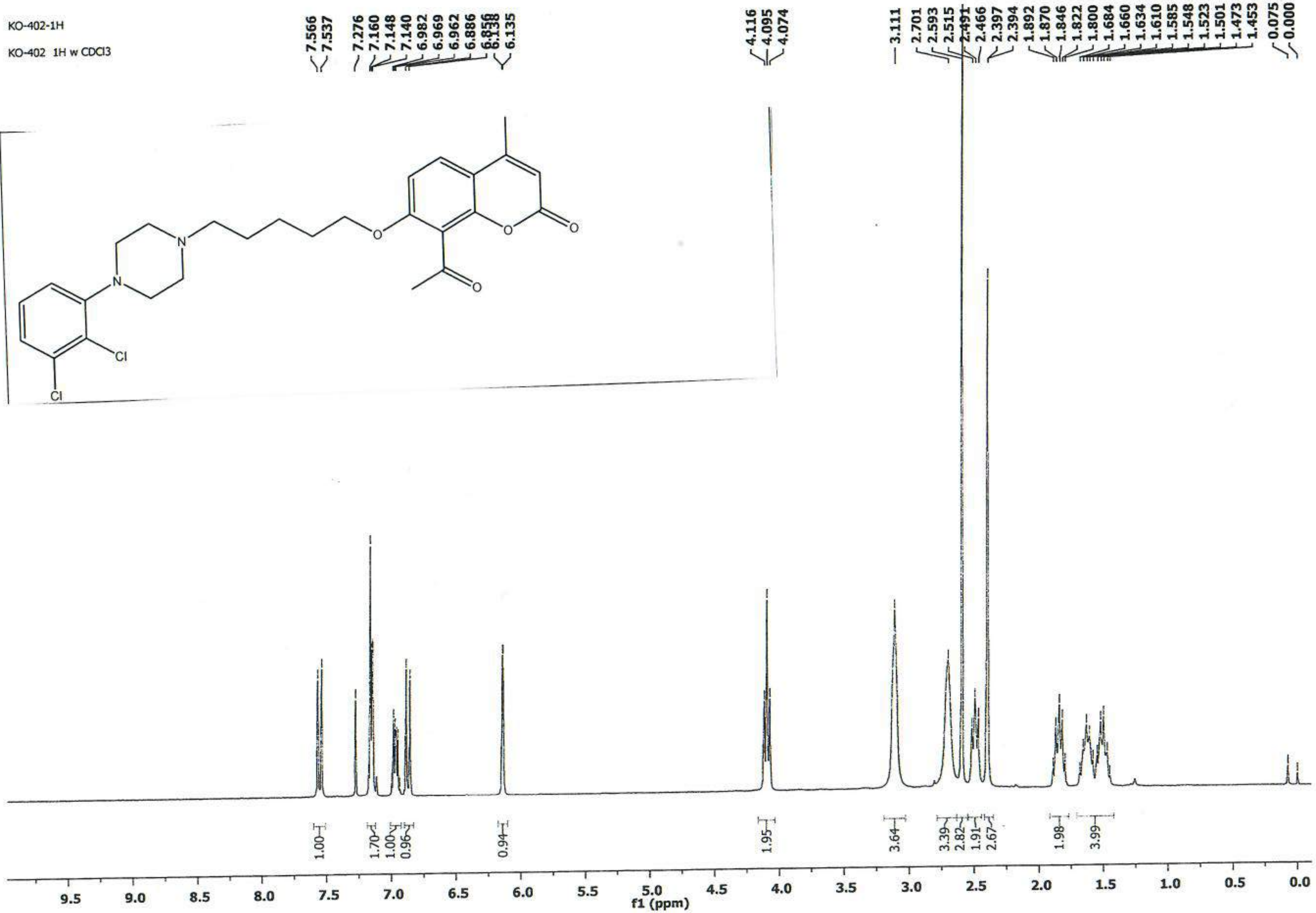
KO-402-1H
KO-402 1H w CDCl3
7.566
7.537
7.276
7.160
7.148
7.140
6.982
6.969
6.962
6.886
6.856
6.138
6.135
4.116
4.095
4.074
3.111
2.701
2.593
2.515
2.491
2.466
2.397
2.394
1.892
1.870
1.846
1.822
1.800
1.684
1.660
1.634
1.610
1.585
1.548
1.523
1.501
1.473
1.453
0.075
0.000
N
N
O
O
O
O
Cl
Cl
1.00
1.70
1.00
0.96
0.94
1.95
3.64
3.39
2.82
1.91
2.67
1.98
3.99
9.5
9.0
8.5
8.0
7.5
7.0
6.5
6.0
5.5
5.0
4.5
4.0
3.5
3.0
2.5
2.0
1.5
1.0
0.5
0.0
f1 (ppm)

5i

5i

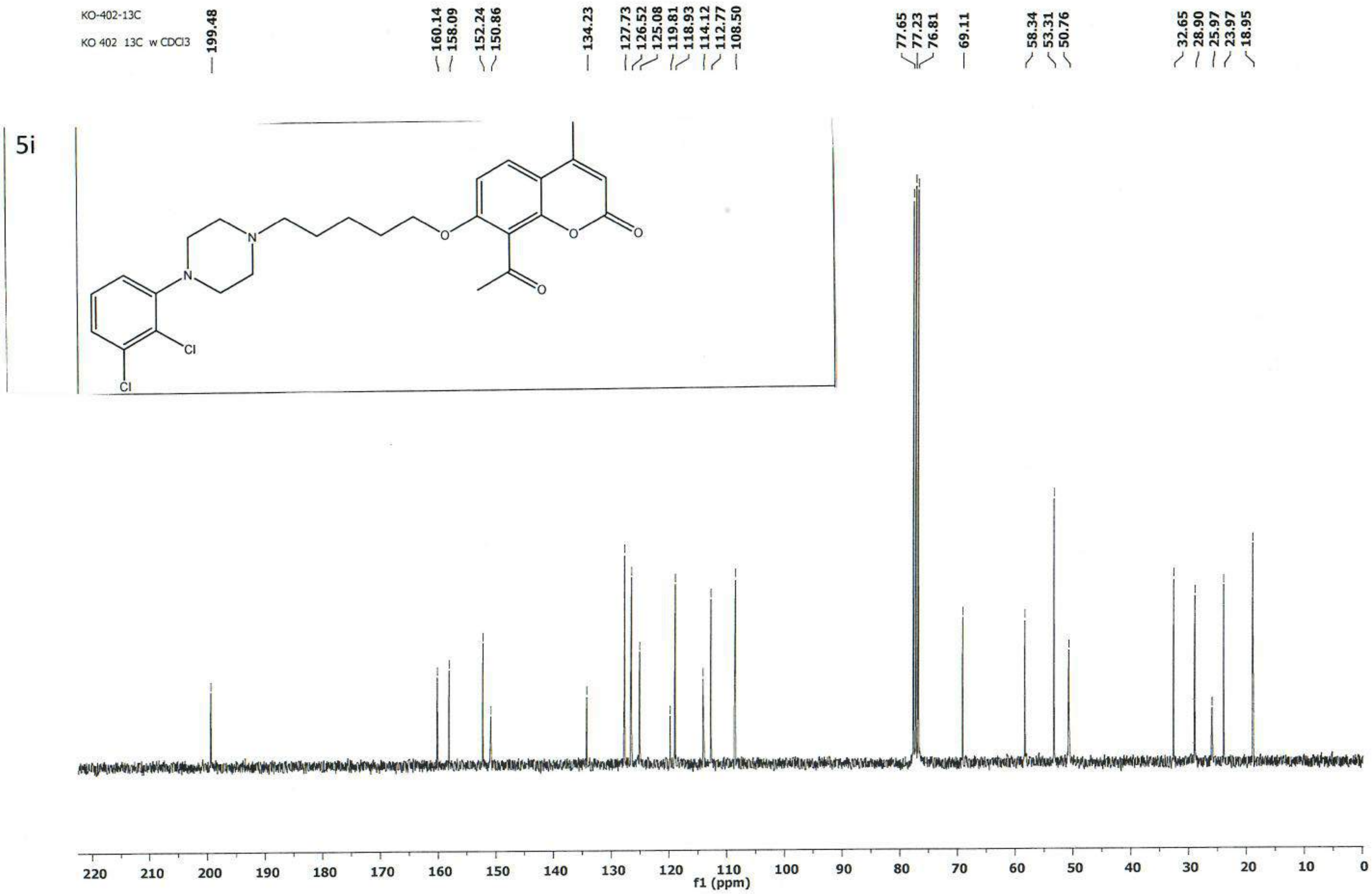
KO-402-13C
KO 402 13C w CDCl3
199.48
160.14
158.09
152.24
150.86
134.23
127.73
126.52
125.08
119.81
118.93
114.12
112.77
108.50
77.65
77.23
76.81
69.11
58.34
53.31
50.76
32.65
28.90
25.97
23.97
18.95
5i
N
N
O
O
O
O
Cl
Cl
220 210 200 190 180 170 160 150 140 130 120 110 100 90 80 70 60 50 40 30 20 10 0
f1 (ppm)

5j

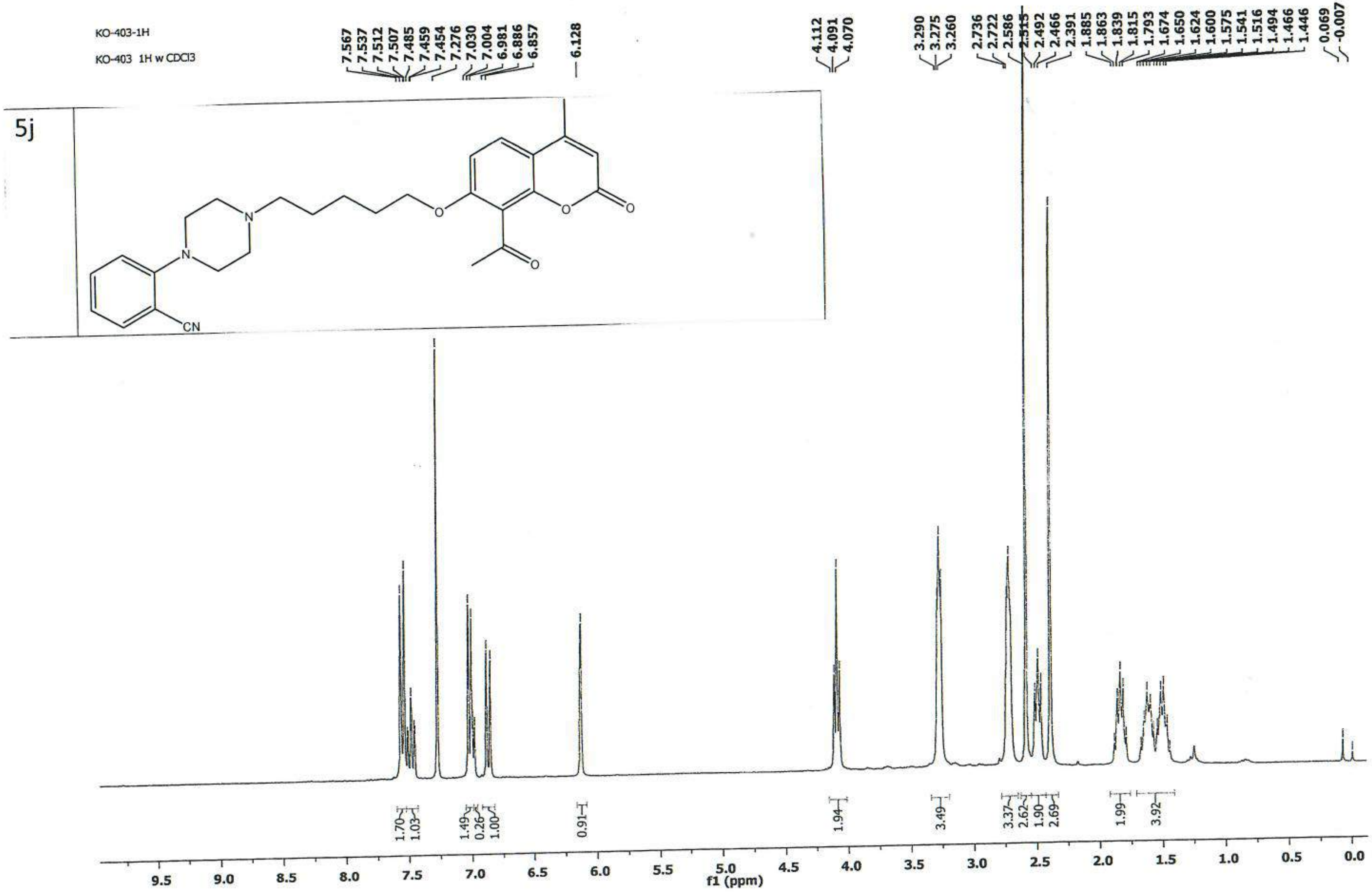
KO-403-1H
KO-403 1H w CDCl3
5j
CN
N
O
7.567
7.537
7.512
7.507
7.485
7.459
7.454
7.276
7.030
7.004
6.981
6.886
6.857
6.128
4.112
4.091
4.070
3.290
3.275
3.260
2.736
2.722
2.586
2.515
2.492
2.466
2.391
1.885
1.863
1.839
1.815
1.793
1.674
1.650
1.624
1.600
1.575
1.541
1.516
1.494
1.466
1.446
0.069
-0.007
1.70
1.03
1.49
0.26
1.00
0.91
1.94
3.49
3.37
2.62
1.90
2.69
1.99
3.92
9.5
9.0
8.5
8.0
7.5
7.0
6.5
6.0
5.5
5.0
4.5
4.0
3.5
3.0
2.5
2.0
1.5
1.0
0.5
0.0
f1 (ppm)

5j

KO-403-13C

KO 403 13C w CDCl3

5j

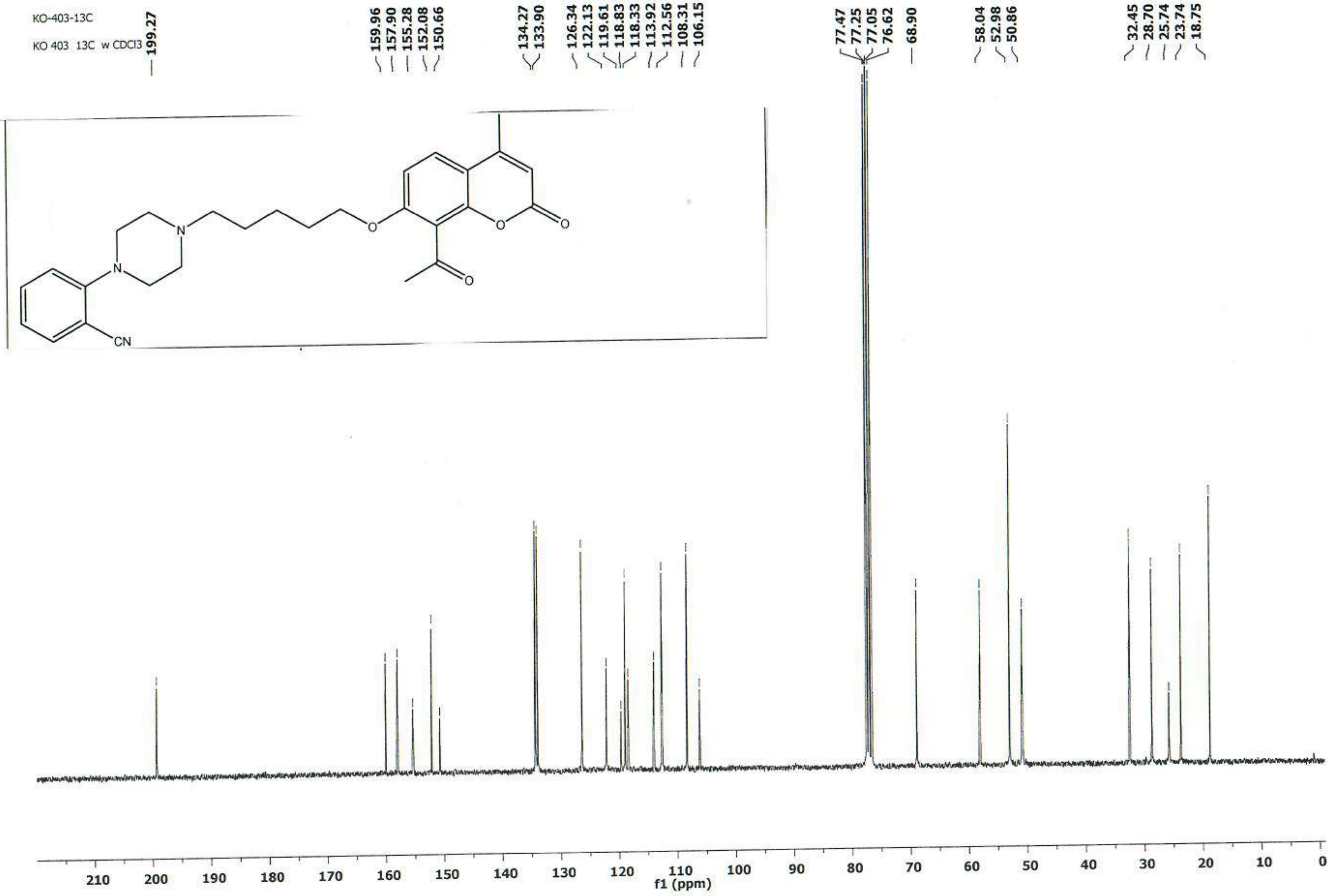

6

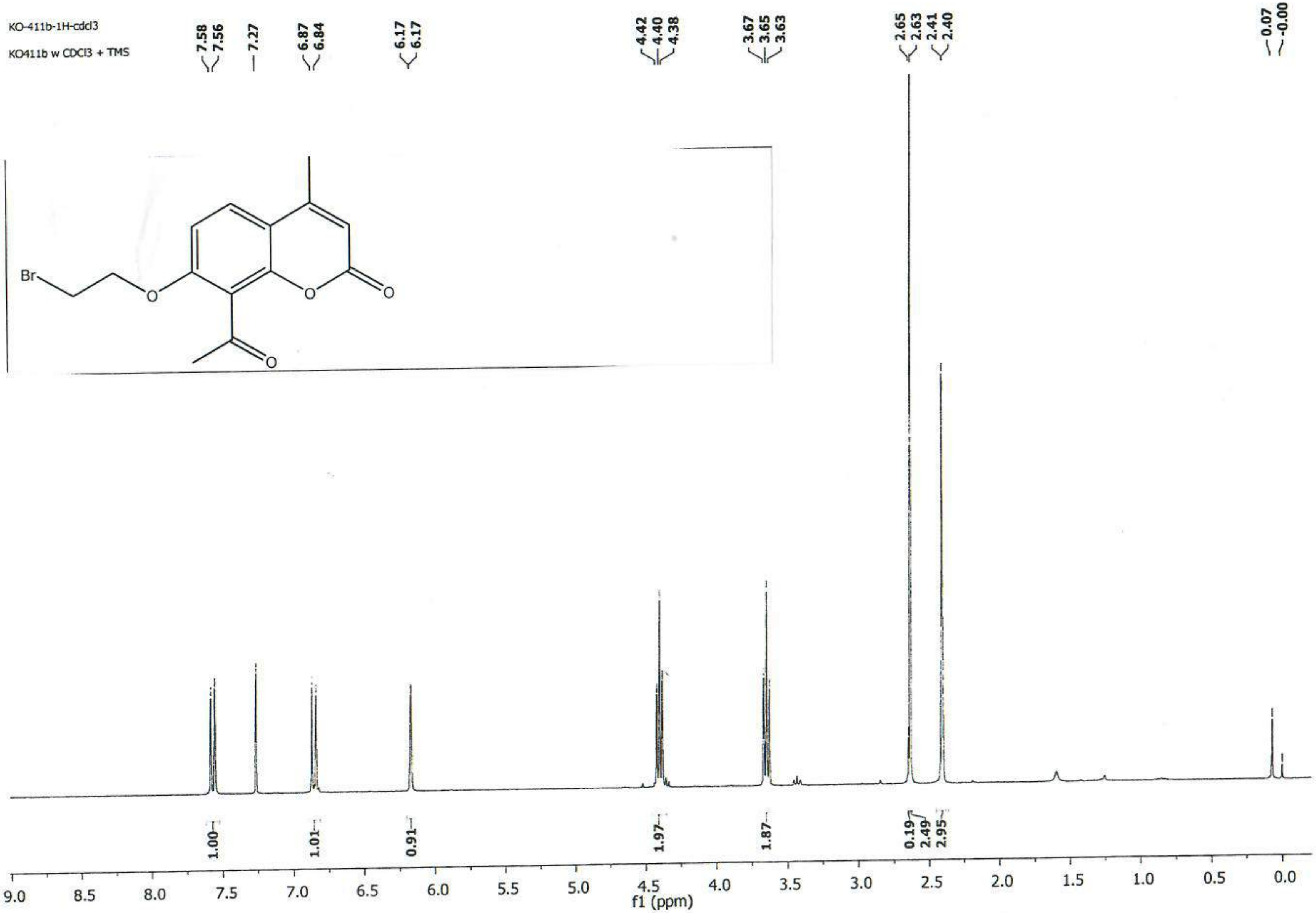
KO-411b-1H-cdcl3
KO411b w CDCl3 + TMS
7.58
7.56
7.27
6.87
6.84
6.17
6.17
4.42
4.40
4.38
3.67
3.65
3.63
2.65
2.63
2.41
2.40
0.07
-0.00
Br
O
O
O
O
1.00
1.01
0.91
1.97
1.87
0.19
2.49
2.95
9.0
8.5
8.0
7.5
7.0
6.5
6.0
5.5
5.0
4.5
4.0
3.5
3.0
2.5
2.0
1.5
1.0
0.5
0.0
f1 (ppm)

6

KO-411b-13C-cdcl3

KO 411b 13C w CDCl3

6

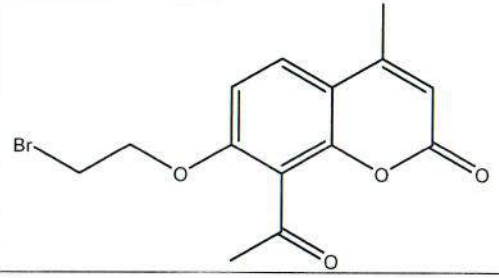

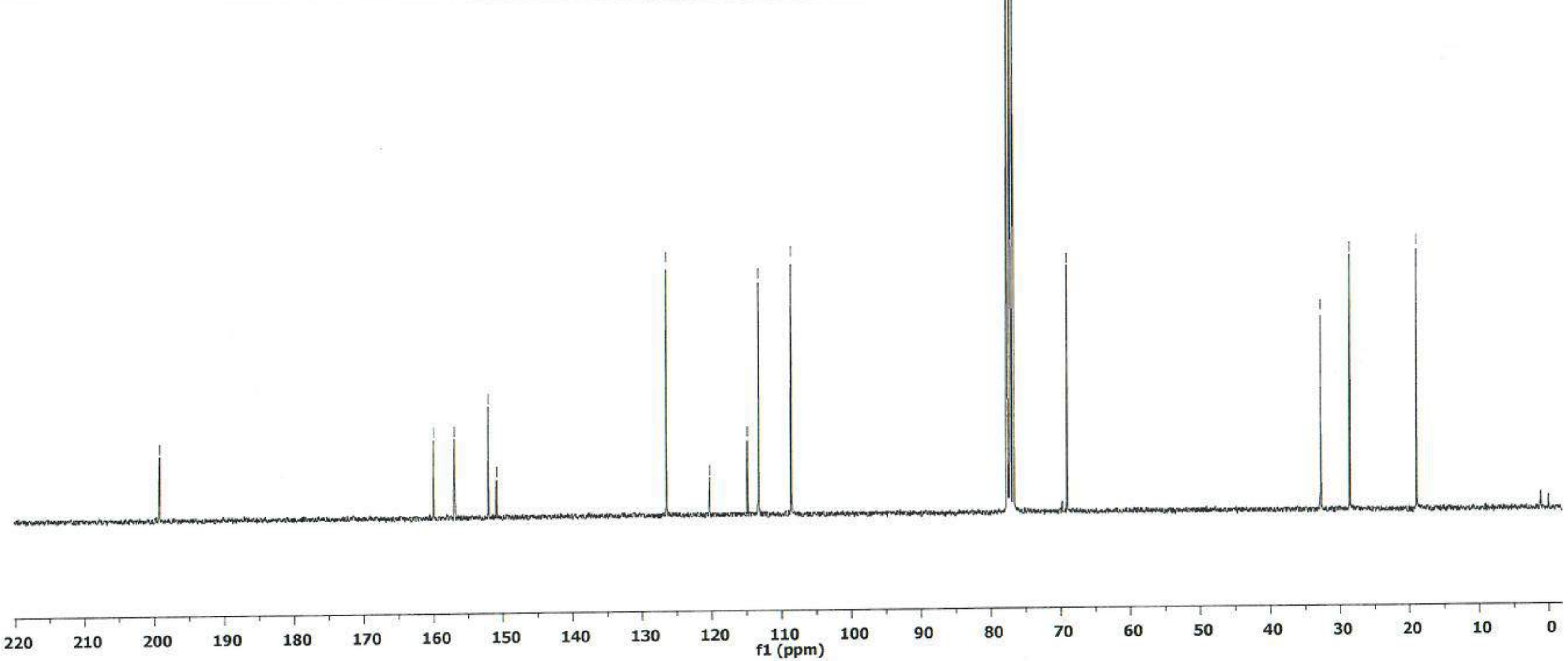

6a

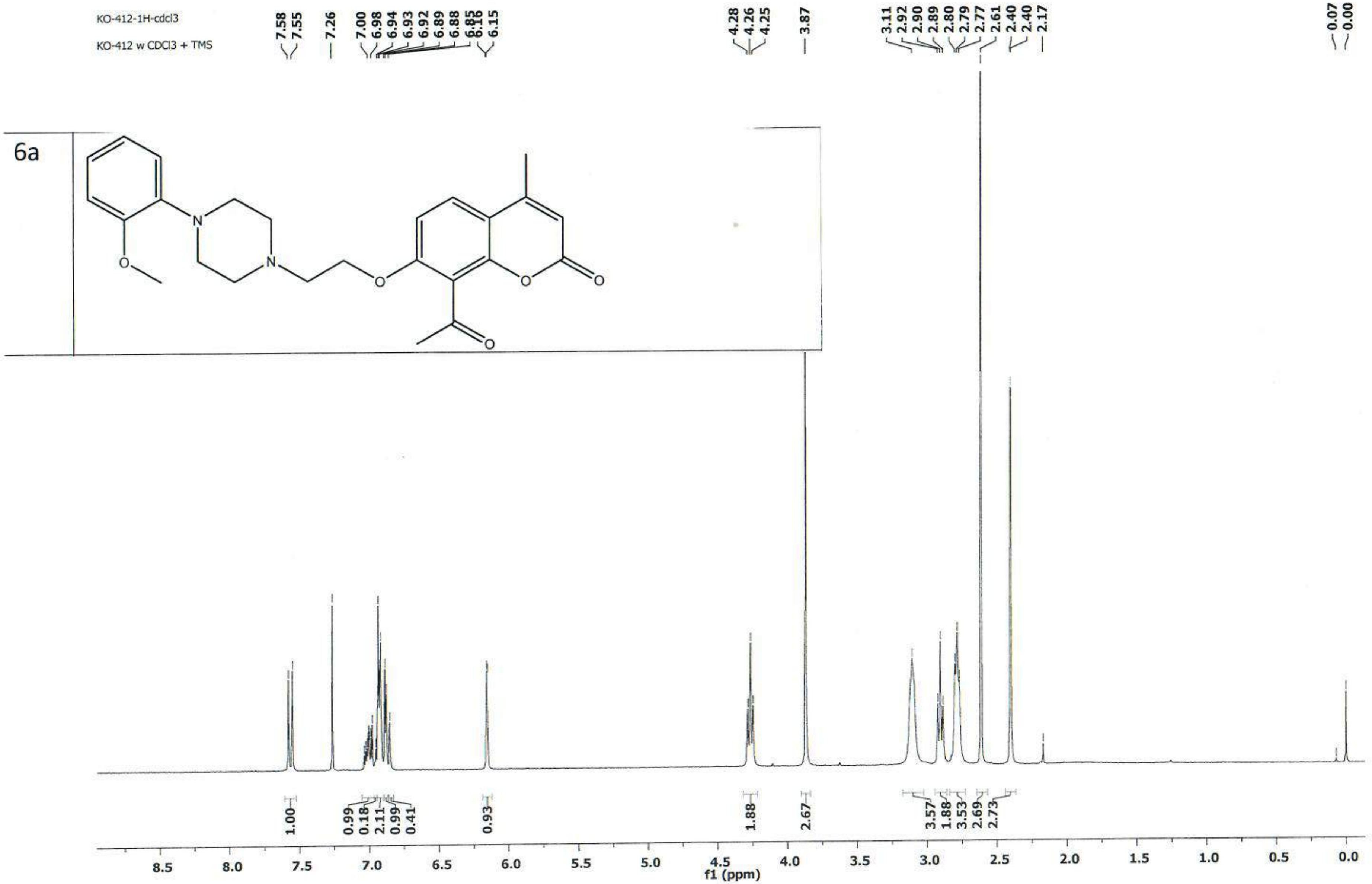
KO-412-1H-cdcl3
KO-412 w CDCl3 + TMS
7.58
7.55
7.26
7.00
6.98
6.94
6.93
6.92
6.89
6.88
6.85
6.16
6.15
4.28
4.26
4.25
3.87
3.11
2.92
2.90
2.89
2.80
2.79
2.77
2.61
2.40
2.40
2.17
0.07
0.00
6a
1.00
0.99
0.18
2.11
0.99
0.41
0.93
1.88
2.67
3.57
1.88
3.53
2.69
2.73
f1 (ppm)

6a

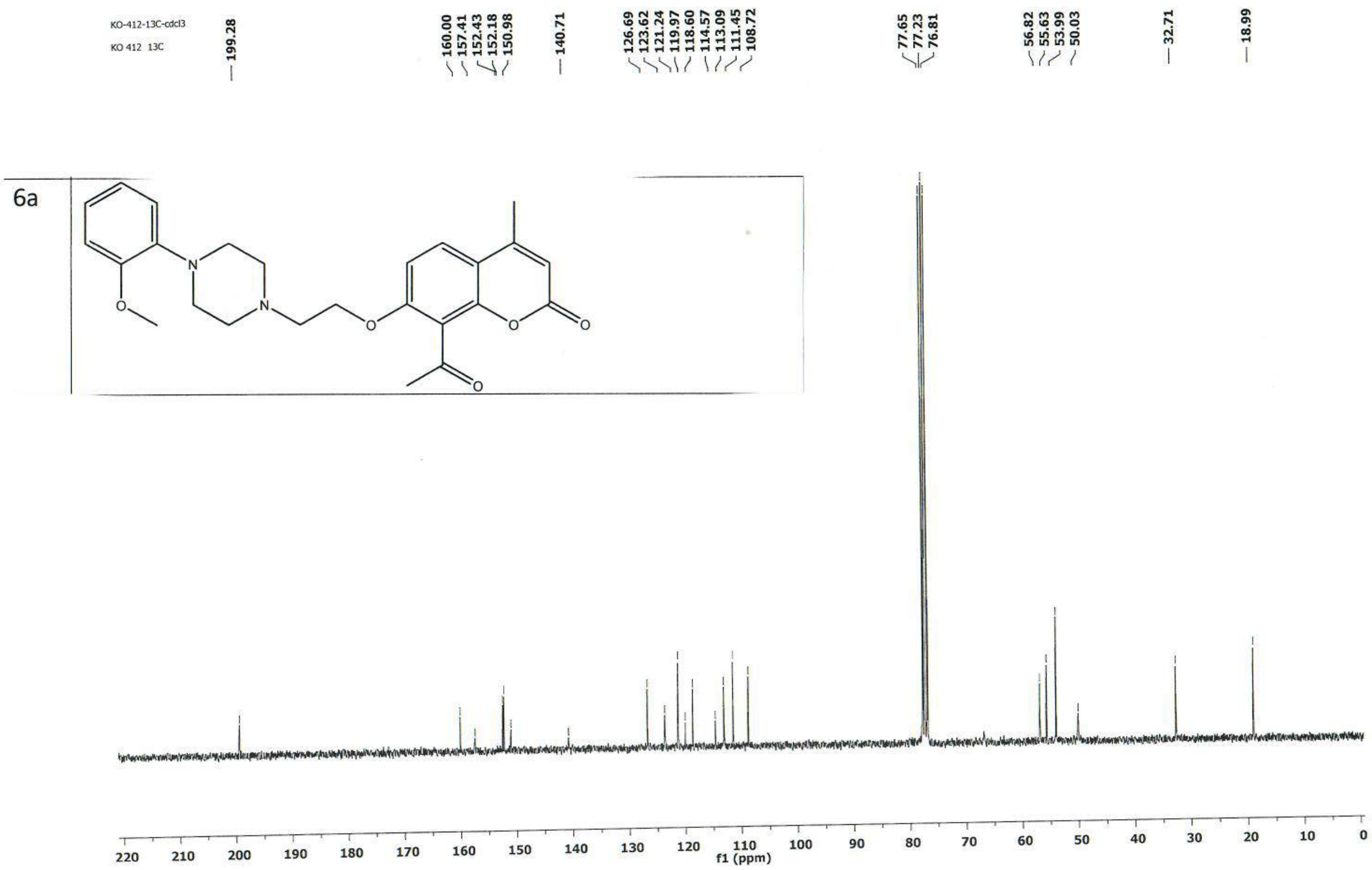
KO-412-13C-cdcl3
KO 412 13C
199.28
160.00
157.41
152.43
152.18
150.98
140.71
126.69
123.62
121.24
119.97
118.60
114.57
113.09
111.45
108.72
77.65
77.23
76.81
56.82
55.63
53.99
50.03
32.71
18.99
6a
220
210
200
190
180
170
160
150
140
130
120
110
f1 (ppm)
100
90
80
70
60
50
40
30
20
10
0

6b

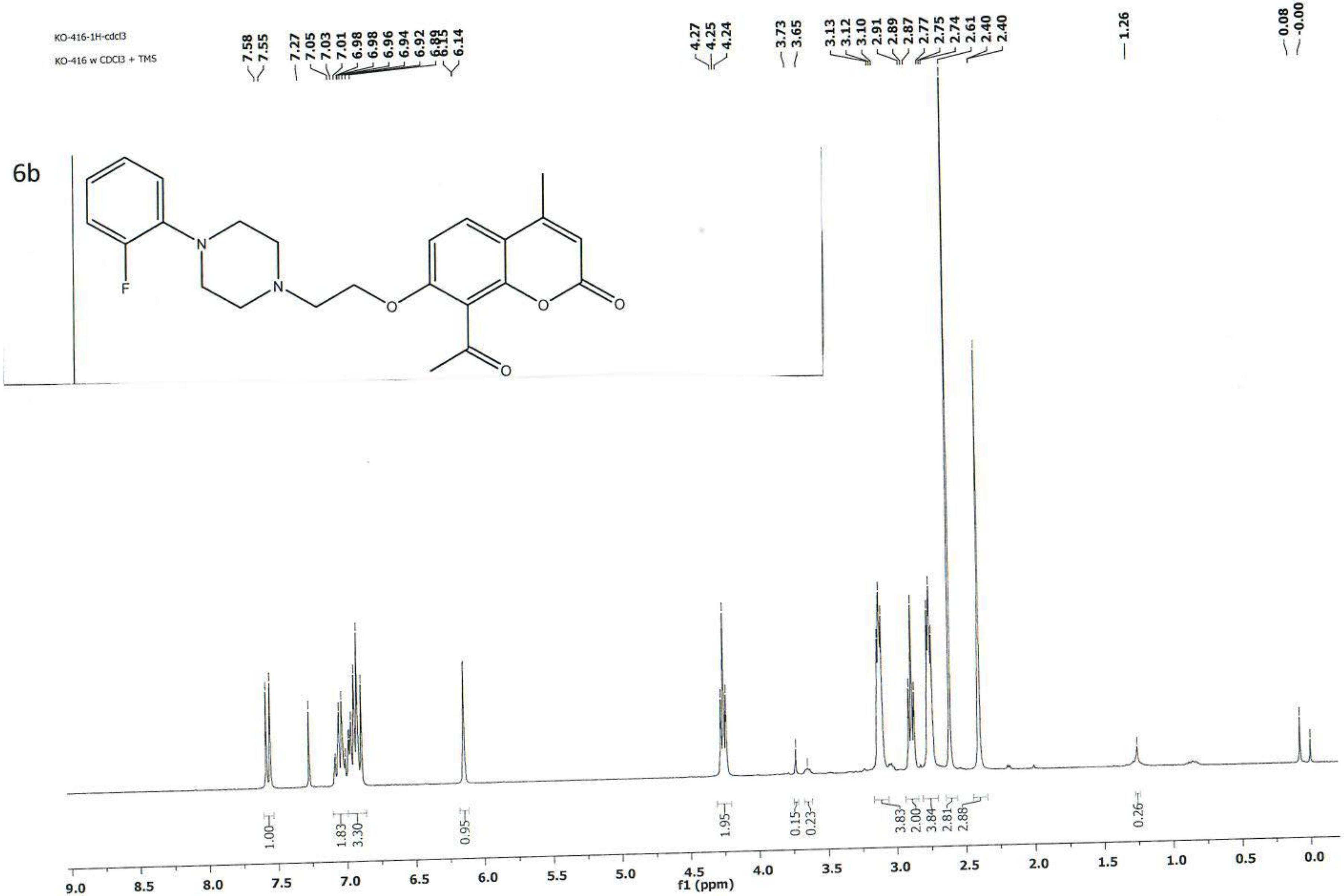
KO-416-1H-cdcl3
KO-416 w CDCl3 + TMS
7.58
7.55
7.27
7.05
7.03
7.01
6.98
6.98
6.96
6.94
6.92
6.89
6.15
6.14
4.27
4.25
4.24
3.73
3.65
3.13
3.12
3.10
2.91
2.89
2.87
2.77
2.75
2.74
2.61
2.40
2.40
1.26
0.08
-0.00
6b
F
N
N
O
O
O
O
1.00
1.83
3.30
0.95
1.95
0.15
0.23
3.83
2.00
3.84
2.81
2.88
0.26
9.0
8.5
8.0
7.5
7.0
6.5
6.0
5.5
5.0
4.5
4.0
3.5
3.0
2.5
2.0
1.5
1.0
0.5
0.0
f1 (ppm)

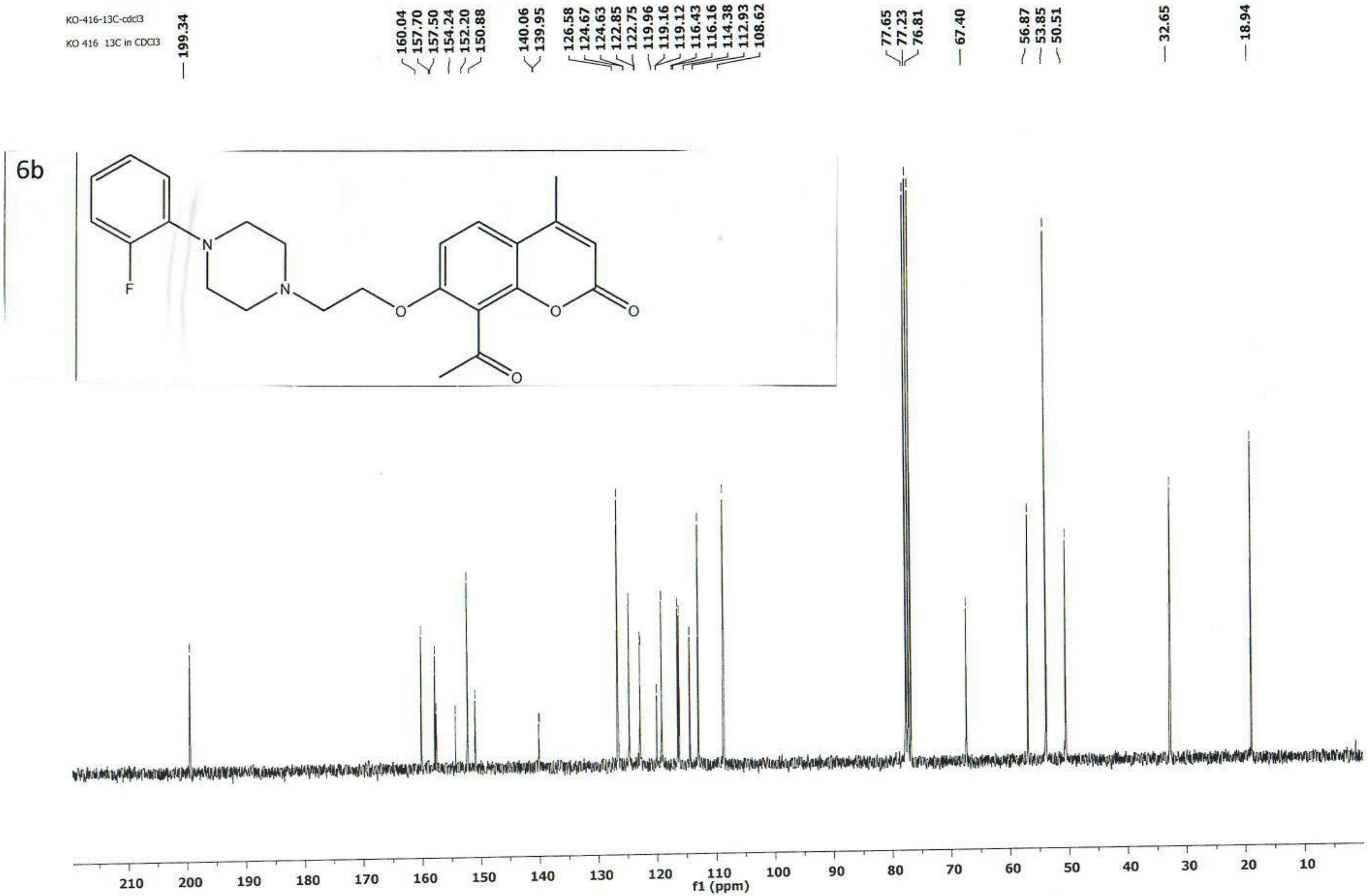
KO-416-13C-cdcl3
KO 416 13C in CDCl3
199.34
160.04
157.70
157.50
154.24
152.20
150.88
140.06
139.95
126.58
124.67
124.63
122.85
122.75
119.96
119.16
119.12
116.43
116.16
114.38
112.93
108.62
77.65
77.23
76.81
67.40
56.87
53.85
50.51
32.65
18.94
6b
N
N
F
O
O
O
O
210
200
190
180
170
160
150
140
130
120
110
f1 (ppm)
100
90
80
70
60
50
40
30
20
10

6c

KO-415-1H-cdcl3

KO-415 w CDCl3 + TMS

6c

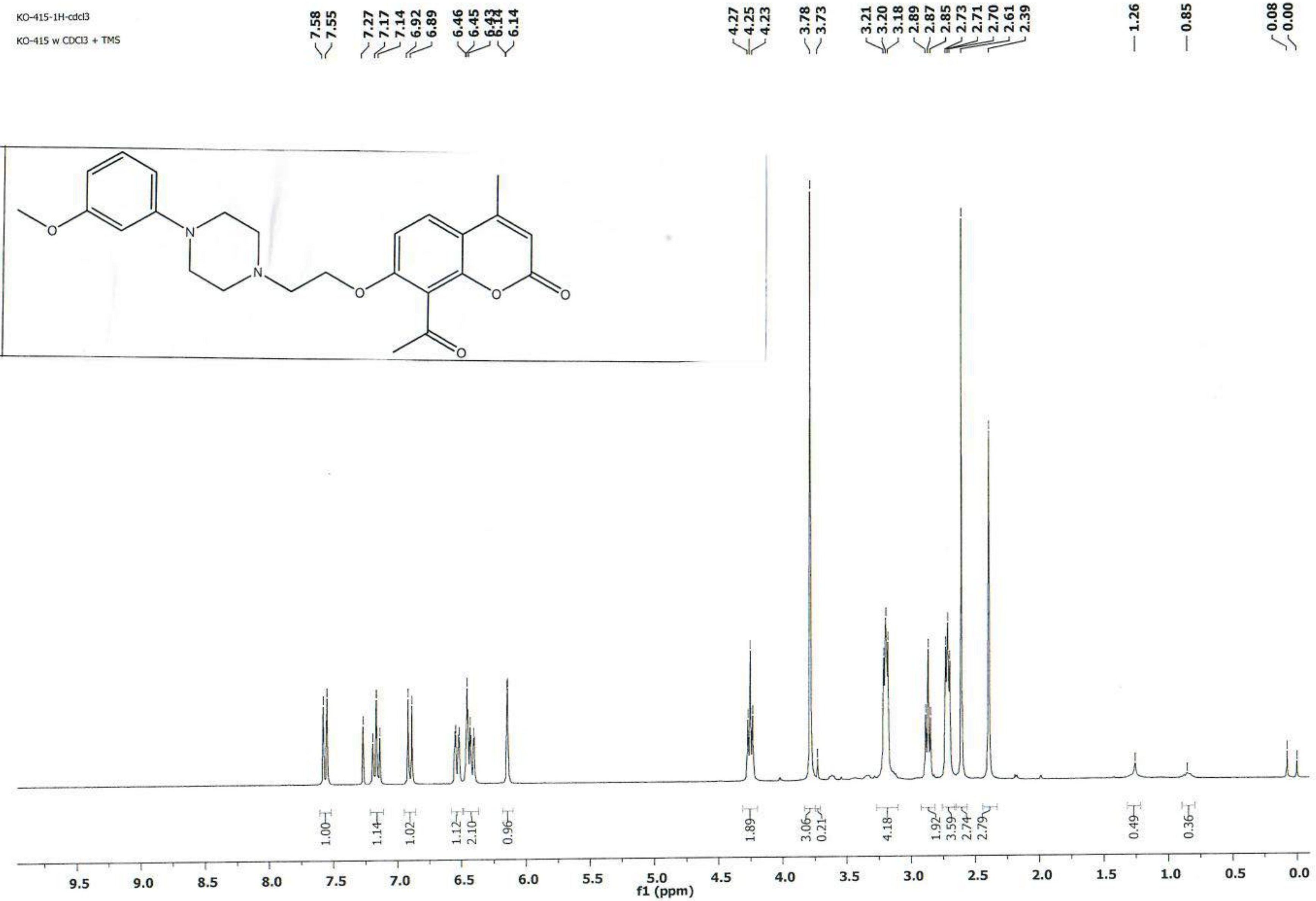

6c

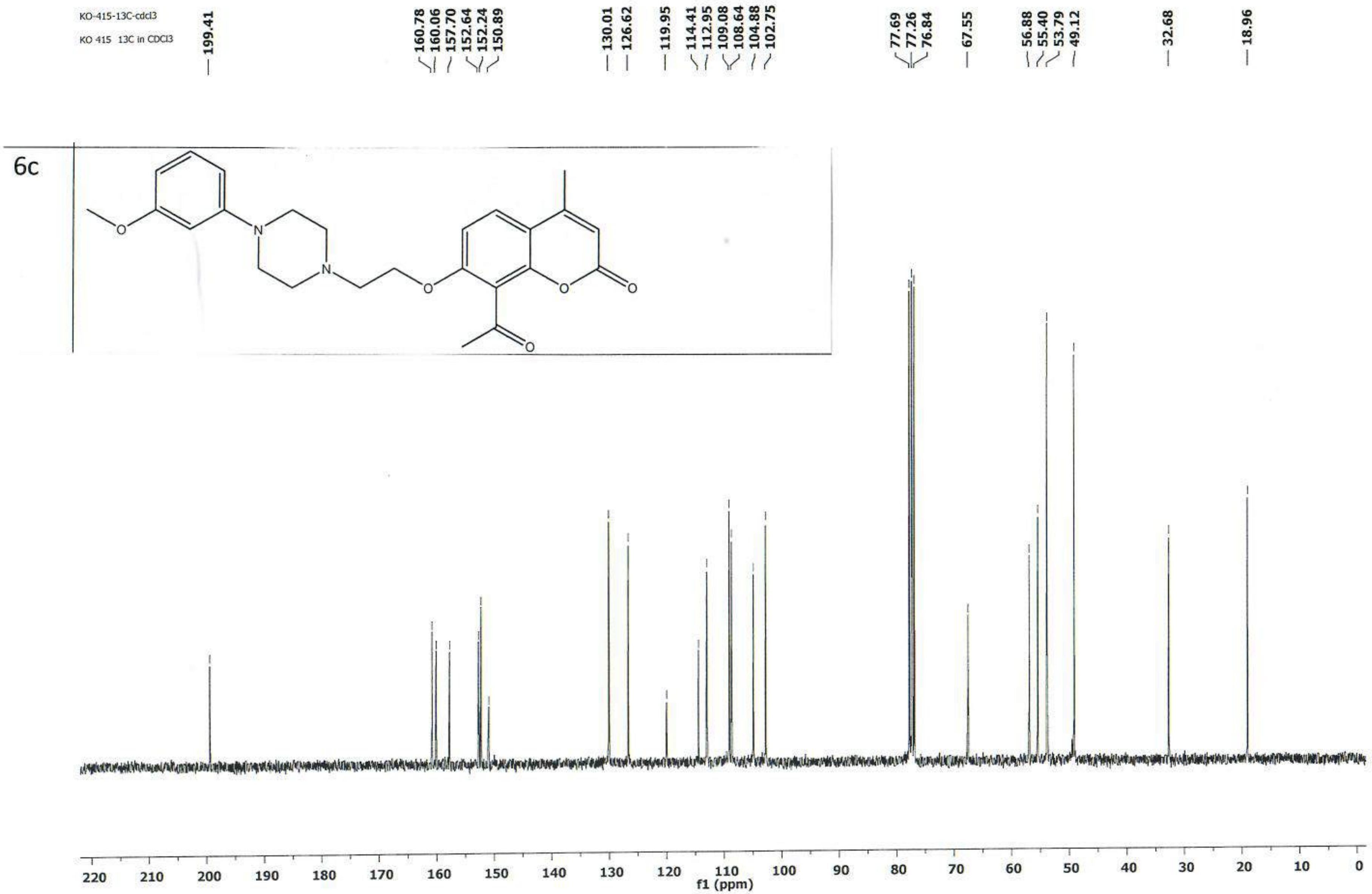

KO-415-13C-cdcl3
KO 415 13C in CDCl3
199.41
160.78
160.06
157.70
152.64
152.24
150.89
130.01
126.62
119.95
114.41
112.95
109.08
108.64
104.88
102.75
77.69
77.26
76.84
67.55
56.88
55.40
53.79
49.12
32.68
18.96
6c
N
N
O
O
O
O
O
220 210 200 190 180 170 160 150 140 130 120 110 100 90 80 70 60 50 40 30 20 10 0
f1 (ppm)

6d

KO-418-1H-cdcl3

KO-418 w CDCl3 + TMS

6d

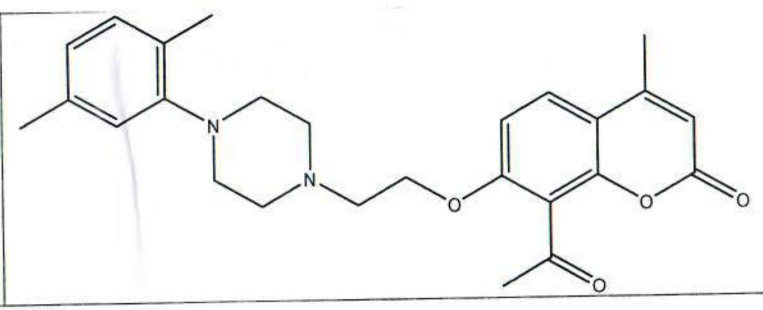

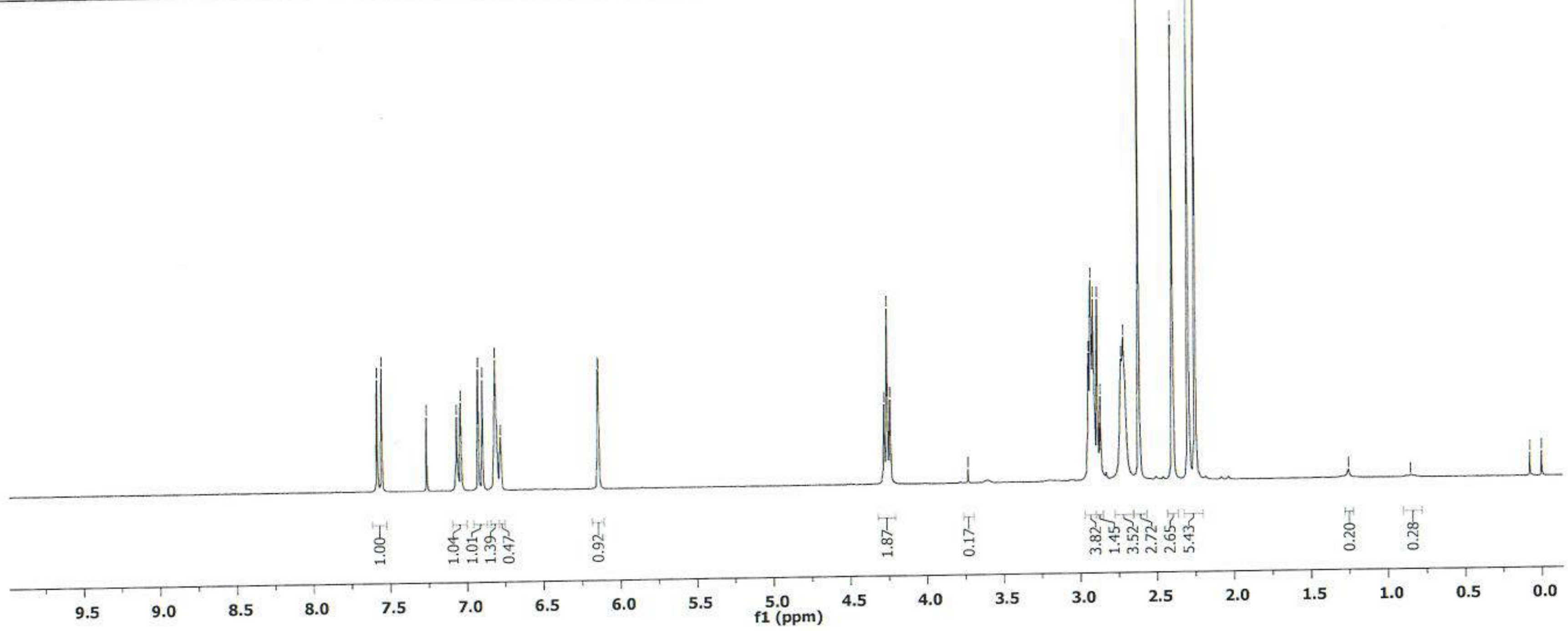

6d

KO-418-13C

KO 418 13C in CDCl3

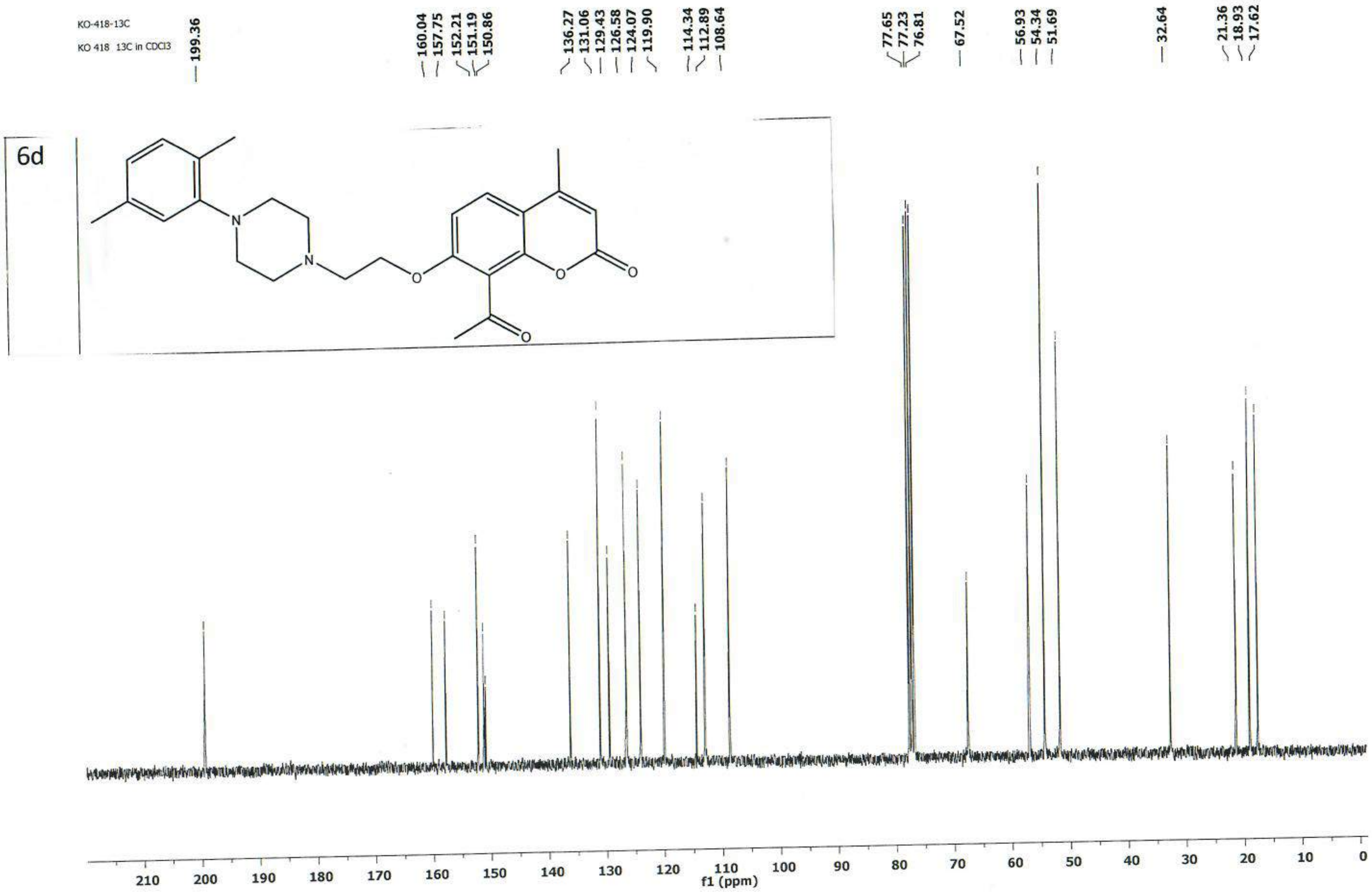

6e

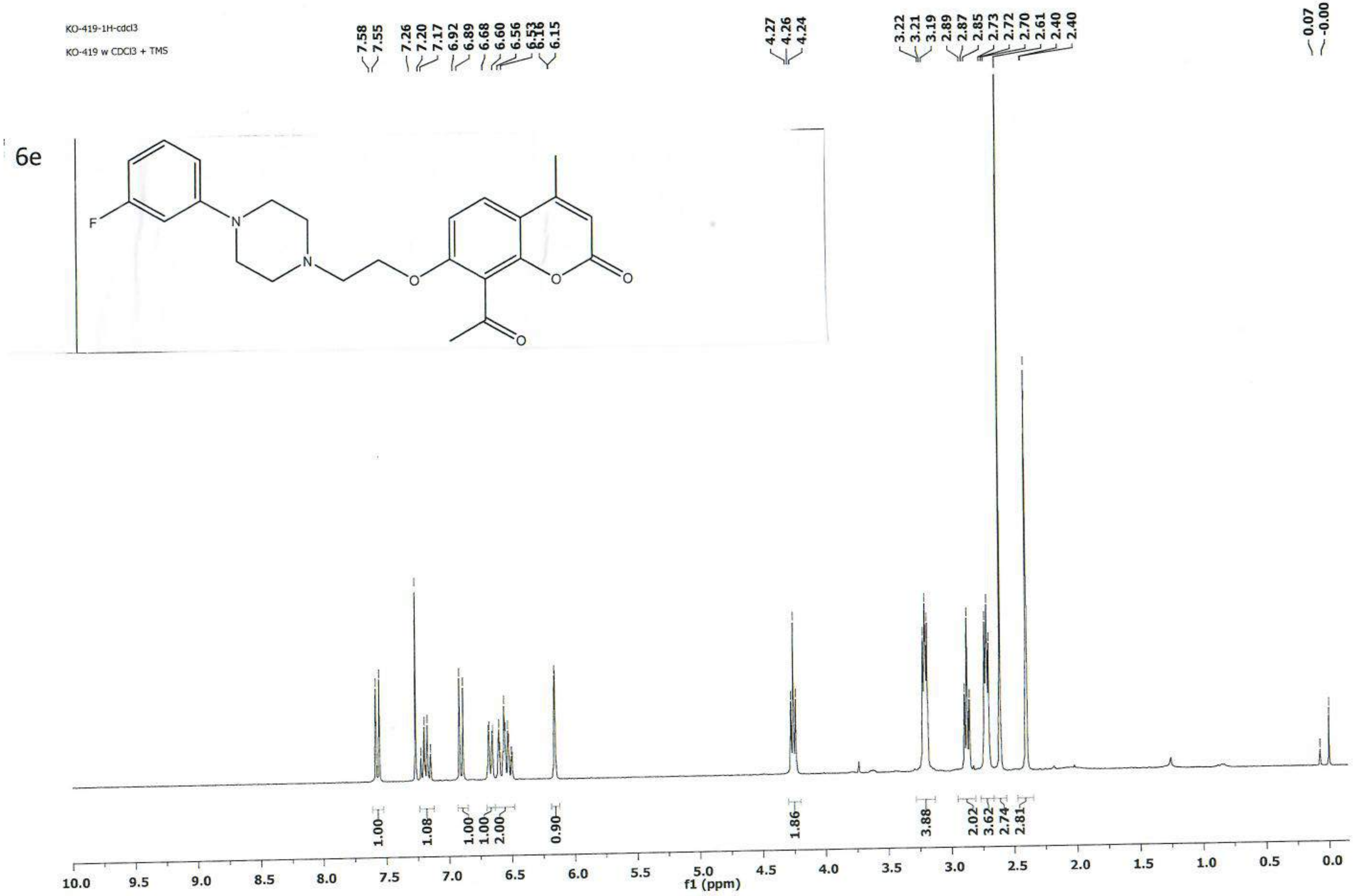
KO-419-1H-cdcl3
KO-419 w CDCl3 + TMS
6e
F
N
N
O
O
O
O
7.58
7.55
7.26
7.20
7.17
6.92
6.89
6.68
6.60
6.56
6.53
6.16
6.15
4.27
4.26
4.24
3.22
3.21
3.19
2.89
2.87
2.85
2.73
2.72
2.70
2.61
2.40
2.40
0.07
-0.00
1.00
1.08
1.00
1.00
2.00
0.90
1.86
3.88
2.02
3.62
2.74
2.81
10.0 9.5 9.0 8.5 8.0 7.5 7.0 6.5 6.0 5.5 5.0 4.5 4.0 3.5 3.0 2.5 2.0 1.5 1.0 0.5 0.0
f1 (ppm)

6e

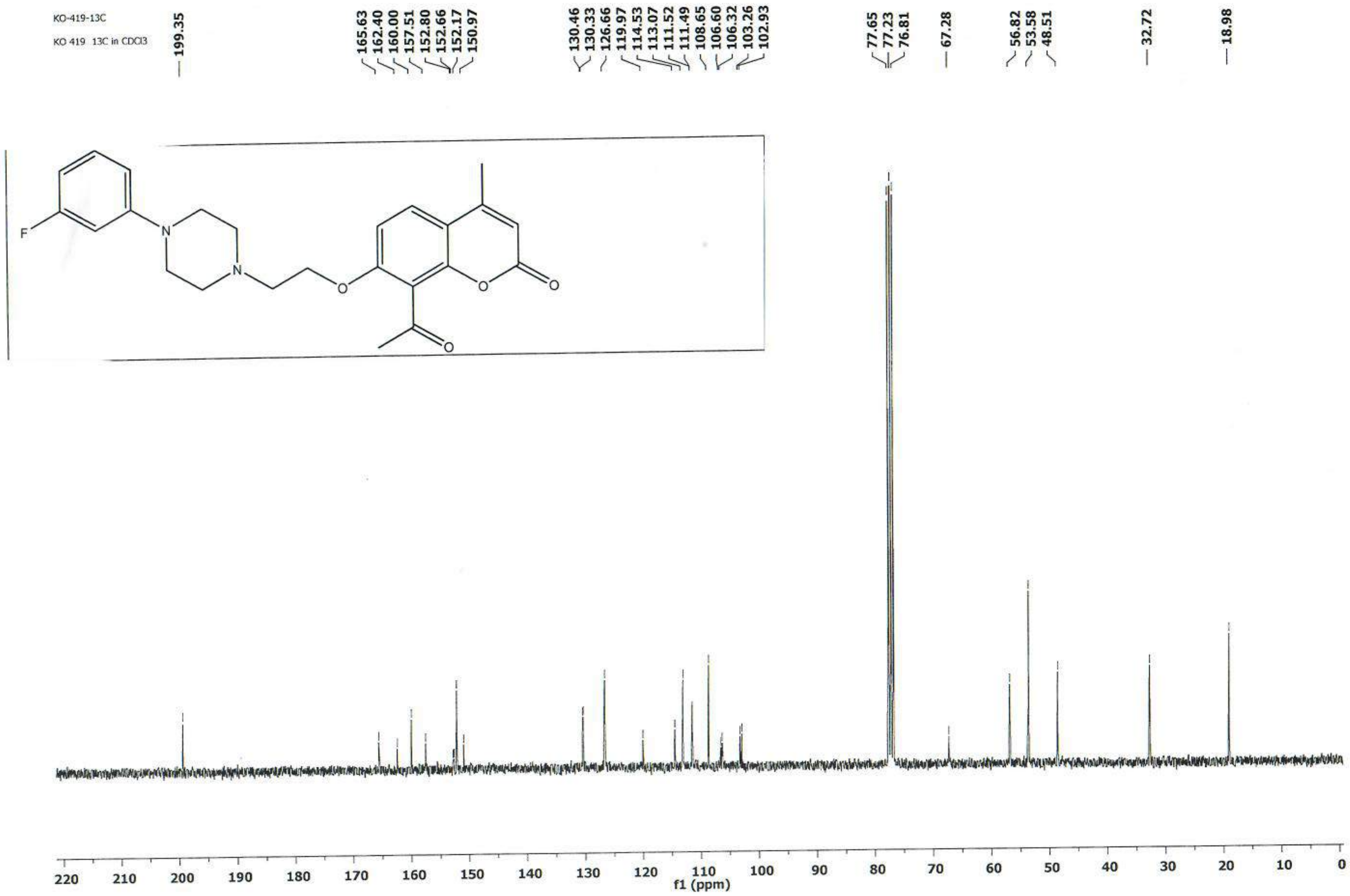
KO-419-13C
KO 419 13C in CDCl3
199.35
165.63
162.40
160.00
157.51
152.80
152.66
152.17
150.97
130.46
130.33
126.66
119.97
114.53
113.07
111.52
111.49
108.65
106.60
106.32
103.26
102.93
77.65
77.23
76.81
67.28
56.82
53.58
48.51
32.72
18.98
6e
F
N
N
O
O
O
O
220 210 200 190 180 170 160 150 140 130 120 110 100 90 80 70 60 50 40 30 20 10 0
f1 (ppm)

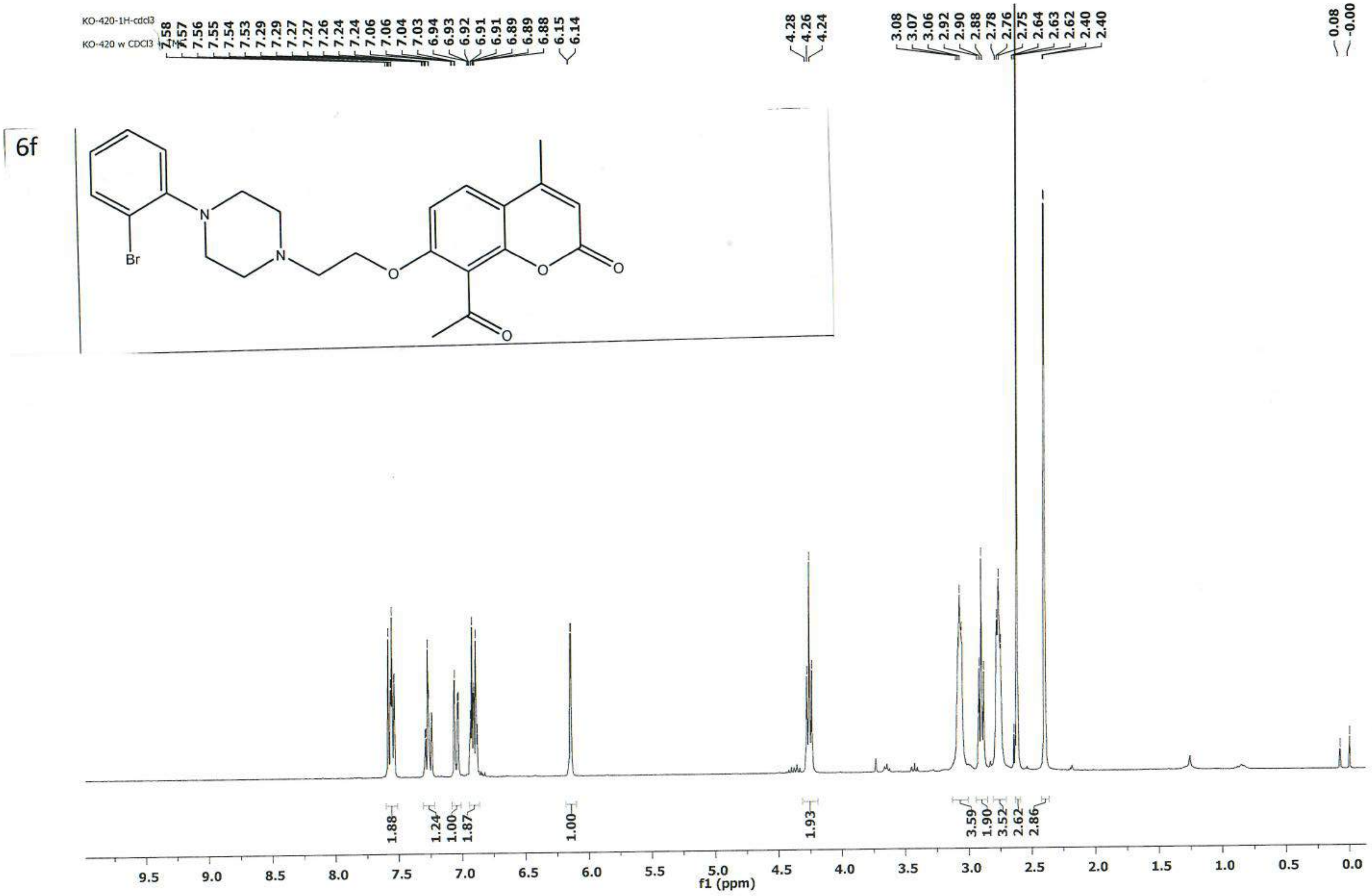
KO-420-1H-cdcl3
KO-420 w CDCl3 + TMS
7.58 7.57 7.56 7.55 7.54 7.53 7.29 7.29 7.27 7.27 7.26 7.24 7.24 7.06 7.06 7.04 7.03 6.94 6.93 6.92 6.91 6.91 6.89 6.89 6.88 6.15 6.14
4.28 4.26 4.24
3.08 3.07 3.06 2.92 2.90 2.88 2.78 2.76 2.75 2.64 2.63 2.62 2.40 2.40
0.08 -0.00
6f
N
N
Br
O
O
O
O
1.88 1.24 1.00 1.87 1.00 1.93 3.59 1.90 3.52 2.62 2.86
9.5 9.0 8.5 8.0 7.5 7.0 6.5 6.0 5.5 5.0 4.5 4.0 3.5 3.0 2.5 2.0 1.5 1.0 0.5 0.0
f1 (ppm)

6f

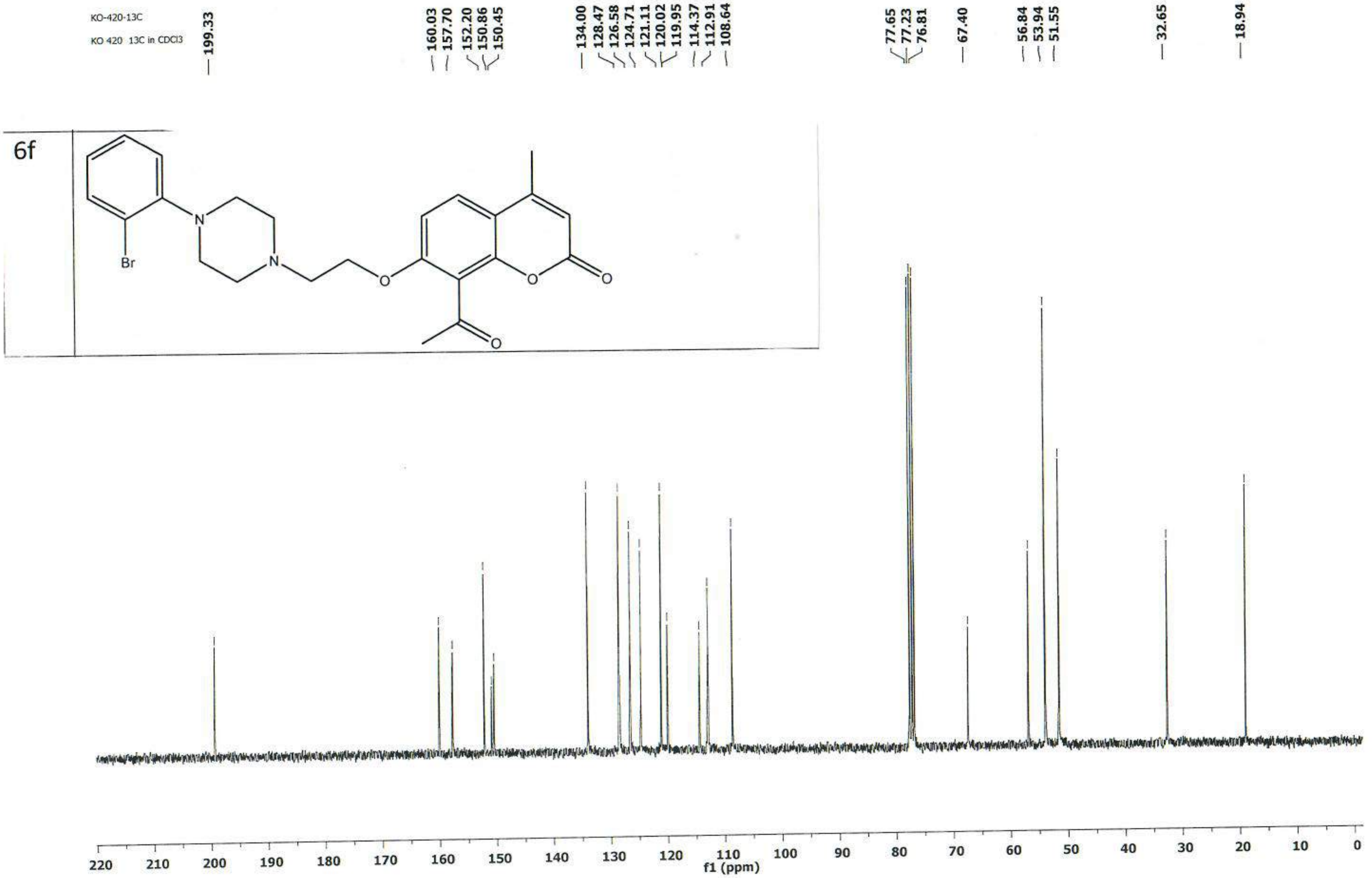
KO-420-13C
KO 420 13C in CDCl3
199.33
160.03
157.70
152.20
150.86
150.45
134.00
128.47
126.58
124.71
121.11
120.02
119.95
114.37
112.91
108.64
77.65
77.23
76.81
67.40
56.84
53.94
51.55
32.65
18.94
6f
Br
N
N
O
O
O
O
220 210 200 190 180 170 160 150 140 130 120 110 100 90 80 70 60 50 40 30 20 10 0
f1 (ppm)

6g

KO-417-1H-cdcl3

KO-417 w CDCl3 + TMS

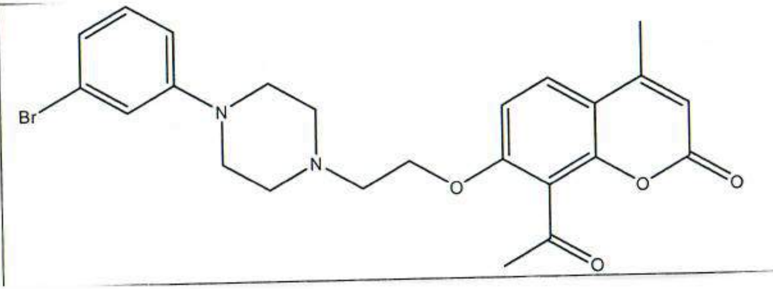

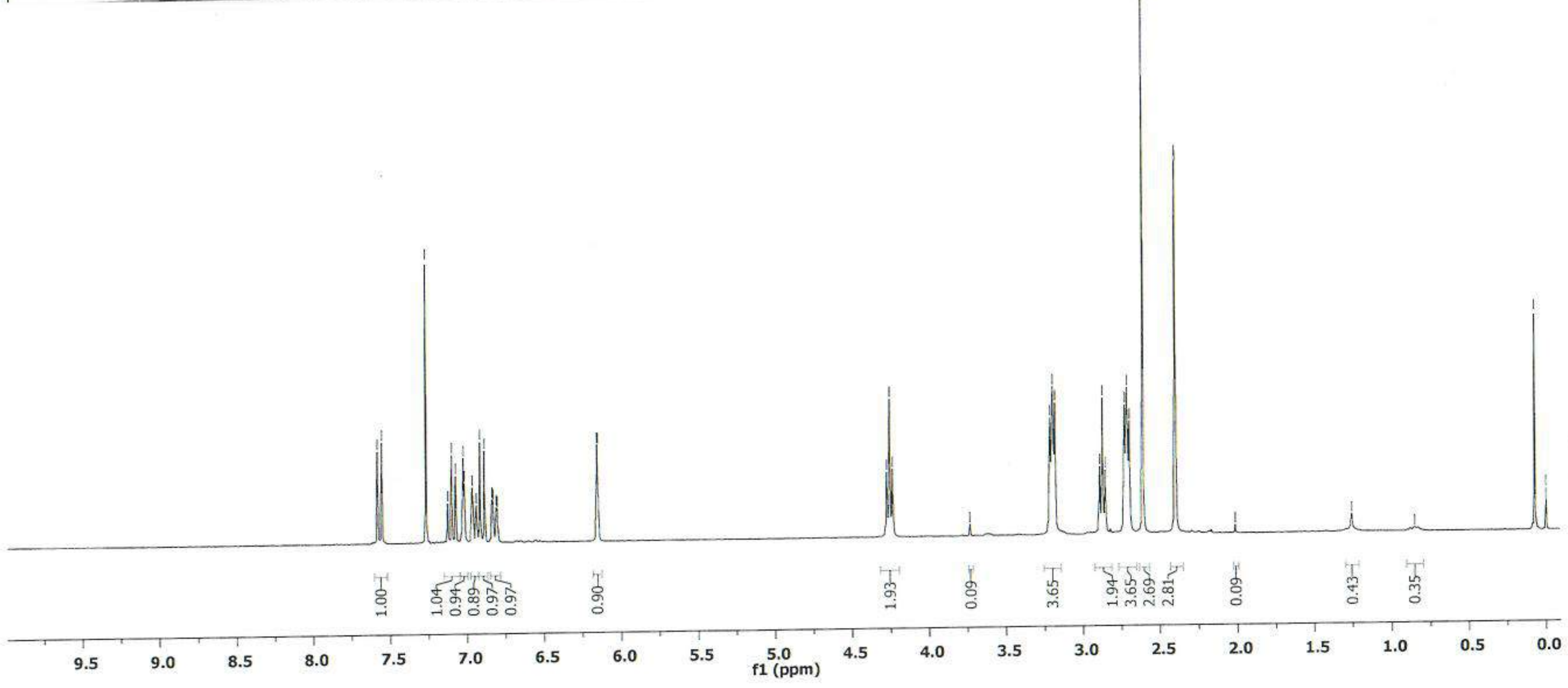

6g

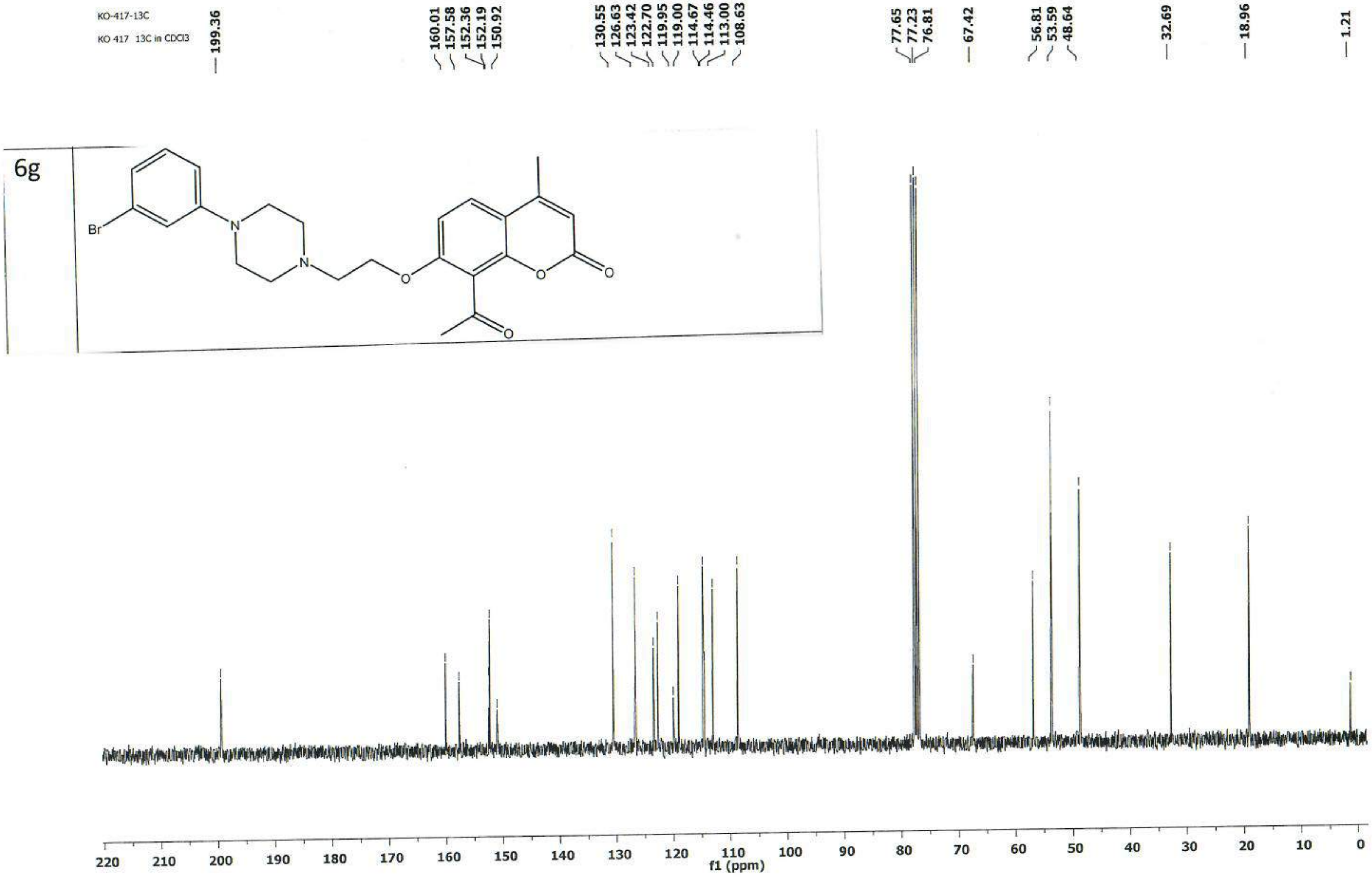
KO-417-13C
KO 417 13C in CDCl3
199.36
160.01
157.58
152.36
152.19
150.92
130.55
126.63
123.42
122.70
119.95
119.00
114.67
114.46
113.00
108.63
77.65
77.23
76.81
67.42
56.81
53.59
48.64
32.69
18.96
1.21
6g
Br
N
N
O
O
O
O
220 210 200 190 180 170 160 150 140 130 120 110 100 90 80 70 60 50 40 30 20 10 0
f1 (ppm)

64

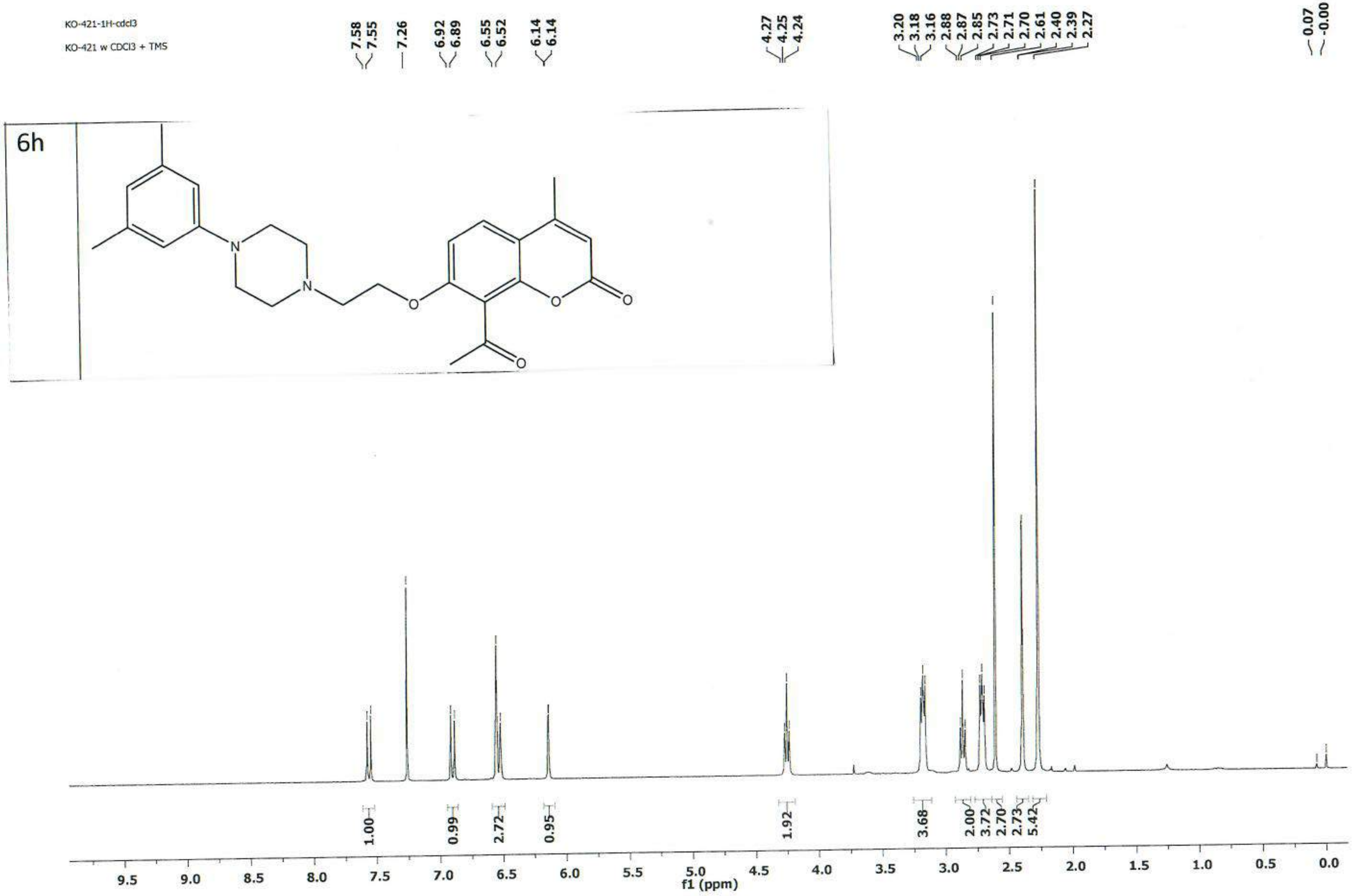

KO-421-1H-cdcl3
KO-421 w CDCl3 + TMS
7.58
7.55
7.26
6.92
6.89
6.55
6.52
6.14
6.14
4.27
4.25
4.24
3.20
3.18
3.16
2.88
2.87
2.85
2.73
2.71
2.70
2.61
2.40
2.39
2.27
0.07
-0.00
6h
N
N
O
O
O
O
1.00
0.99
2.72
0.95
1.92
3.68
2.00
3.72
2.70
2.73
5.42
9.5
9.0
8.5
8.0
7.5
7.0
6.5
6.0
5.5
5.0
4.5
4.0
3.5
3.0
2.5
2.0
1.5
1.0
0.5
0.0
f1 (ppm)

6h

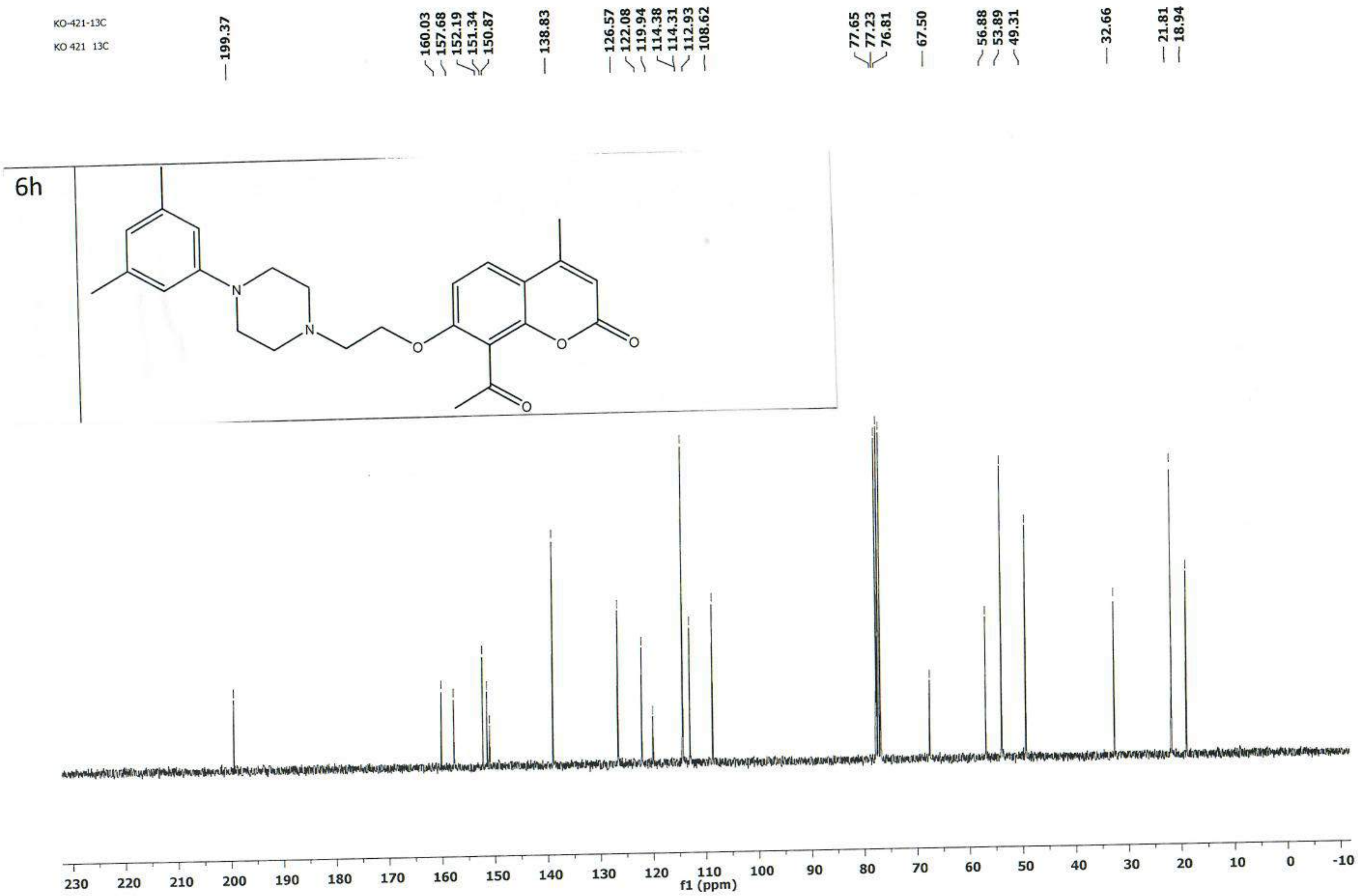
KO-421-13C
KO 421 13C
199.37
160.03
157.68
152.19
151.34
150.87
138.83
126.57
122.08
119.94
114.38
114.31
112.93
108.62
77.65
77.23
76.81
67.50
56.88
53.89
49.31
32.66
21.81
18.94
6h
N
N
O
O
O
O
230 220 210 200 190 180 170 160 150 140 130 120 110 100 90 80 70 60 50 40 30 20 10 0 -10
f1 (ppm)

6i

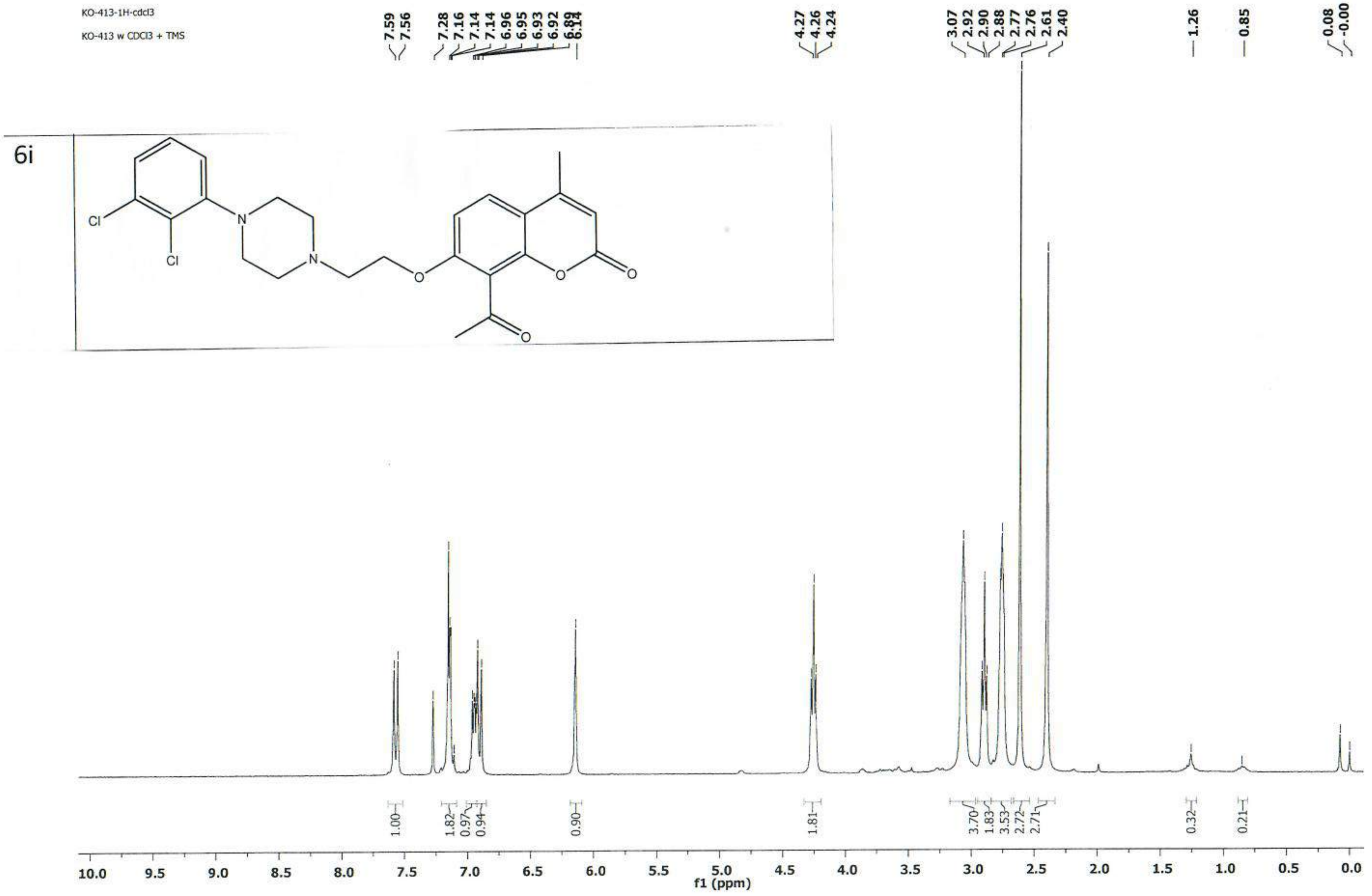

KO-413-1H-cdcl3
KO-413 w CDCl3 + TMS
7.59
7.56
7.28
7.16
7.14
7.14
6.96
6.95
6.93
6.92
6.89
6.14
4.27
4.26
4.24
3.07
2.92
2.90
2.88
2.77
2.76
2.61
2.40
1.26
0.85
0.08
-0.00
6i
Cl
Cl
N
N
O
O
O
O
1.00
1.82
0.97
0.94
0.90
1.81
3.70
1.83
3.53
2.72
2.71
0.32
0.21
10.0
9.5
9.0
8.5
8.0
7.5
7.0
6.5
6.0
5.5
5.0
4.5
4.0
3.5
3.0
2.5
2.0
1.5
1.0
0.5
0.0
f1 (ppm)

6i

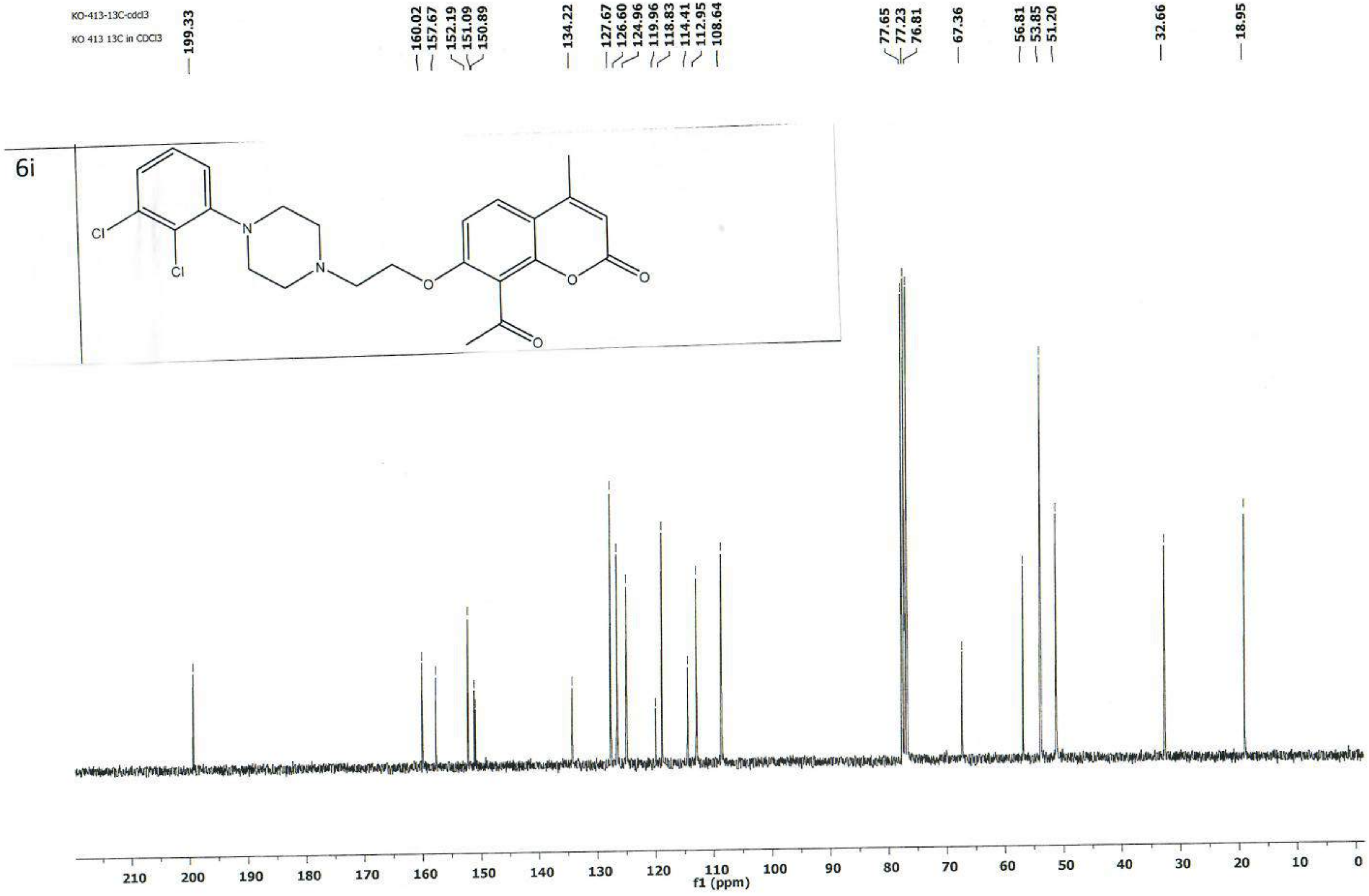
KO-413-13C-cdcl3
KO 413 13C in CDCl3
199.33
160.02
157.67
152.19
151.09
150.89
134.22
127.67
126.60
124.96
119.96
118.83
114.41
112.95
108.64
77.65
77.23
76.81
67.36
56.81
53.85
51.20
32.66
18.95
6i
Cl
Cl
N
N
O
O
O
O
210
200
190
180
170
160
150
140
130
120
110
100
90
80
70
60
50
40
30
20
10
0
f1 (ppm)

6j

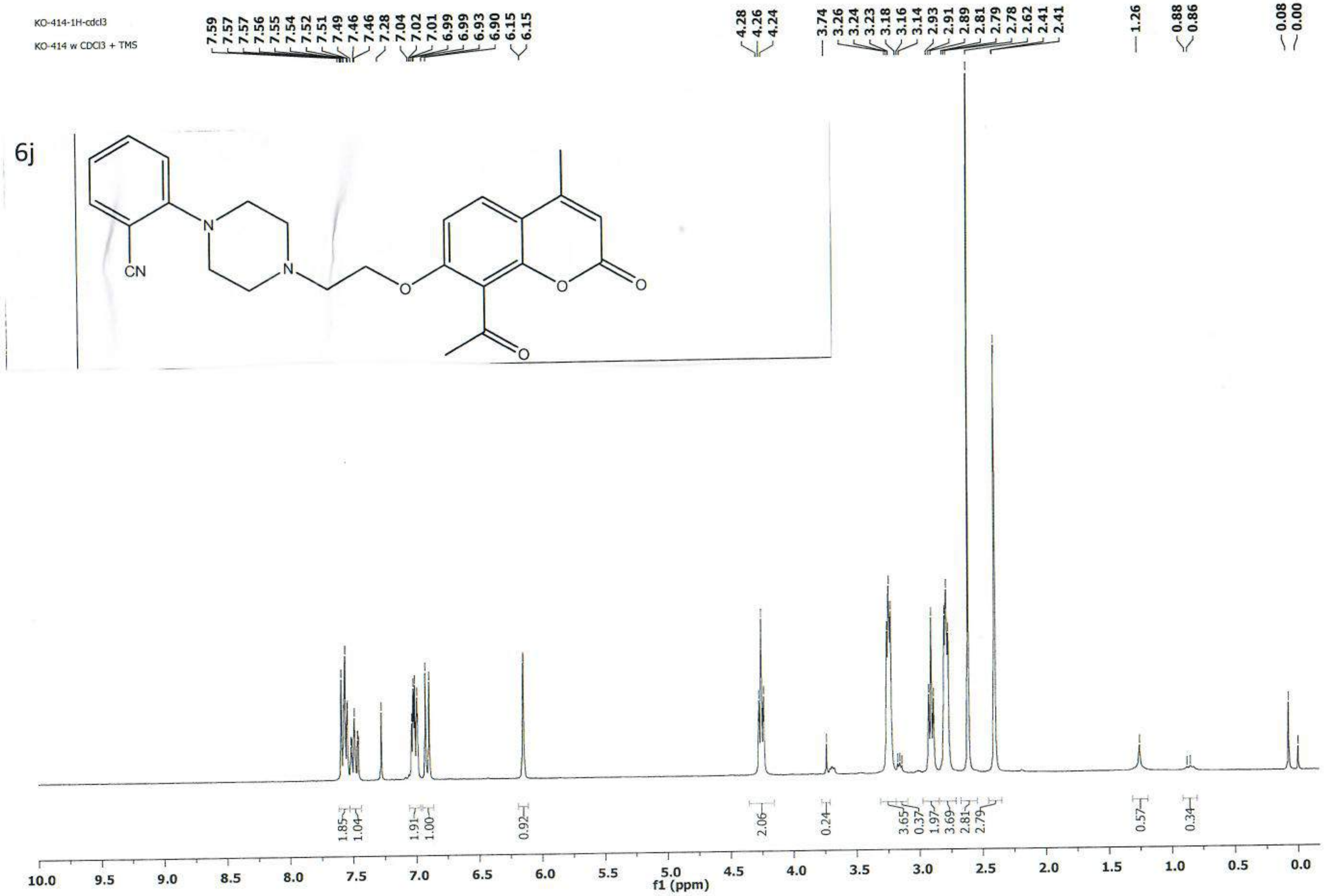
KO-414-1H-cdcl3
KO-414 w CDCl3 + TMS
7.59 7.57 7.57 7.56 7.55 7.54 7.52 7.51 7.49 7.46 7.46 7.28 7.04 7.02 7.01 6.99 6.99 6.93 6.90 6.15 6.15
4.28 4.26 4.24
3.74 3.26 3.24 3.23 3.18 3.16 3.14 2.93 2.91 2.89 2.81 2.79 2.78 2.62 2.41 2.41
1.26
0.88 0.86
0.08 0.00
6j
CN
N
N
O
O
O
O
1.85 1.04 1.91 1.00 0.92 2.06 0.24 3.65 0.37 1.97 3.69 2.81 2.79 0.57 0.34
10.0 9.5 9.0 8.5 8.0 7.5 7.0 6.5 6.0 5.5 5.0 4.5 4.0 3.5 3.0 2.5 2.0 1.5 1.0 0.5 0.0
f1 (ppm)

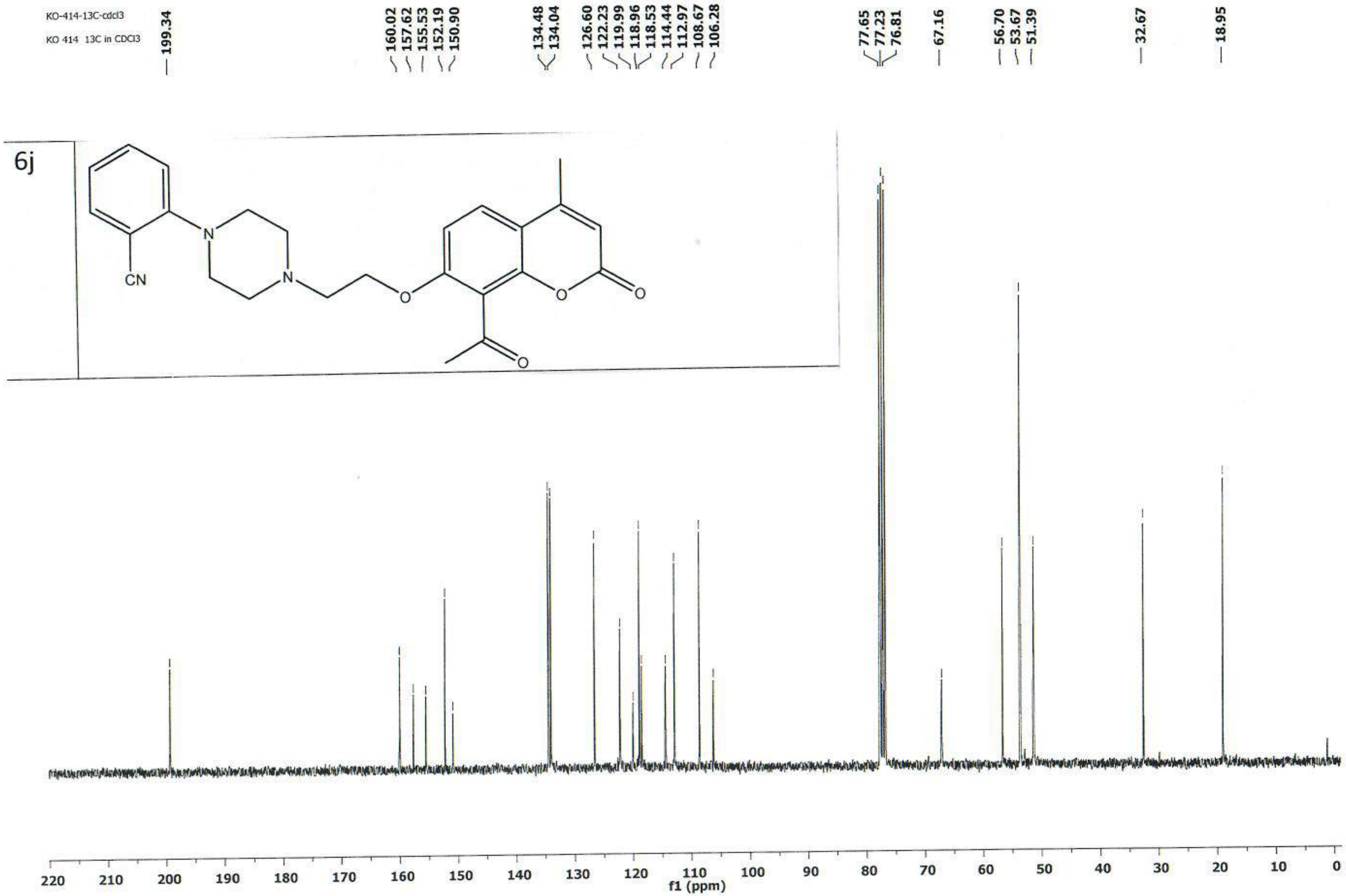
6j
CN
N
N
O
O
O
O
KO-414-13C-cdcl3
KO 414 13C in CDCl3
199.34
160.02
157.62
155.53
152.19
150.90
134.48
134.04
126.60
122.23
119.99
118.96
118.53
114.44
112.97
108.67
106.28
77.65
77.23
76.81
67.16
56.70
53.67
51.39
32.67
18.95
220 210 200 190 180 170 160 150 140 130 120 110 100 90 80 70 60 50 40 30 20 10 0
f1 (ppm)

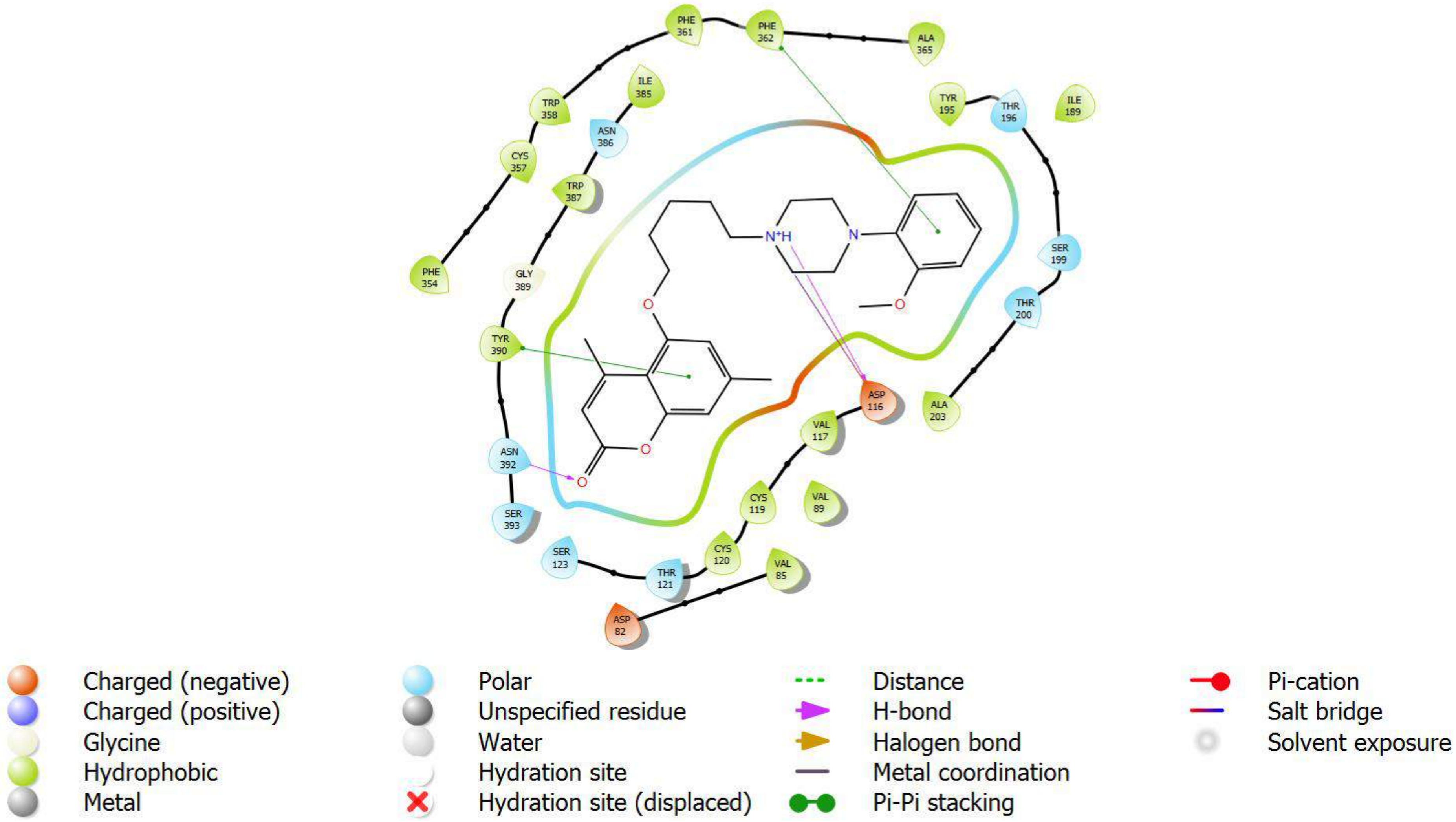

Figure S2. Ligand interaction diagram for compound **1a** docked to $5HT_{1A}$ receptor.

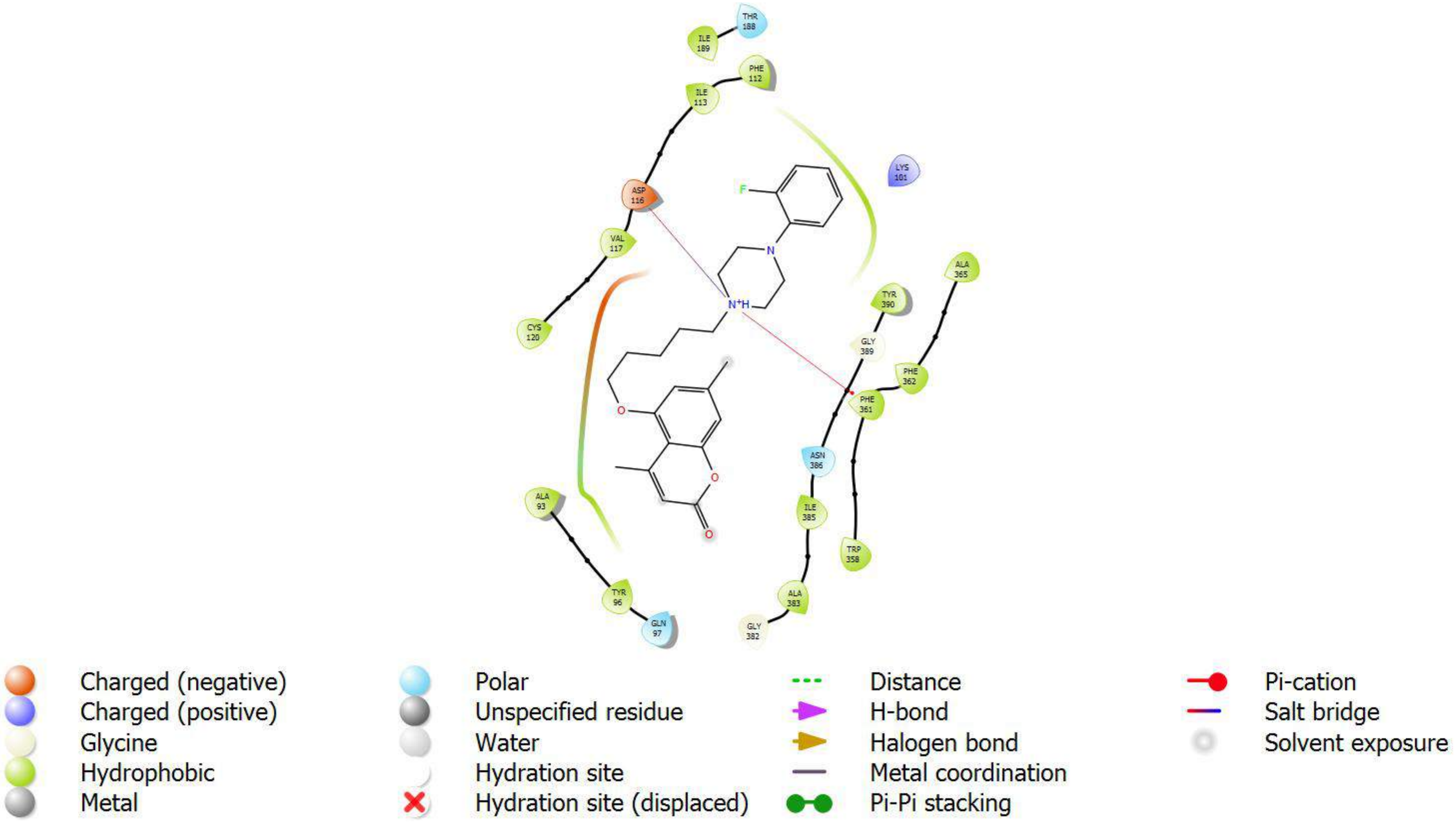

Figure S3. Ligand interaction diagram for compound **1b** docked to $5HT_{1A}$ receptor.

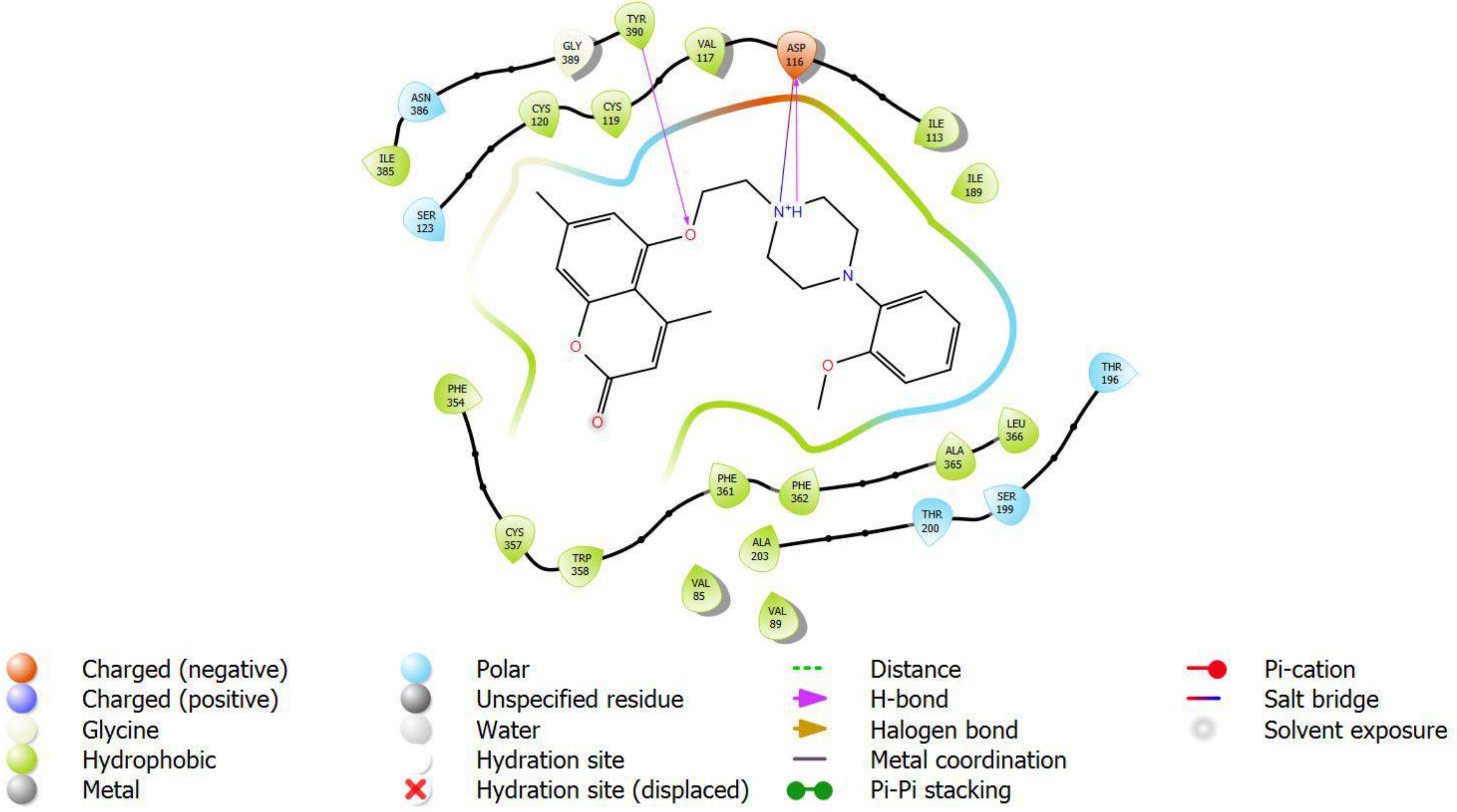

Figure S4. Ligand interaction diagram for compound **2a** docked to $5HT_{1A}$ receptor.

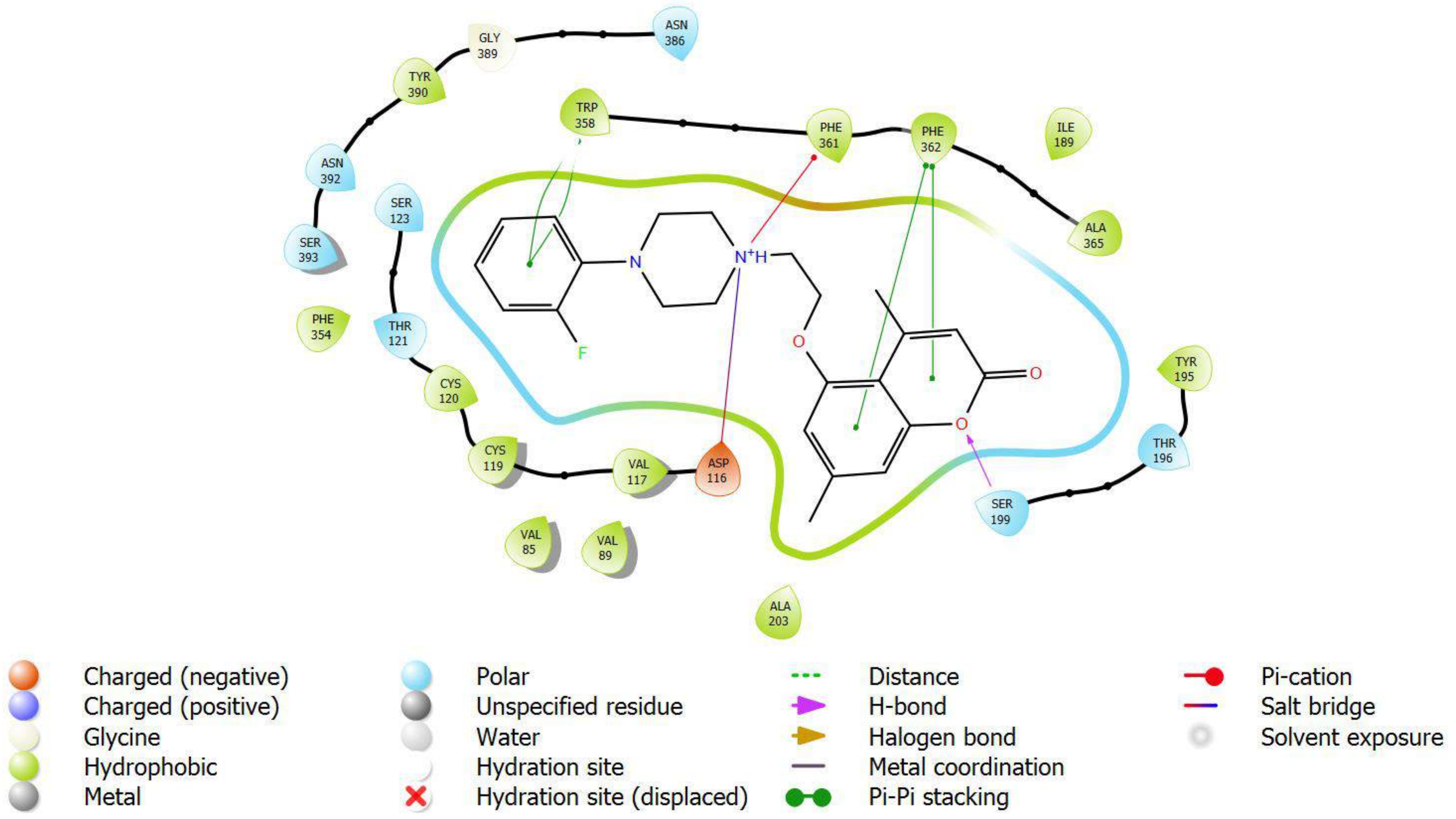

Figure S5. Ligand interaction diagram for compound **2b** docked to $5HT_{1A}$ receptor.

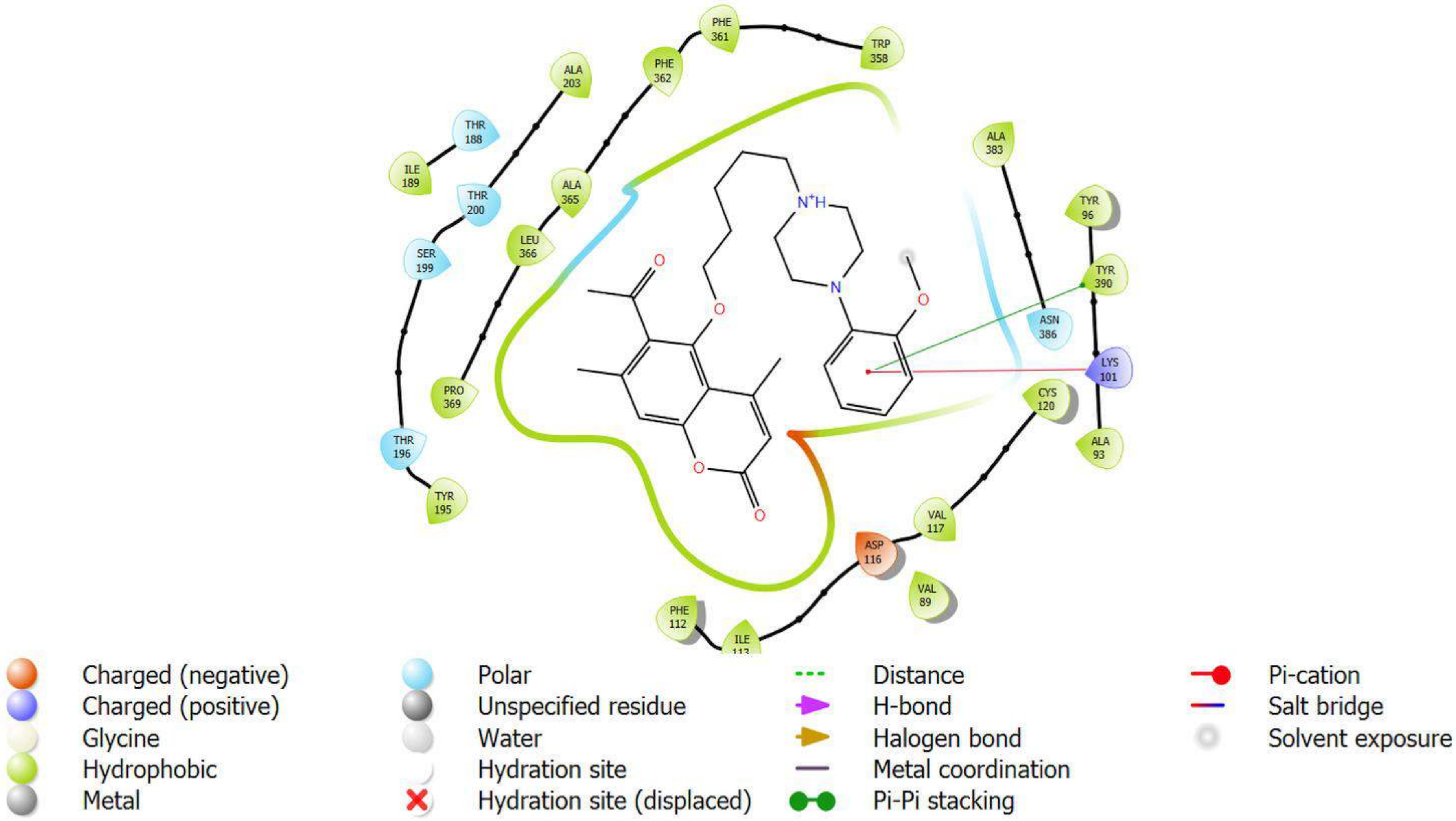

Figure S6. Ligand interaction diagram for compound **3a** docked to $5HT_{1A}$ receptor.

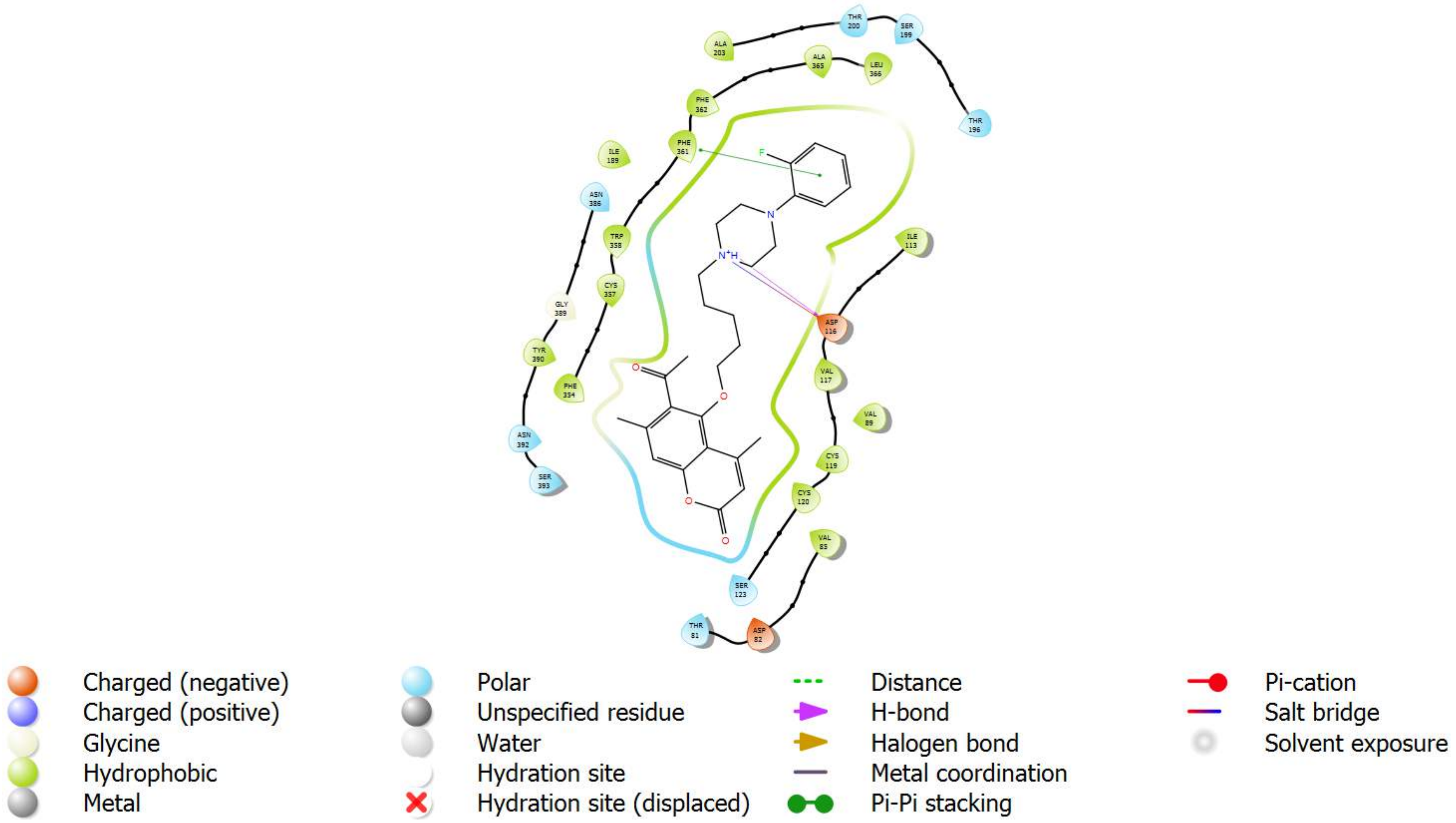

Figure S7. Ligand interaction diagram for compound **3b** docked to $5HT_{1A}$ receptor.

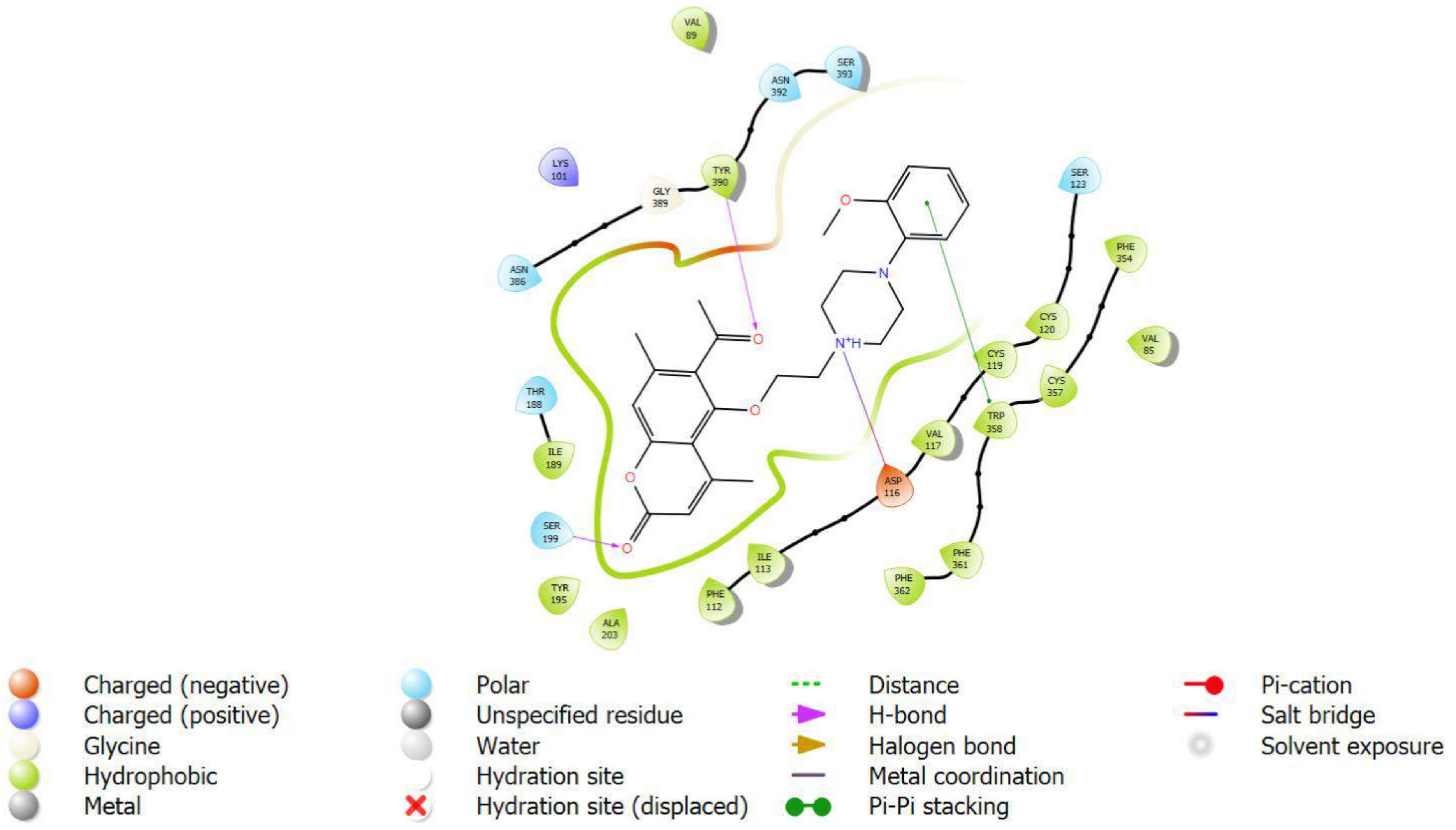

Figure S8. Ligand interaction diagram for compound **4a** docked to $5HT_{1A}$ receptor.

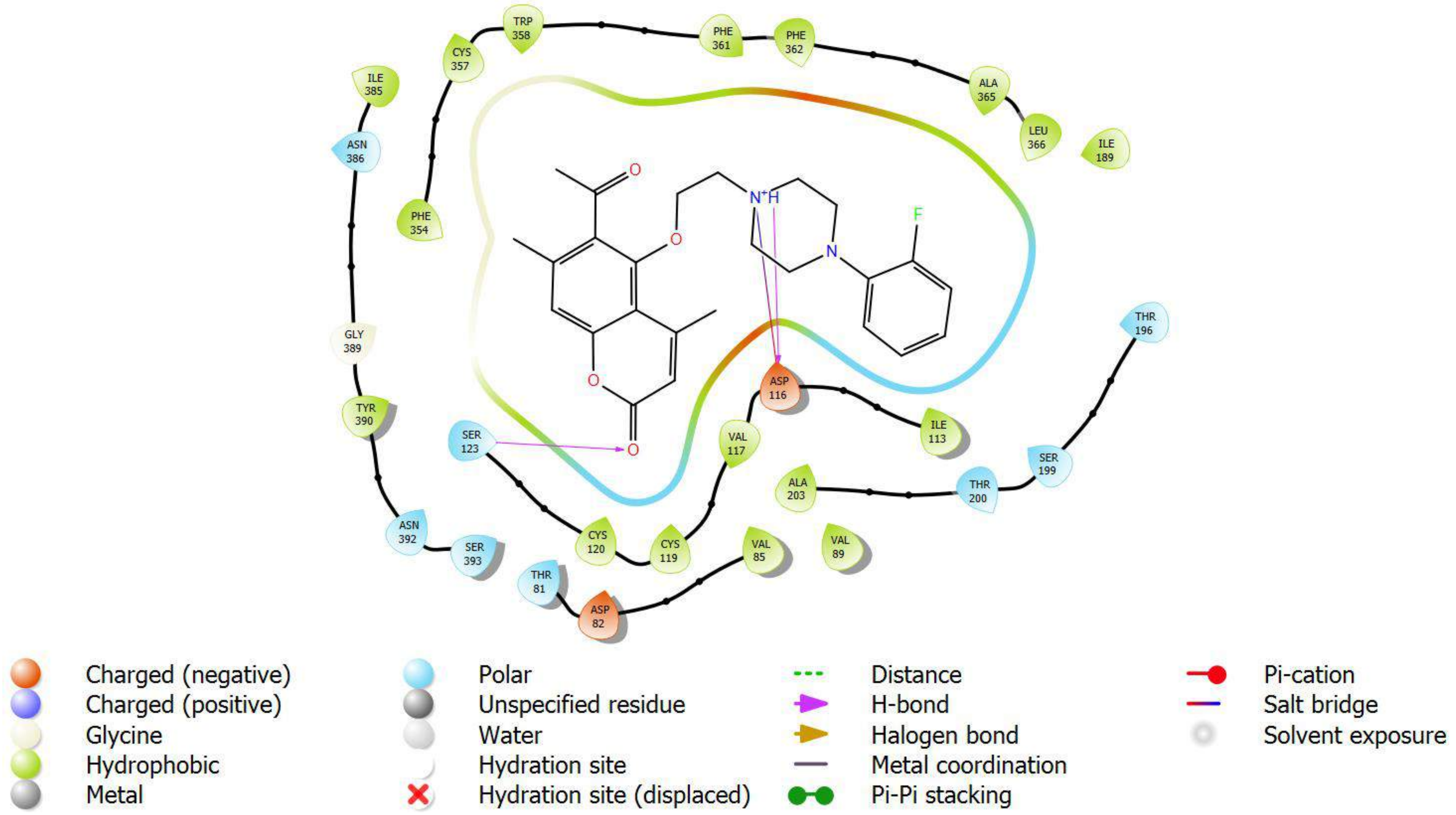

Figure S9. Ligand interaction diagram for compound **4b** docked to $5HT_{1A}$ receptor.

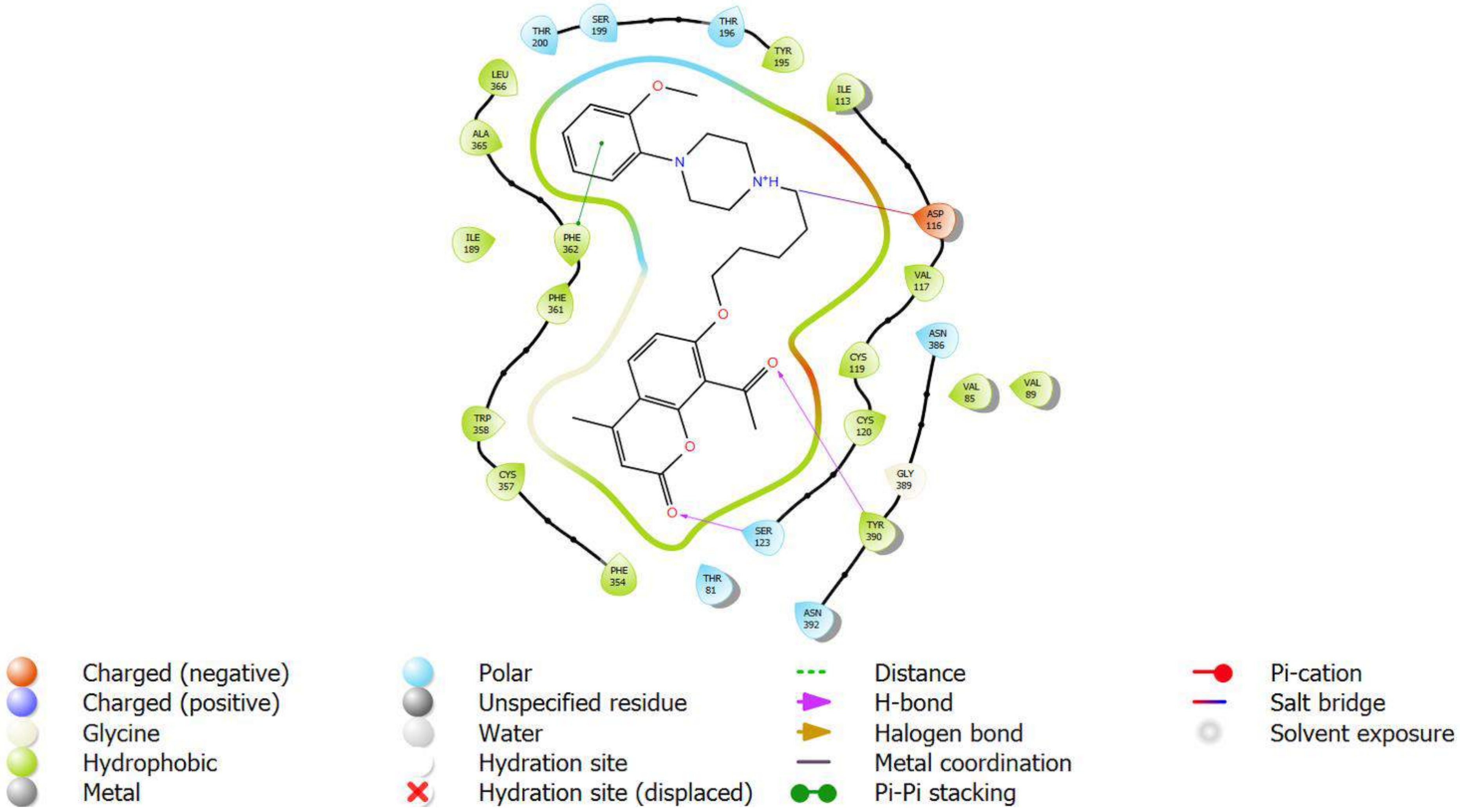

Figure S10. Ligand interaction diagram for compound **5a** docked to $5HT_{1A}$ receptor.

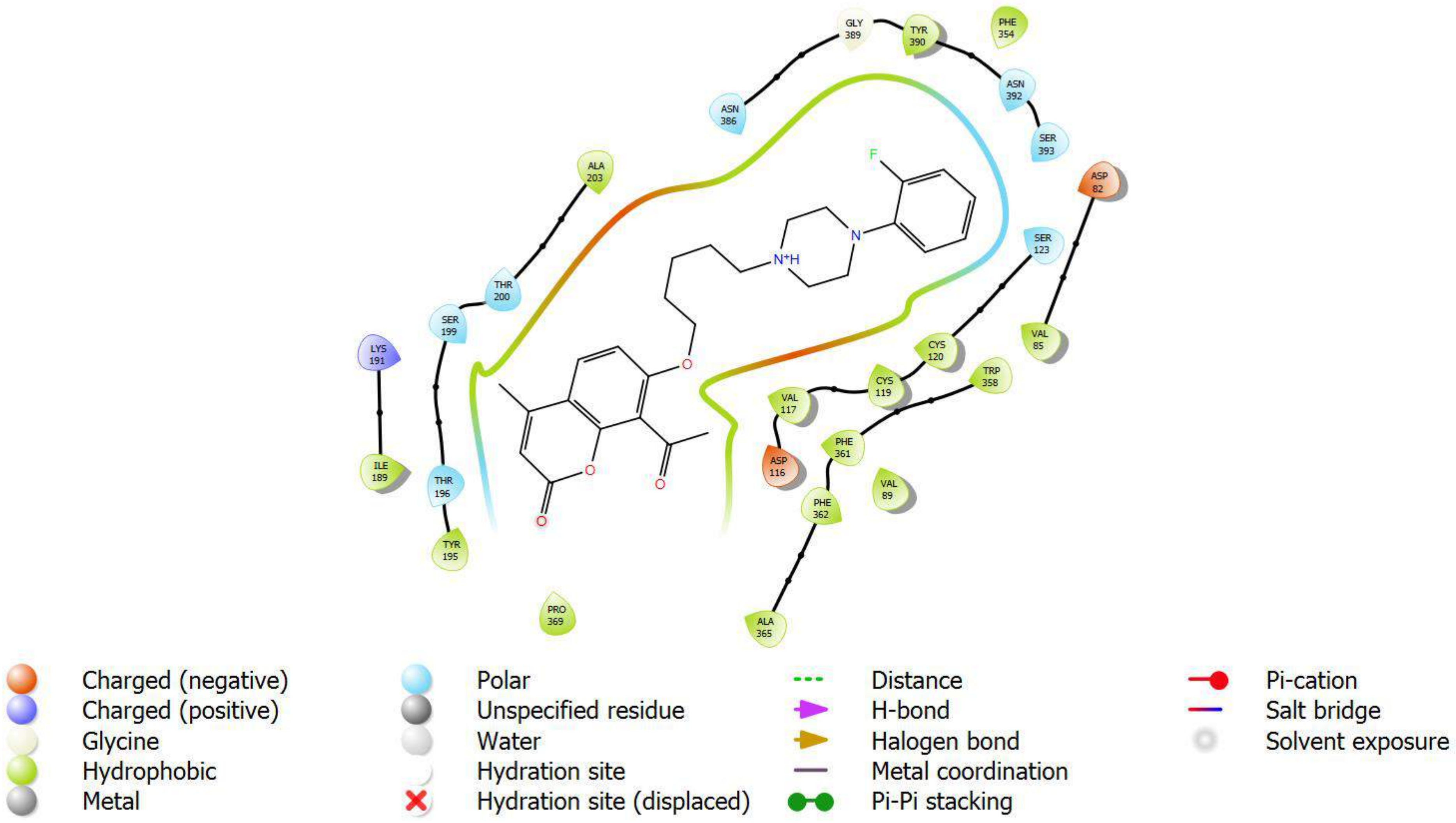

Figure S11. Ligand interaction diagram for compound **5b** docked to $5HT_{1A}$ receptor.

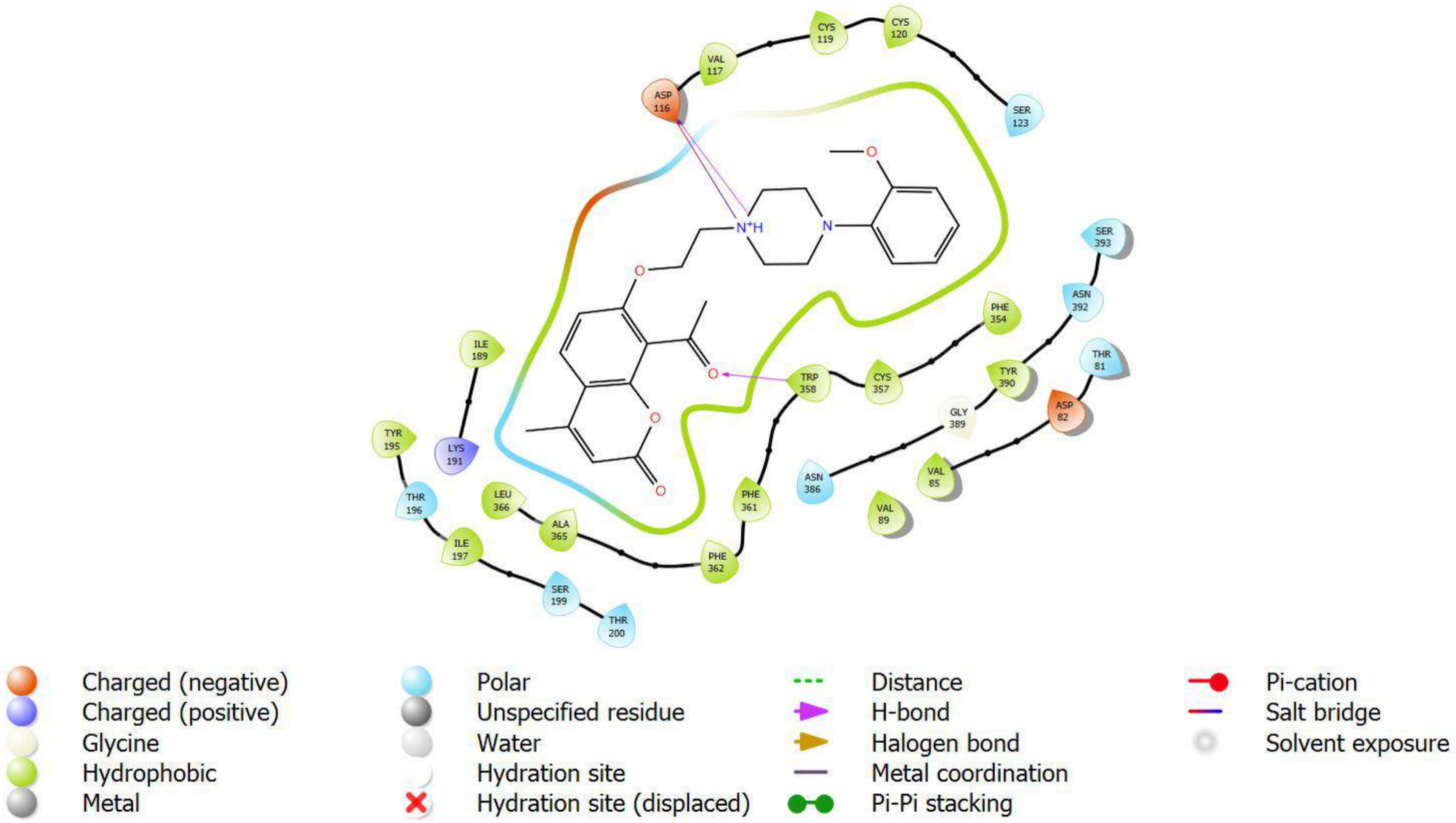

Figure S12. Ligand interaction diagram for compound **6a** docked to $5HT_{1A}$ receptor.

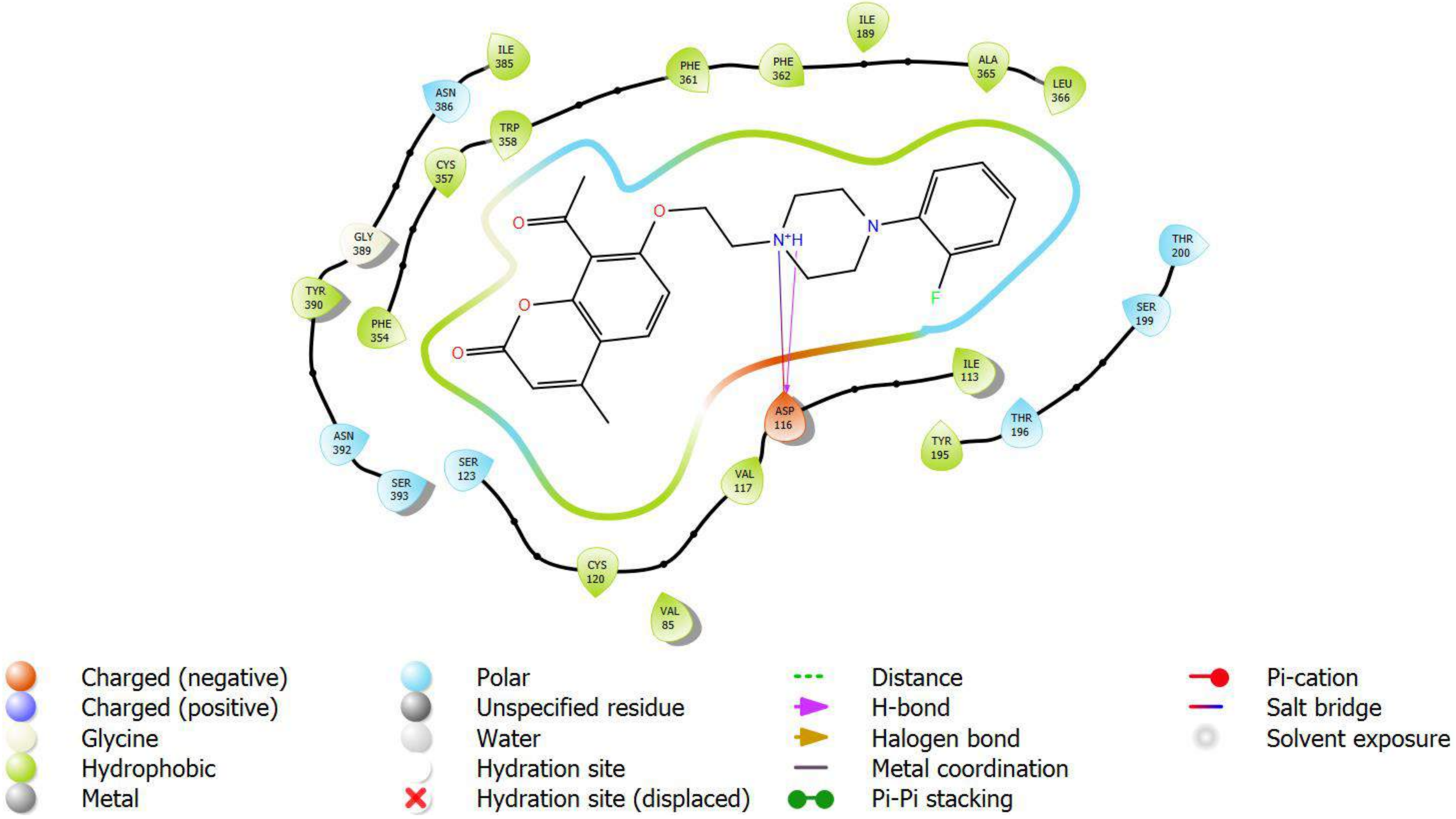

Figure S13. Ligand interaction diagram for compound **6b** docked to $5HT_{1A}$ receptor.

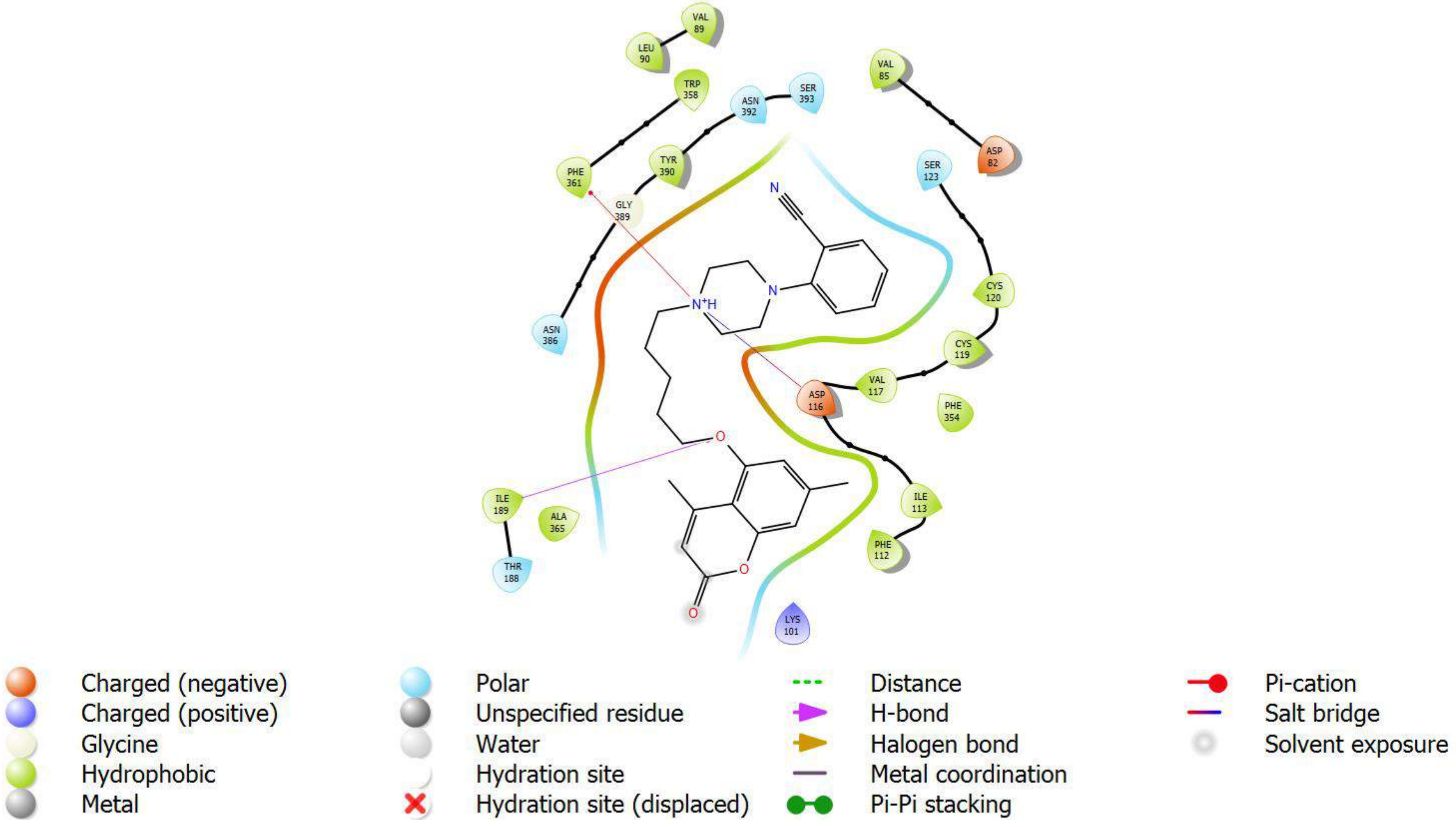

Figure S14. Ligand interaction diagram for compound **1j** docked to $5HT_{1A}$ receptor.

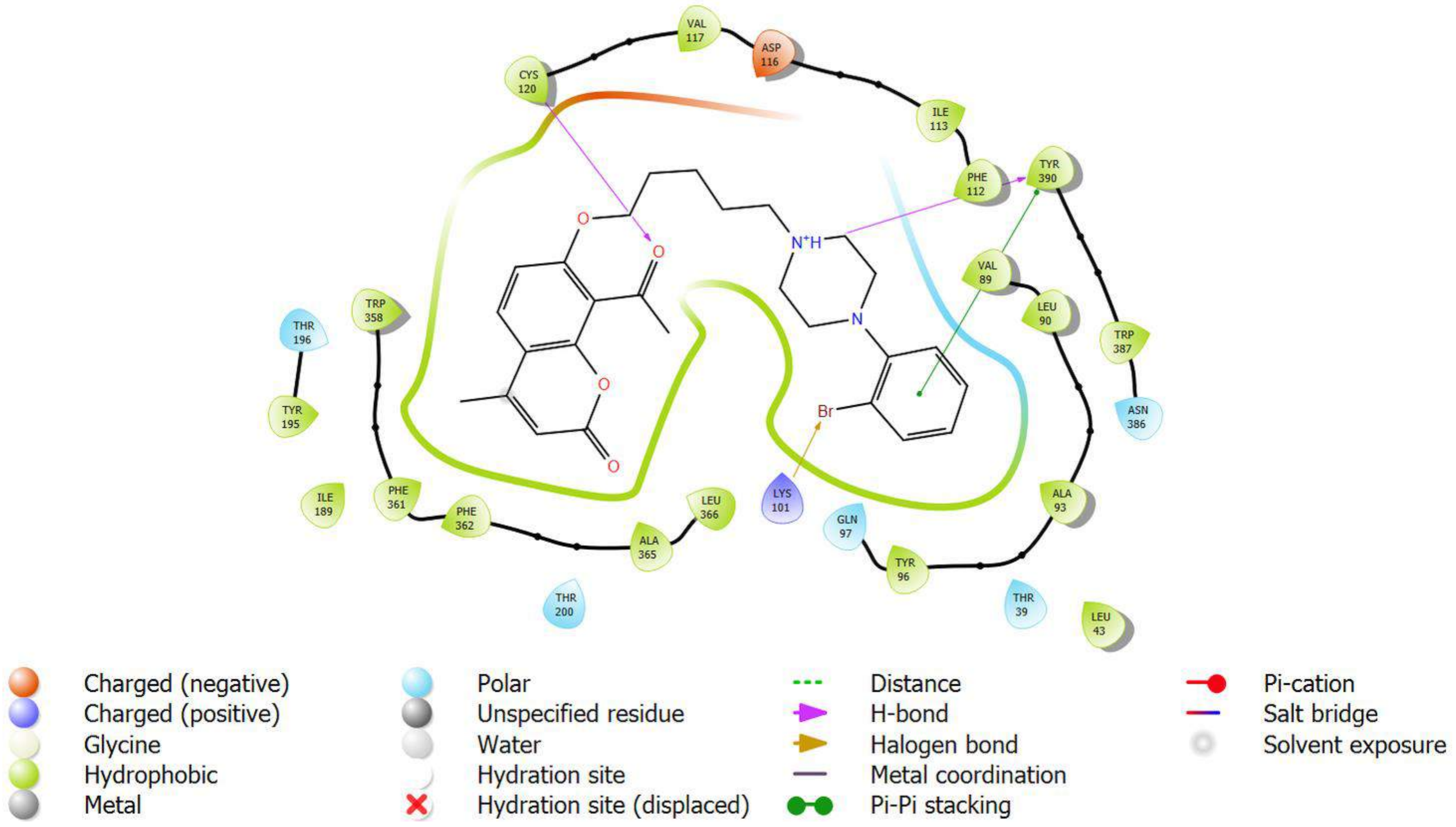

Figure S15. Ligand interaction diagram for compound **5f** docked to $5HT_{1A}$ receptor.

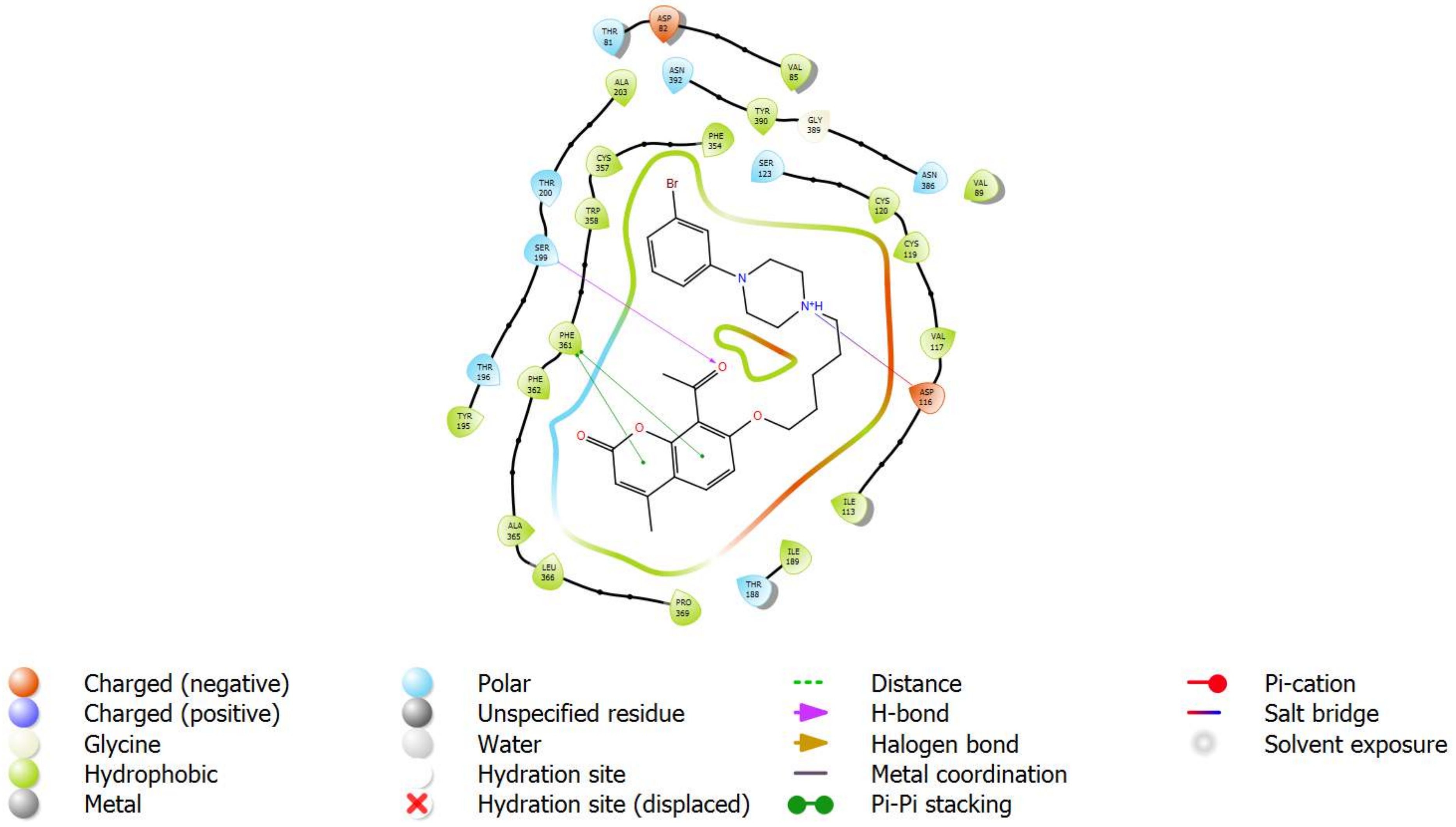

Figure S16. Ligand interaction diagram for compound **5g** docked to $5HT_{1A}$ receptor.

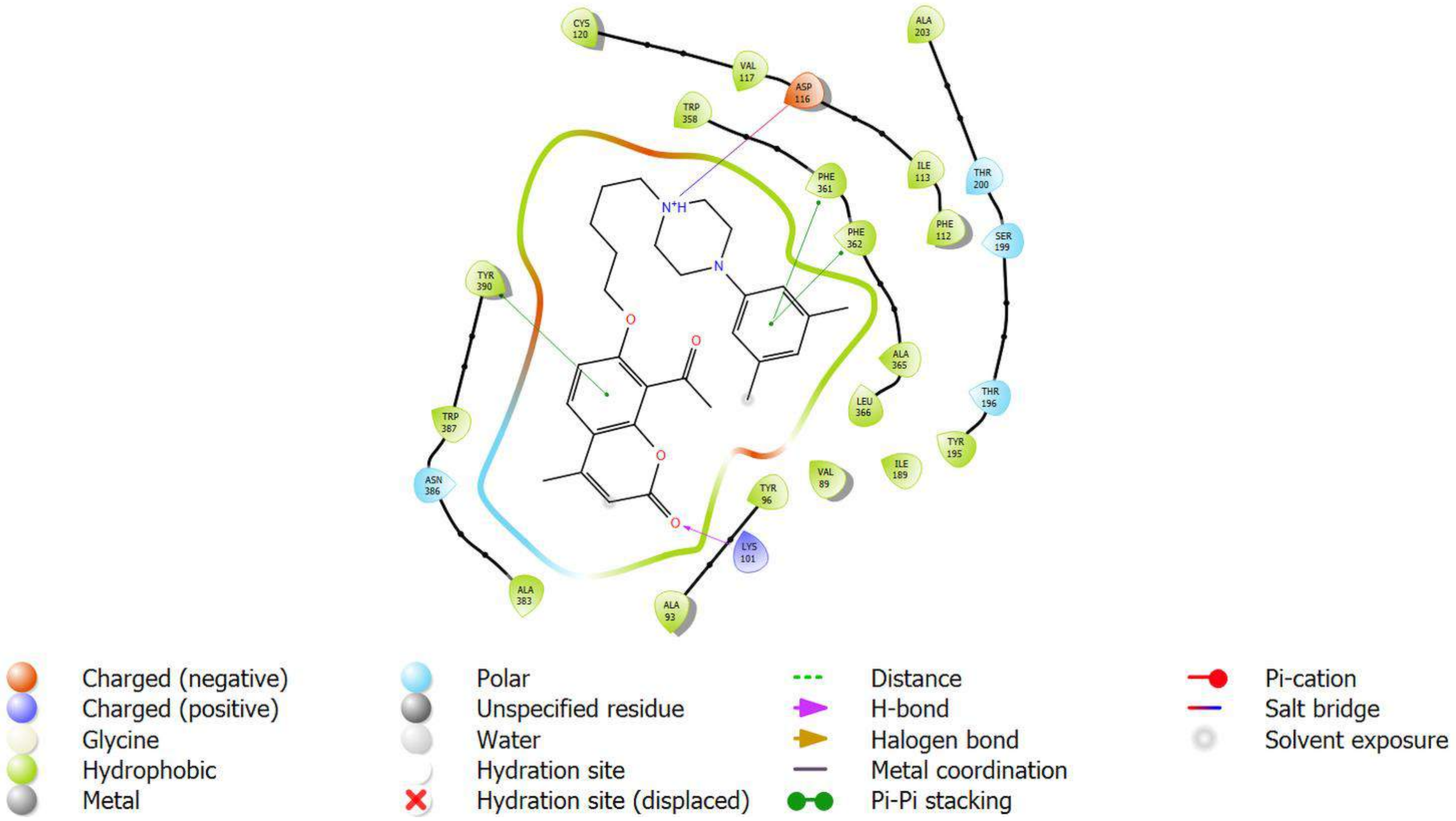

Figure S17. Ligand interaction diagram for compound **5h** docked to $5HT_{1A}$ receptor.

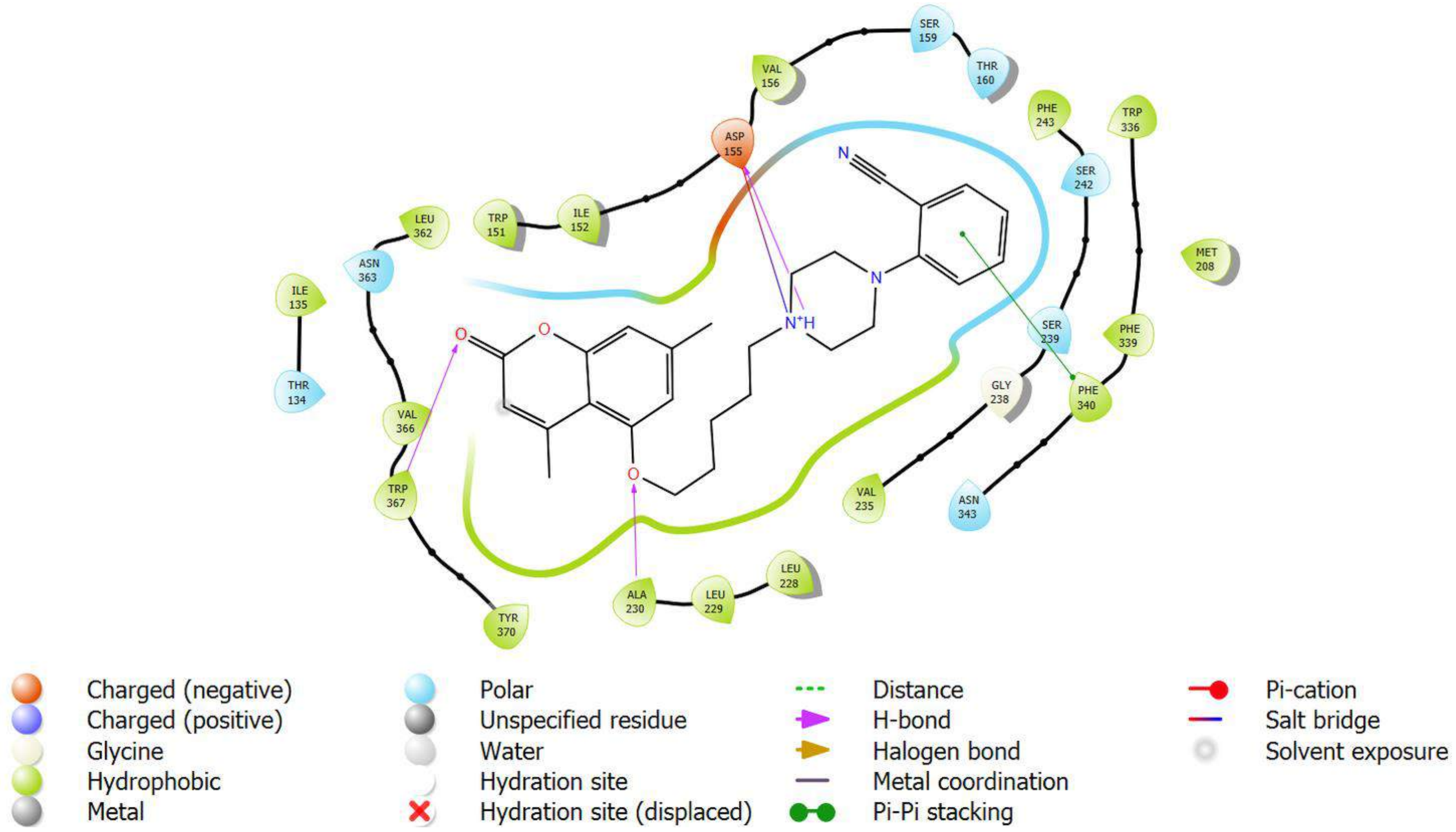

Figure S18. Ligand interaction diagram for compound **1j** docked to $5HT_{2A}$ receptor.

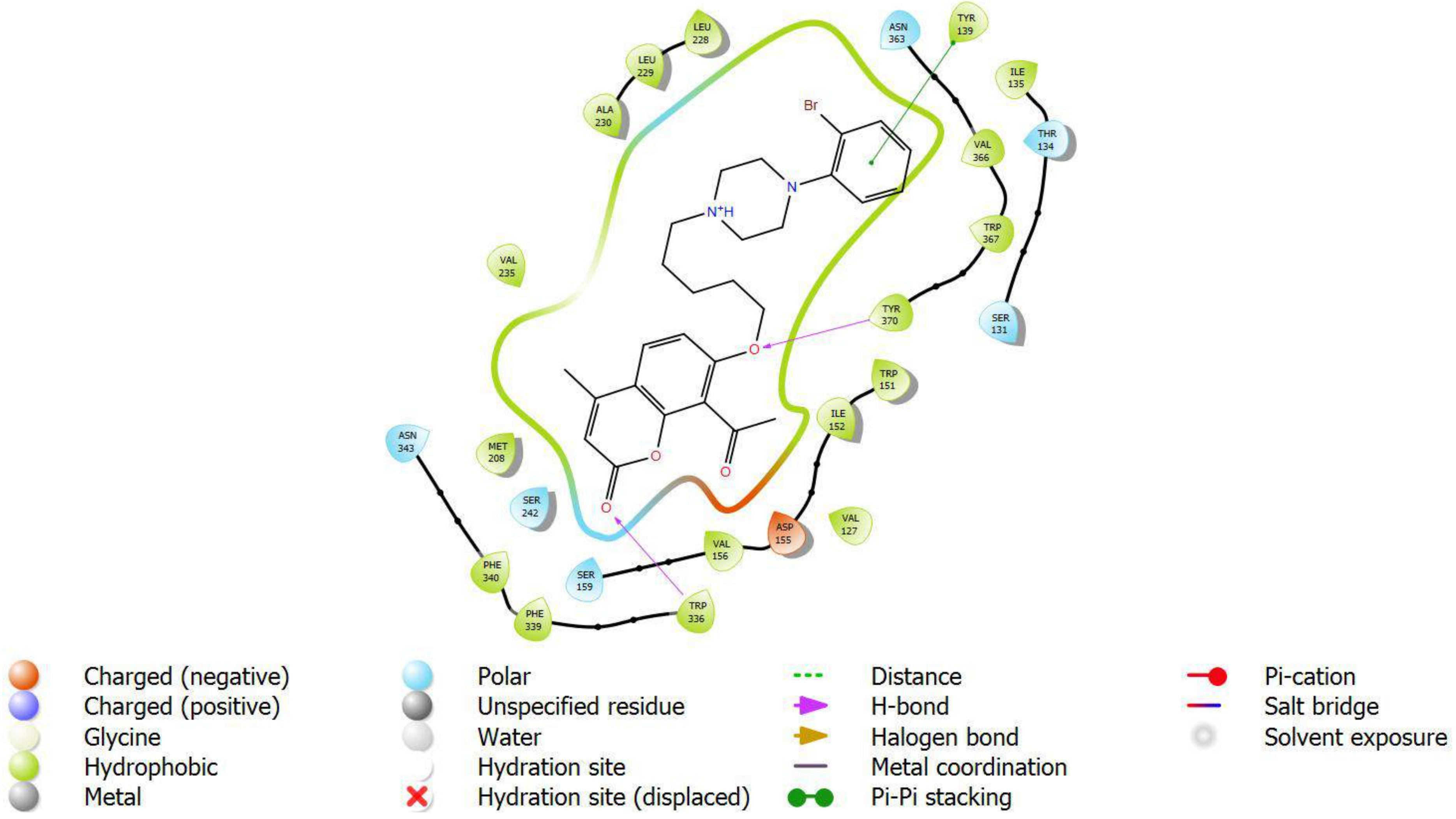

Figure S19. Ligand interaction diagram for compound **5f** docked to $5HT_{2A}$ receptor.

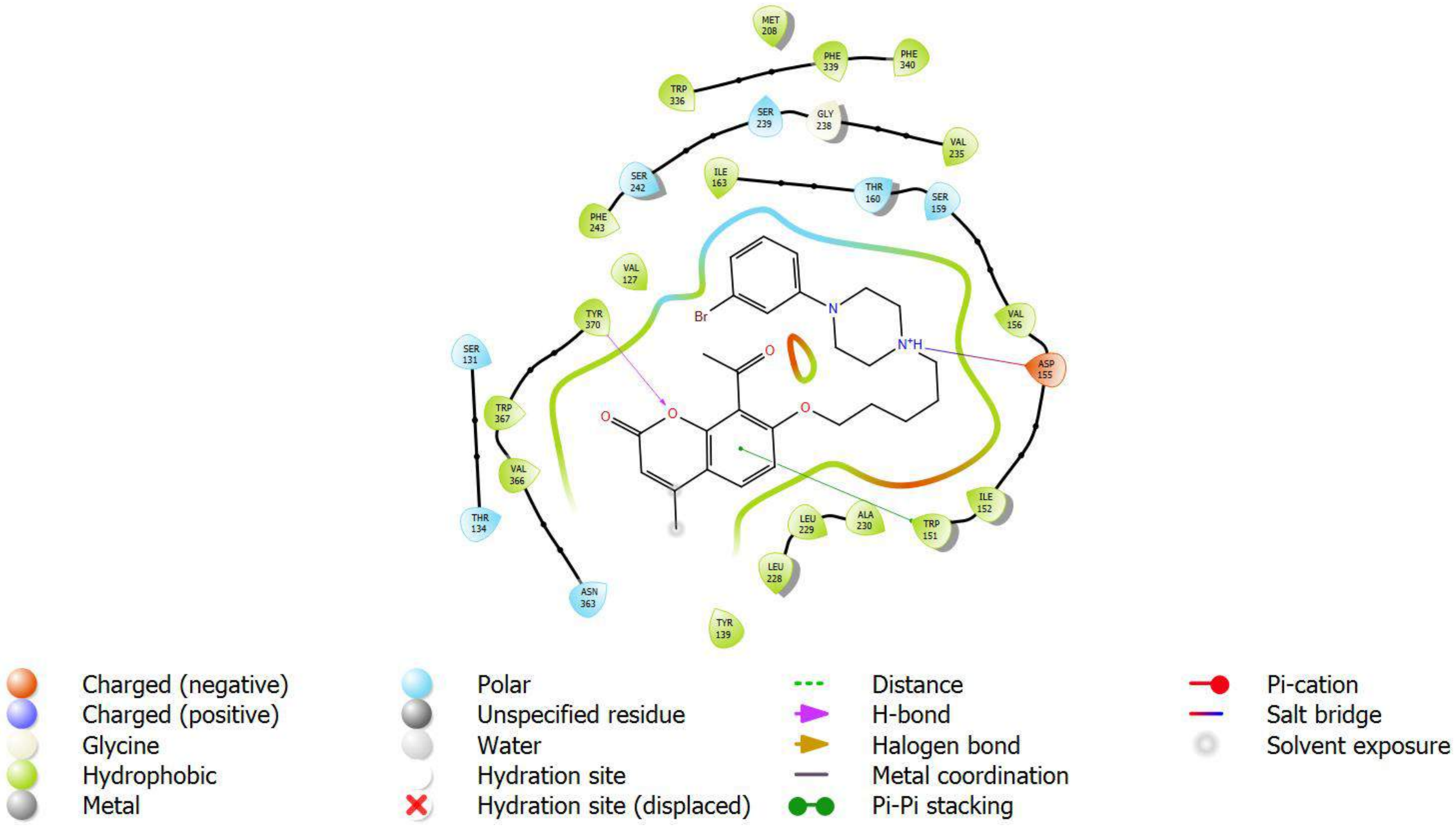

Figure S20. Ligand interaction diagram for compound **5g** docked to $5HT_{2A}$ receptor.

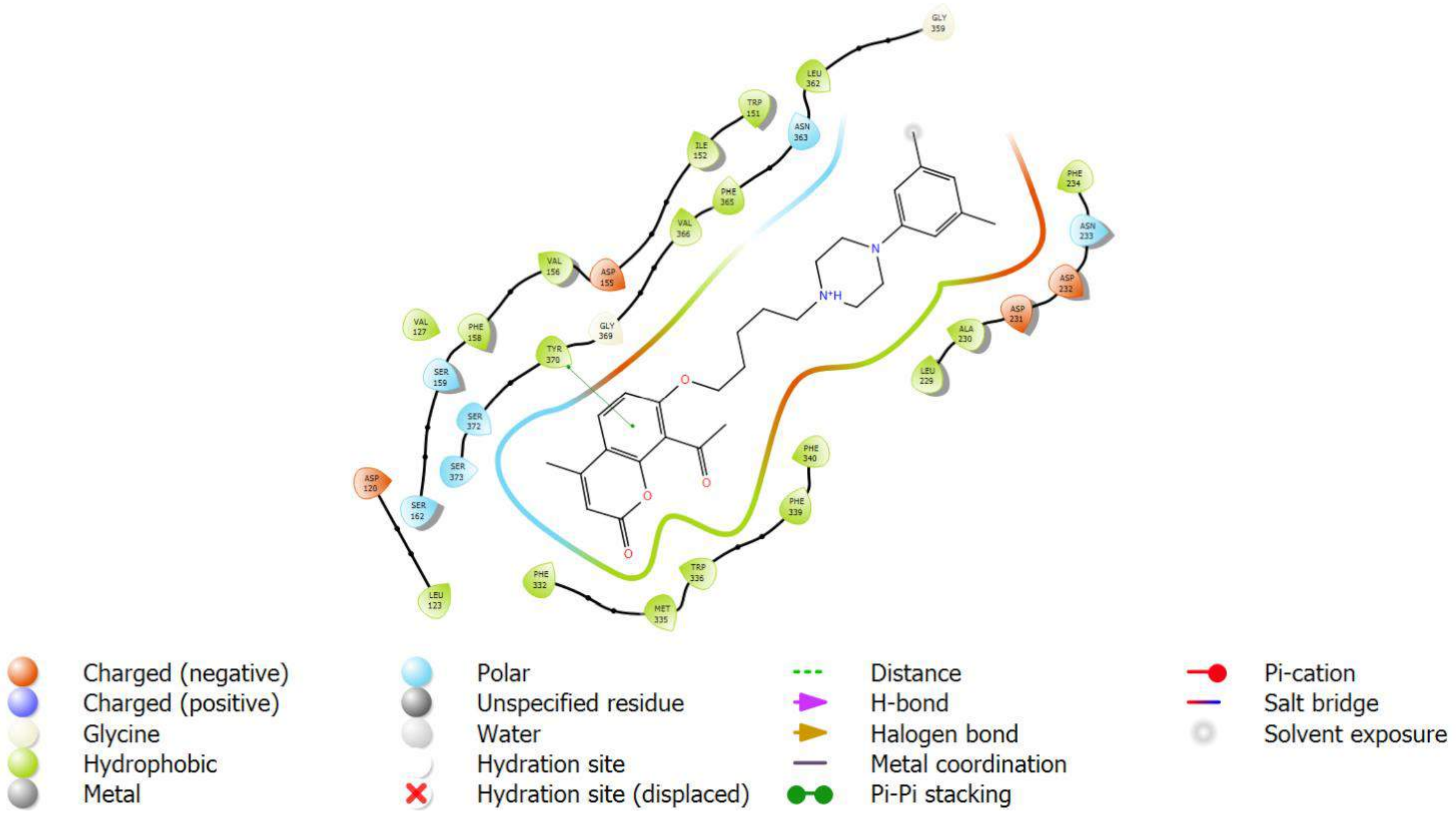

Figure S21. Ligand interaction diagram for compound **5h** docked to $5HT_{2A}$ receptor.
